# Supplementary material for: RNF144B negatively regulates antiviral immunity by targeting MDA5 for autophagic degradation
Source: EMBO Rep. 2024 Sep 16;25(10):4594–624. doi: 10.1038/s44319-024-00256-w (PMC11467429; doi:10.1038/s44319-024-00256-w)
Supplement: Supplementary file 1 — Appendix [file 44319_2024_256_MOESM1_ESM.pdf]

Appendix for:

# RNF144B negatively regulates antiviral immunity by targeting MDA5 for autophagic degradation

Contents:

Appendix Table S1 ........ 2

Appendix Table S2 ........ 4

Appendix Table S3 ........ 7

Appendix Table S1: Sequence for shRNA or siRNA assays

| Gene name | ShRNA sequence (5'-3') |
|---|---|
| shRNF144B-Human-F1 | CCGGCGGGTTTATATCGAACGCAATCTCGAGATTGCGT TCGATATAAACCCGTTTTTG |
| shRNF144B-Human-R1 | AATTCAAAAACGGGTTTATATCGAACGCAATCTCGAG ATTGCGTTCGATATAAACCCG |
| shRNF144B-Human-F2 | CCGGCCCATGTATAATCTGTTGTGTCTCGAGACACAAC AGATTATACATGGGTTTTTG |
| shRNF144B-Human-R2 | AATTCAAAAACCCATGTATAATCTGTTGTGTCTCGAGA CACAACAGATTATACATGGG |
| shRNF144B-Human-F3 | CCGGGCTGAGATTGCCTGTTTGGTACTCGAGTACCAAA CAGGCAATCTCAGCTTTTTG |
| shRNF144B-Human-R3 | AATTCAAAAAGCTGAGATTGCCTGTTTGGTACTCGAGT ACCAAACAGGCAATCTCAGC |
| shp62-1F | CCGGGCAGATGAGAAAGATCGCCTTCTCGAGAAGGCG ATCTTTCTCATCTGCTTTTTG |
| shp62-1R | AATTCAAAAAGCAGATGAGAAAGATCGCCTT CTCGAGAAGGCGATCTTTCTCATCTGC |
| shp62-2F | CCGG CCGAATCTACATTAAAGAGAACTCGAG TTCTCTTTAATGTAGATTCGG TTTTTG |
| shp62-2R | AATTCAAAAA CCGAATCTACATTAAAGAGAACTCGAG TTCTCTTTAATGTAGATTCGG |
| shp62-3F | CCGG CCTCTGGGCATTGAAGTTGAT CTCGAG ATCAACTTCAATGCCCAGAGG TTTTTG |
| shp62-3R | AATTCAAAAA CCTCTGGGCATTGAAGTTGATCTCGAG ATCAACTTCAATGCCCAGAGG |
| shtollip-1F | CCGG GAACAAGGATGCCGCCATCAA CTCGAG TTGATGGCGGCATCCTTGTTC TTTTTG |
| shtollip-1R | AATTCAAAAA GAACAAGGATGCCGCCATCAA CTCGAG TTGATGGCGGCATCCTTGTTC |
| shtollip-2F | CCGG GGCAAAGTTGGCCAAGAATTA CTCGAG TAATTCTTGGCCAACTTTGCC TTTTTG |
| shtollip-2R | AATTCAAAAA GGCAAAGTTGGCCAAGAATTA CTCGAG TAATTCTTGGCCAACTTTGCC |
| shtollip-3F | CCGG TCGAGATCTTCGATGAGAGAG CTCGAG CTCTCTCATCGAAGATCTCGA TTTTTG |

| | |
|---|---|
| shtollip-3R | AATTCAAAAA TCGAGATCTTCGATGAGAGAG CTCGAG CTCTCTCATCGAAGATCTCGA |
| siFJX1(H)-746-F | GCGAGGCCCUGUCUUACUATT |
| siFJX1(H)-746-R | UAGUAAGACAGGGCCUCGCTT |
| siFJX1(H)-1088-F | GGCUCGUAAGCAACCUCUUTT |
| siFJX1(H)-1088-R | AAGAGGUUGCUUACGAGCCTT |
| siFJX1(H)-1229-F | GCAUGUGGGACAAGUAUAATT |
| siFJX1(H)-1229-R | UUAUACUUGUCCCACAUGCTT |
| siZC3H12C(H)-186-F | CCAGCUGUACCUUGGUCAATT |
| siZC3H12C(H)-186-R | UUGACCAAGGUACAGCUGGTT |
| siZC3H12C(H)-1564-F | CCAGGUCUGUACCUUCCUUTT |
| siZC3H12C(H)-1564-R | AAGGAAGGUACAGACCUGGTT |
| siZC3H12C(H)-2254-F | CCACGAGAAUAGACAGCAUTT |
| siZC3H12C(H)-2254-R | AUGCUGUCUAUUCUCGUGGTT |
| siRNF144B(H)-532-F | CCAUCACUUGCCCUGACAUTT |
| siRNF144B(H)-532-R | AUGUCAGGGCAAGUGAUGGTT |
| siRNF144B(H)-976-F | GGUACUGCCUCCAGAACUUTT |
| siRNF144B(H)-976-R | AAGUUCUGGAGGCAGUACCTT |
| siRNF144B(H)-1112-F | GGGCAUCAUUGCCUUGGUUTT |
| siRNF144B(H)-1112-R | AACCAAGGCAAUGAUGCCCTT |
| siMDA5(M)-F | CCUACAAAUCAACGACACGTT |
| siMDA5(M)-R | CGUGUCGUUGAUUUGUAGGTT |
| siCCDC50-F | GGAACAAGAGAUUGAGCAUTT |
| siCCDC50-R | AUGCUCAAUCUCUUGUUCCTT |

Appendix Table S2: Primers for Real-Time PCR assays

| Gene name | Primer sequence (5'→3') |
|---|---|
| GAPDH HF | GAGTCAACGGATTTGGTCGT |
| GAPDH HR | GACAAGCTTCCCGTTCTCAG |
| IFNB1 HF | CACGACAGCTCTTTCCATGA |
| IFNB1 HR | AGCCAGTGCTCGATGAATCT |
| ISG15 HF | ATGGGCTGGGACCTGACGG |
| ISG15 HR | TTAGCTCCGCCCGCCAGGCT |
| ISG56 HF | ACGGCTGCCTAATTTACAGC |
| ISG56 HR | AGTGGCTGATATCTGGGTGC |
| Hrnf144bRTF | CTGGTAGGCTCCACTATCTCG |
| Hrnf144bRTR | GGGCAAGTGATGGGAGACC |
| Human IL-1β-F | CACGATGCACCTGTACGATCA |
| Human IL-1β-R | GTTGCTCCATATCCTGTCCCT |
| Human IL-6-F | GTACATCCTCGACGGCATCTCA |
| Human IL-6-R | GCACAGCTCTGGCTTGTTCCTC |
| Human TNF-α-F | TCTCGAACCCCGAGTGACA |
| Human TNF-α-R | GCCCGGCGGTTCA |
| mGAPDH-F | AGGTCGGTGTGAACGGATTTG |
| mGAPDH-R | TGTAGACCATGTAGTTGAGGTCA |
| mTNFa-F | AAGCCTGTAGCCCACGTCGTA |
| mTNFa-R | GGCACCACTAGTTGGTTGTCTTTG |
| mIL-6-F | TAGTCCTTCCTACCCCAATTTCC |
| mIL-6-R | TTGGTCCTTAGCCACTCCTTC |
| Mouse Ifnb1-F | GCCTTTGCCATCCAAGAGATGC |
| Mouse Ifnb1-R | ACACTGTCTGCTGGTGGAGTTC |
| Mouse *Isg15*-F | TGGTACAGAACTGCAGCGAG |
| Mouse *Isg15*-R | AGCCAGAACTGGTCTTCGTG |

| | |
|---|---|
| Mouse *Ifit1*-F | TGCTGAGATGGACTGTGAGGAA |
| Mouse *Ifit1*-R | TCTTGGCGATAGGCTACGACTG |
| Mouse *Ifit2*-F | CCTAAACAGTTACTCCACCTTCG |
| Mouse *Ifit2*-R | TTGCTGACCTCCTCCATTCT |
| Mouse *Ifit3*-F | TTCCCAGCAGCACAGAAAC |
| Mouse *Ifit3*-R | AAATTCCAGGTGAAATGGCA |
| Mouse *Mx1*-F | GTACGGTGCAGACATACCAG |
| Mouse *Mx1*-R | CGGTTTCCTGTGCTTGTATGA |
| mouse-RNF144B-F | AAAACCCACCCTCTGGAGAC |
| mouse-RNF144B-R | TTAGGCACACCATGTCAGGA |
| mACTIN-F | AGTGTGACGTTGACATCCGT |
| mACTIN-R | GCAGCTCAGTAACAGTCCGC |
| EMCV-F1 | GATGATTCGGGCGGTAGTGA |
| EMCV-R1 | TGGTTGGAAGACTTGACGGG |
| VSV-F | TGATAGTACCGGAGGATTGACGAC |
| VSV-R | CCTTGCAGTGACATGACTGCTCTT |
| EDNRB-F2 | CTGGCCATTTGGAGCTGAGA |
| EDNRB-F2 | CCAGAACCACAGAGACCACC |
| ATP10A-F2 | GCCGAATCCTCCCTGGAAAA |
| ATP10A-R2 | GGTGGCATTCAGGGTGATGA |
| ARRDC3-F2 | ATGGTGCTGGGAAAGGTGAAG |
| ARRDC3-R2 | GCTTTCACCCAATAGCGCAC |
| PMAIP1-F1 | CGAGGAACAAGTGCAAGTAGC |
| PMAIP1-R1 | ACGTGCACCTCCTGAGAAAA |
| FJX1-F2 | ACTACCTGACGGCCAACTTC |
| FJX1-R2 | GCAACAGCGGCTCGTTATAC |
| STX11-F1 | AACTGCAAGATCCGCATCCA |
| STX11-R1 | CTCTCGATCTCGTTGAGGGC |

| | |
|---|---|
| MX1-F2 | TGTGTCATTCCACCCAGAGC |
| MX1-R2 | CATCGCCGTTTGCTGAAACA |
| TNF-F2 | CTGGGCAGGTCTACTTTGGG |
| TNF-R2 | CTGGAGGCCCCAGTTTGAAT |
| SOCS1-F1 | AGCTCCTTCCCCTTCCAGAT |
| SOCS1-R1 | GGGTACCCACATGGTTCCAG |
| IDO1-F2 | AGGACATGCTGCTCAGTTCC |
| IDO1-R2 | CAGGCCAGCATCACCTTTTG |
| CCL2-F2 | AGCAGCAAGTGTCCCAAAGA |
| CCL2-R2 | GGTGTCTGGGGAAAGCTAGG |
| IL-1A-F2 | TTGGCGTTTGAGTCAGCAAA |
| IL-1A-R2 | CATGGAGTGGGCCATAGCTT |
| TNFAIP3-F1 | AGAGAGATCACACCCCCAGC |
| TNFAIP3-R1 | GTGCTCTCCAACACCTCTCC |
| TNFSF10-F2 | AGTCAAGTGGCAACTCCGTC |
| TNFSF10-R2 | GAGCTGCTACTCTCTGAGGAC |
| FOS-F2 | GGGGCAAGGTGGAACAGTTA |
| FOS-R2 | TCCTTCAGCAGGTTGGCAAT |
| NFKBIZ-F2 | GGTCAGACGGCGAGTTCTT |
| NFKBIZ-R2 | TCGAGAGTTCAGCATCAGGC |
| ZC3H12C-F2 | GGCTATGCCGGGTGGC |
| ZC3H12C-R2 | AACTGTGGGTCAGTGCTCTC |
| PARP14-F1 | GTTCAGCGCCTCACGAAATC |
| PARP14-R1 | GGAAGAGAAACAGCGTGCCT |
| EGR1-F2 | CACCTGACCGCAGAGTCTTT |
| EGR1-R2 | CTGACCAAGCTGAAGAGGGG |
| BATF2-F1 | GGTTGCTGAATGGCTCCTGT |
| BATF2-R1 | GAGCAGGAGGCACAATCCAT |

| | |
|---|---|
| TNFAIP6-F1 | TCACCTACGCAGAAGCTAAGGC |
| TNFAIP6-R1 | TCCAACTCTGCCCTTAGCCATC |
| CD274-1F | TGGCATTTGCTGAACGCATTT |
| CD274-1R | TGCAGCCAGGTCTAATTGTTTT |

## Appendix Table S3: Primers for PCR assays

| Gene name | Primer sequence (5’→3’) |
|---|---|
| RNF144B-F | CCGGAATTCCGGCCACCATGGGCTCAGCTGGTAGGCTCCACTA TCT |
| RNF144B- R1 | ATAAGAATGCGGCCGCTTAGGTTGTGGATGGGTCGTGCTTTTTC TTCTT |
| RING1-C48A-F | AAGCCCGGTGCATCTTTTGCACAGCTTGCCTG |
| RING1-C48A-R' | AAAGATGCACCGGGCTTCCTGGAGTGTGGTCATCTTGT |
| RING1-C50A-F | ATGCCGGGCCATCTTTTGCACAGCTTGCCTGA |
| RING1-C50A-R | AAAAGATGGCCCGGCATTCCTGGAGTGTGGTC |
| RING1-C53A-F | CATCTTTGCCACAGCTTGCCTGAAACAGTACATG |
| RING1-C53A-R | AAGCTGTGGCAAAGATGCACCGGCATTCCTGG |
| RING1-C75A-F | ATCACTGCCCCTGACATGGTGTGCCTAAACCA |
| RING1-C75A-R | ATGTCAGGGGCAGTGATGGGAGATCCACATCCTT |
| RING1-C80A-F | ACATGGTGGCCCTAAACCACGGGACCCTGCAG |
| RING1-C80A-R | GTTTAGGGCCACCATGTCAGGGCAAGTGATGG |
| RING2-C193A-F | TAAGCAAGCCCCAGTTTGCCGGGTTTATATCG |
| RING2-C193A-R | AAACTGGGGCTTGCTTAATGGGGGCTTCTGCA |
| RING2-C196A-F | CAGTTGCCCGGGTTTATATCGAACGCAATGAA |
| RING2-C196A-R | ATAAACCCGGGCAACTGGGCATTGCTTAATGGG |
| RING2-C206A-F | AATGAAGGCGCCGCTCAGATGATGTGCAAAAACTG |
| RING2-C206A-R | TGAGCGGCGCCTTCATTGCGTTCGATATAAAC |
| RING2-C211A-F | TGATGGCCAAAAACTGCAAGCATACATTTTGC |

| | |
|---|---|
| RING2-C211A-R | GCAGTTTTTGGCCATCATCTGAGCGCAGCCTT |
| RING2-C214A-F | GCAAAAACGCCAAGCATACATTTTGCTGGTACTGC |
| RING2-C214A-R | ATGCTTGGCGTTTTTGCACATCATCTGAGCGC |
| RING2-C219A-F | ACATTTGCCTGGTACTGCCTCCAGAACTTGGA |
| RING2-C219A-R | CAGTACCAGGCAAATGTATGCTTGCAGTTTTTGCA |
| RING2-C222A-F | TACGCCCTCCAGAACTTGGACAATGACATTTT |
| RING2-C222A-R | AAGTTCTGGAGGGCGTACCAGCAAAATGTATGCTTGCA |
| MDA5-CARDs-F | CCCTCGAGGGGCCACCATGTCGAATGGGTATTCCACAGACGAG |
| MDA5-CARDs -R | AAGGAAAAAAGCGGCCGCTTAAACTTCCATTTGGTAAGGCCTG AGC |
| MDA5- ATP-F | GGGGTACCGCCACCATGGCCCAGCCAGCCTTGGAAGGG |
| MDA5-ATP-R: | CCCTCGAGTTCATTTTCATATTCTGGGTTTTCAGCCAG |
| MDA5- CTD-F | GGGGTACCGCCACCATGAAGCTGACCAAATTAAGAAAT |
| MDA5- CTD-R | CCCTCGAGATCCTCATCACTAAATAAACAGCA |
| MDA5-K23R-F | CAGGGTGAGAATGTACATCCAGGTGGAGCCTG |
| MDA5-K23R-R | TGTACATTCTCACCCTGGCCCTGAAGCACGAG |
| MDA5-K43R-F | AGAGGTGAGAGAGCAGATTCAGAGGACAGTCGC |
| MDA5-K43R-R | AGAGGTGAGAGAGCAGATTCAGAGGACAGTCGC |
| MDA5-K68R-F | CTTGGAGAGAGGAGTCTGGCACCTTGGTTGGA |
| MDA5-K68R-R | AGACTCCTCTCTCCAAGGTGCTCAGCAGCAGT |
| MDA5-K128R-F | GGTGGACAGACTTCTAGTTAGAGACGTCTTGGATAAGTG |
| MDA5-K128R-R | CTAGAAGTCTGTCCACCAGAGTGGGCTGAAGG |
| MDA5-K137R-F | CTTGGATAGATGCATGGAGGAGGAACTGTTGA |
| MDA5-K137R-R | CCATGCATCTATCCAAGACGTCTCTAACTAGAAGCTT |
| MDA5-K169R-F | GAGAGCTACTAAGAAGGATTGTGCAGAAAGAAAACTGG |
| MDA5-K169R-R | CCTTCTTAGTAGCTCTCTTACACCTGATTCATTTC |
| MDA5-K174R-F | GTGCAGAGAGAAAACTGGTTCTCTGCATTTCTG |
| MDA5-K174R-R | CAGTTTTCtctCTGCACAATCCTTTTTAGTAGCTCTC |

| | |
|---|---|
| miceF1 | AGAGCCAGATTTTGGATTTCTGC |
| miceR1 | TTCTTTCTGACTAGGGCTGGTACT |
| miceF2 | GAGCTTCATGAAAGATTAAGGCCAC |
| P62-F | CGGAATTCCGGCCACCATGCAGCGCCGGGACGACCCCGC |
| P62-R | GGGGTACCTTAATAACGTCCATATGGGTGCTCTC |
| BNIP3L-F | CGGAATTCCGGCCACCATGAACAGCAGCAATGGCAATGAT |
| BNIP3L-R | CCCTCGAGTCAGTAGGTGCTGGCAGAGGGTG |
| TOLLIP-F | CGGAATTCCGGCCACCATGGCGACCACCGTCAGCACTCAGC |
| TOLLIP-R | GGGGTACCCTATGGCTCCTCCCCCATCTGCAGCAGG |
| OPTN-F | CGGAATTCCGGCCACCATGTCCCATCAACCTCTCAGCTGCCTCA |
| OPTN-R | CCCTCGAGTTAAATGATGCAATCCATCACGTGAA |
| NBR1-F | CCCTCGAGGGGCCACCATGGAACCACAGGTTACTCTAAATG |
| NBR1-R | AAGGAAAAAAGCGGCCGCTCAATAGCGTTGGCTGTACCAGTCG TT |
| K27/K33R-F | CGCAAAGATCCAAGACCGCGAAGGCATCCCTCCTGACCA |
| K27/K33R-R | GGTCTTGGATCTTTGCGCGGACATTCTCAATGGTGTCACTCGG |
