# Supplementary figures and images for: RNF144B negatively regulates antiviral immunity by targeting MDA5 for autophagic degradation (part 1 of 2)
Source: EMBO Rep. 2024 Sep 16;25(10):4594–624. doi: 10.1038/s44319-024-00256-w (PMC11467429; doi:10.1038/s44319-024-00256-w)

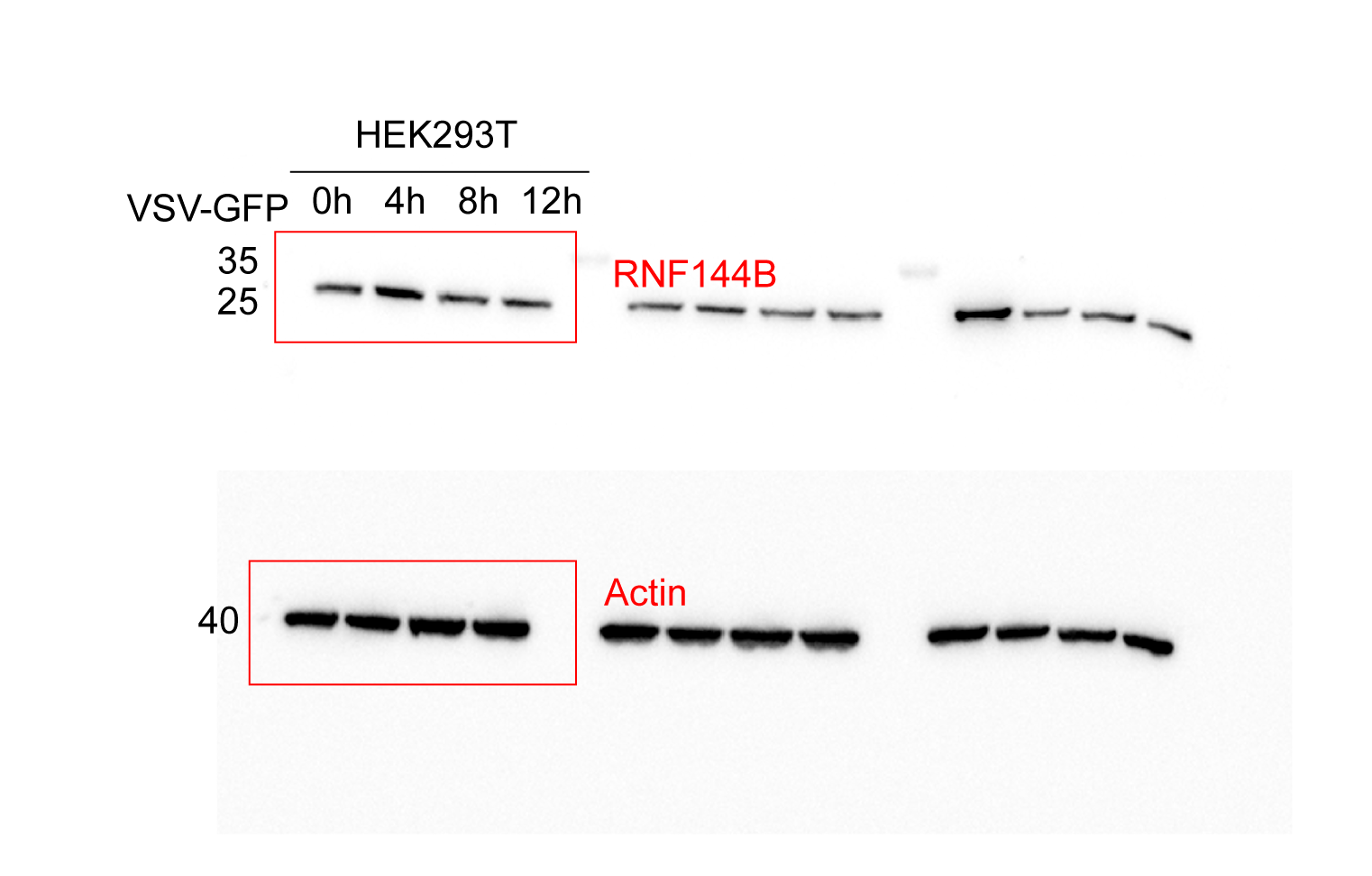

Supplement: Supplementary file 4 — Source data Fig. 1 [file 44319_2024_256_MOESM4_ESM.zip › SourceDateForFigure 1/1B.tif]

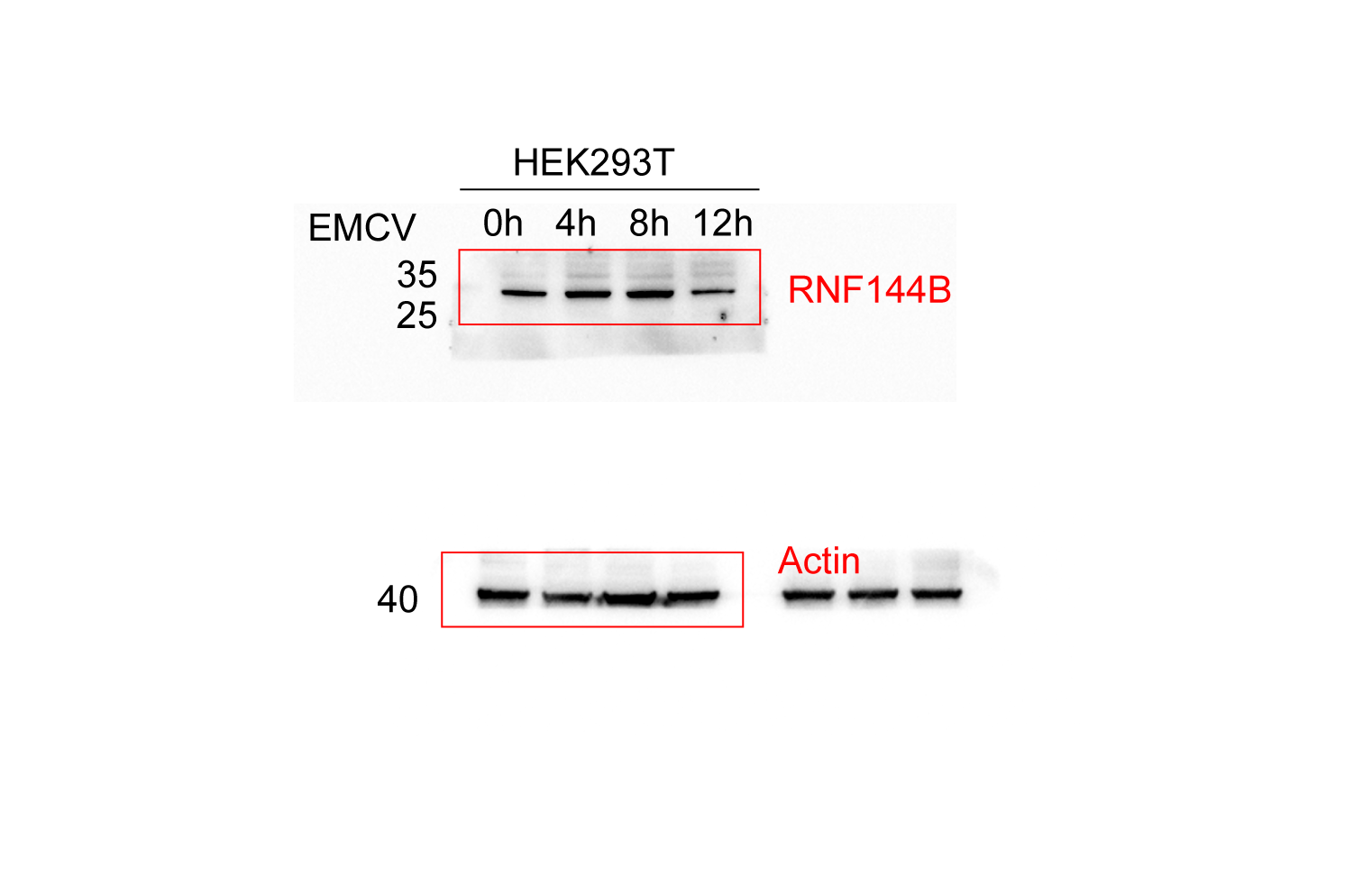

Supplement: Supplementary file 4 — Source data Fig. 1 [file 44319_2024_256_MOESM4_ESM.zip › SourceDateForFigure 1/1C.tif]

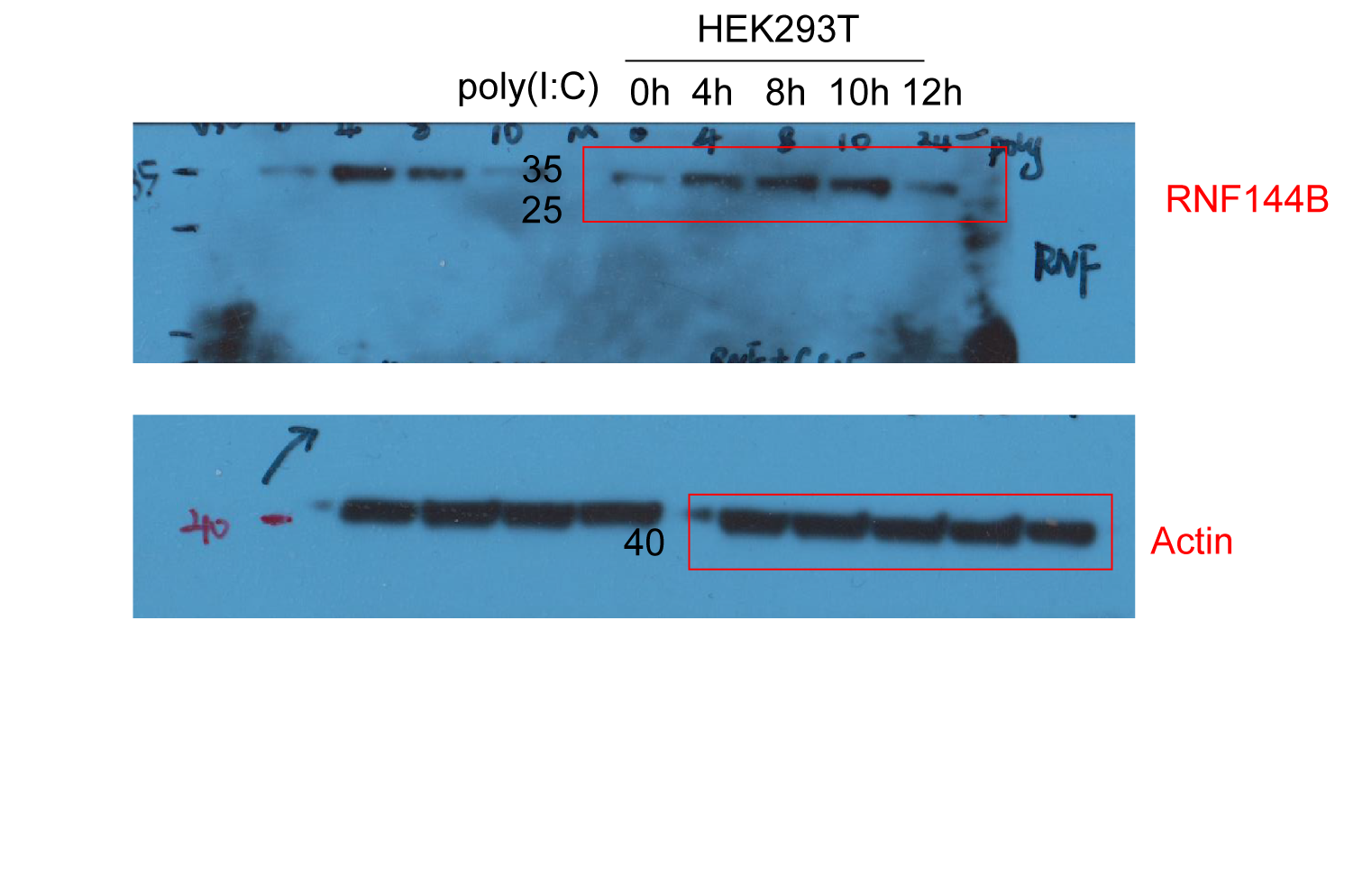

Supplement: Supplementary file 4 — Source data Fig. 1 [file 44319_2024_256_MOESM4_ESM.zip › SourceDateForFigure 1/1D.tif]

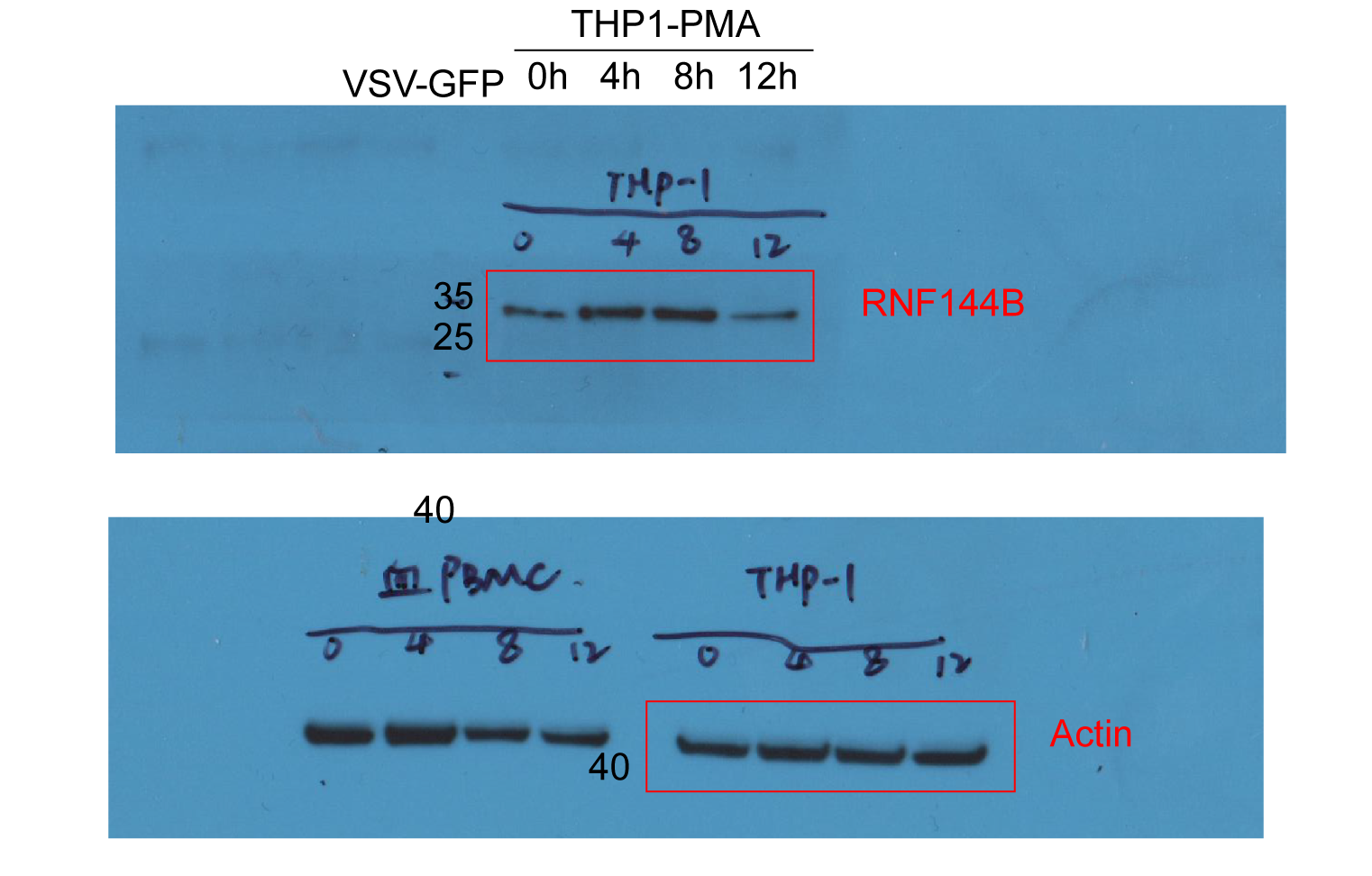

Supplement: Supplementary file 4 — Source data Fig. 1 [file 44319_2024_256_MOESM4_ESM.zip › SourceDateForFigure 1/1E.tif]

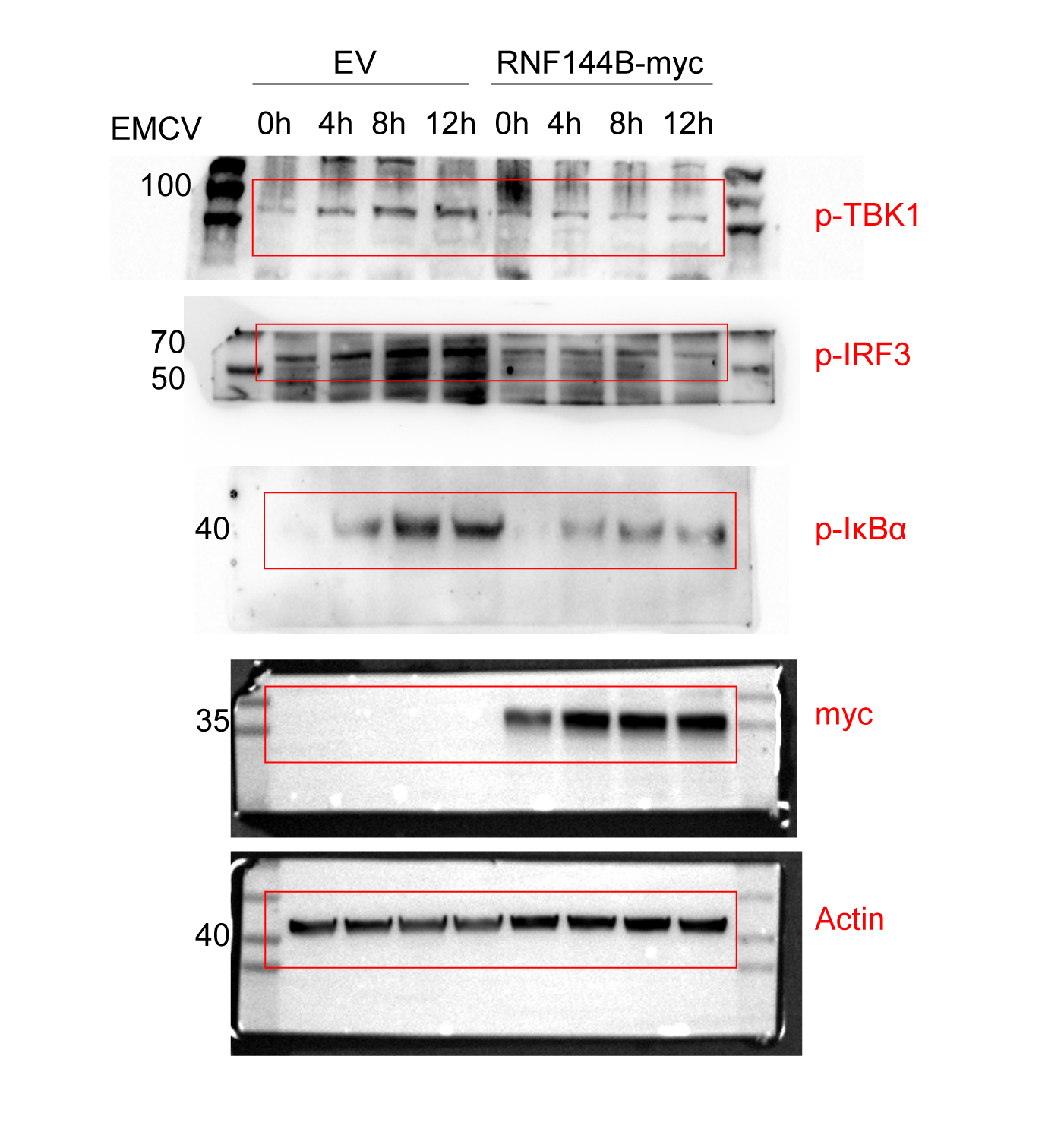

Supplement: Supplementary file 4 — Source data Fig. 1 [file 44319_2024_256_MOESM4_ESM.zip › SourceDateForFigure 1/1G.tif]

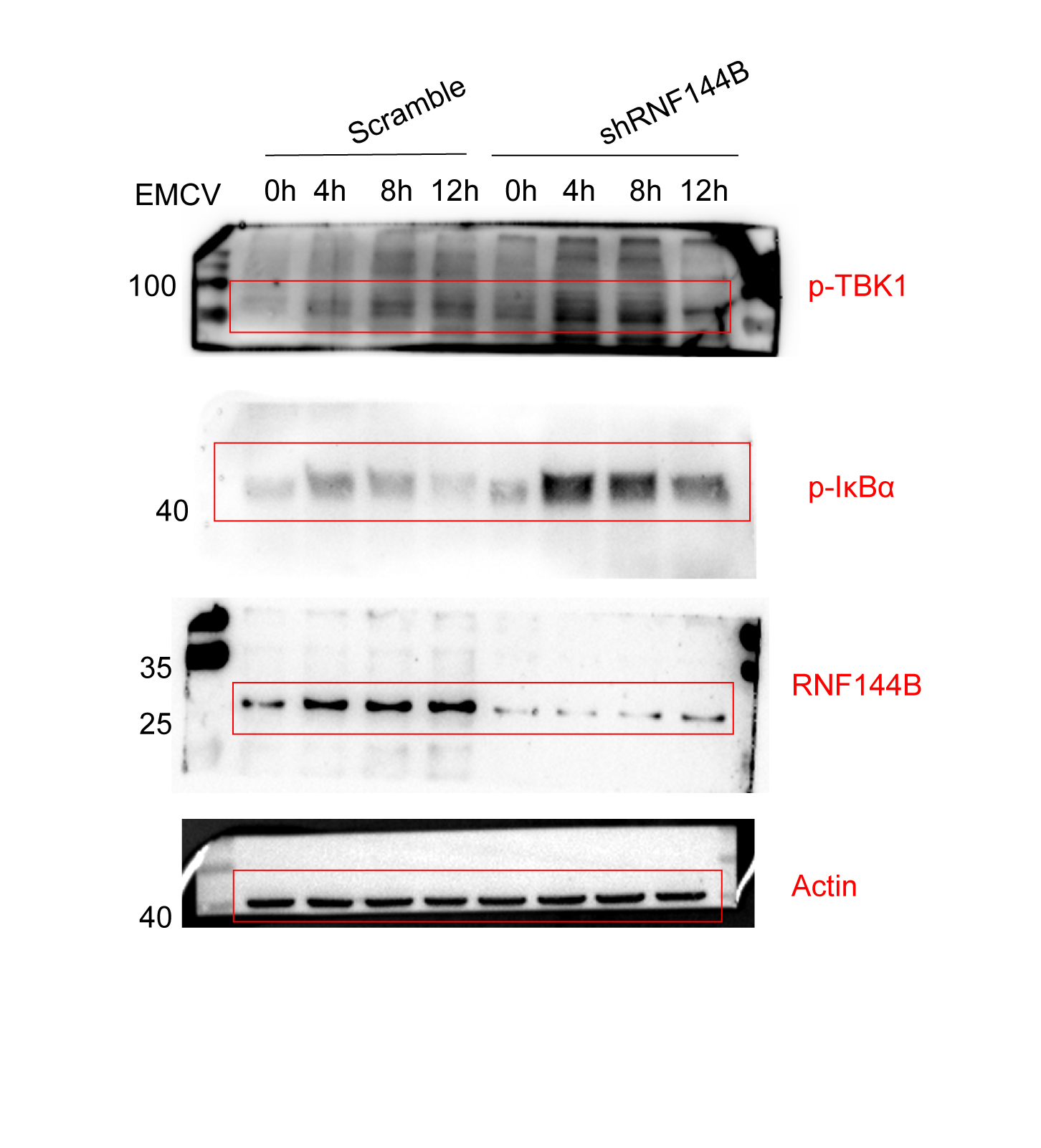

Supplement: Supplementary file 4 — Source data Fig. 1 [file 44319_2024_256_MOESM4_ESM.zip › SourceDateForFigure 1/1I.tif]

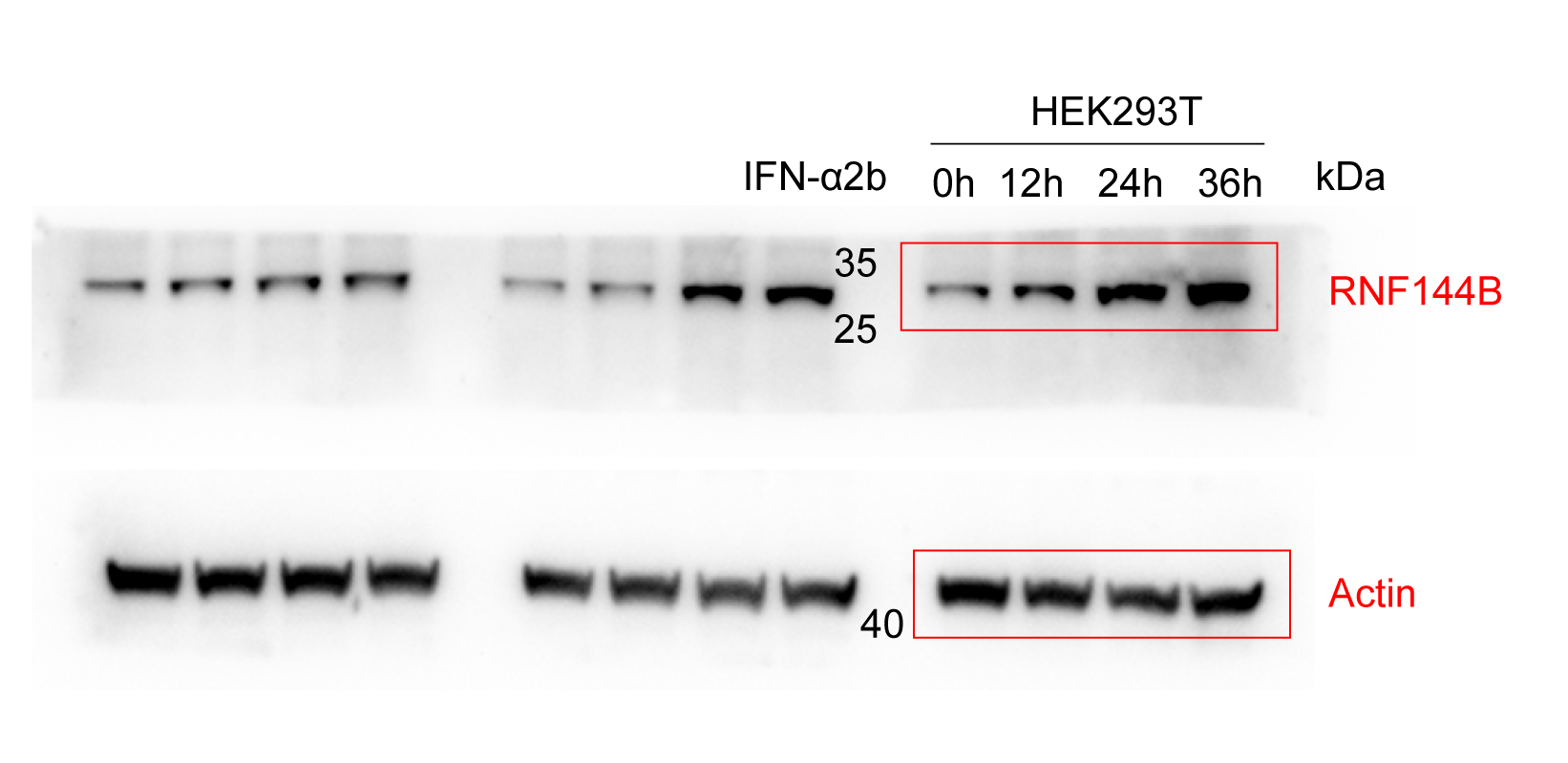

Supplement: Supplementary file 4 — Source data Fig. 1 [file 44319_2024_256_MOESM4_ESM.zip › SourceDateForFigure 1/1L.tif]

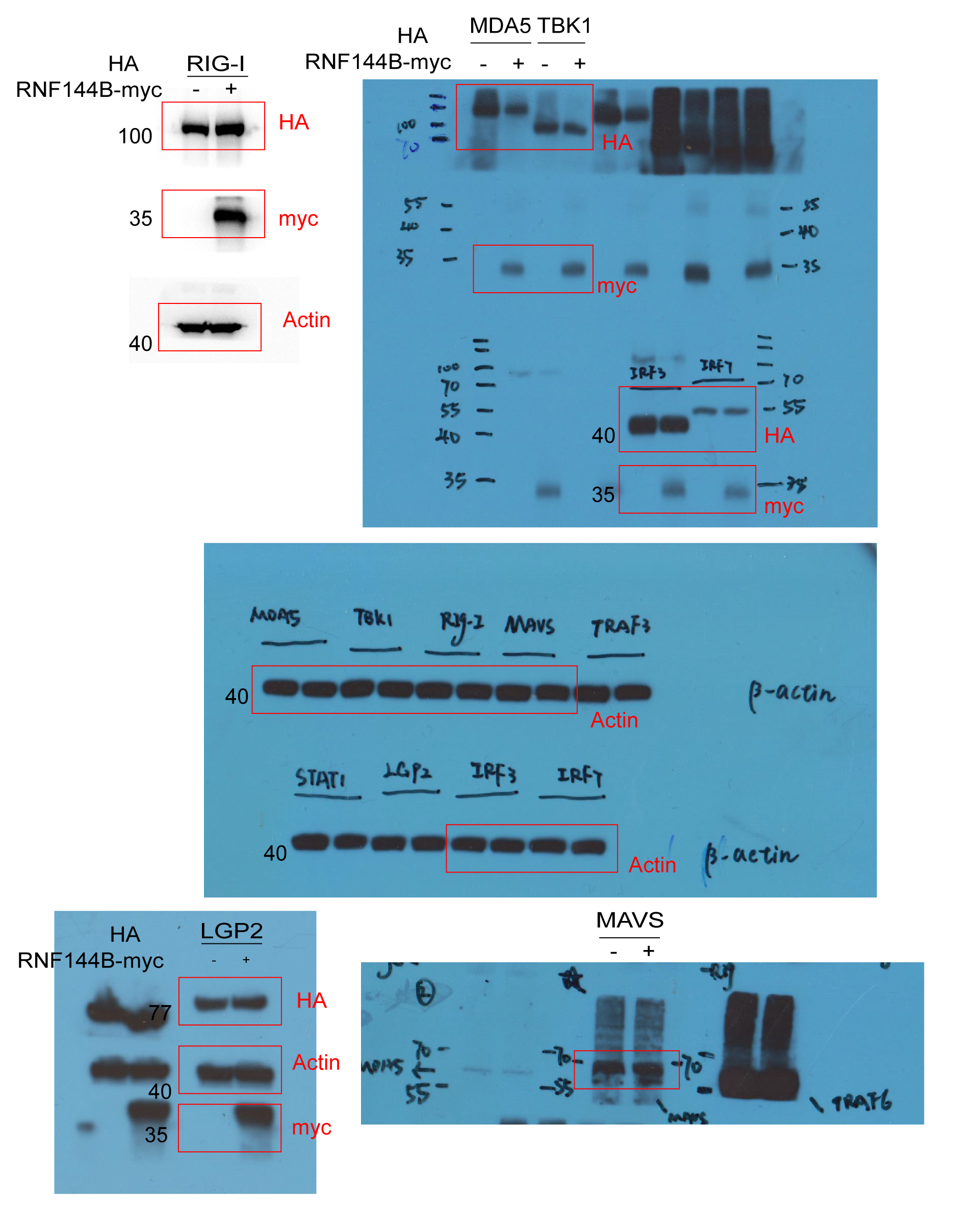

Supplement: Supplementary file 5 — Source data Fig. 2 [file 44319_2024_256_MOESM5_ESM.zip › SourceDateForFigure 2/2A.tif]

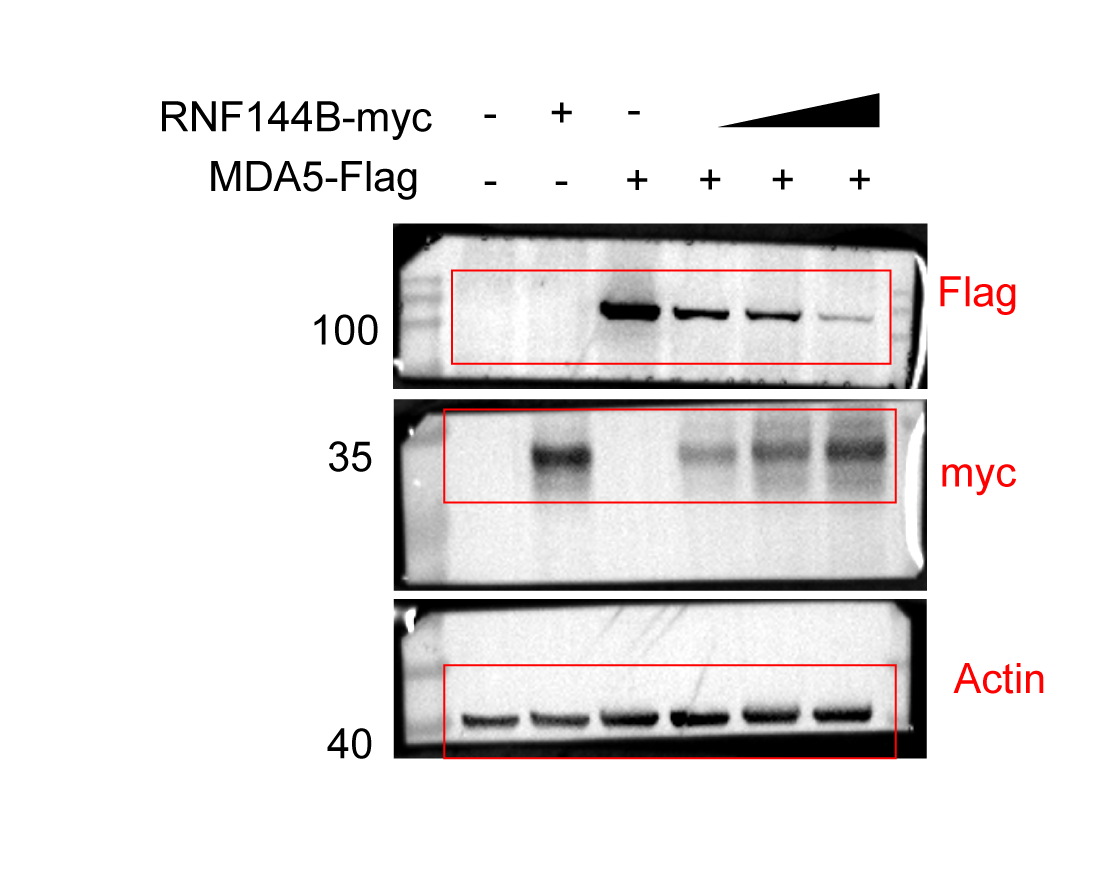

Supplement: Supplementary file 5 — Source data Fig. 2 [file 44319_2024_256_MOESM5_ESM.zip › SourceDateForFigure 2/2B.tif]

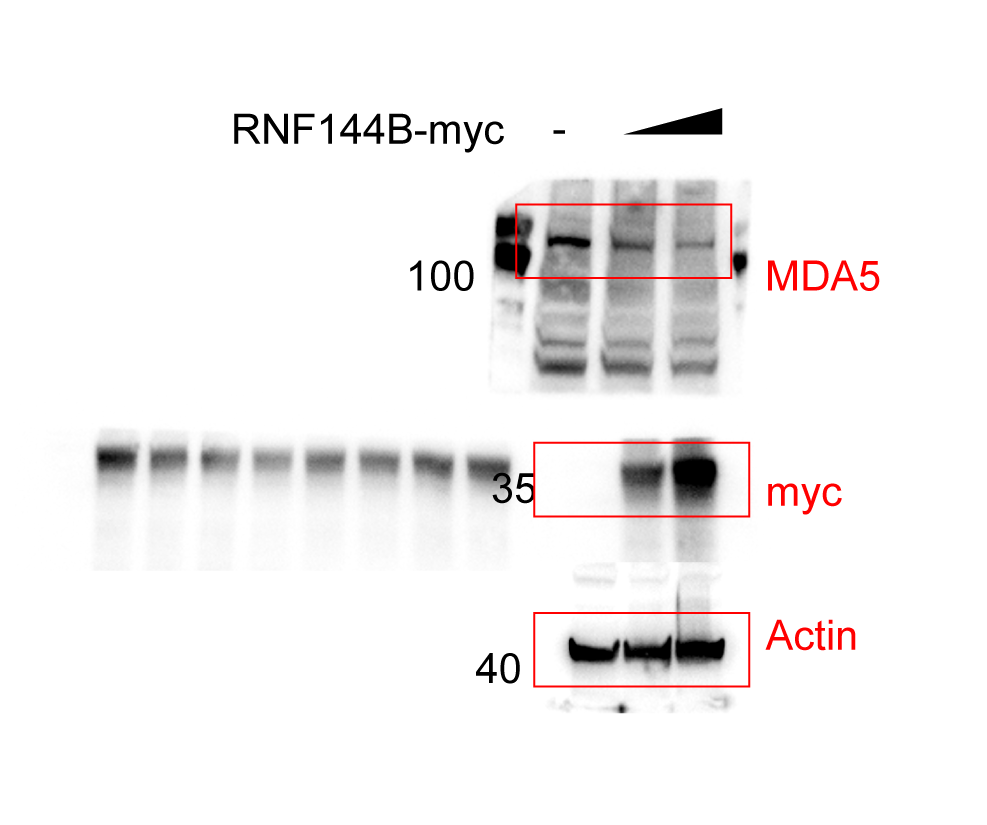

Supplement: Supplementary file 5 — Source data Fig. 2 [file 44319_2024_256_MOESM5_ESM.zip › SourceDateForFigure 2/2C.tif]

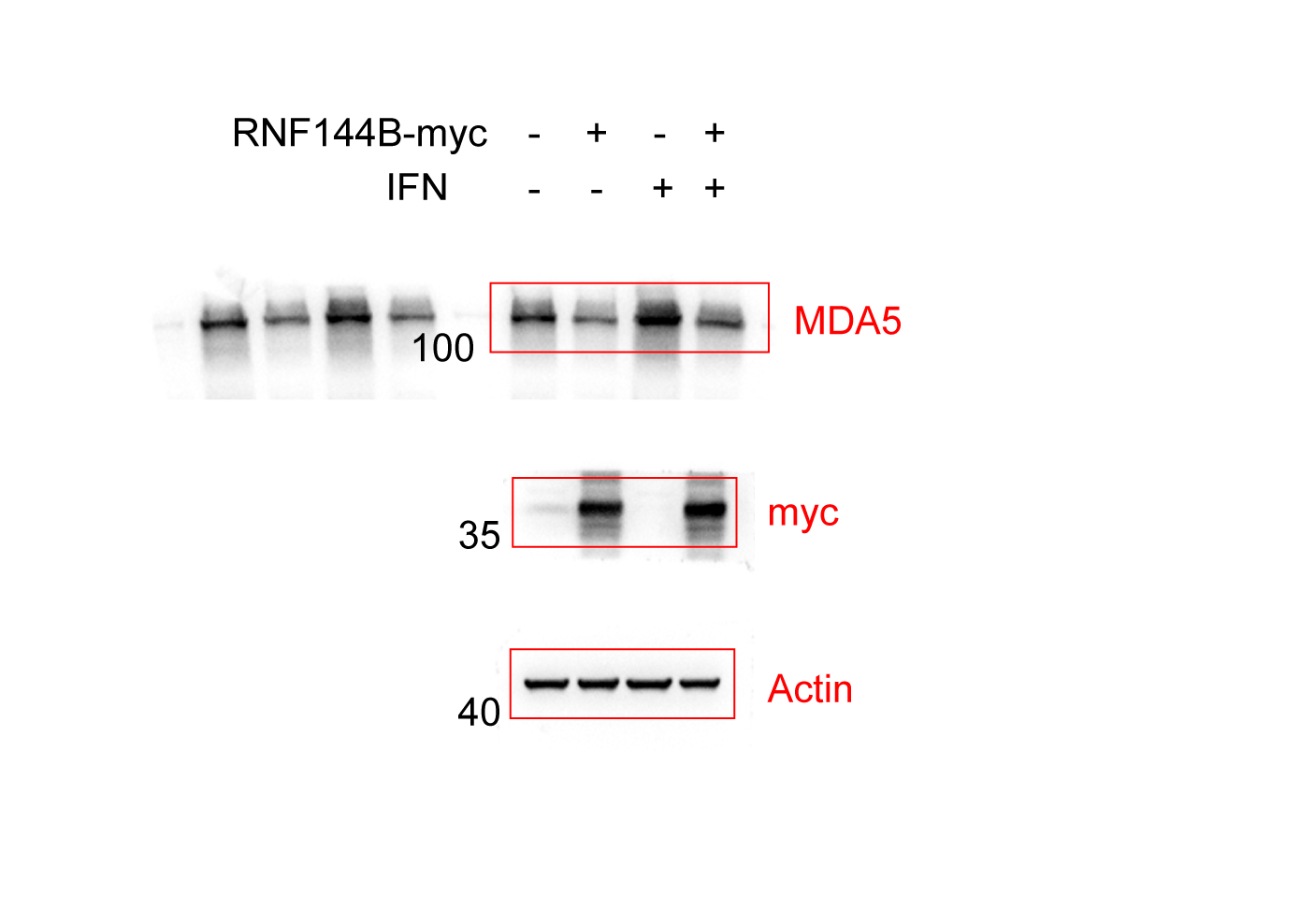

Supplement: Supplementary file 5 — Source data Fig. 2 [file 44319_2024_256_MOESM5_ESM.zip › SourceDateForFigure 2/2D.tif]

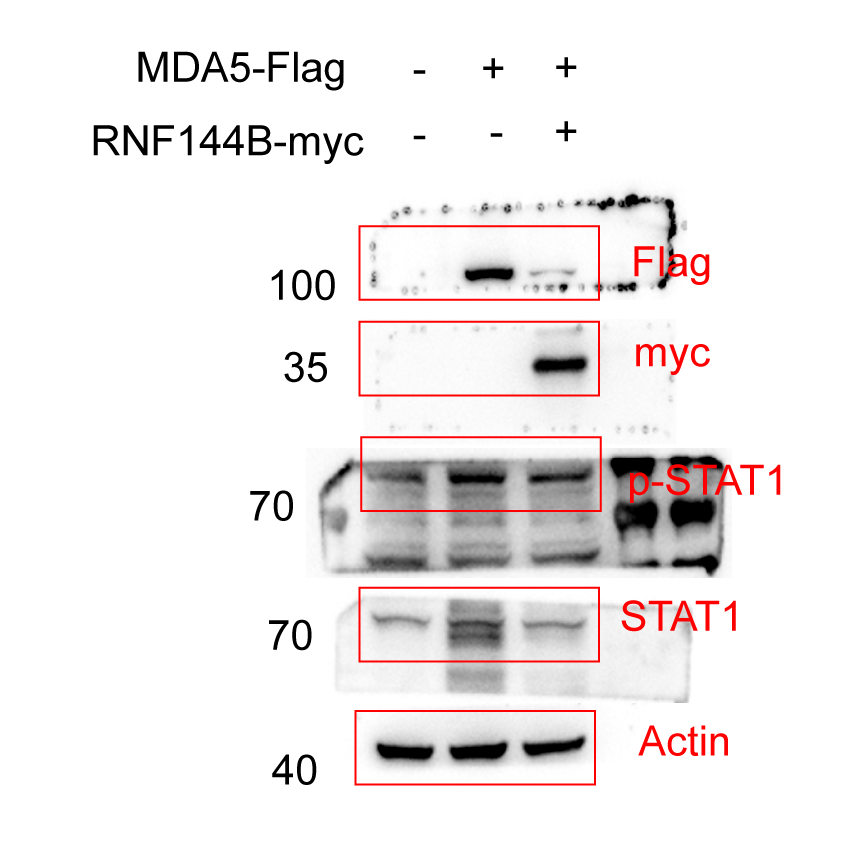

Supplement: Supplementary file 5 — Source data Fig. 2 [file 44319_2024_256_MOESM5_ESM.zip › SourceDateForFigure 2/2E.tif]

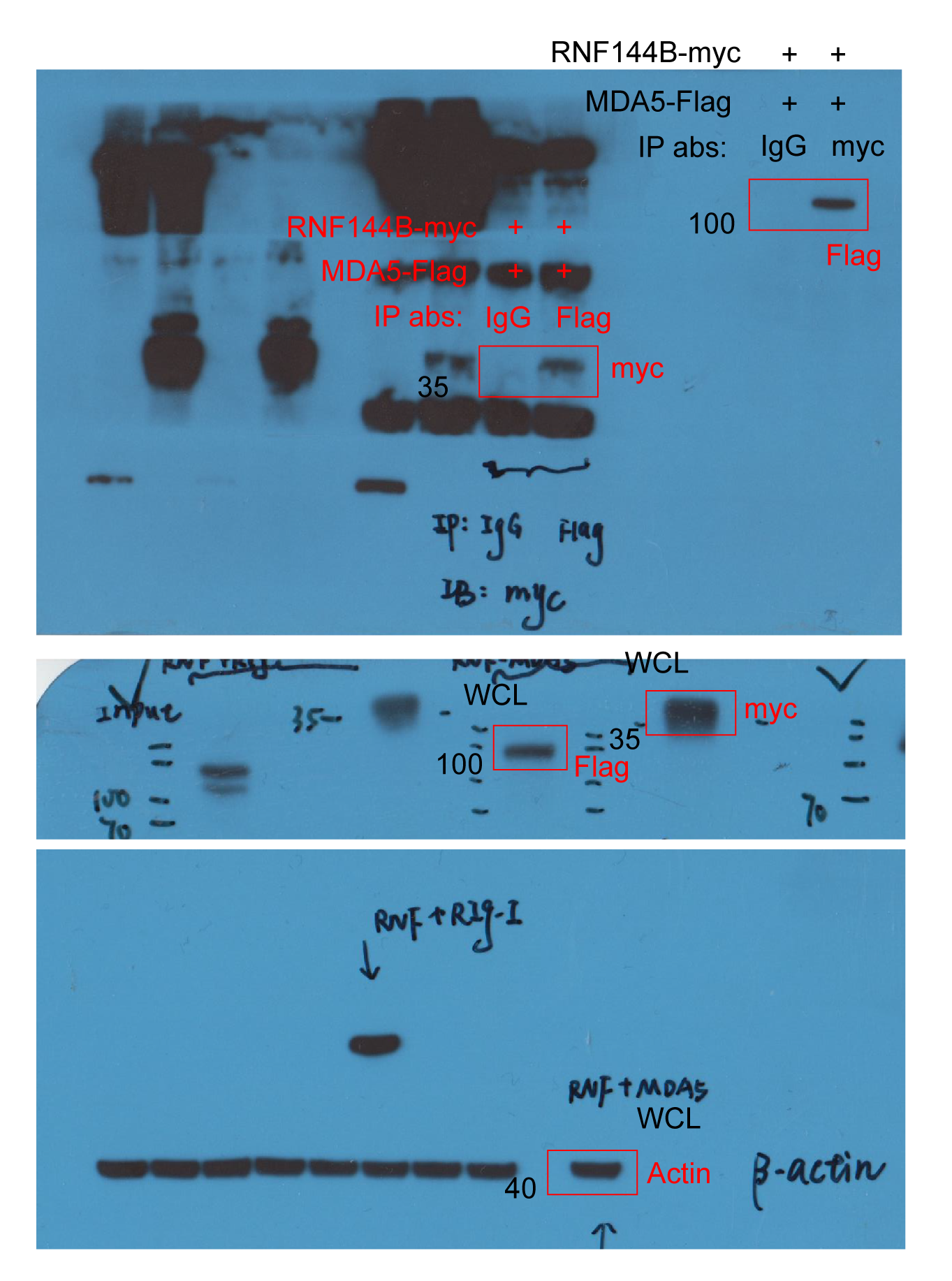

Supplement: Supplementary file 5 — Source data Fig. 2 [file 44319_2024_256_MOESM5_ESM.zip › SourceDateForFigure 2/2F.tif]

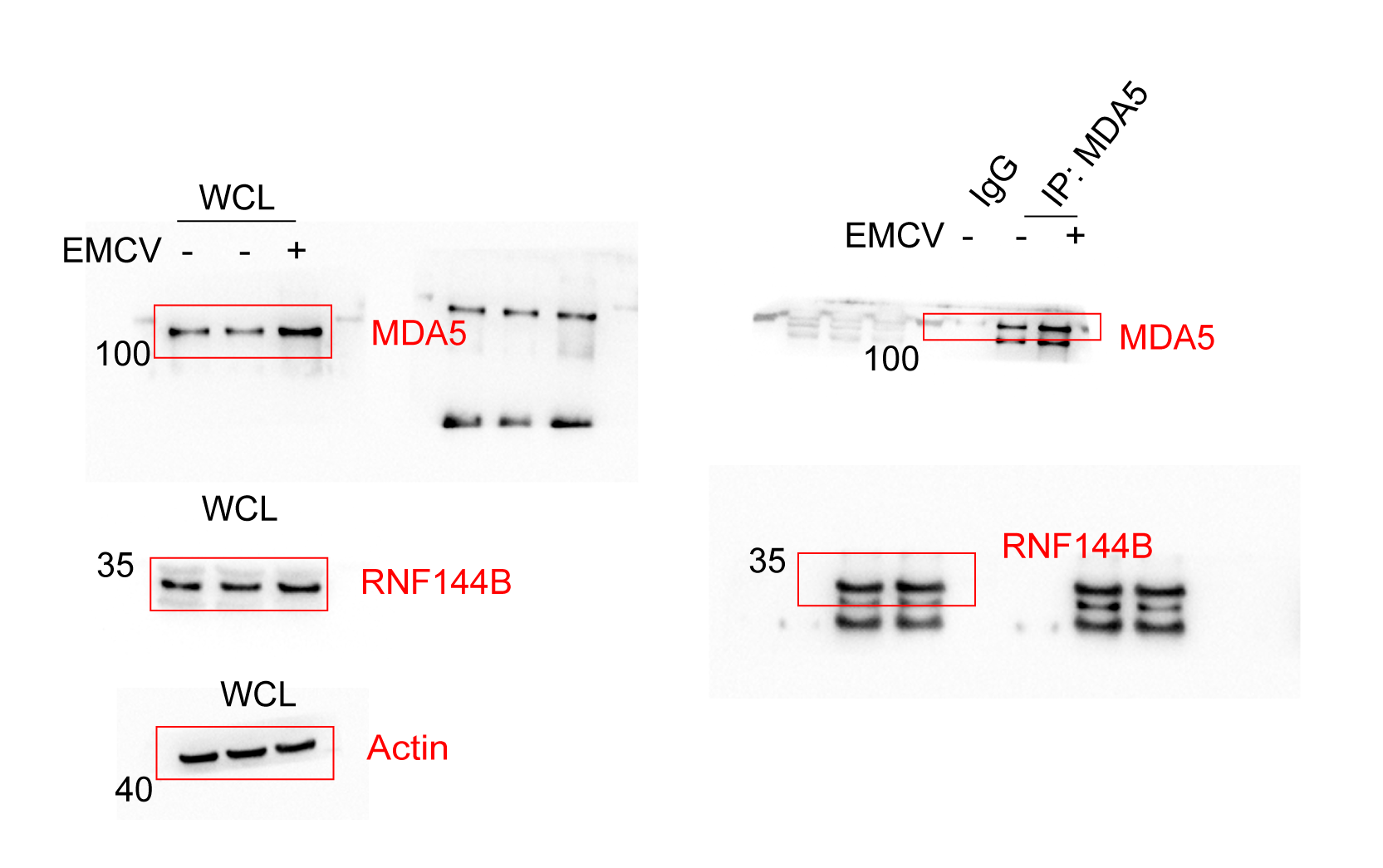

Supplement: Supplementary file 5 — Source data Fig. 2 [file 44319_2024_256_MOESM5_ESM.zip › SourceDateForFigure 2/2G.tif]

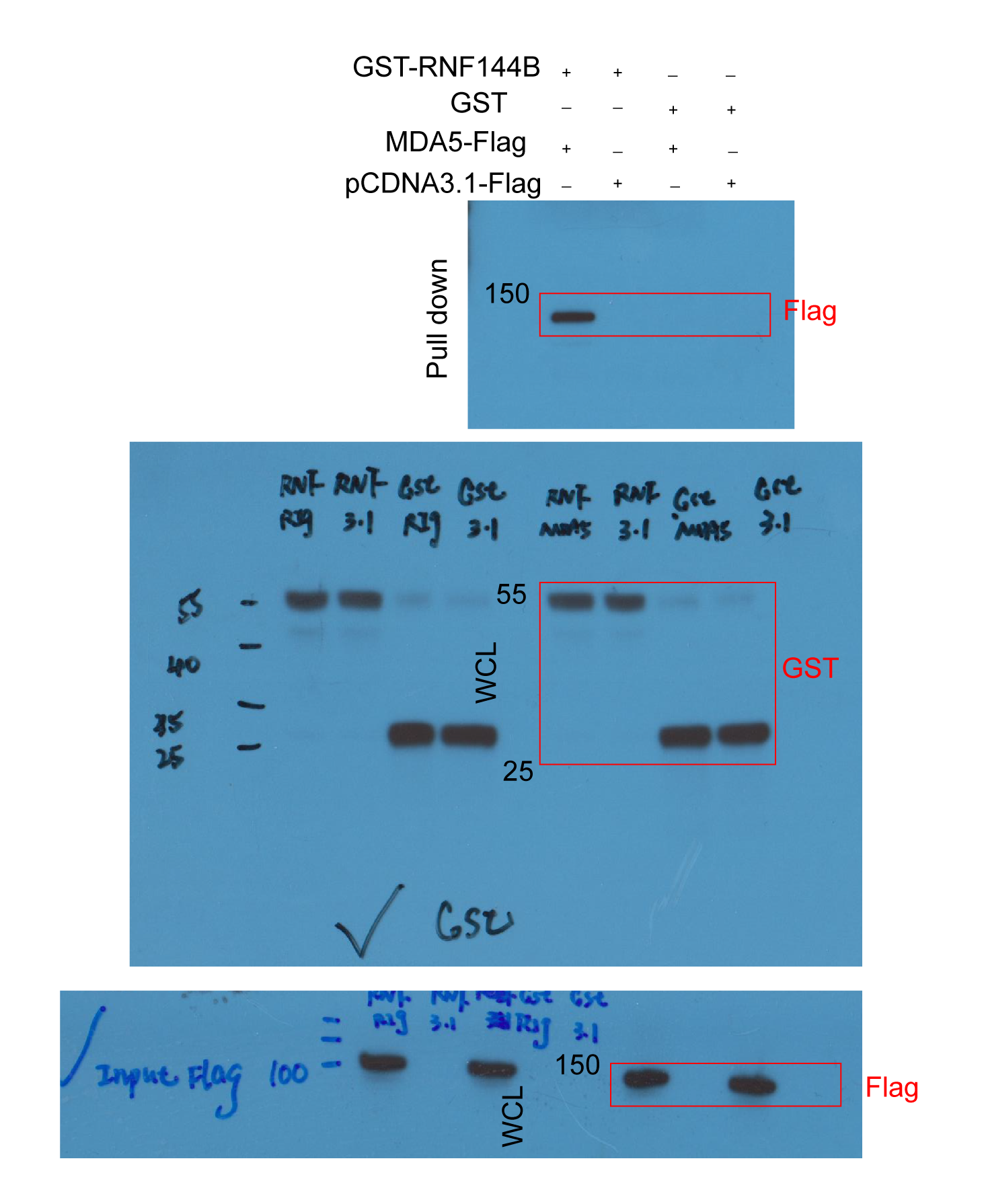

Supplement: Supplementary file 5 — Source data Fig. 2 [file 44319_2024_256_MOESM5_ESM.zip › SourceDateForFigure 2/2H.tif]

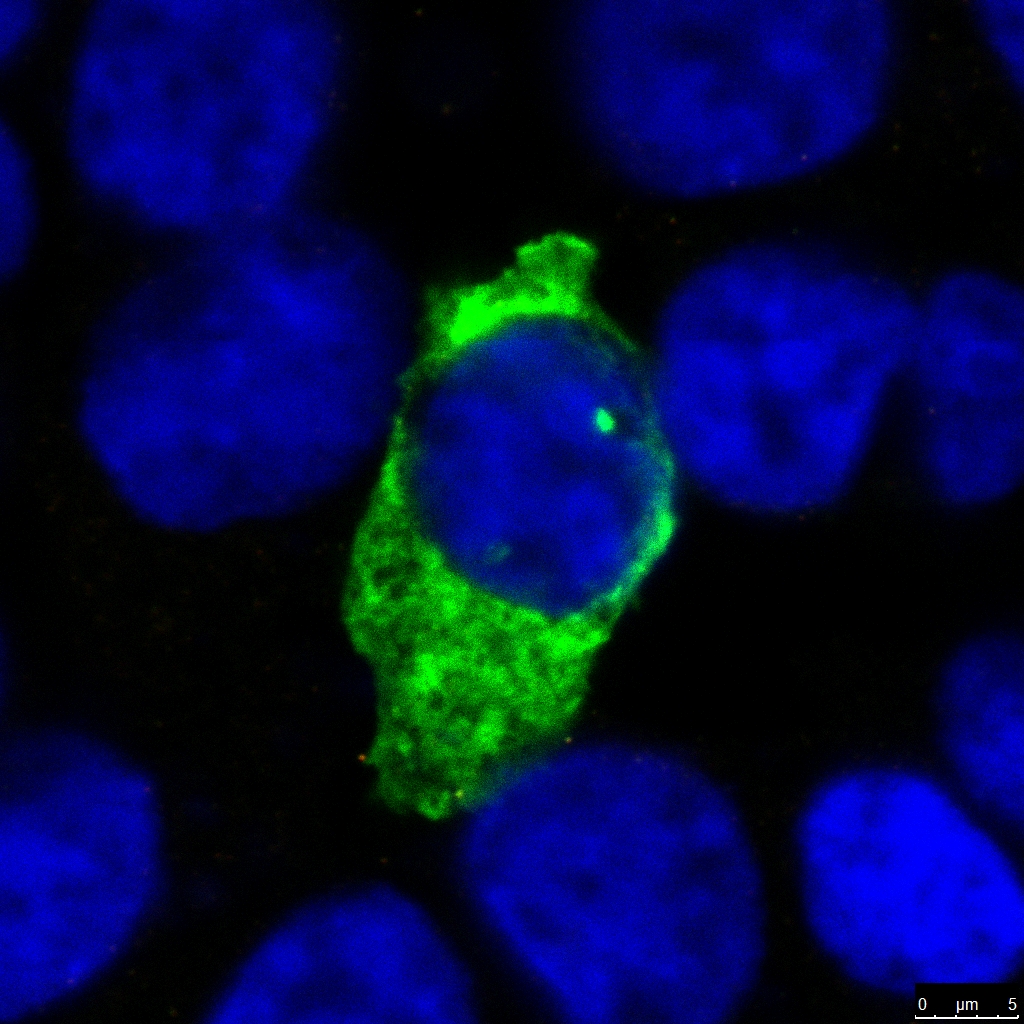

Supplement: Supplementary file 5 — Source data Fig. 2 [file 44319_2024_256_MOESM5_ESM.zip › SourceDateForFigure 2/2I/Experiment.lif_plvx-mda5-1-FIG-2I/Experiment.lif_plvx-mda5-1_z0.jpg]

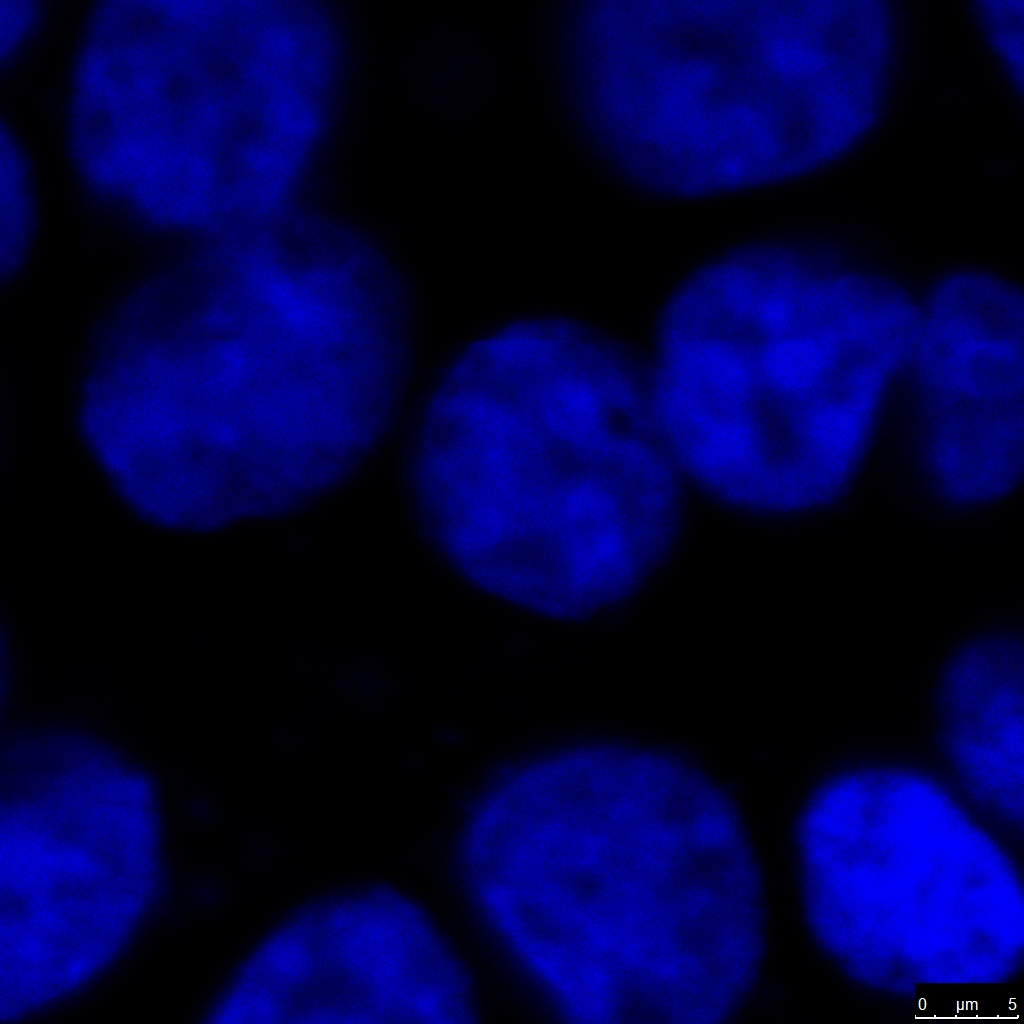

Supplement: Supplementary file 5 — Source data Fig. 2 [file 44319_2024_256_MOESM5_ESM.zip › SourceDateForFigure 2/2I/Experiment.lif_plvx-mda5-1-FIG-2I/Experiment.lif_plvx-mda5-1_z0_ch00.jpg]

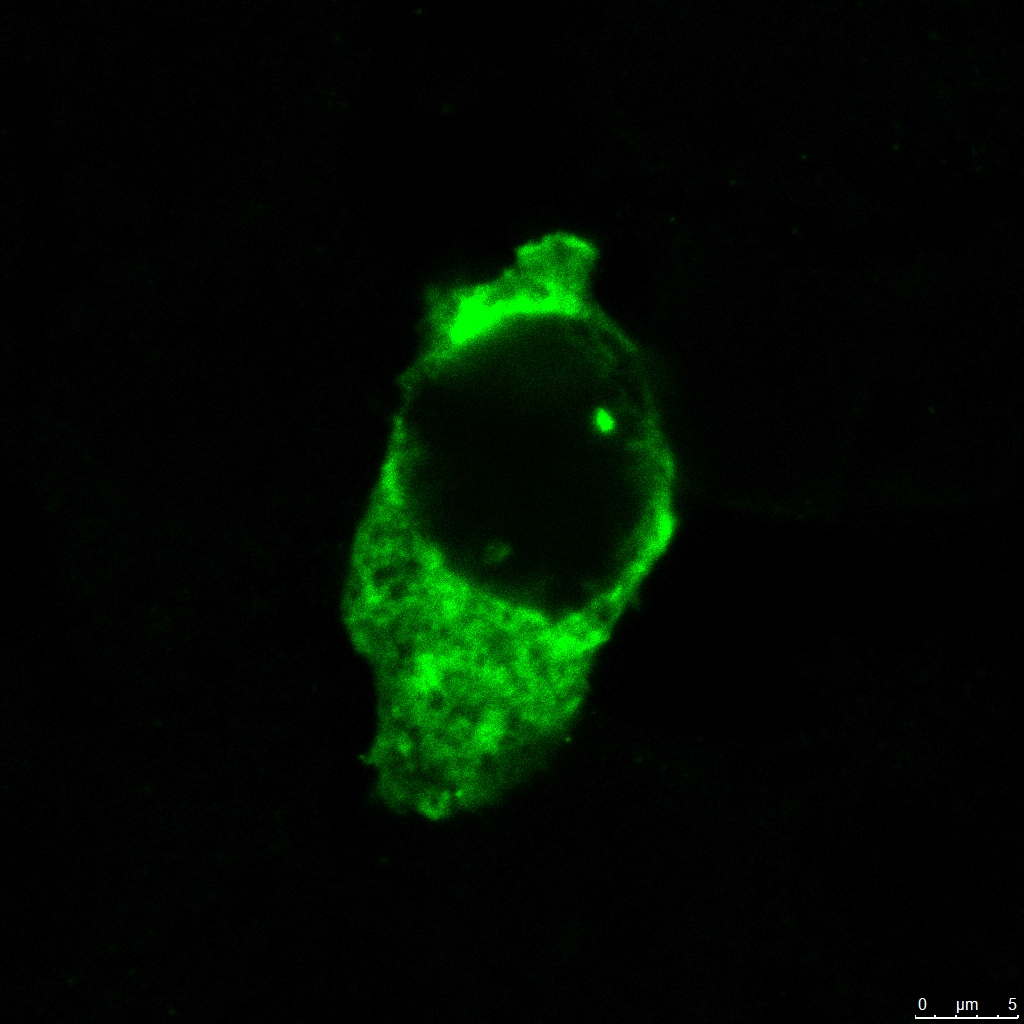

Supplement: Supplementary file 5 — Source data Fig. 2 [file 44319_2024_256_MOESM5_ESM.zip › SourceDateForFigure 2/2I/Experiment.lif_plvx-mda5-1-FIG-2I/Experiment.lif_plvx-mda5-1_z0_ch01.jpg]

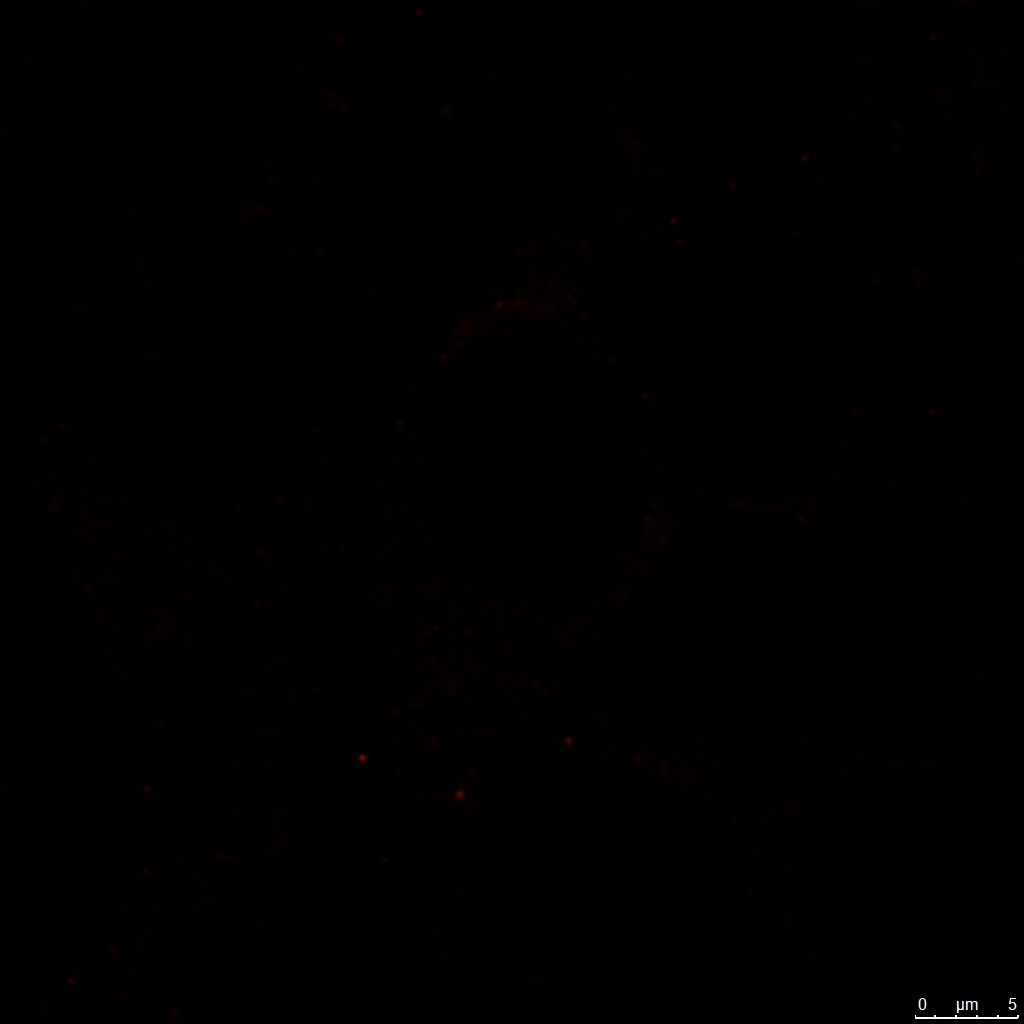

Supplement: Supplementary file 5 — Source data Fig. 2 [file 44319_2024_256_MOESM5_ESM.zip › SourceDateForFigure 2/2I/Experiment.lif_plvx-mda5-1-FIG-2I/Experiment.lif_plvx-mda5-1_z0_ch02.jpg]

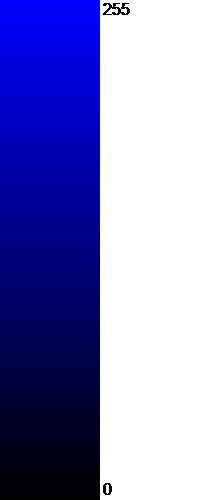

Supplement: Supplementary file 5 — Source data Fig. 2 [file 44319_2024_256_MOESM5_ESM.zip › SourceDateForFigure 2/2I/Experiment.lif_plvx-mda5-1-FIG-2I/MetaData/Experiment.lif_plvx-mda5-1ch0LUT.png]

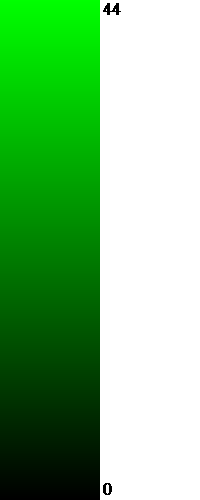

Supplement: Supplementary file 5 — Source data Fig. 2 [file 44319_2024_256_MOESM5_ESM.zip › SourceDateForFigure 2/2I/Experiment.lif_plvx-mda5-1-FIG-2I/MetaData/Experiment.lif_plvx-mda5-1ch1LUT.png]

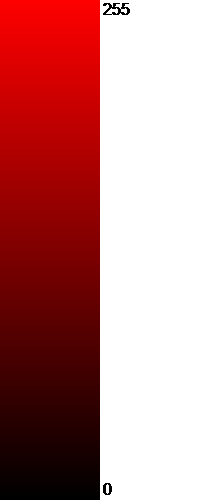

Supplement: Supplementary file 5 — Source data Fig. 2 [file 44319_2024_256_MOESM5_ESM.zip › SourceDateForFigure 2/2I/Experiment.lif_plvx-mda5-1-FIG-2I/MetaData/Experiment.lif_plvx-mda5-1ch2LUT.png]

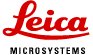

Supplement: Supplementary file 5 — Source data Fig. 2 [file 44319_2024_256_MOESM5_ESM.zip › SourceDateForFigure 2/2I/Experiment.lif_plvx-mda5-1-FIG-2I/MetaData/LeicaLogo.jpg]

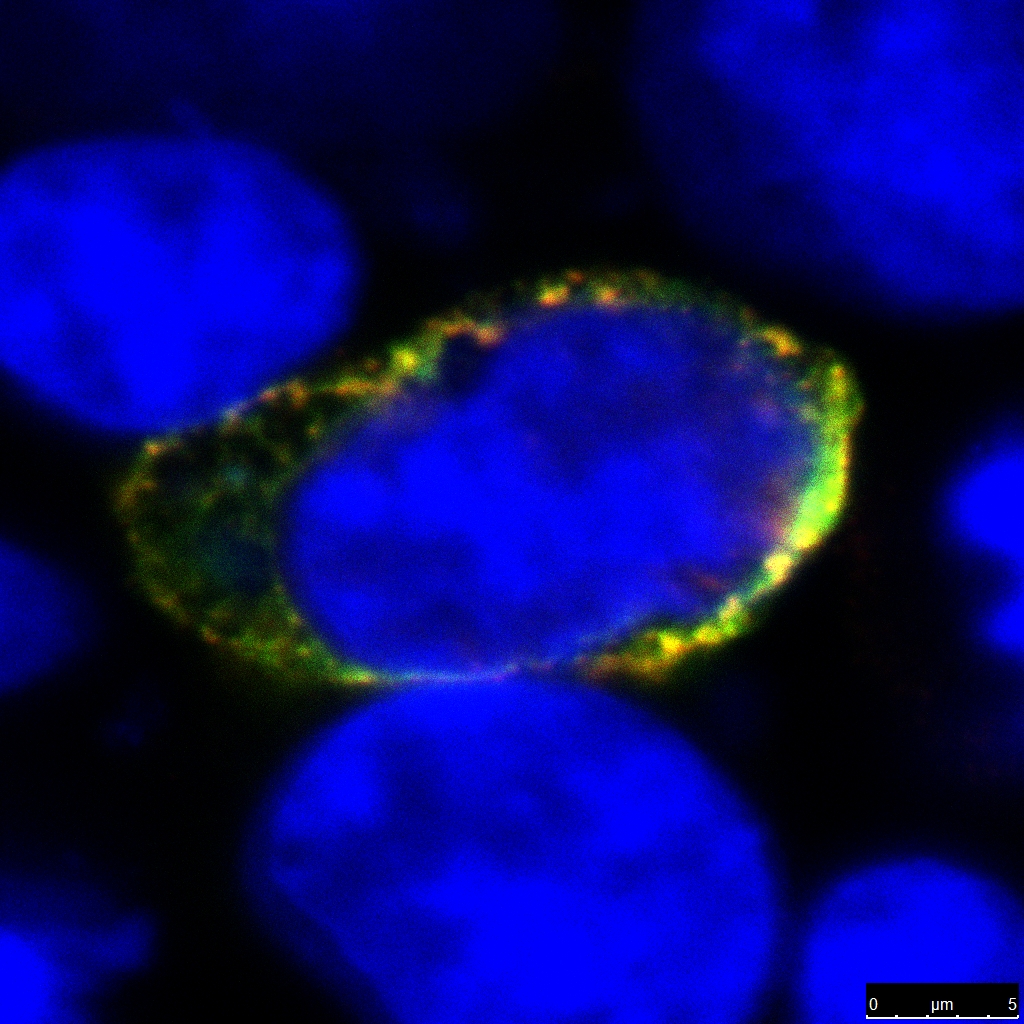

Supplement: Supplementary file 5 — Source data Fig. 2 [file 44319_2024_256_MOESM5_ESM.zip › SourceDateForFigure 2/2I/Experiment.lif_rnf-mda5-2-FIG-2I/Experiment.lif_rnf-mda5-2_z0.jpg]

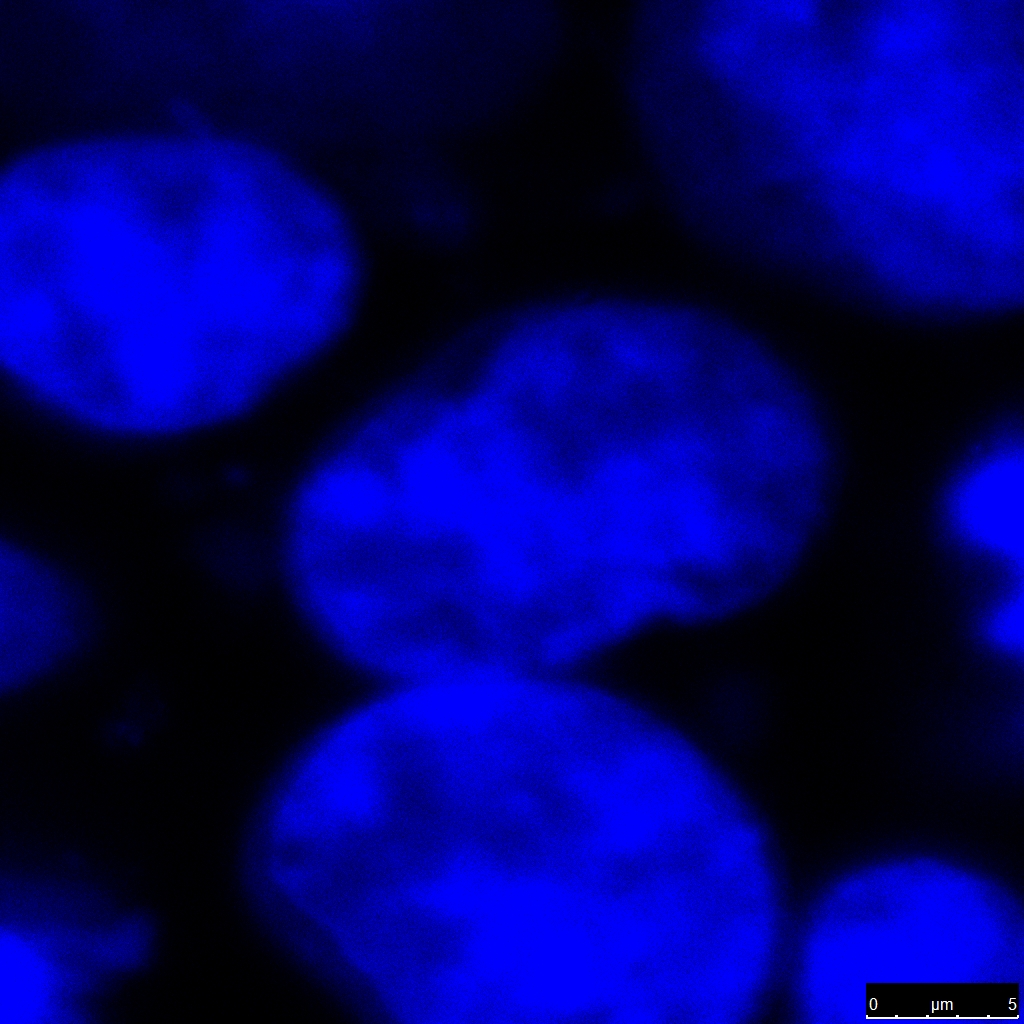

Supplement: Supplementary file 5 — Source data Fig. 2 [file 44319_2024_256_MOESM5_ESM.zip › SourceDateForFigure 2/2I/Experiment.lif_rnf-mda5-2-FIG-2I/Experiment.lif_rnf-mda5-2_z0_ch00.jpg]

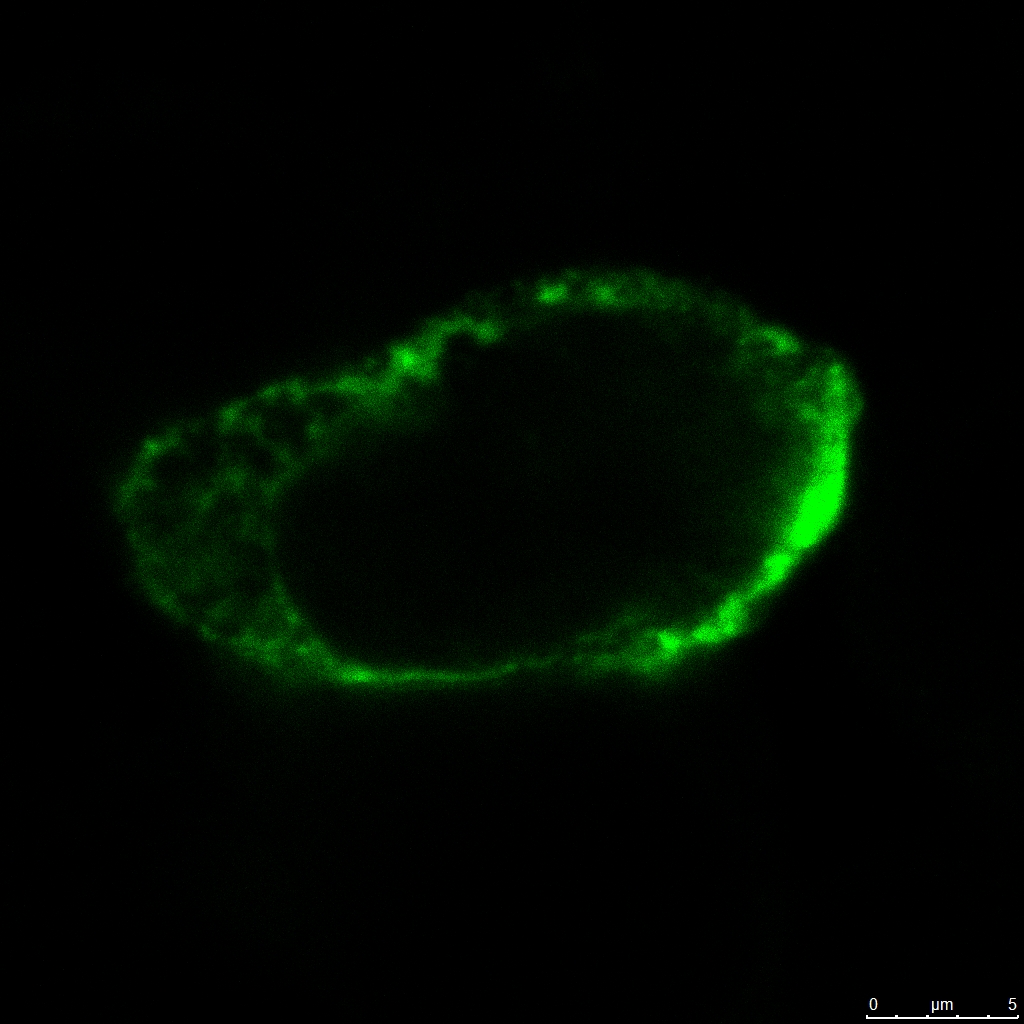

Supplement: Supplementary file 5 — Source data Fig. 2 [file 44319_2024_256_MOESM5_ESM.zip › SourceDateForFigure 2/2I/Experiment.lif_rnf-mda5-2-FIG-2I/Experiment.lif_rnf-mda5-2_z0_ch01.jpg]

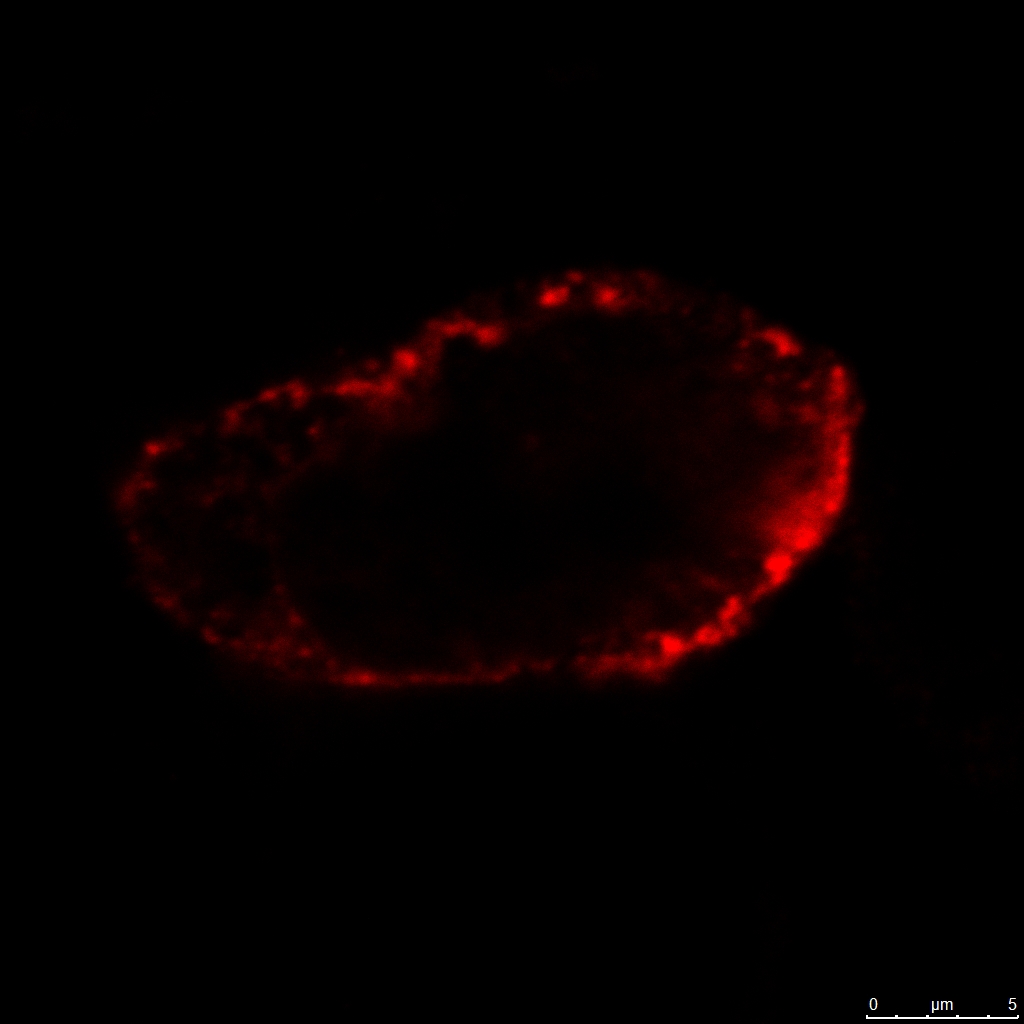

Supplement: Supplementary file 5 — Source data Fig. 2 [file 44319_2024_256_MOESM5_ESM.zip › SourceDateForFigure 2/2I/Experiment.lif_rnf-mda5-2-FIG-2I/Experiment.lif_rnf-mda5-2_z0_ch02.jpg]

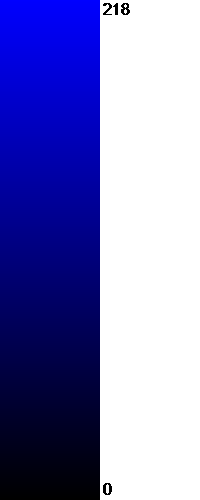

Supplement: Supplementary file 5 — Source data Fig. 2 [file 44319_2024_256_MOESM5_ESM.zip › SourceDateForFigure 2/2I/Experiment.lif_rnf-mda5-2-FIG-2I/MetaData/Experiment.lif_rnf-mda5-2ch0LUT.png]

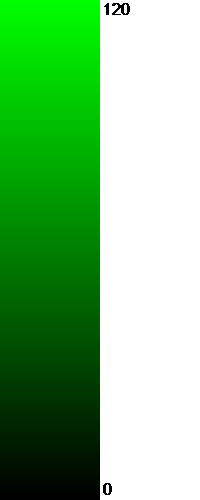

Supplement: Supplementary file 5 — Source data Fig. 2 [file 44319_2024_256_MOESM5_ESM.zip › SourceDateForFigure 2/2I/Experiment.lif_rnf-mda5-2-FIG-2I/MetaData/Experiment.lif_rnf-mda5-2ch1LUT.png]

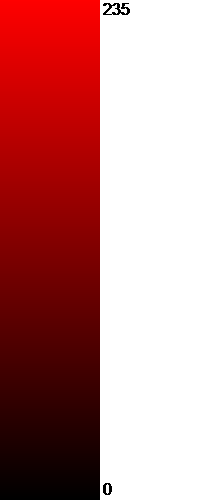

Supplement: Supplementary file 5 — Source data Fig. 2 [file 44319_2024_256_MOESM5_ESM.zip › SourceDateForFigure 2/2I/Experiment.lif_rnf-mda5-2-FIG-2I/MetaData/Experiment.lif_rnf-mda5-2ch2LUT.png]

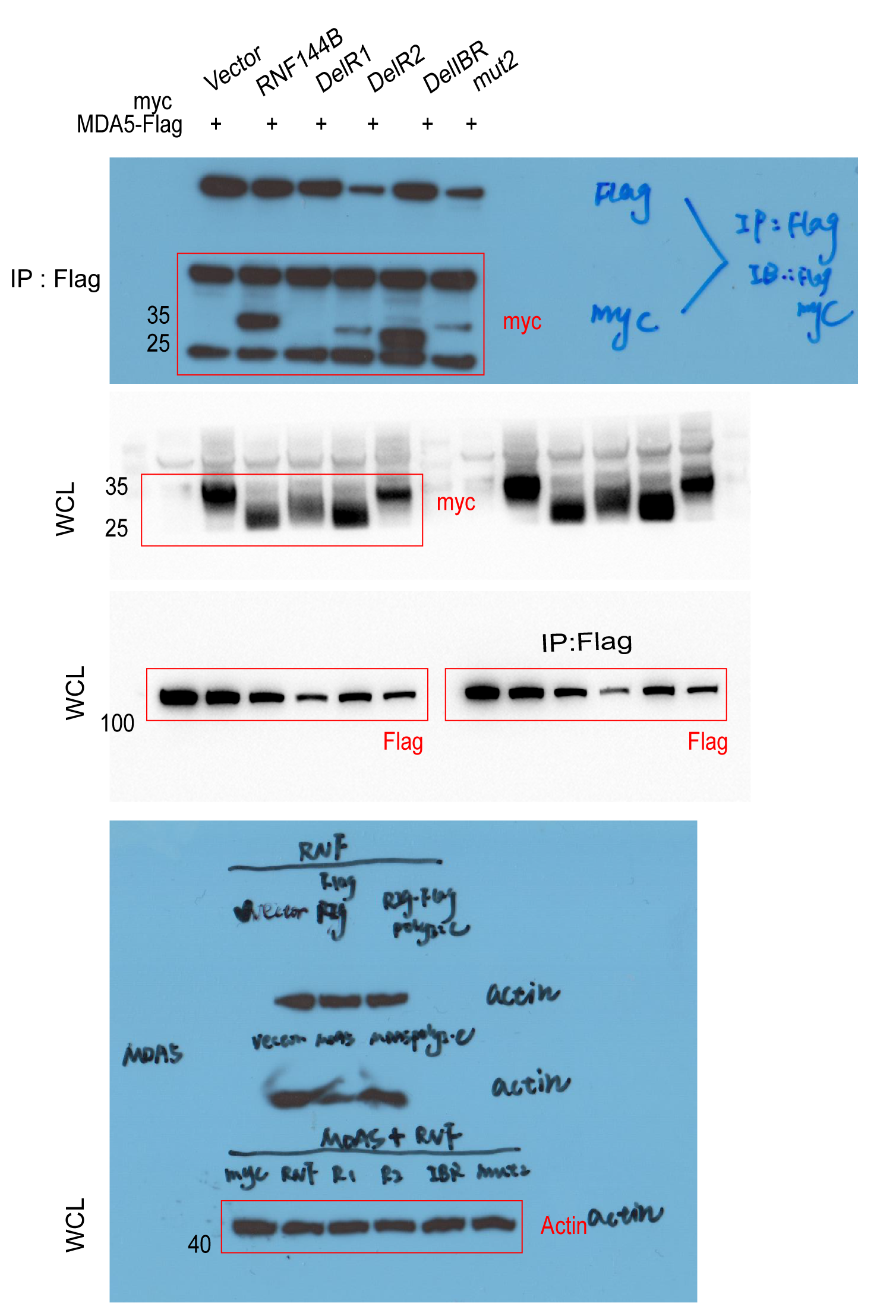

Supplement: Supplementary file 5 — Source data Fig. 2 [file 44319_2024_256_MOESM5_ESM.zip › SourceDateForFigure 2/2J.tif]

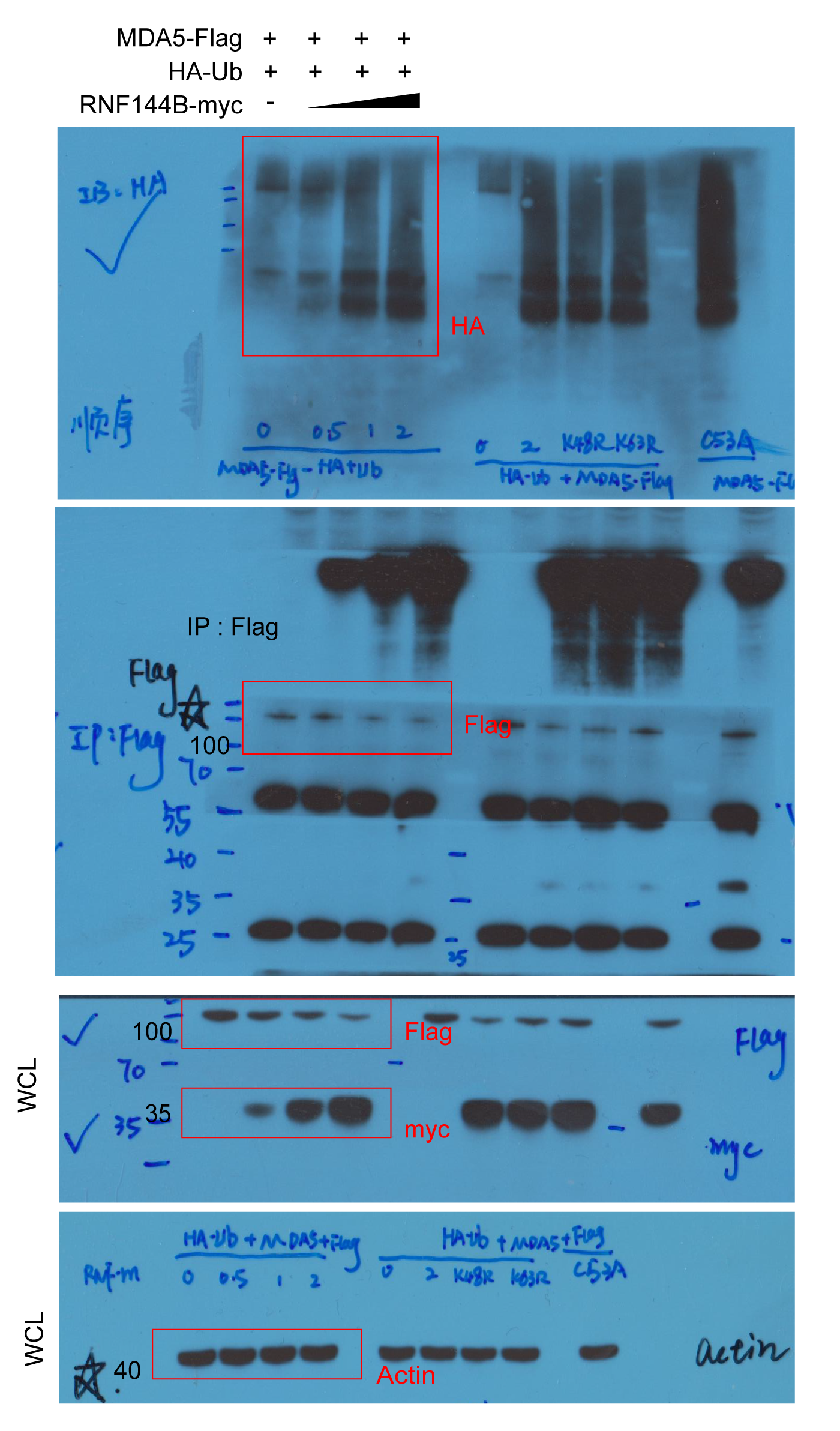

Supplement: Supplementary file 6 — Source data Fig. 3 [file 44319_2024_256_MOESM6_ESM.zip › SourceDateForFigure 3/3A.tif]

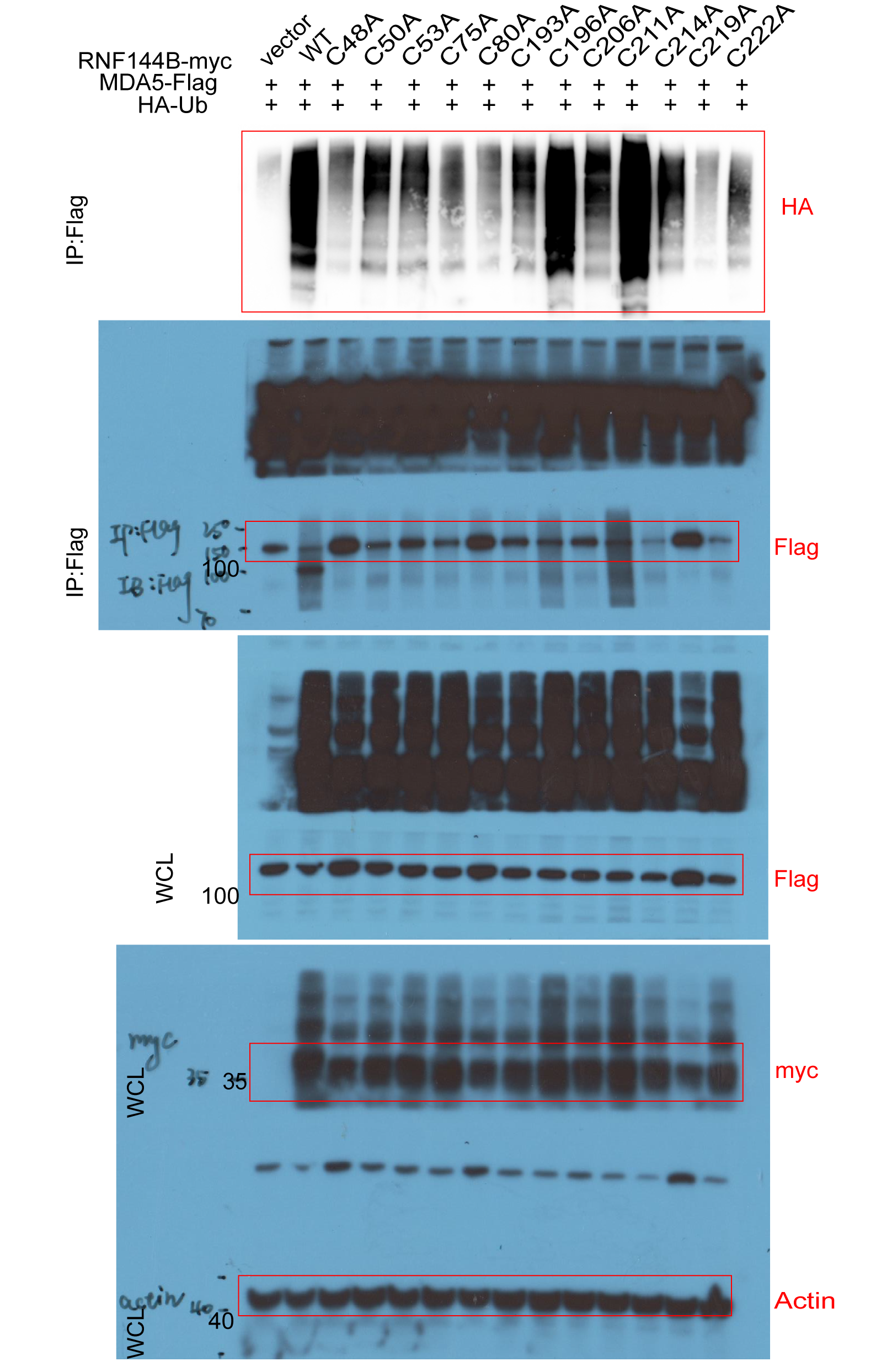

Supplement: Supplementary file 6 — Source data Fig. 3 [file 44319_2024_256_MOESM6_ESM.zip › SourceDateForFigure 3/3B.tif]

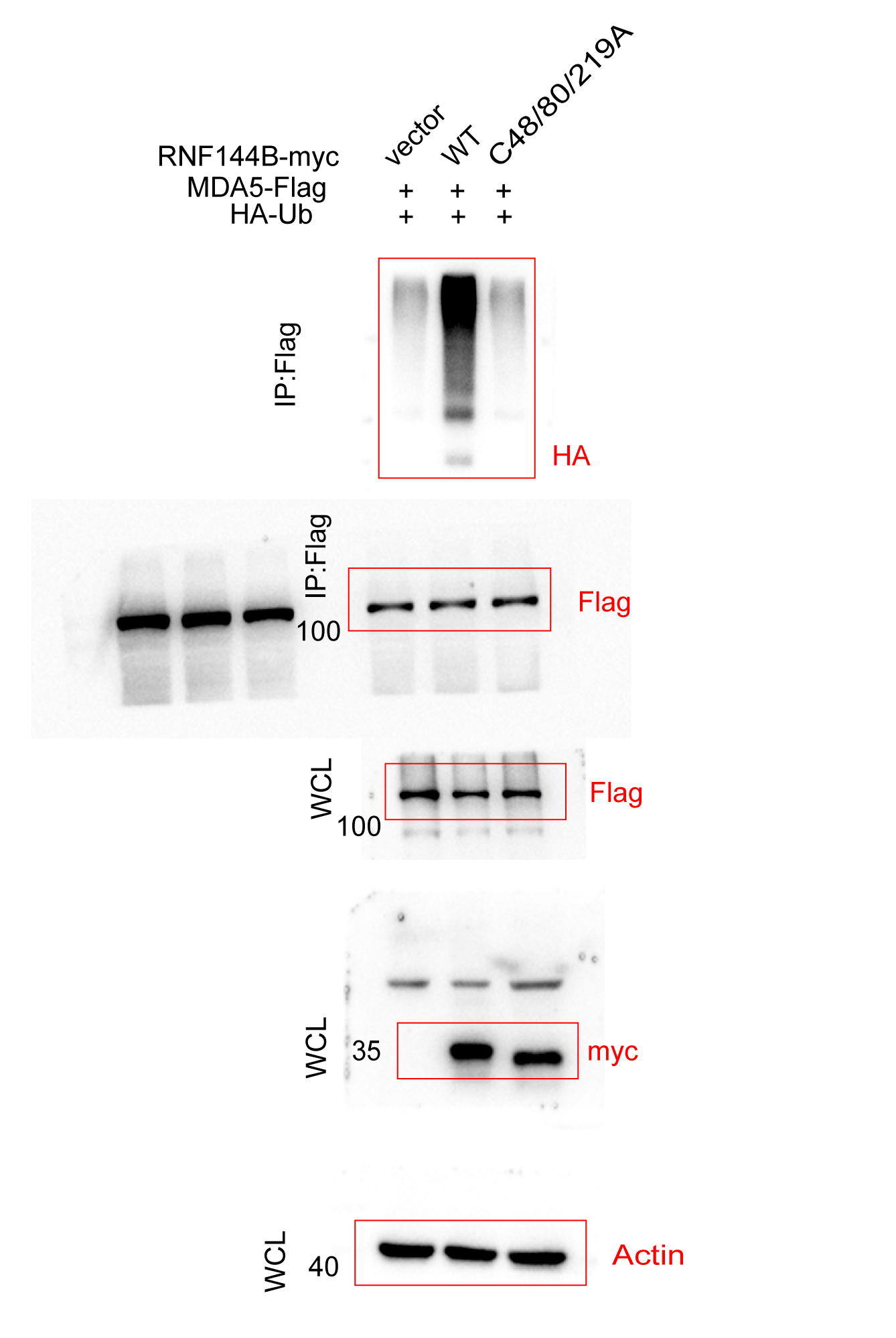

Supplement: Supplementary file 6 — Source data Fig. 3 [file 44319_2024_256_MOESM6_ESM.zip › SourceDateForFigure 3/3C.tif]

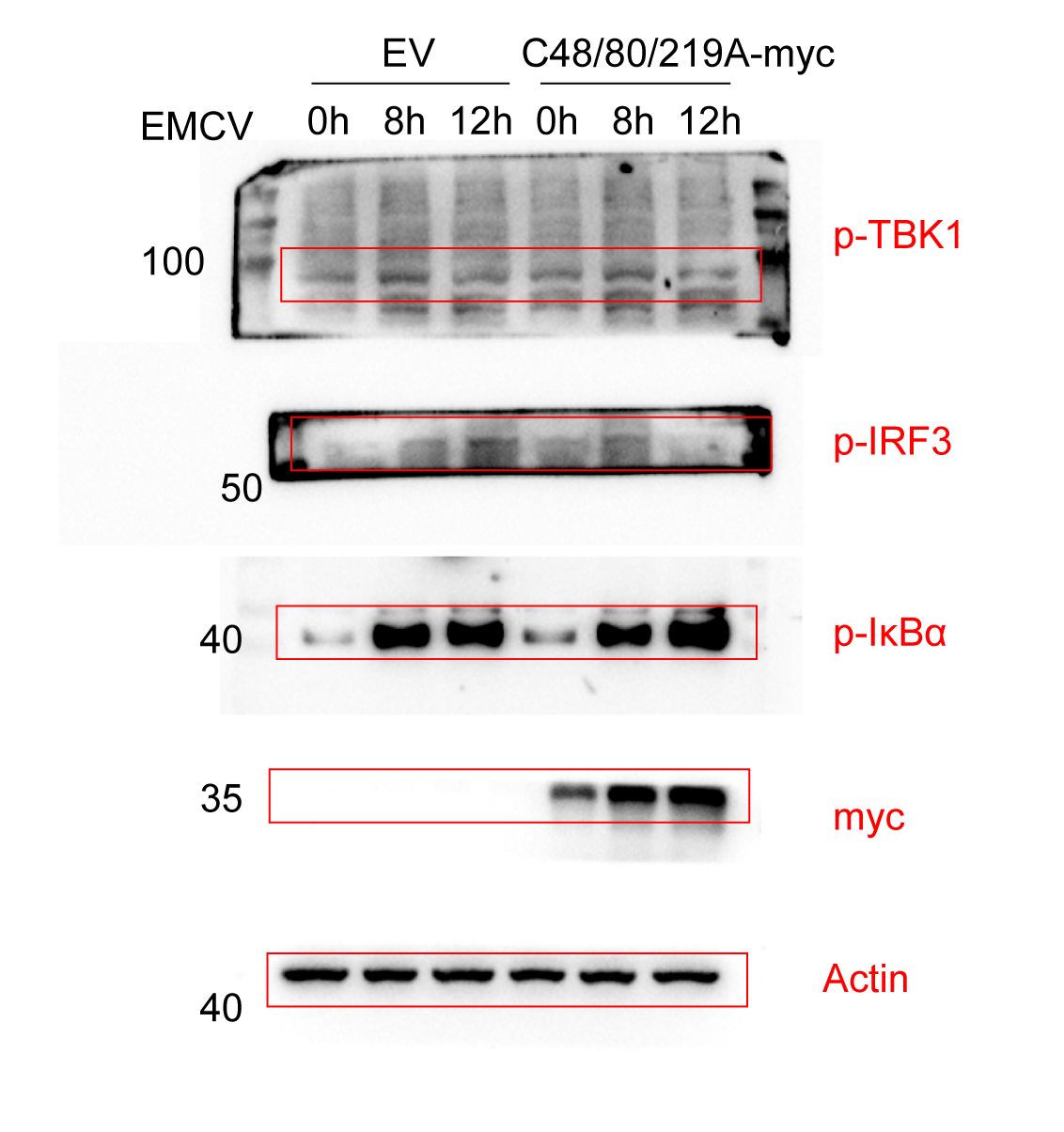

Supplement: Supplementary file 6 — Source data Fig. 3 [file 44319_2024_256_MOESM6_ESM.zip › SourceDateForFigure 3/3E.tif]

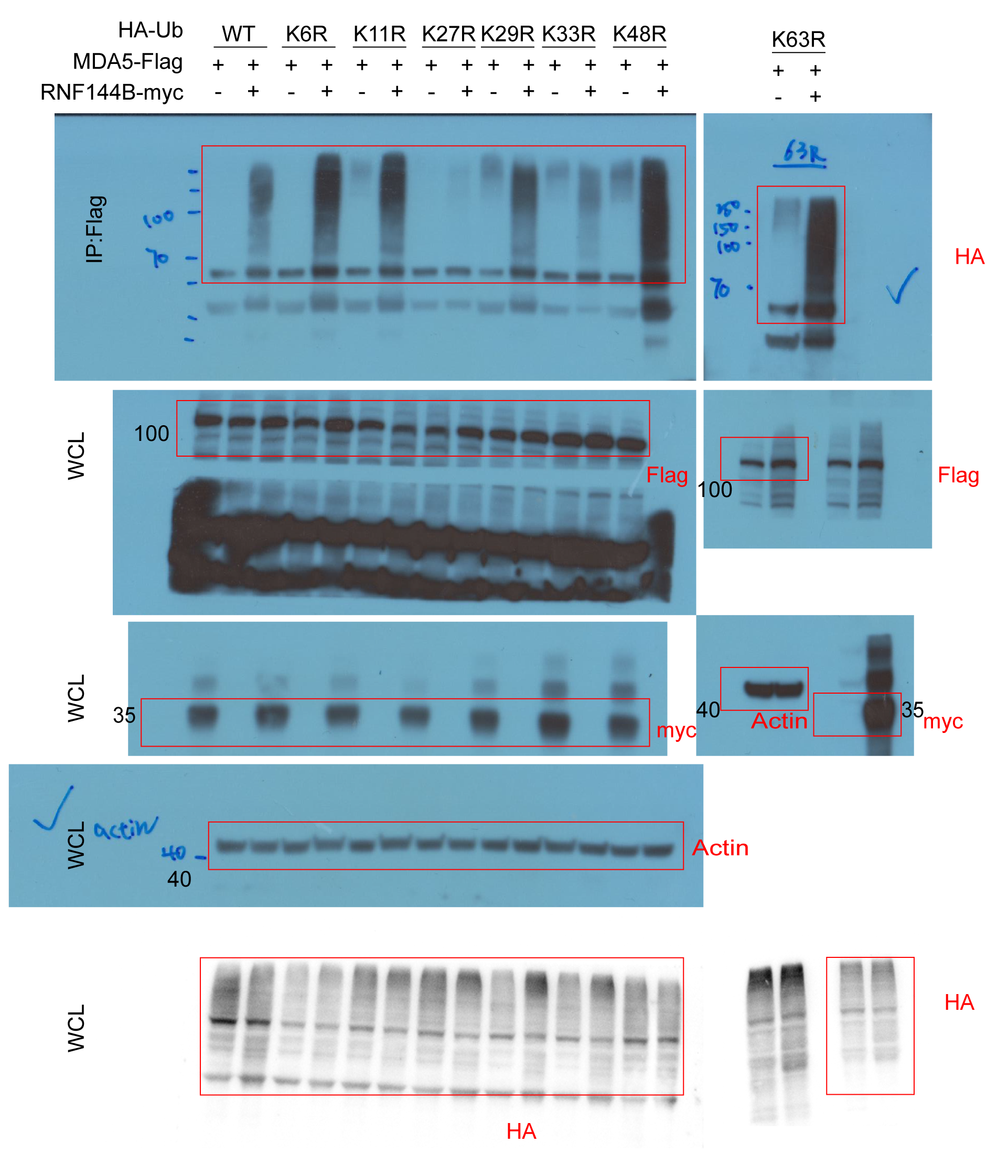

Supplement: Supplementary file 6 — Source data Fig. 3 [file 44319_2024_256_MOESM6_ESM.zip › SourceDateForFigure 3/3F.tif]

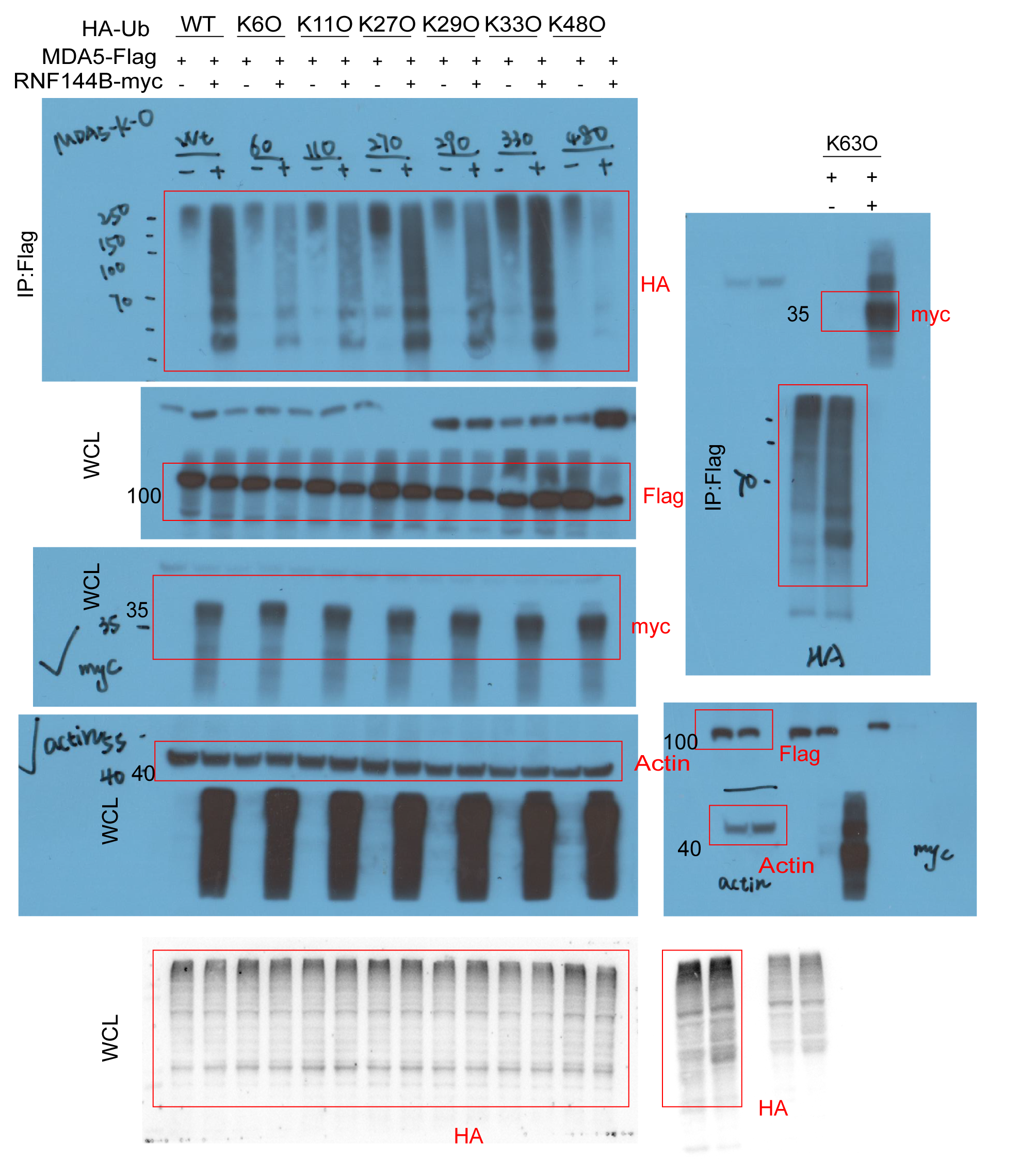

Supplement: Supplementary file 6 — Source data Fig. 3 [file 44319_2024_256_MOESM6_ESM.zip › SourceDateForFigure 3/3G.tif]

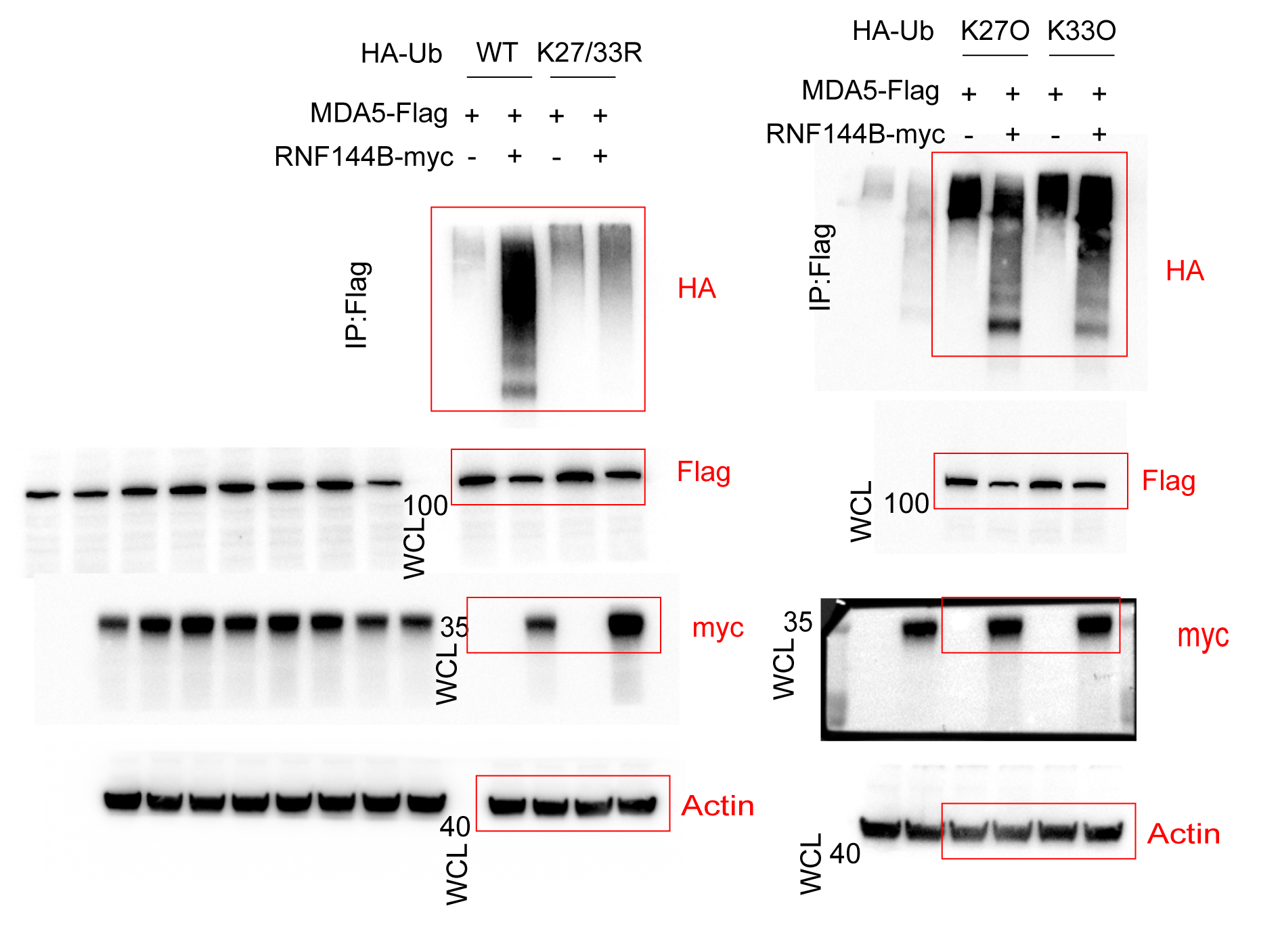

Supplement: Supplementary file 6 — Source data Fig. 3 [file 44319_2024_256_MOESM6_ESM.zip › SourceDateForFigure 3/3H.tif]

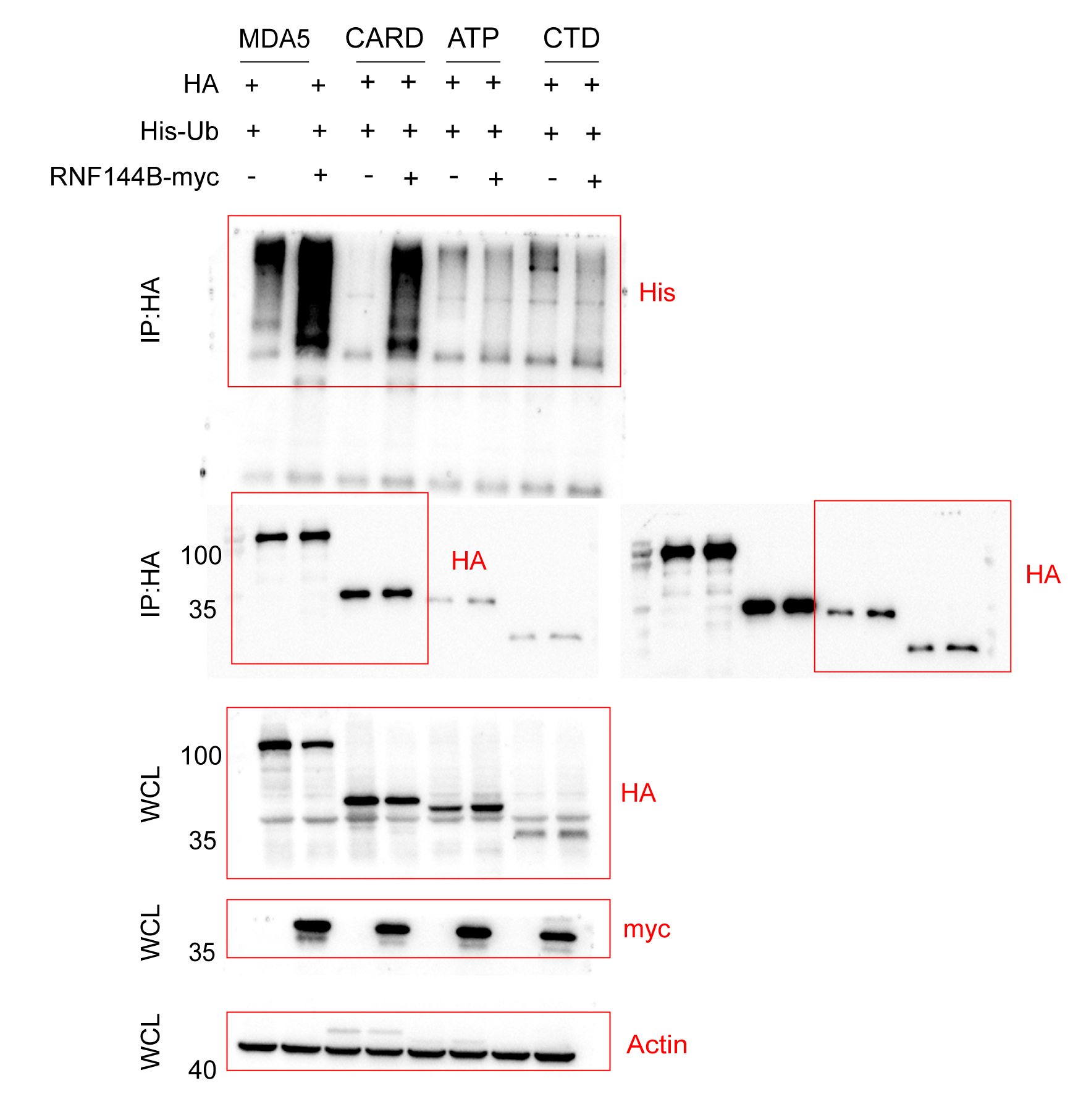

Supplement: Supplementary file 7 — Source data Fig. 4 [file 44319_2024_256_MOESM7_ESM.zip › SourceDateForFigure 4/4A-4D.tif]

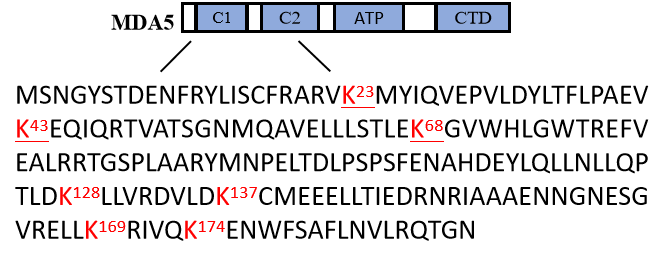

Supplement: Supplementary file 7 — Source data Fig. 4 [file 44319_2024_256_MOESM7_ESM.zip › SourceDateForFigure 4/4E.png]

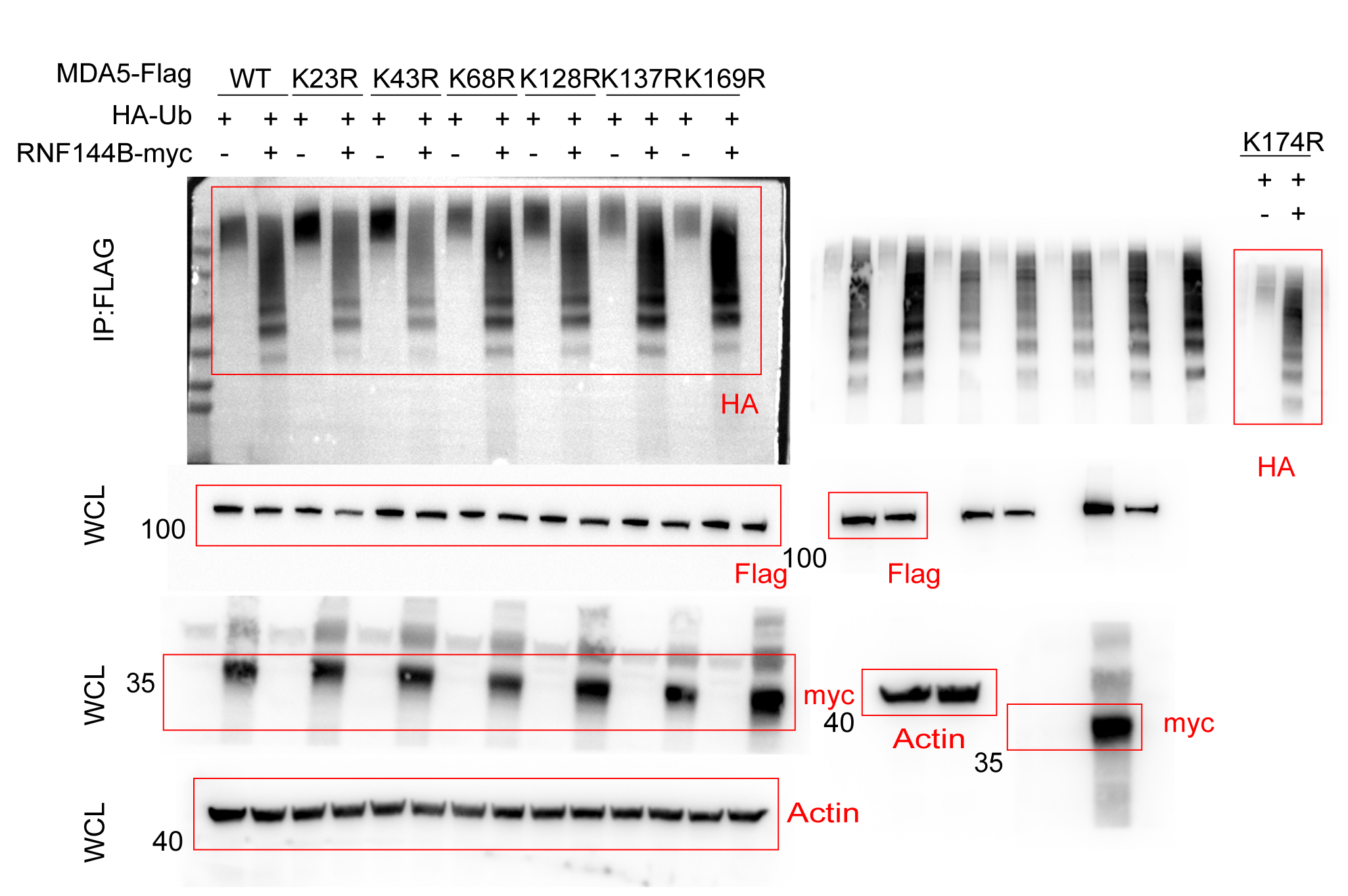

Supplement: Supplementary file 7 — Source data Fig. 4 [file 44319_2024_256_MOESM7_ESM.zip › SourceDateForFigure 4/4F.tif]

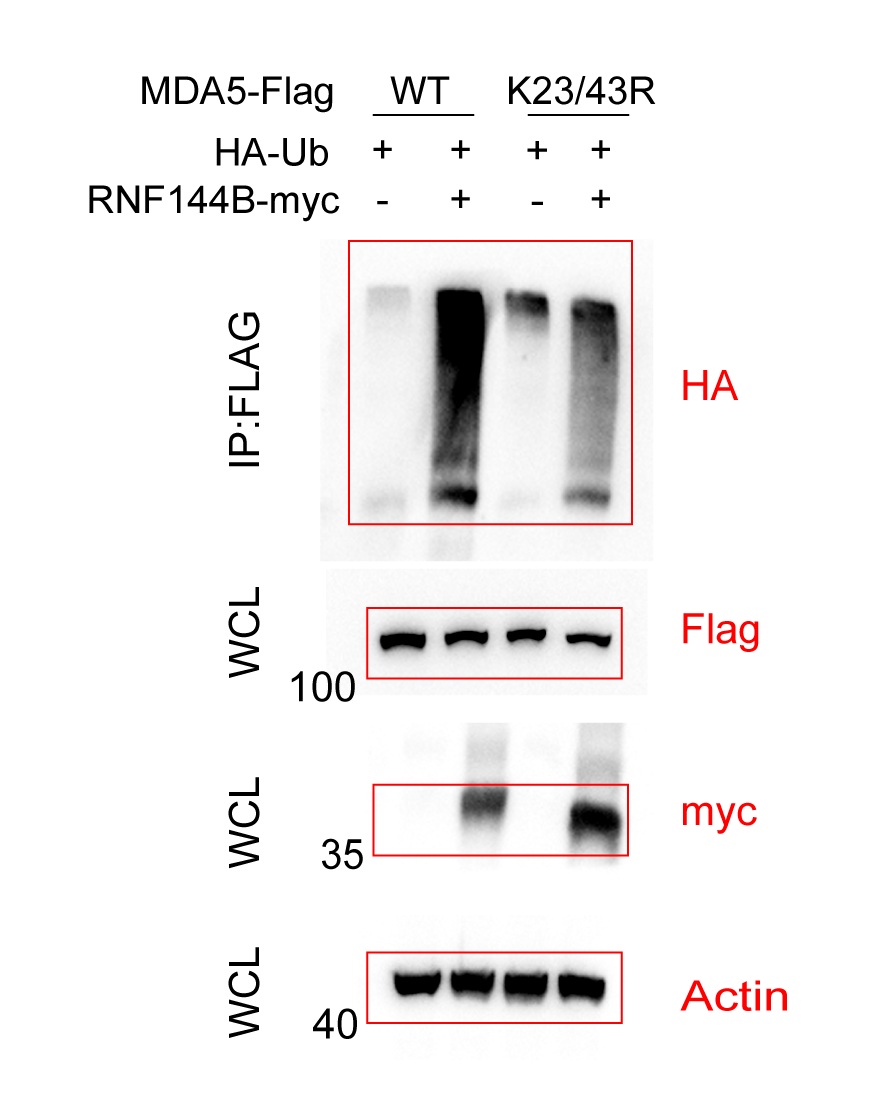

Supplement: Supplementary file 7 — Source data Fig. 4 [file 44319_2024_256_MOESM7_ESM.zip › SourceDateForFigure 4/4G.tif]

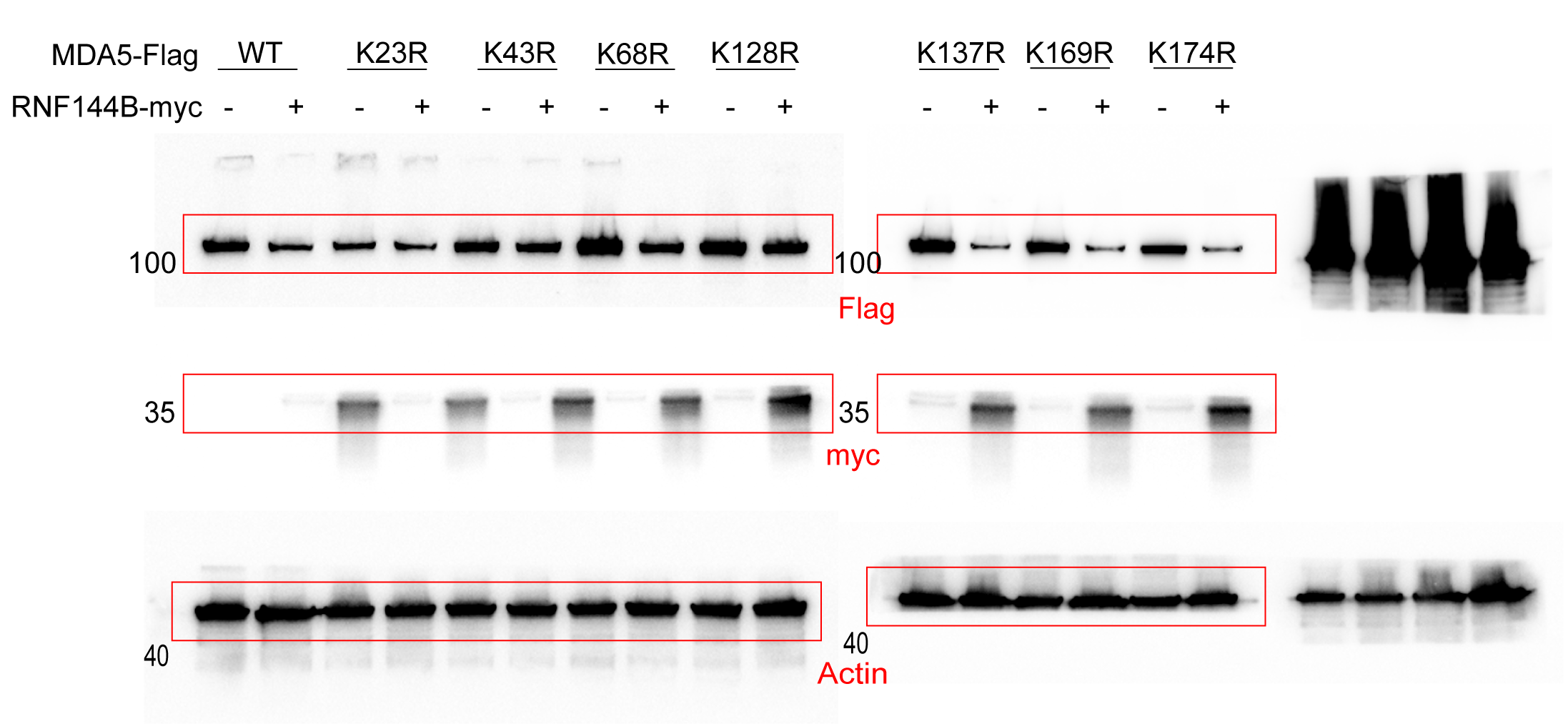

Supplement: Supplementary file 7 — Source data Fig. 4 [file 44319_2024_256_MOESM7_ESM.zip › SourceDateForFigure 4/4H.tif]

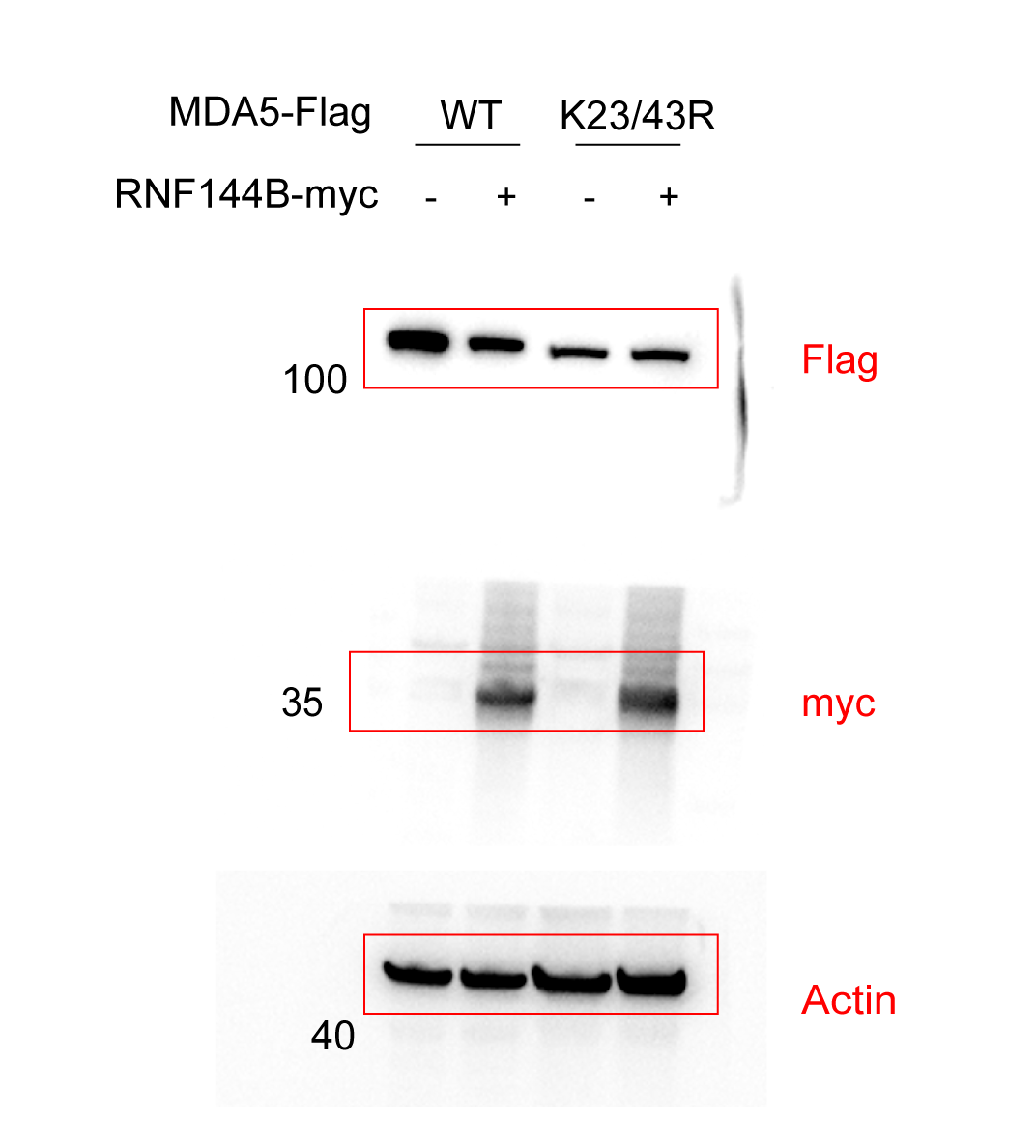

Supplement: Supplementary file 7 — Source data Fig. 4 [file 44319_2024_256_MOESM7_ESM.zip › SourceDateForFigure 4/4I.tif]

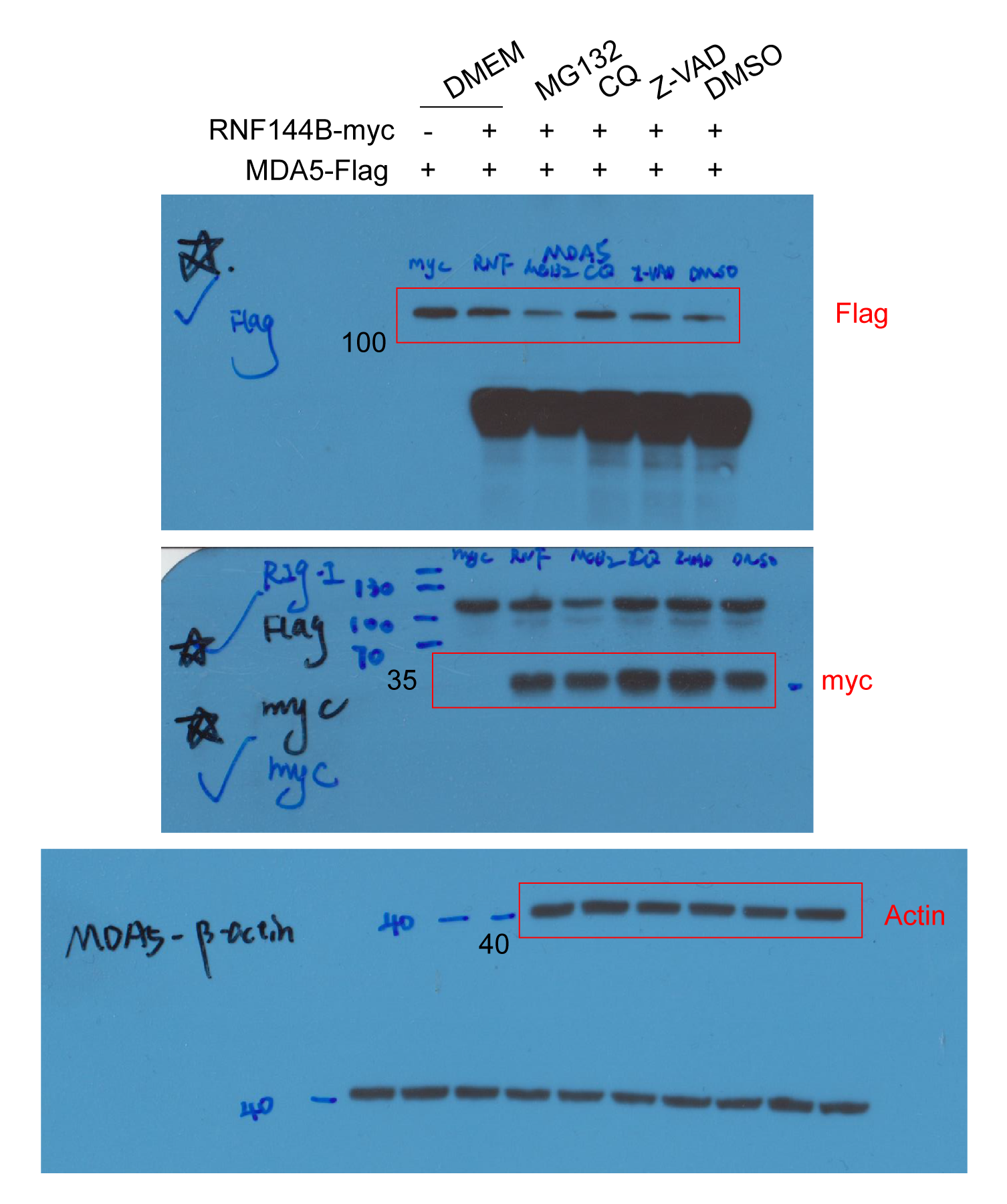

Supplement: Supplementary file 8 — Source data Fig. 5 [file 44319_2024_256_MOESM8_ESM.zip › SourceDateForFigure 5/5A.tif]

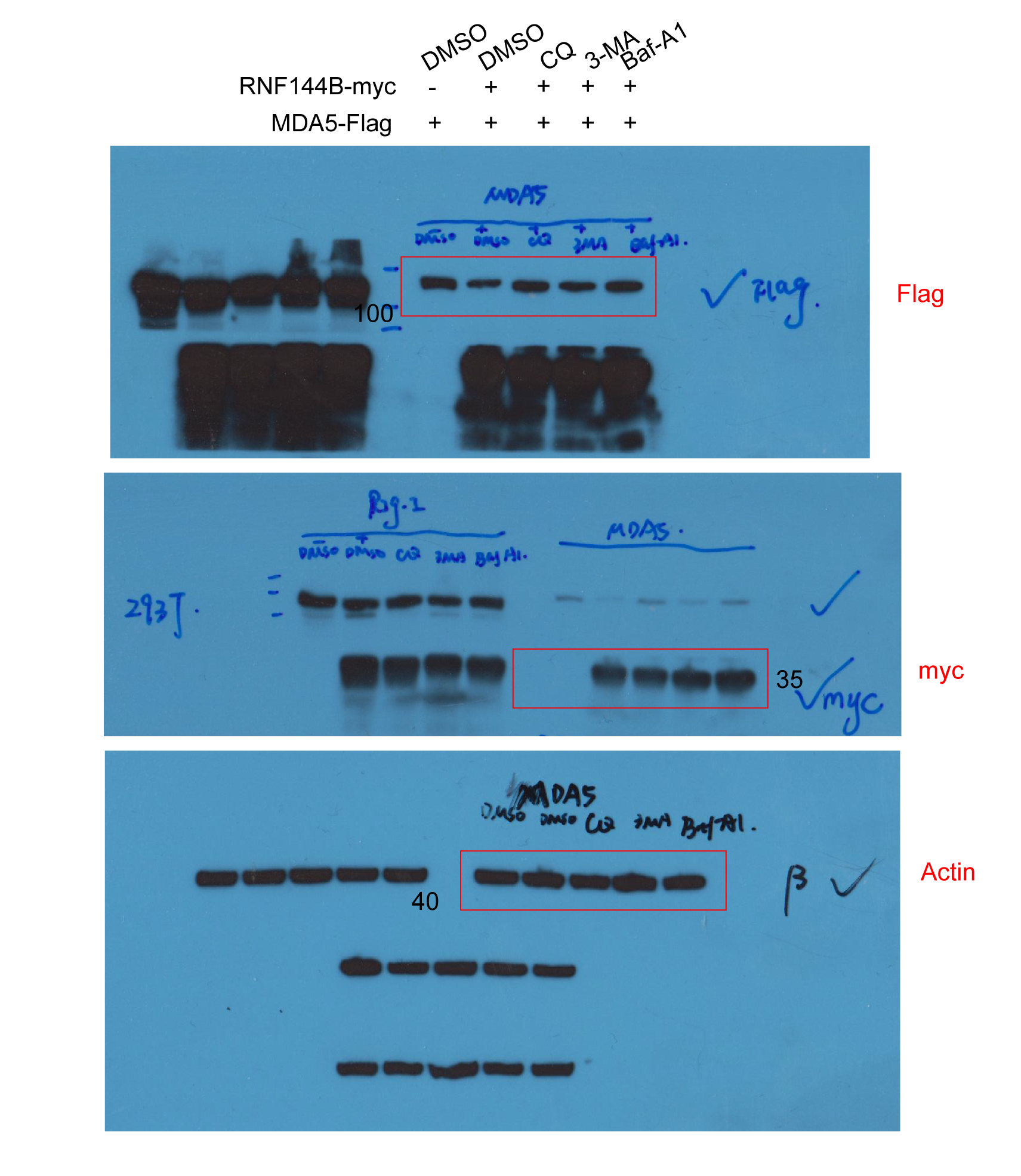

Supplement: Supplementary file 8 — Source data Fig. 5 [file 44319_2024_256_MOESM8_ESM.zip › SourceDateForFigure 5/5B.tif]

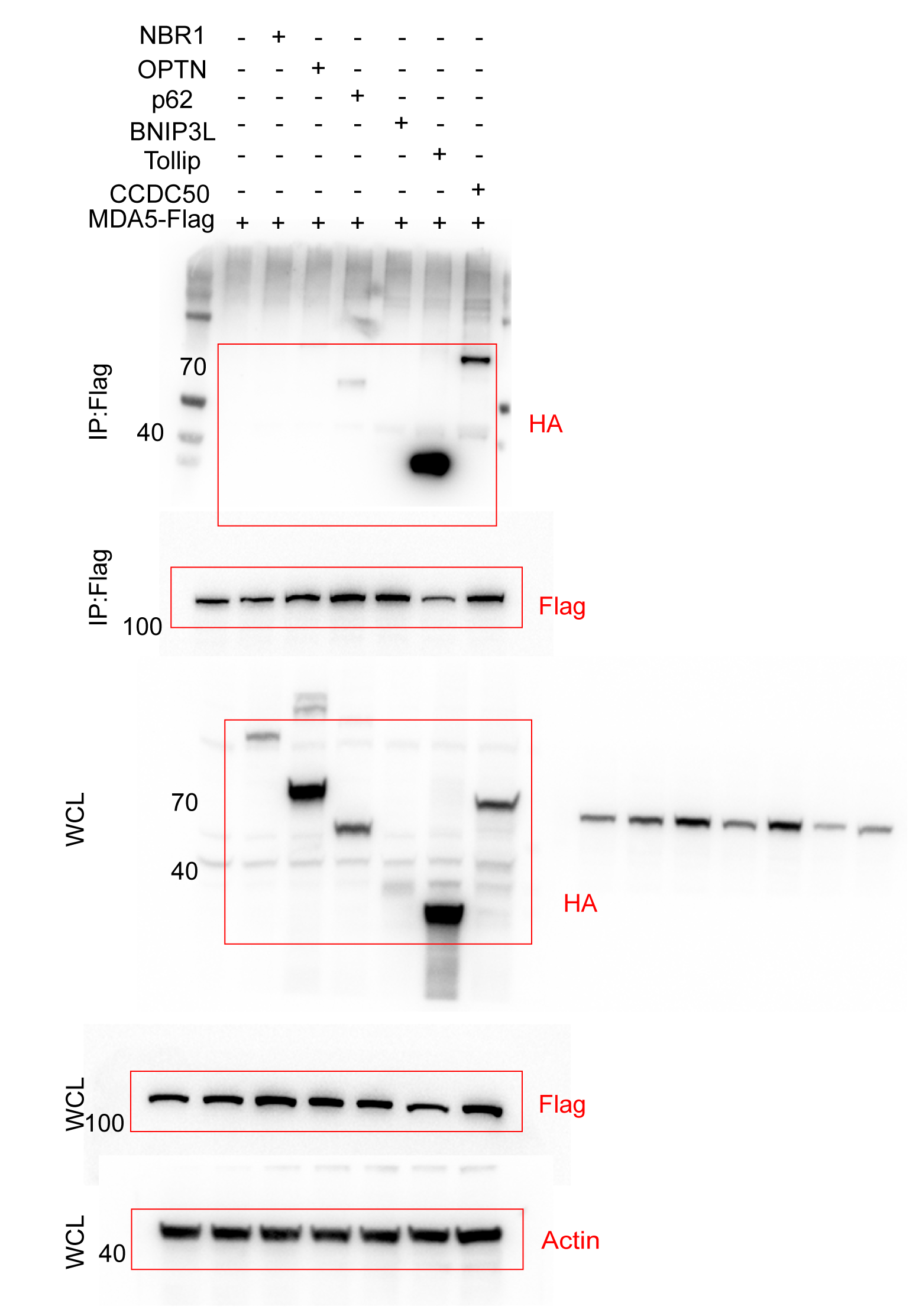

Supplement: Supplementary file 8 — Source data Fig. 5 [file 44319_2024_256_MOESM8_ESM.zip › SourceDateForFigure 5/5C.tif]

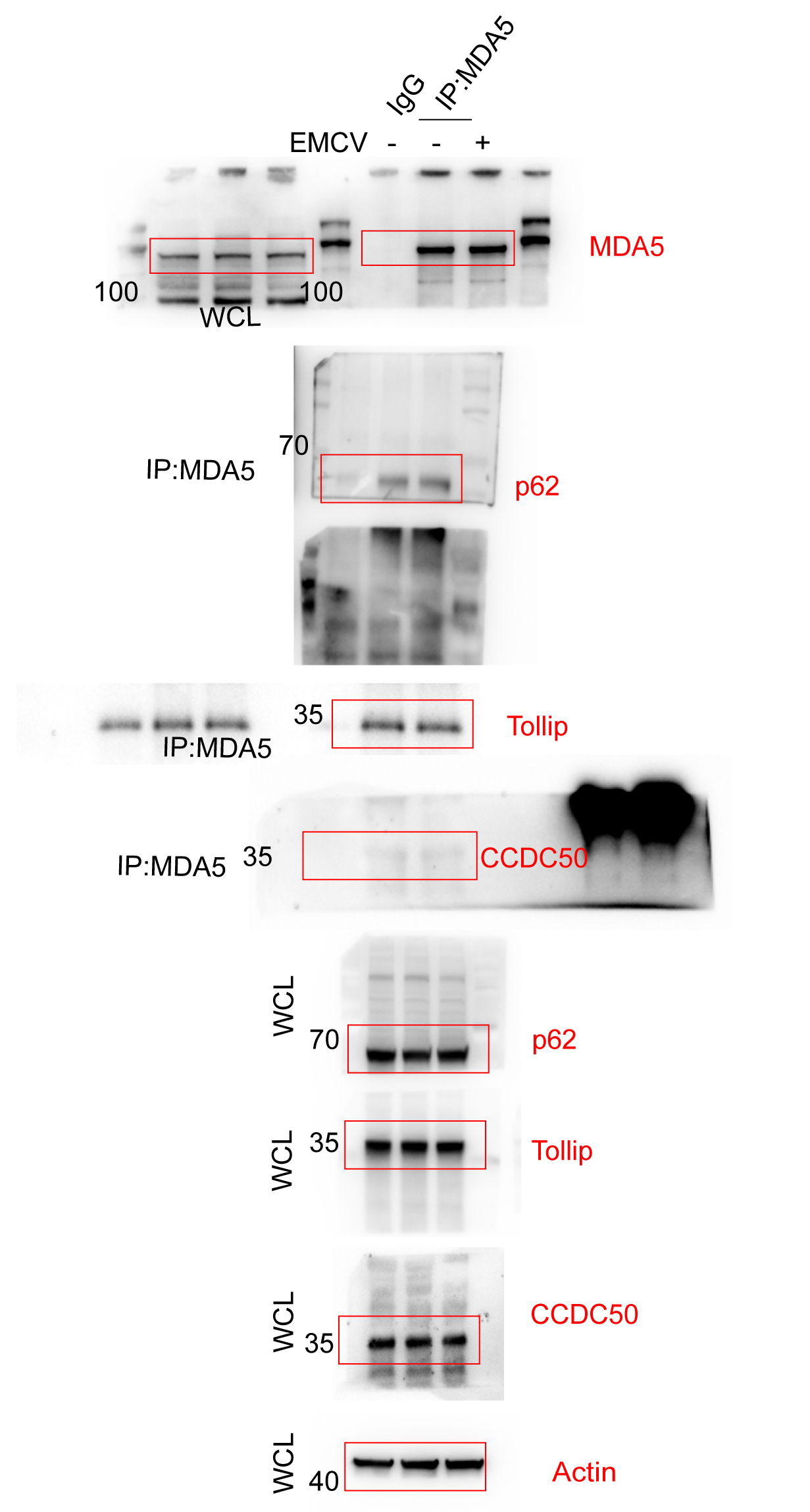

Supplement: Supplementary file 8 — Source data Fig. 5 [file 44319_2024_256_MOESM8_ESM.zip › SourceDateForFigure 5/5D.tif]

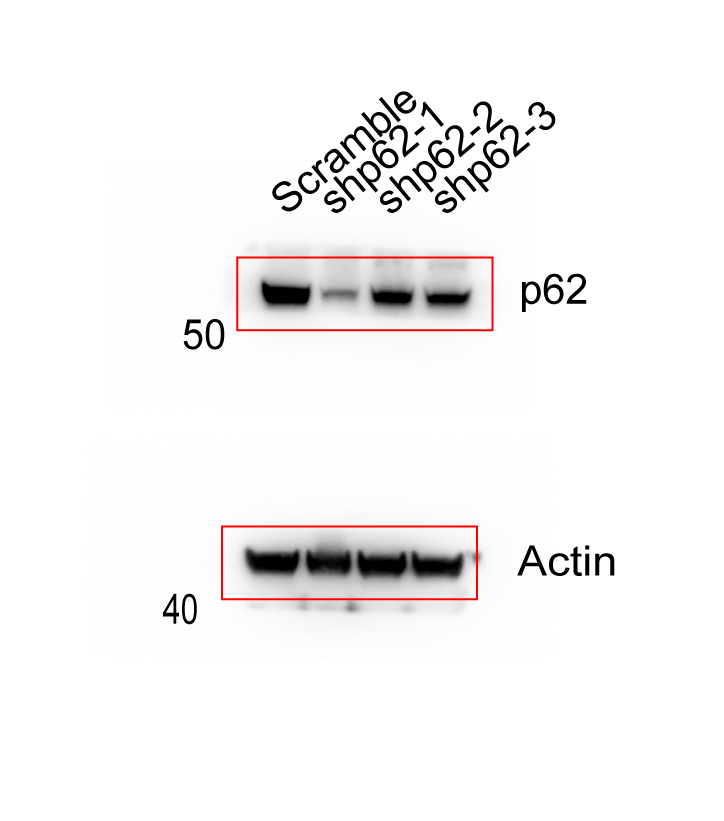

Supplement: Supplementary file 8 — Source data Fig. 5 [file 44319_2024_256_MOESM8_ESM.zip › SourceDateForFigure 5/5E.tif]

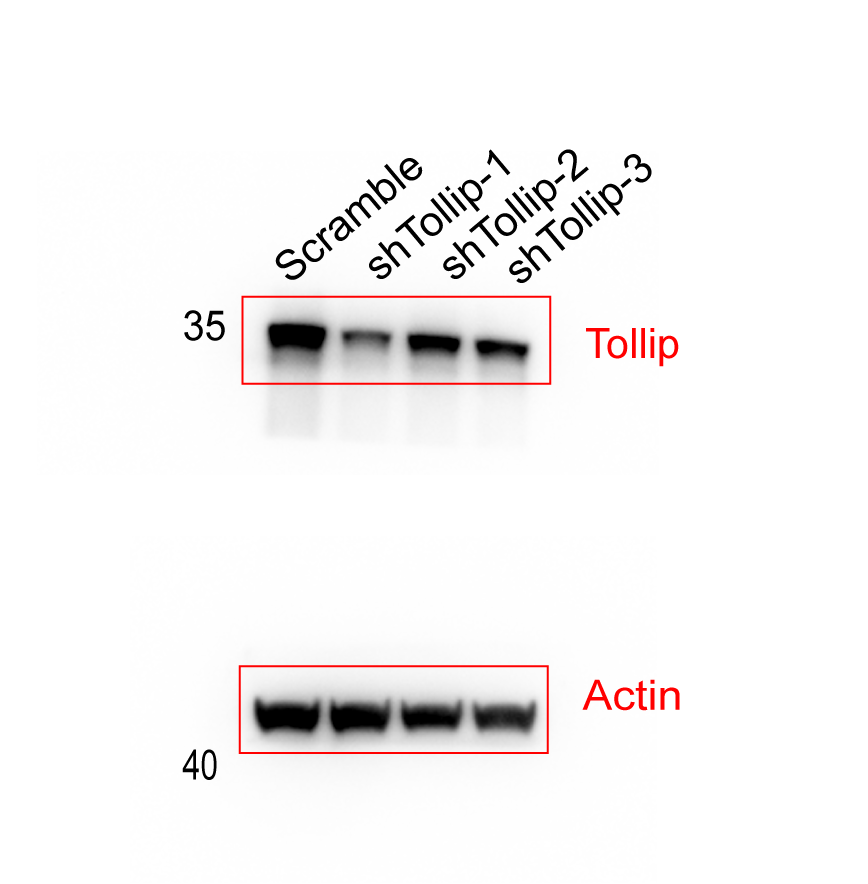

Supplement: Supplementary file 8 — Source data Fig. 5 [file 44319_2024_256_MOESM8_ESM.zip › SourceDateForFigure 5/5F.tif]

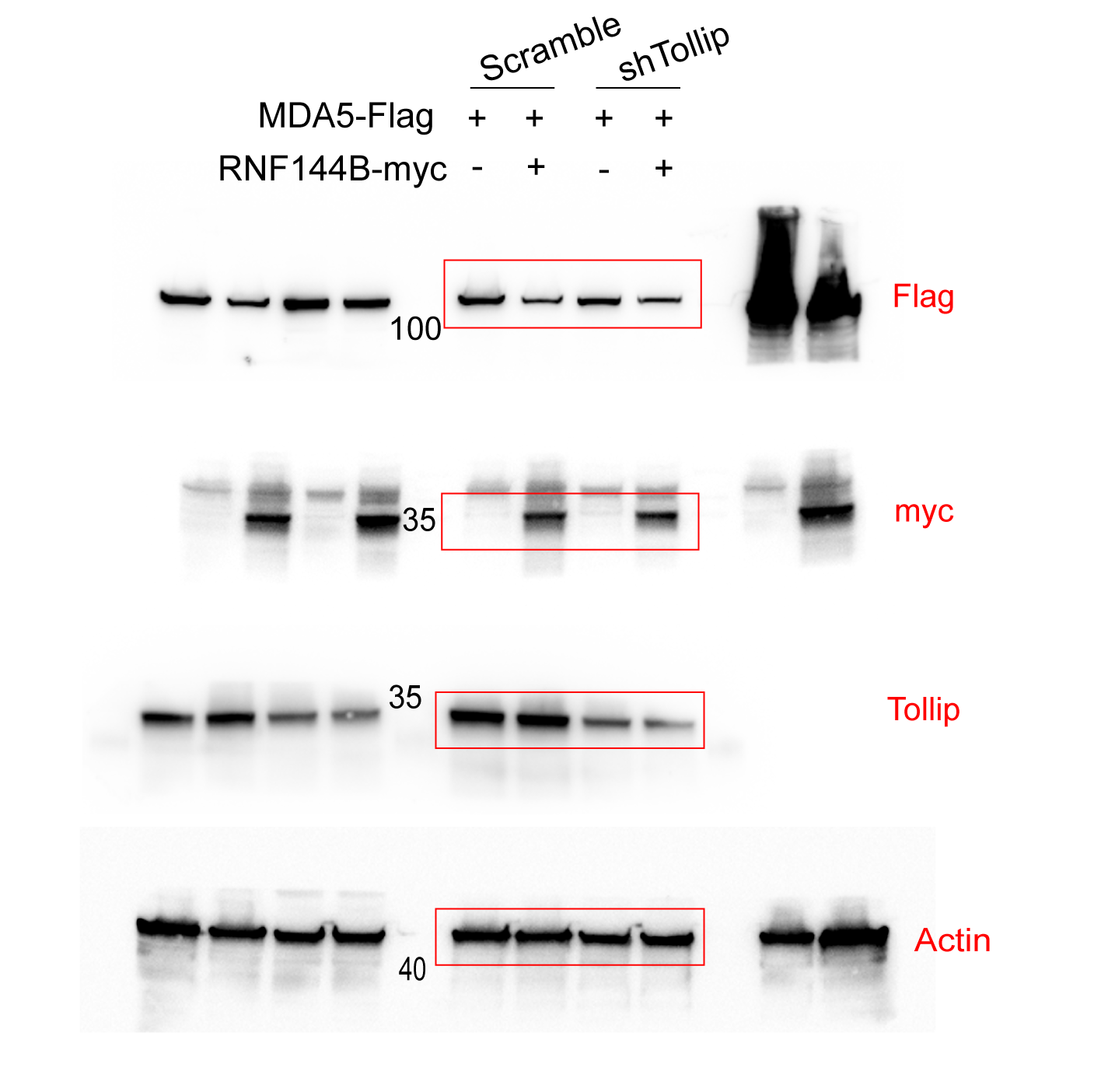

Supplement: Supplementary file 8 — Source data Fig. 5 [file 44319_2024_256_MOESM8_ESM.zip › SourceDateForFigure 5/5G.tif]

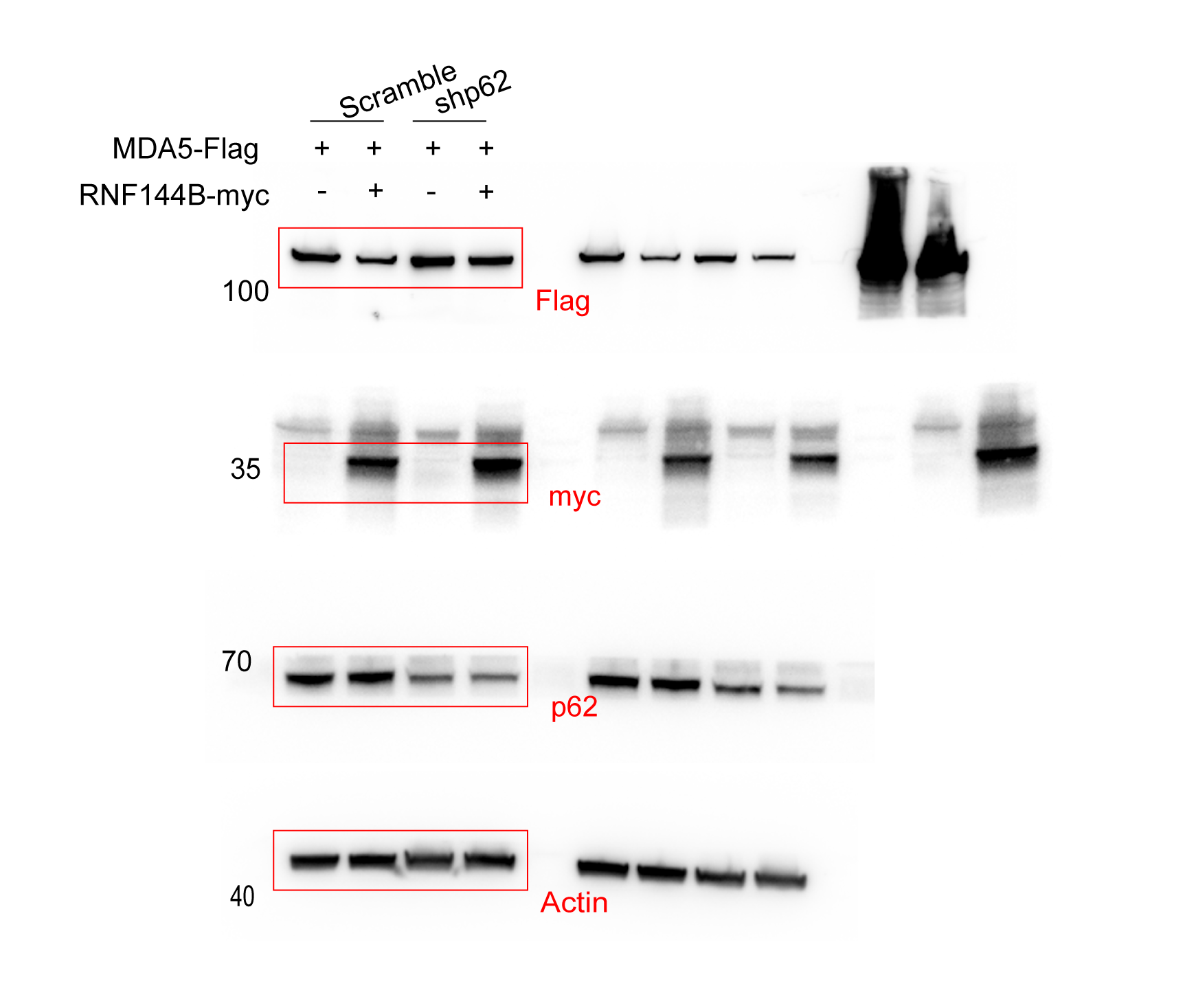

Supplement: Supplementary file 8 — Source data Fig. 5 [file 44319_2024_256_MOESM8_ESM.zip › SourceDateForFigure 5/5H.tif]

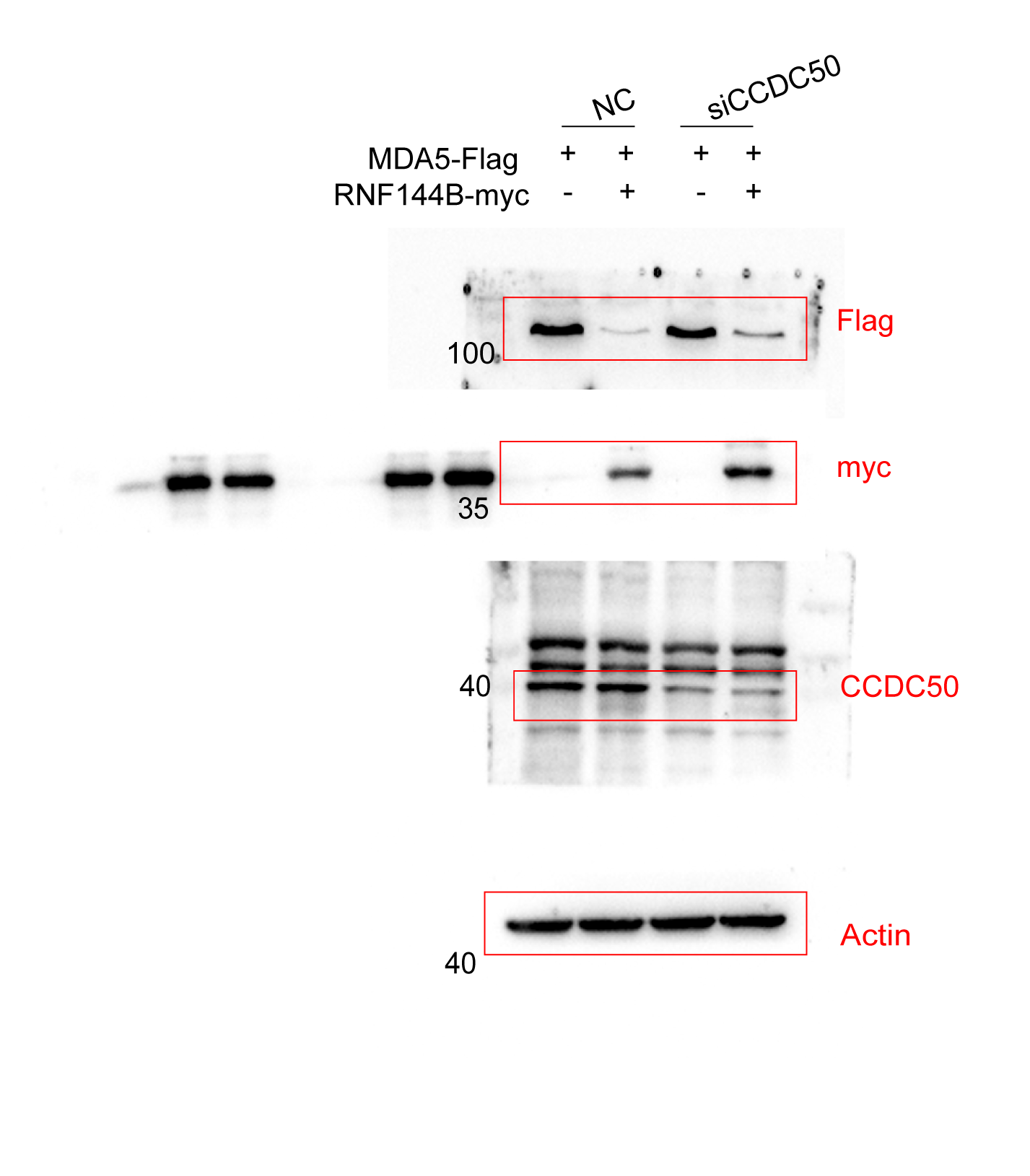

Supplement: Supplementary file 8 — Source data Fig. 5 [file 44319_2024_256_MOESM8_ESM.zip › SourceDateForFigure 5/5I.tif]

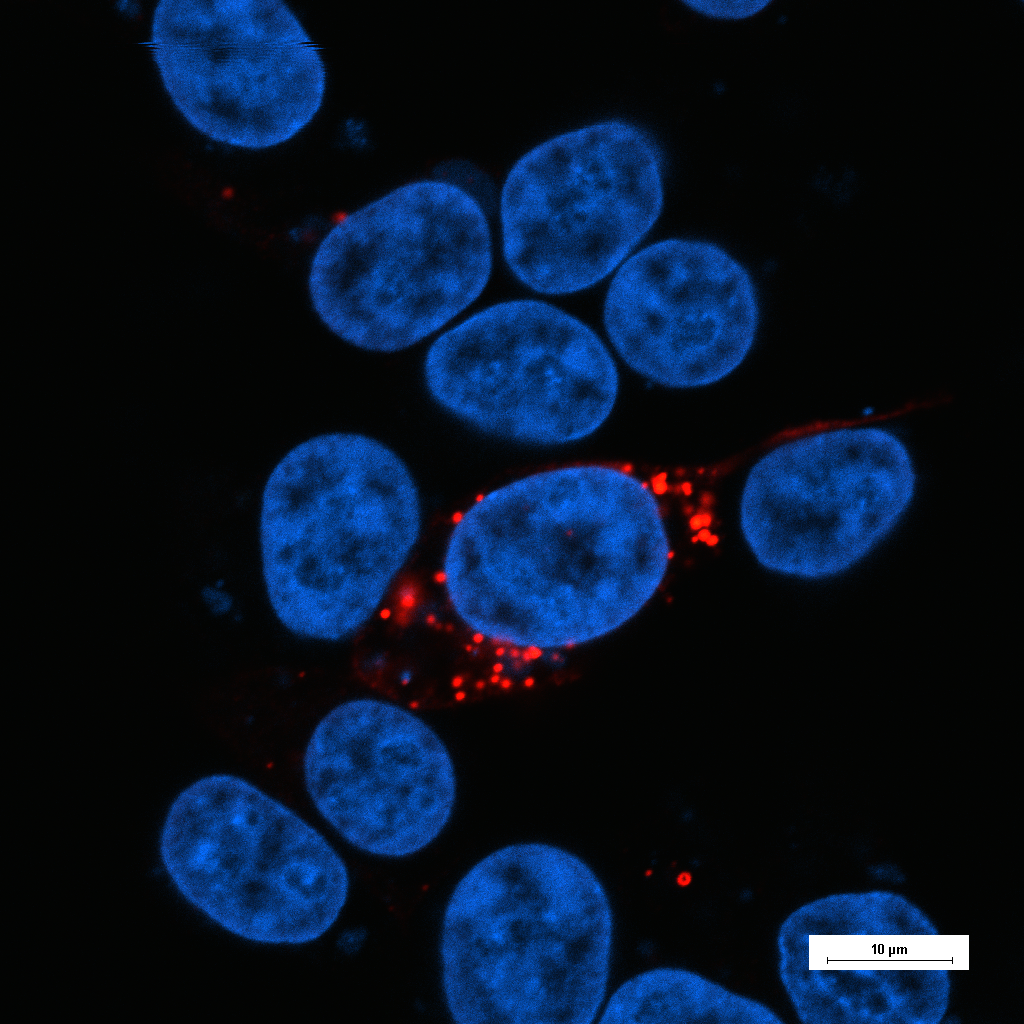

Supplement: Supplementary file 8 — Source data Fig. 5 [file 44319_2024_256_MOESM8_ESM.zip › SourceDateForFigure 5/5J/3,1-p62-4_RGB.tif]

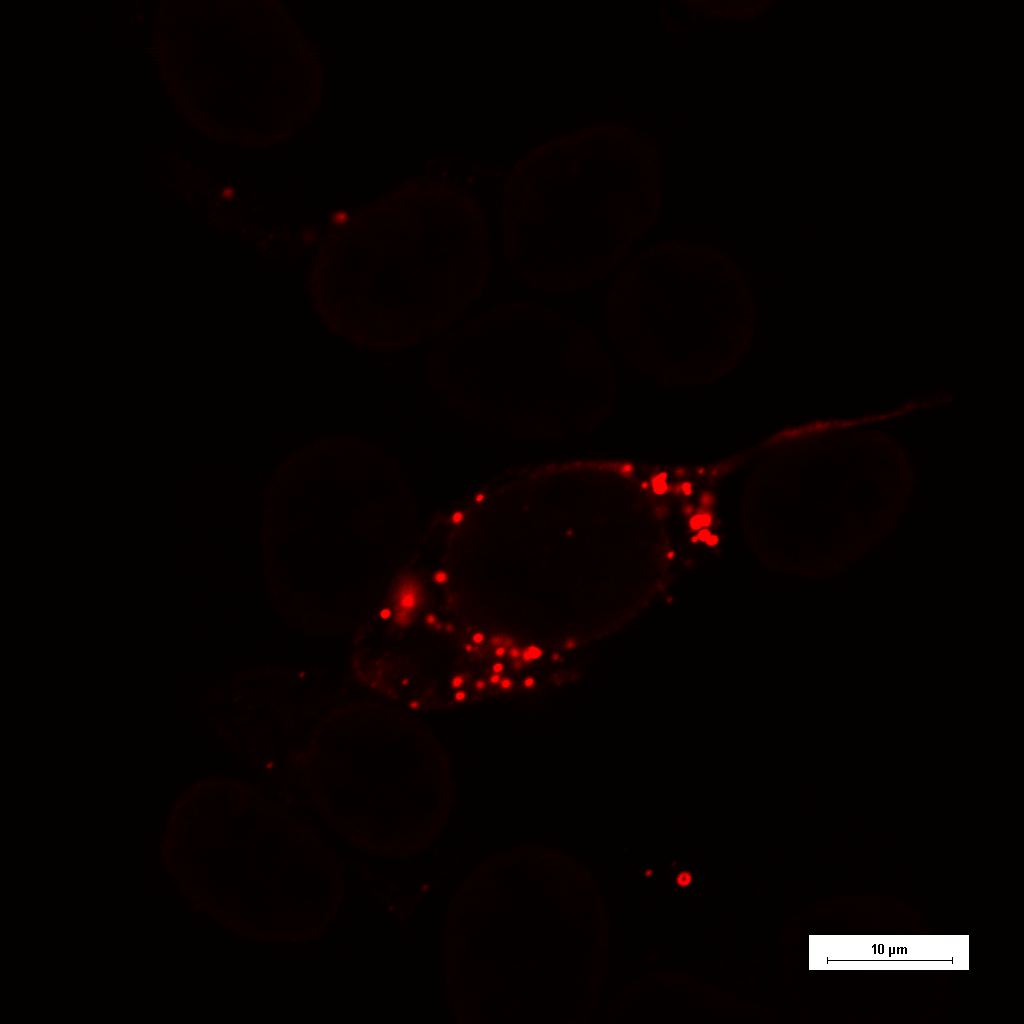

Supplement: Supplementary file 8 — Source data Fig. 5 [file 44319_2024_256_MOESM8_ESM.zip › SourceDateForFigure 5/5J/3,1-p62-4_RGB_Cy3.tif]

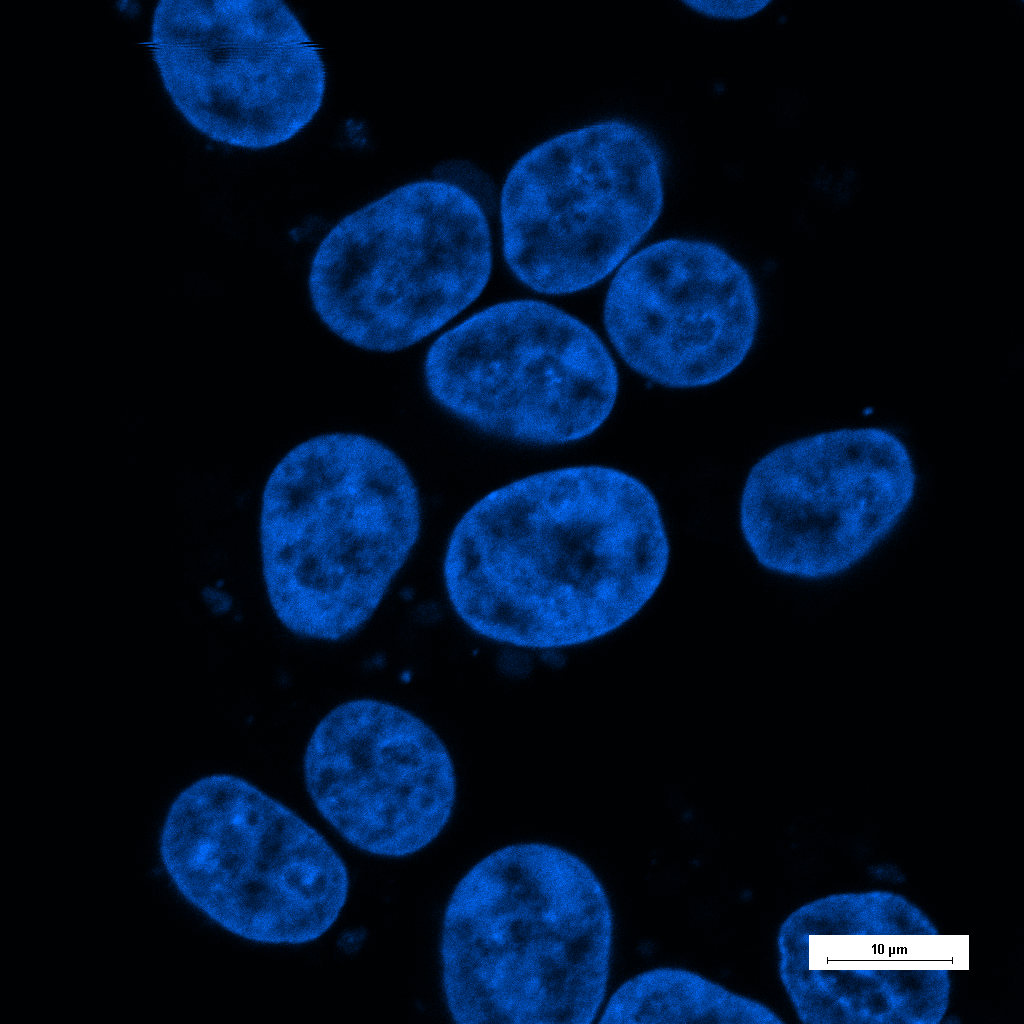

Supplement: Supplementary file 8 — Source data Fig. 5 [file 44319_2024_256_MOESM8_ESM.zip › SourceDateForFigure 5/5J/3,1-p62-4_RGB_DAPI.tif]

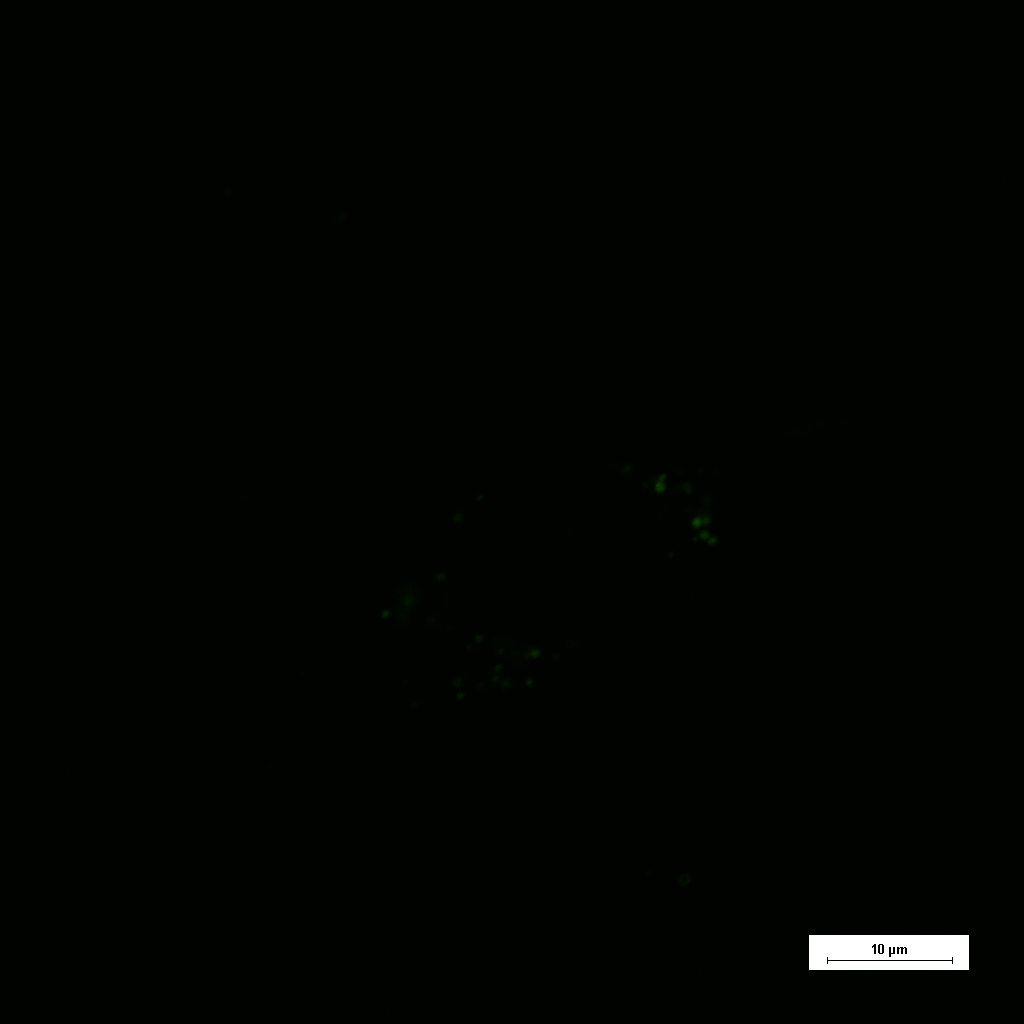

Supplement: Supplementary file 8 — Source data Fig. 5 [file 44319_2024_256_MOESM8_ESM.zip › SourceDateForFigure 5/5J/3,1-p62-4_RGB_FITC .tif]

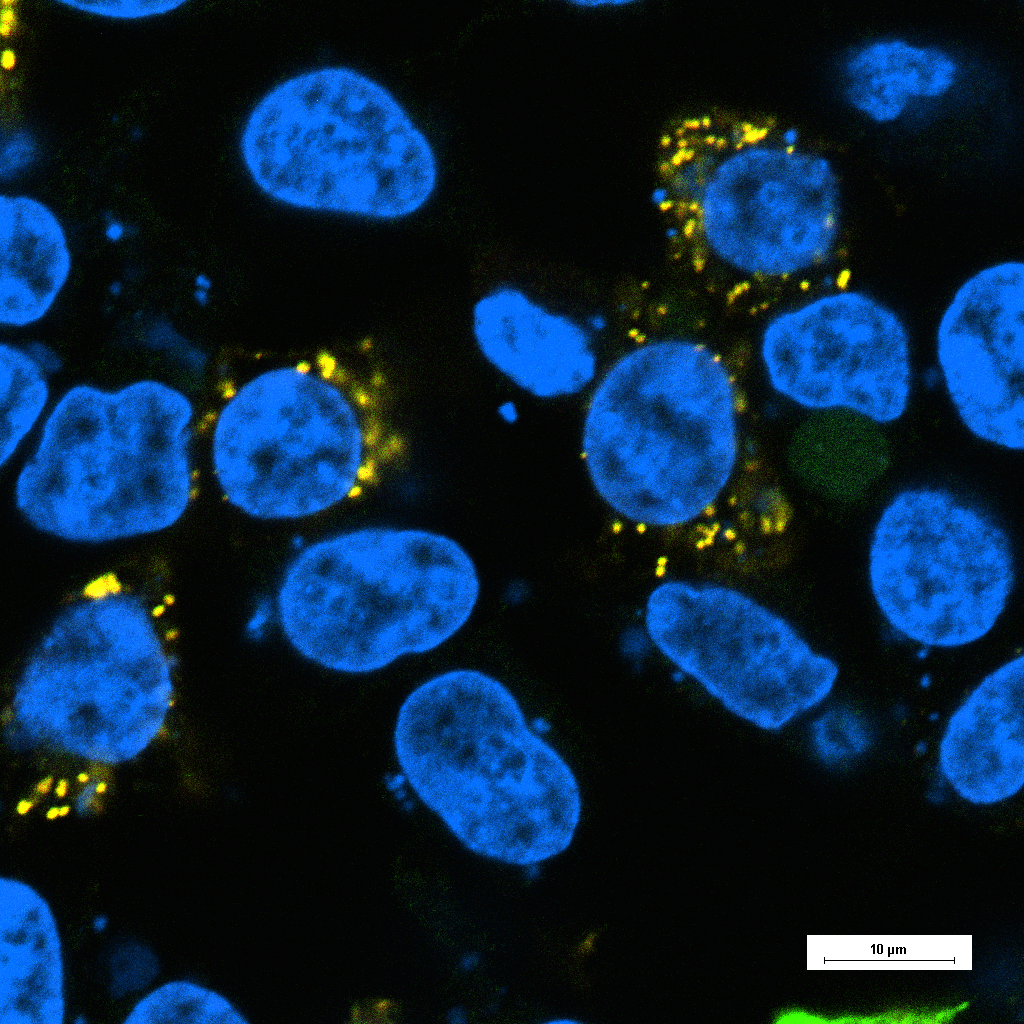

Supplement: Supplementary file 8 — Source data Fig. 5 [file 44319_2024_256_MOESM8_ESM.zip › SourceDateForFigure 5/5J/mda5-p62-4_RGB.tif]

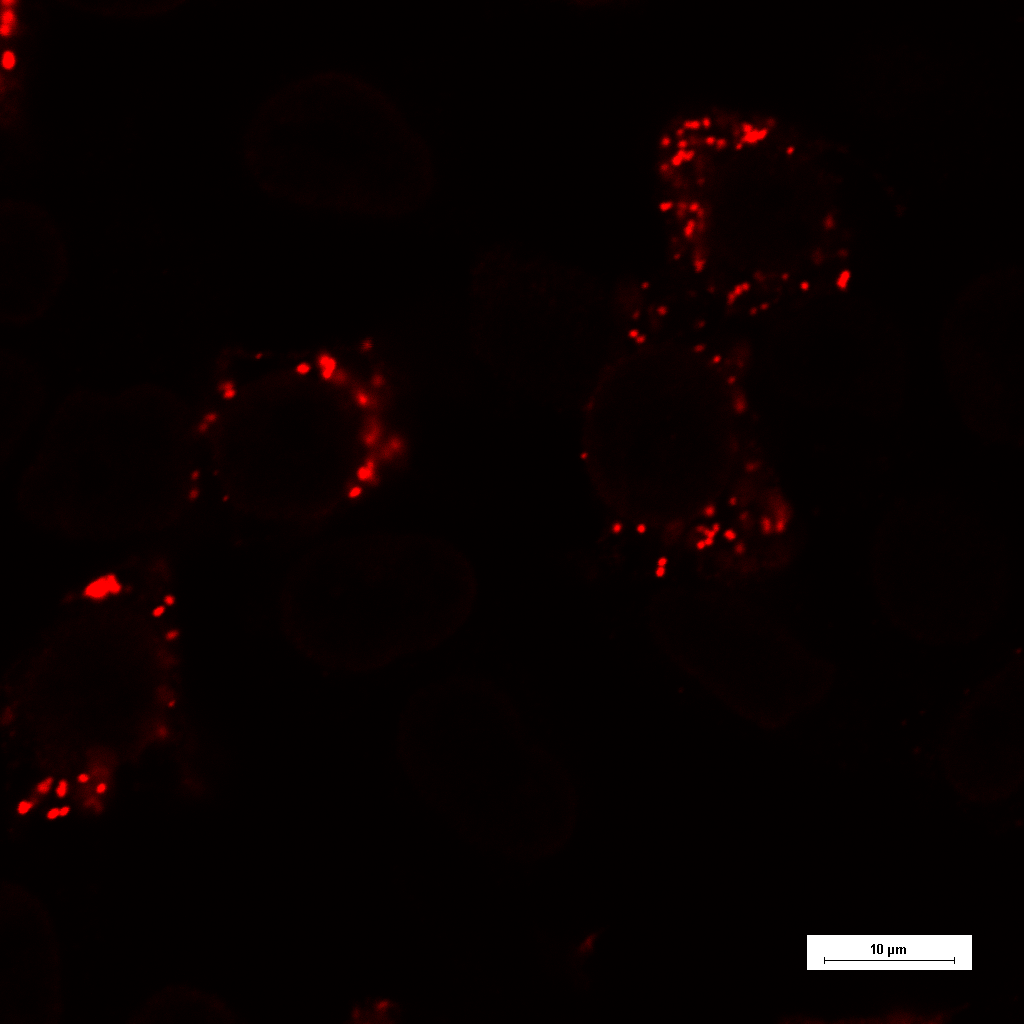

Supplement: Supplementary file 8 — Source data Fig. 5 [file 44319_2024_256_MOESM8_ESM.zip › SourceDateForFigure 5/5J/mda5-p62-4_RGB_Cy3.tif]

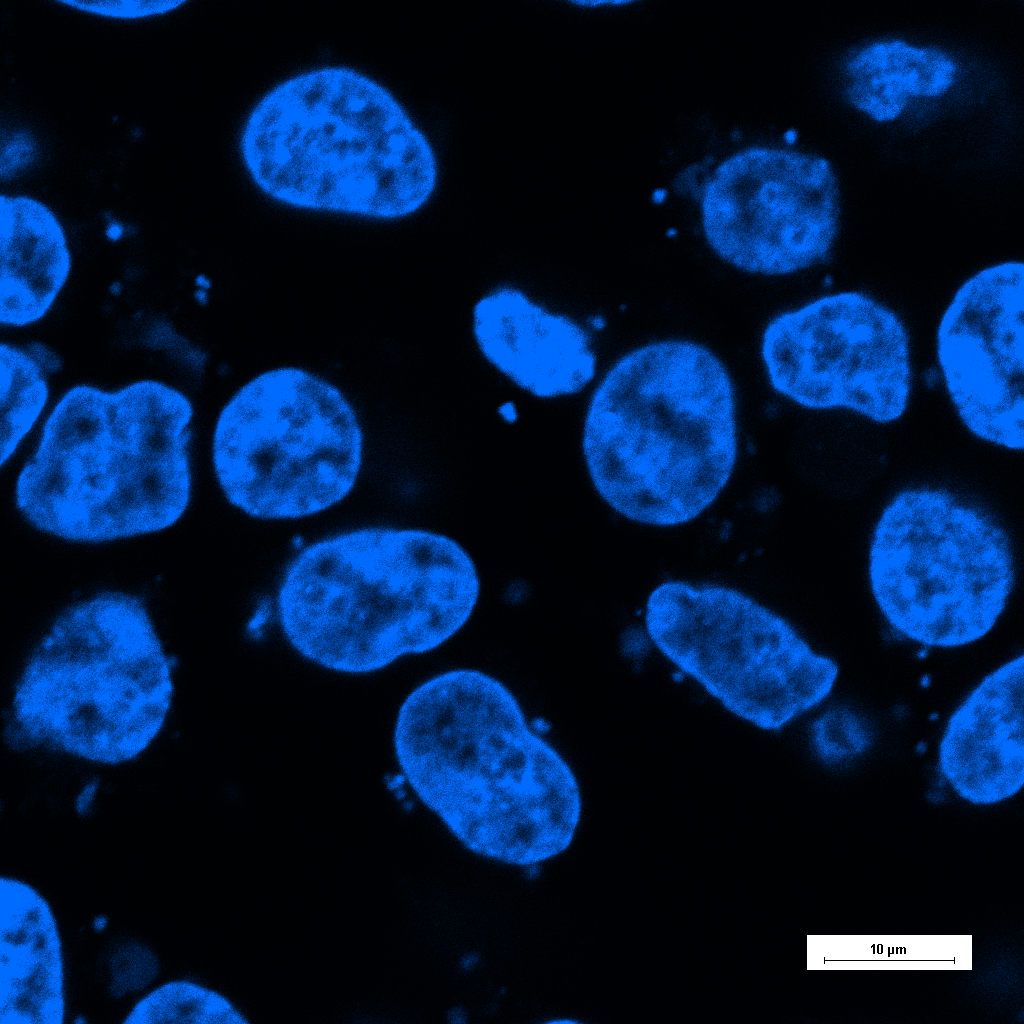

Supplement: Supplementary file 8 — Source data Fig. 5 [file 44319_2024_256_MOESM8_ESM.zip › SourceDateForFigure 5/5J/mda5-p62-4_RGB_DAPI.tif]

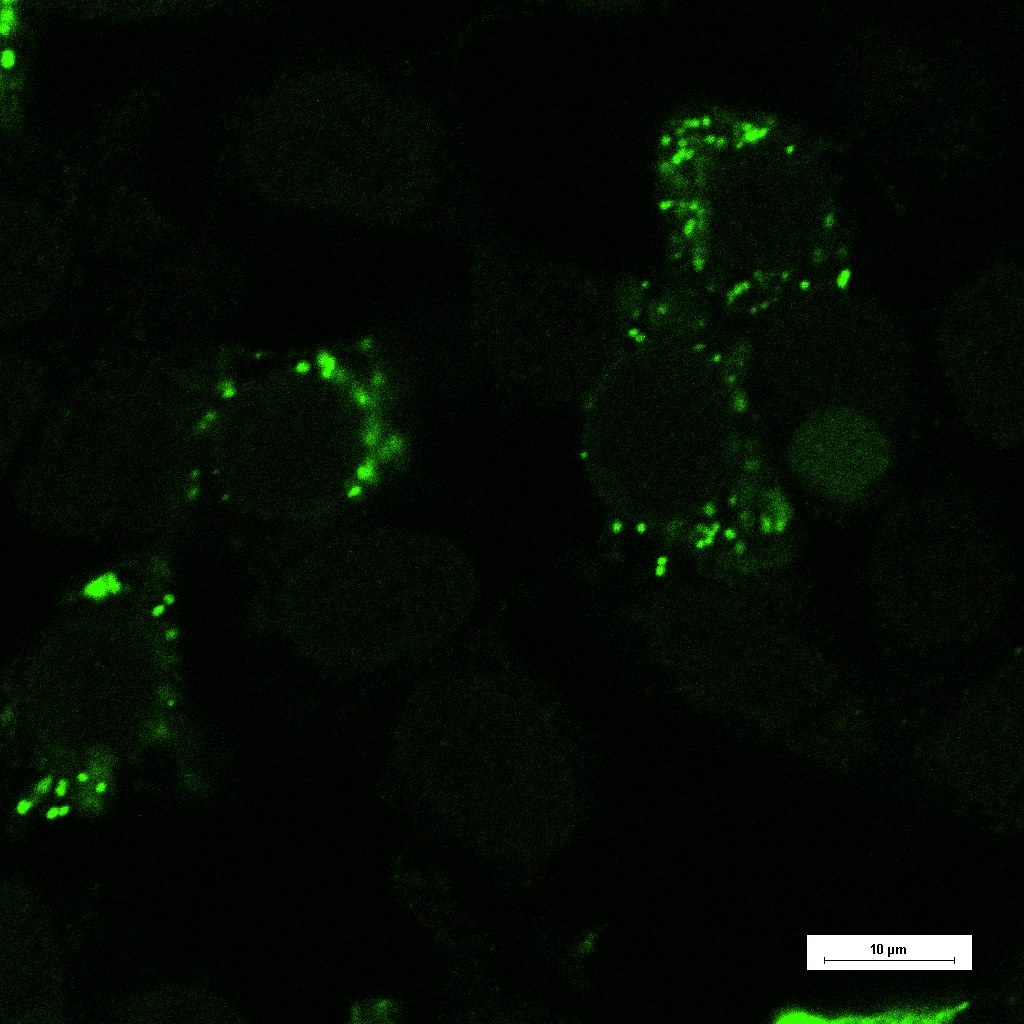

Supplement: Supplementary file 8 — Source data Fig. 5 [file 44319_2024_256_MOESM8_ESM.zip › SourceDateForFigure 5/5J/mda5-p62-4_RGB_FITC .tif]

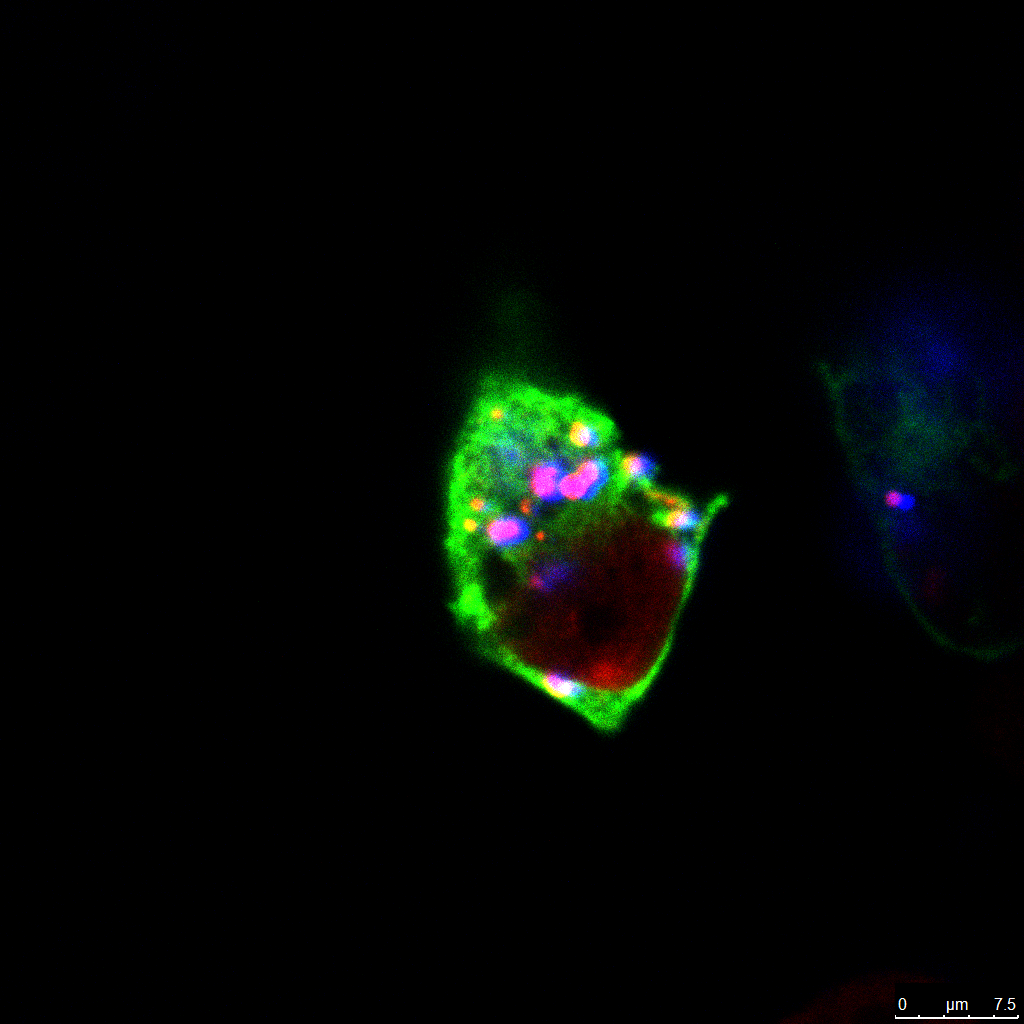

Supplement: Supplementary file 8 — Source data Fig. 5 [file 44319_2024_256_MOESM8_ESM.zip › SourceDateForFigure 5/5K/Experiment001-27-33R/Experiment001_3-4_z0.tif]

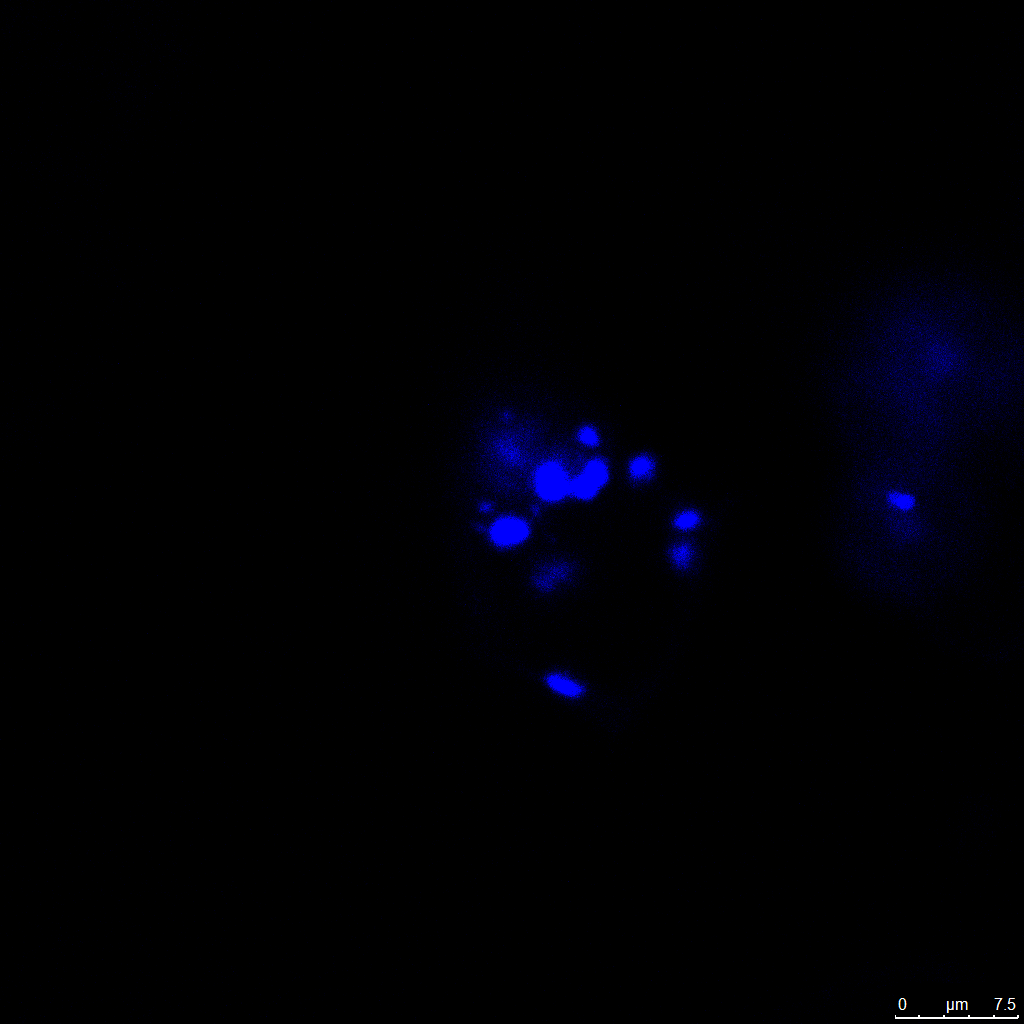

Supplement: Supplementary file 8 — Source data Fig. 5 [file 44319_2024_256_MOESM8_ESM.zip › SourceDateForFigure 5/5K/Experiment001-27-33R/Experiment001_3-4_z0_ch00.tif]

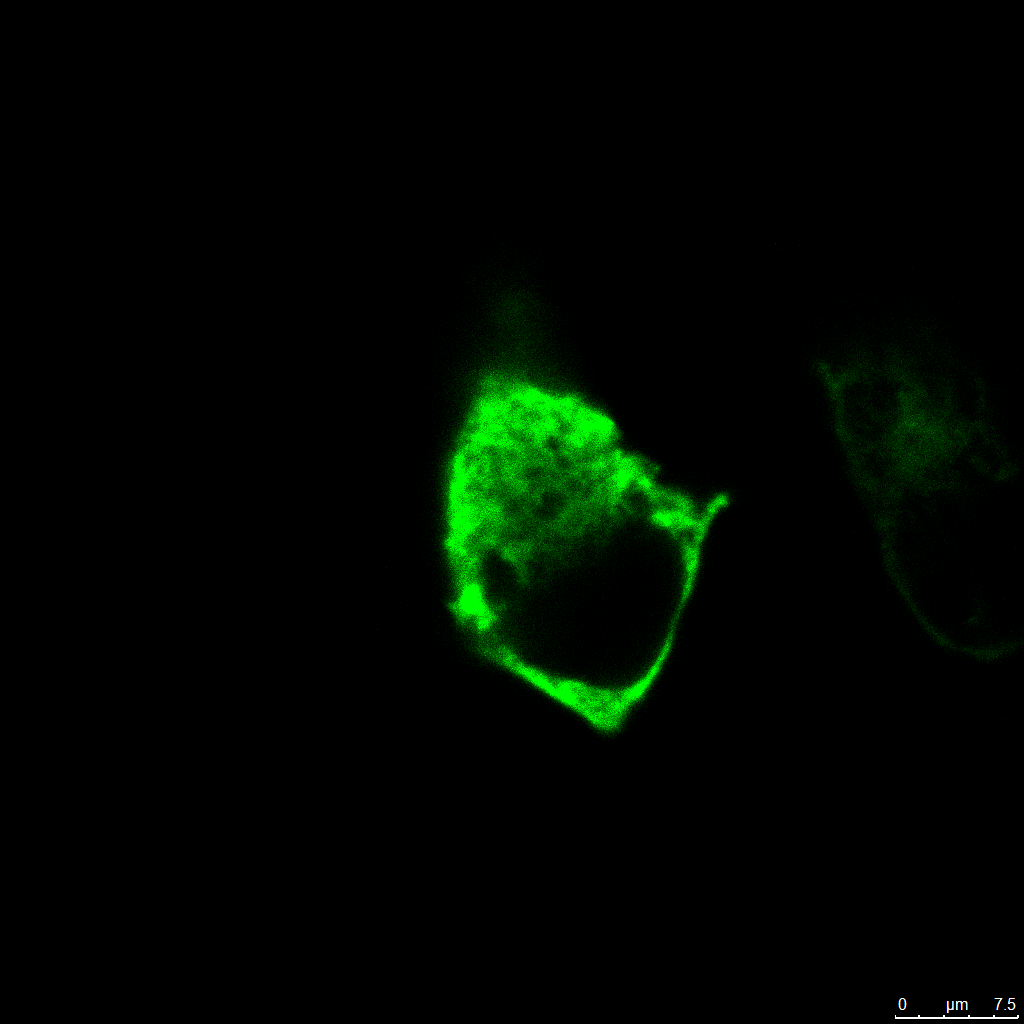

Supplement: Supplementary file 8 — Source data Fig. 5 [file 44319_2024_256_MOESM8_ESM.zip › SourceDateForFigure 5/5K/Experiment001-27-33R/Experiment001_3-4_z0_ch01.tif]

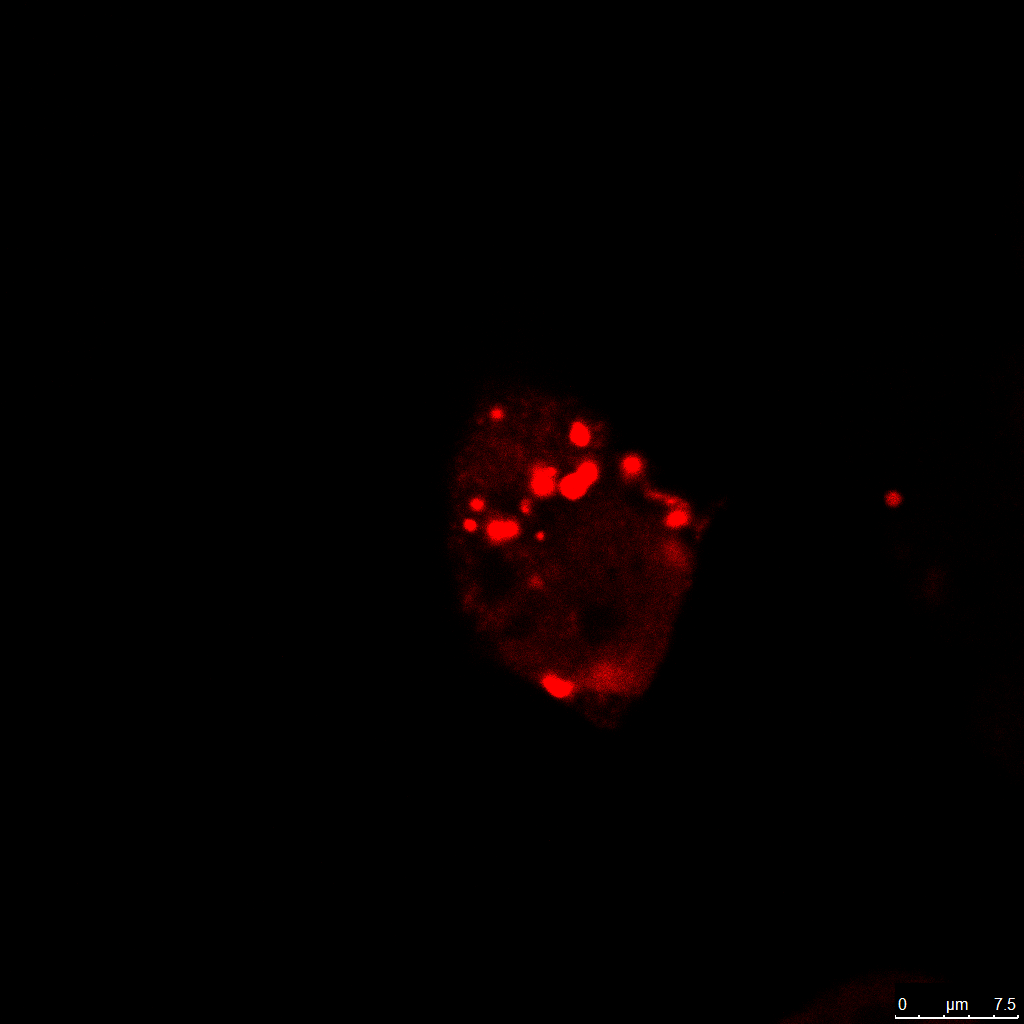

Supplement: Supplementary file 8 — Source data Fig. 5 [file 44319_2024_256_MOESM8_ESM.zip › SourceDateForFigure 5/5K/Experiment001-27-33R/Experiment001_3-4_z0_ch02.tif]

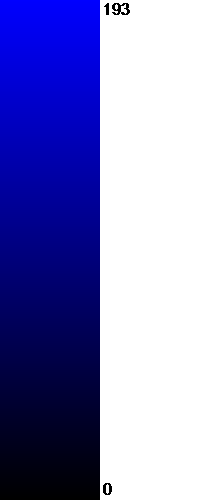

Supplement: Supplementary file 8 — Source data Fig. 5 [file 44319_2024_256_MOESM8_ESM.zip › SourceDateForFigure 5/5K/Experiment001-27-33R/MetaData/Experiment001_3-4ch0LUT.png]

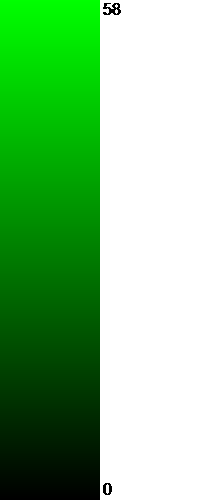

Supplement: Supplementary file 8 — Source data Fig. 5 [file 44319_2024_256_MOESM8_ESM.zip › SourceDateForFigure 5/5K/Experiment001-27-33R/MetaData/Experiment001_3-4ch1LUT.png]

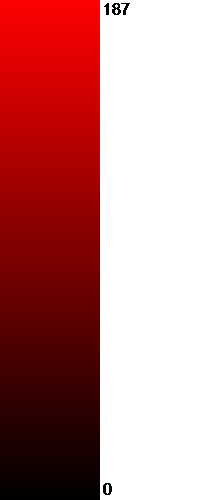

Supplement: Supplementary file 8 — Source data Fig. 5 [file 44319_2024_256_MOESM8_ESM.zip › SourceDateForFigure 5/5K/Experiment001-27-33R/MetaData/Experiment001_3-4ch2LUT.png]

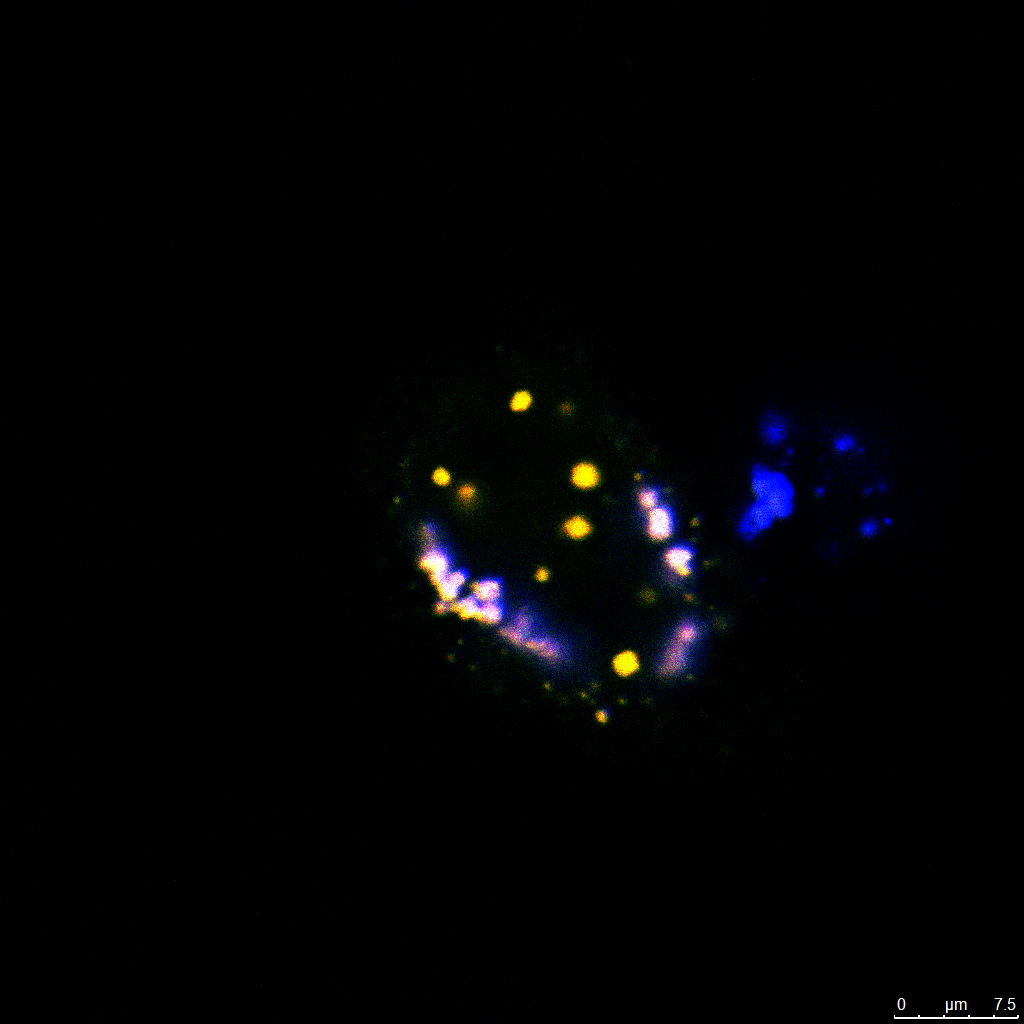

Supplement: Supplementary file 8 — Source data Fig. 5 [file 44319_2024_256_MOESM8_ESM.zip › SourceDateForFigure 5/5K/Experiment001_27O/Experiment001_3-6_z0.tif]

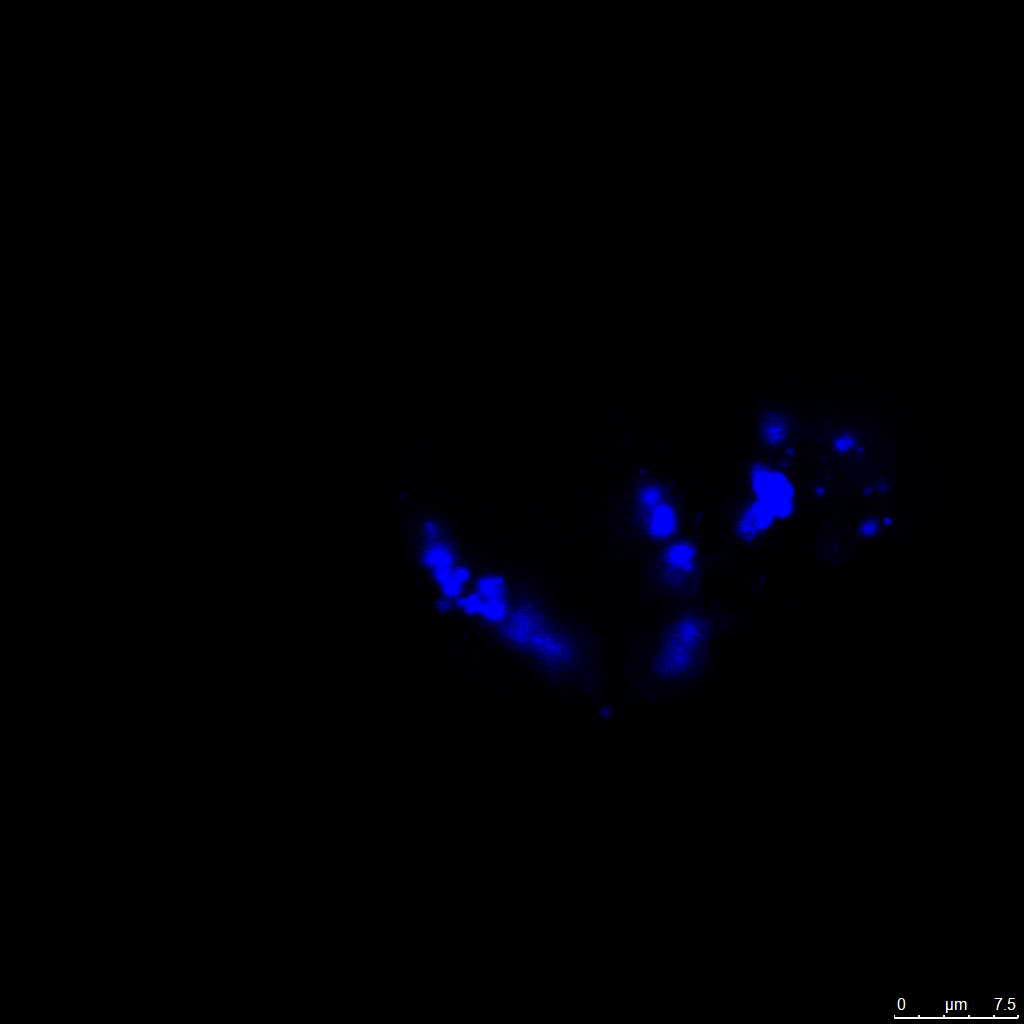

Supplement: Supplementary file 8 — Source data Fig. 5 [file 44319_2024_256_MOESM8_ESM.zip › SourceDateForFigure 5/5K/Experiment001_27O/Experiment001_3-6_z0_ch00.tif]

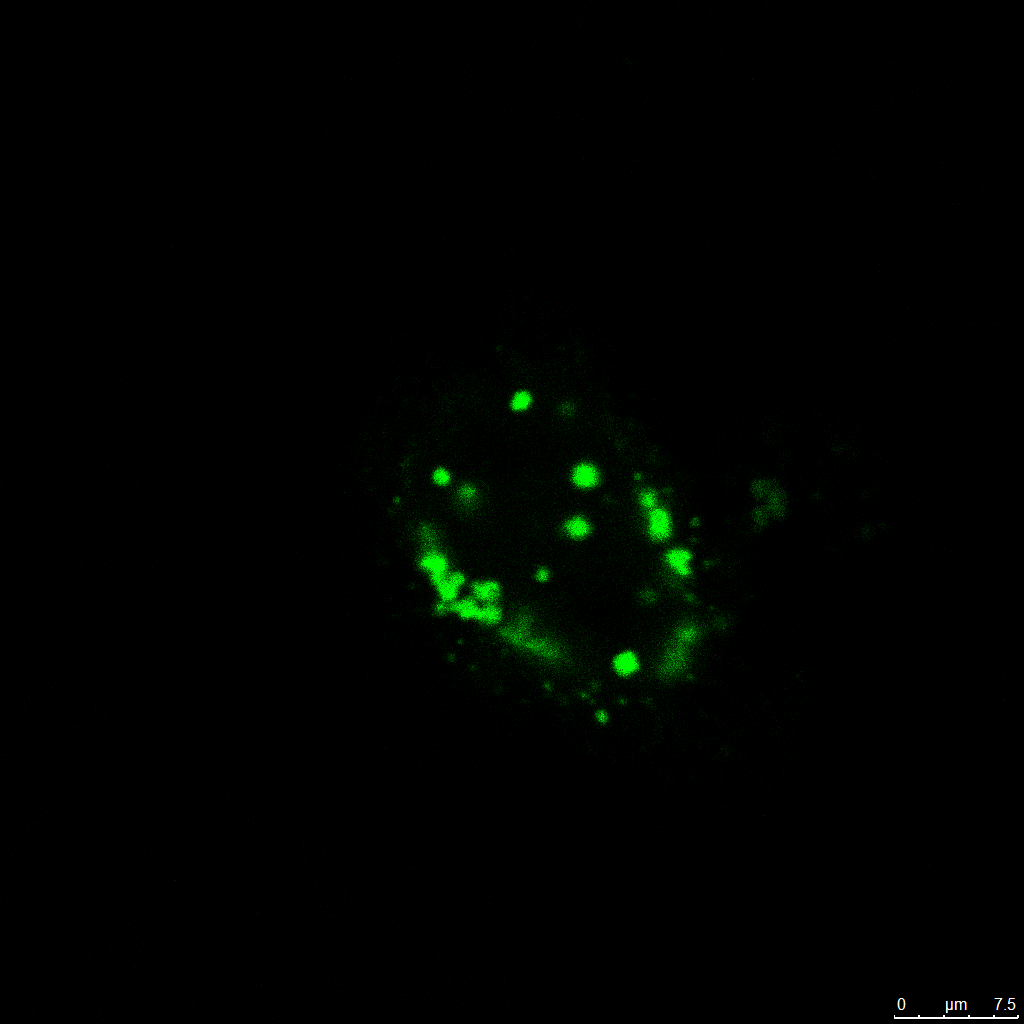

Supplement: Supplementary file 8 — Source data Fig. 5 [file 44319_2024_256_MOESM8_ESM.zip › SourceDateForFigure 5/5K/Experiment001_27O/Experiment001_3-6_z0_ch01.tif]

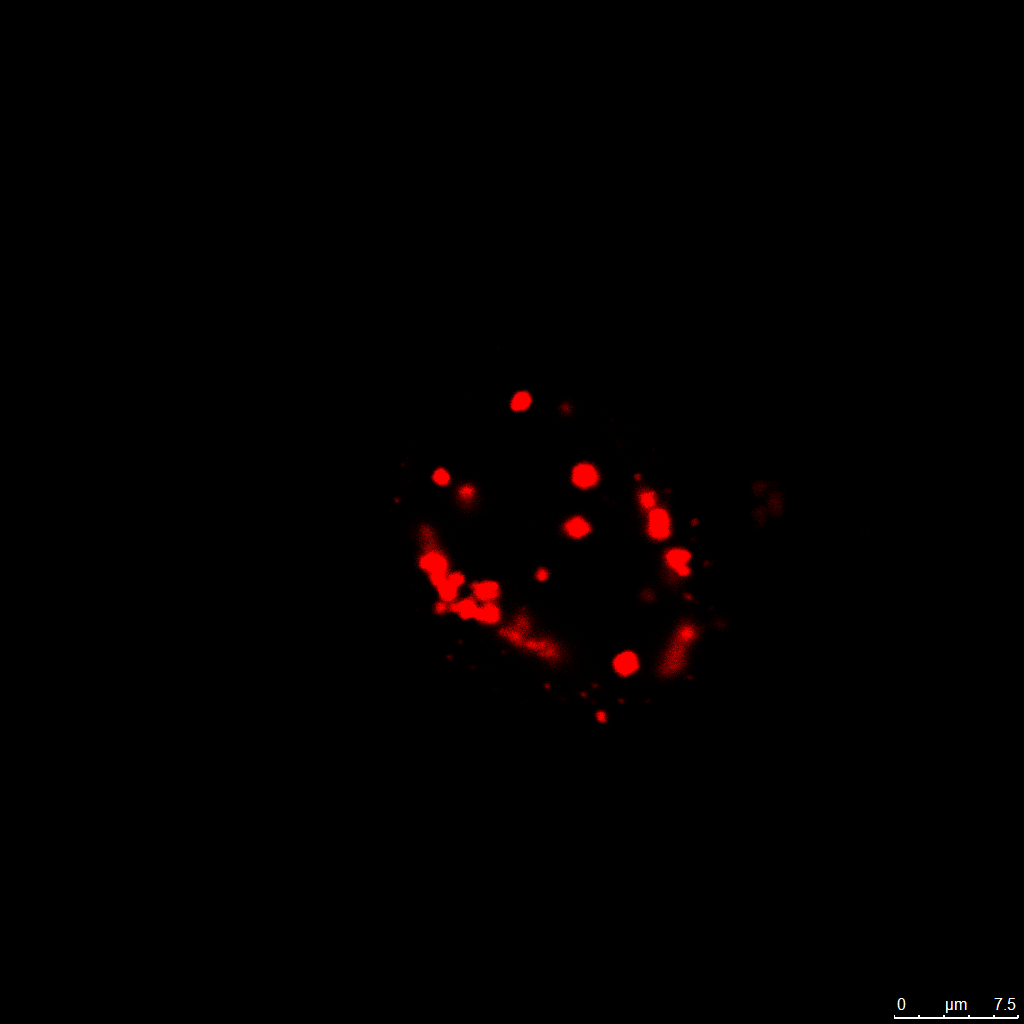

Supplement: Supplementary file 8 — Source data Fig. 5 [file 44319_2024_256_MOESM8_ESM.zip › SourceDateForFigure 5/5K/Experiment001_27O/Experiment001_3-6_z0_ch02.tif]

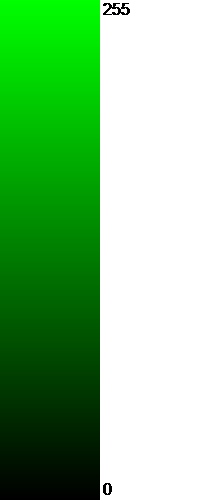

Supplement: Supplementary file 8 — Source data Fig. 5 [file 44319_2024_256_MOESM8_ESM.zip › SourceDateForFigure 5/5K/Experiment001_27O/MetaData/Experiment001_3-6ch1LUT.png]

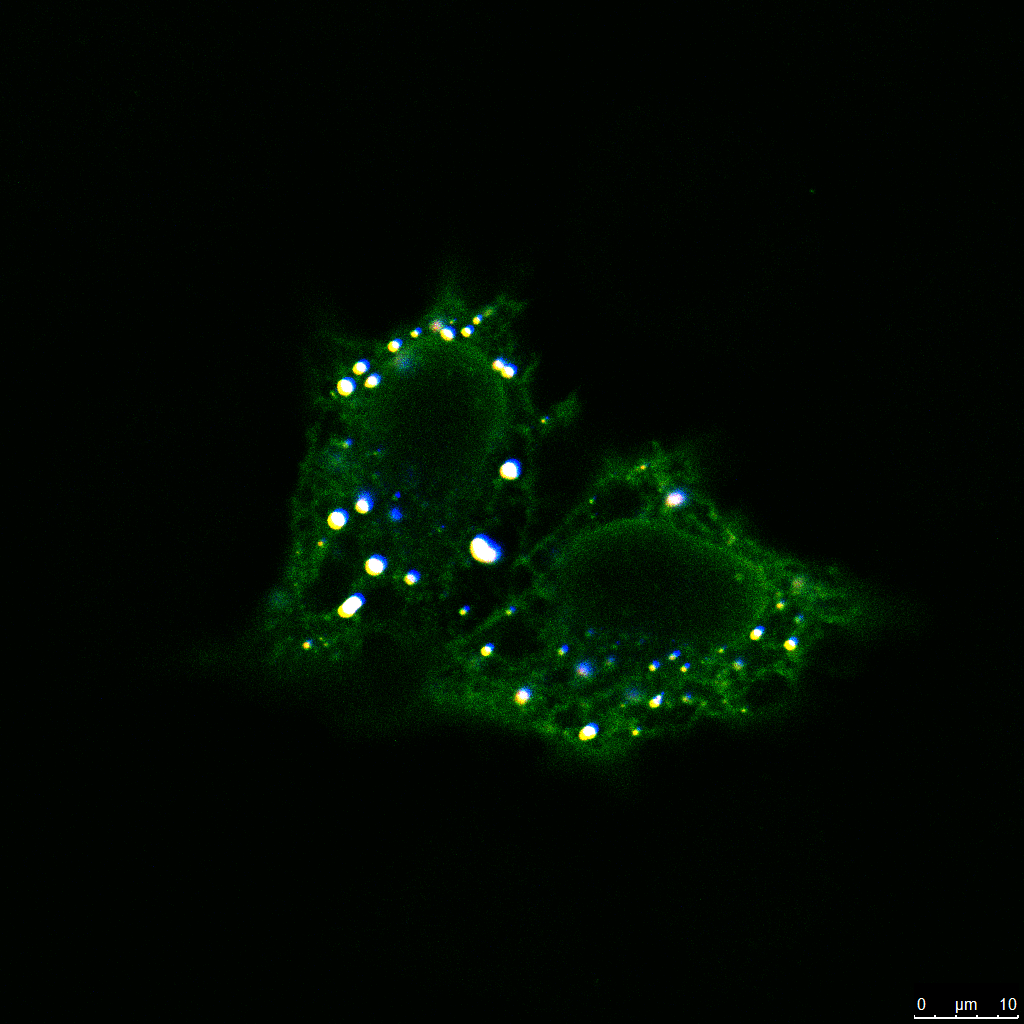

Supplement: Supplementary file 8 — Source data Fig. 5 [file 44319_2024_256_MOESM8_ESM.zip › SourceDateForFigure 5/5K/Experiment001_33O/Experiment001_lgx2-9_z0.tif]

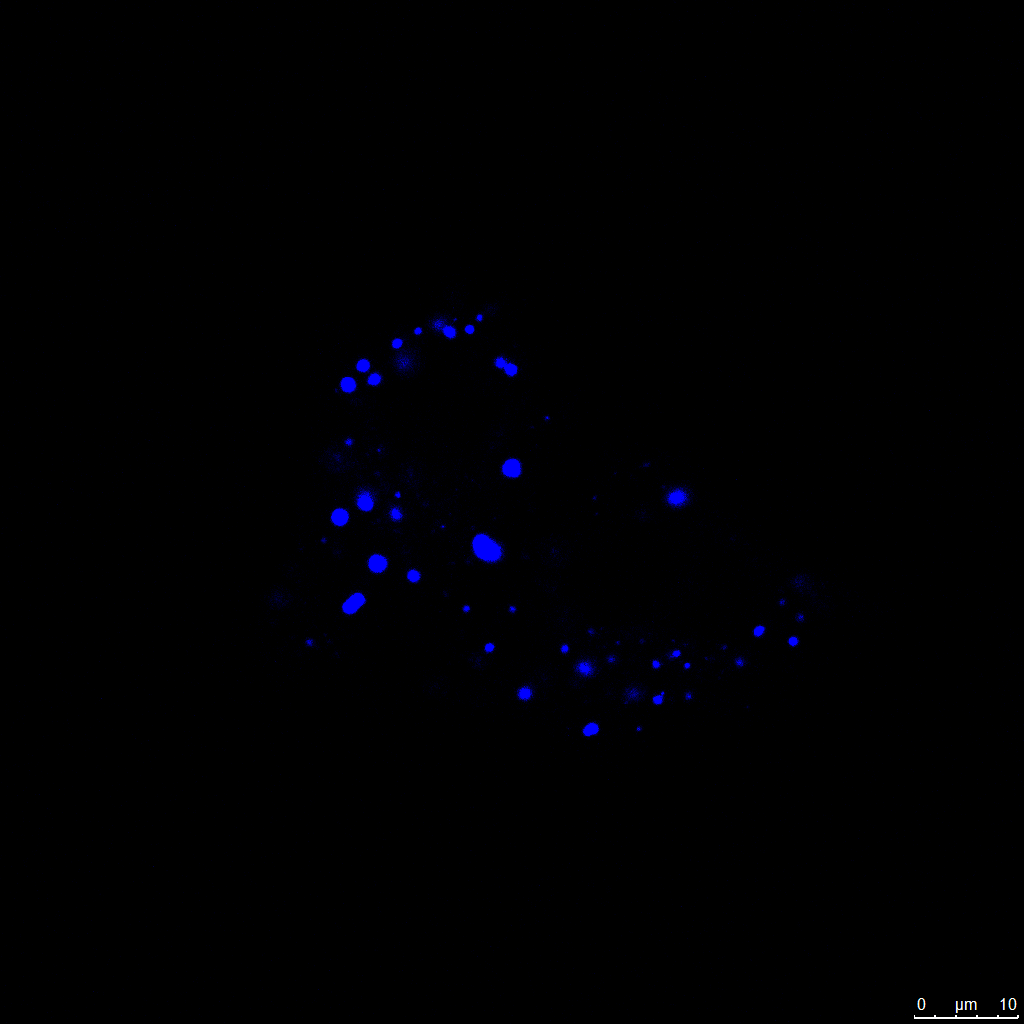

Supplement: Supplementary file 8 — Source data Fig. 5 [file 44319_2024_256_MOESM8_ESM.zip › SourceDateForFigure 5/5K/Experiment001_33O/Experiment001_lgx2-9_z0_ch00.tif]

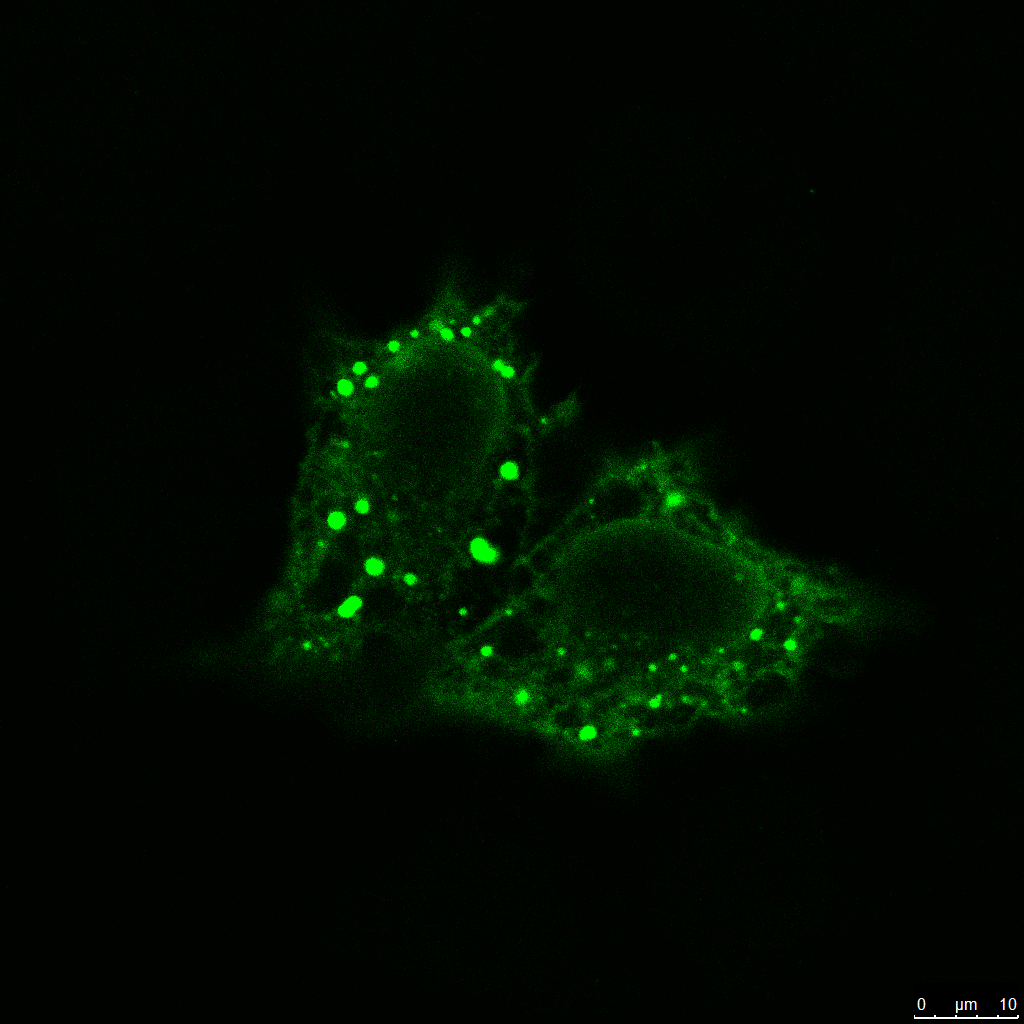

Supplement: Supplementary file 8 — Source data Fig. 5 [file 44319_2024_256_MOESM8_ESM.zip › SourceDateForFigure 5/5K/Experiment001_33O/Experiment001_lgx2-9_z0_ch01.tif]

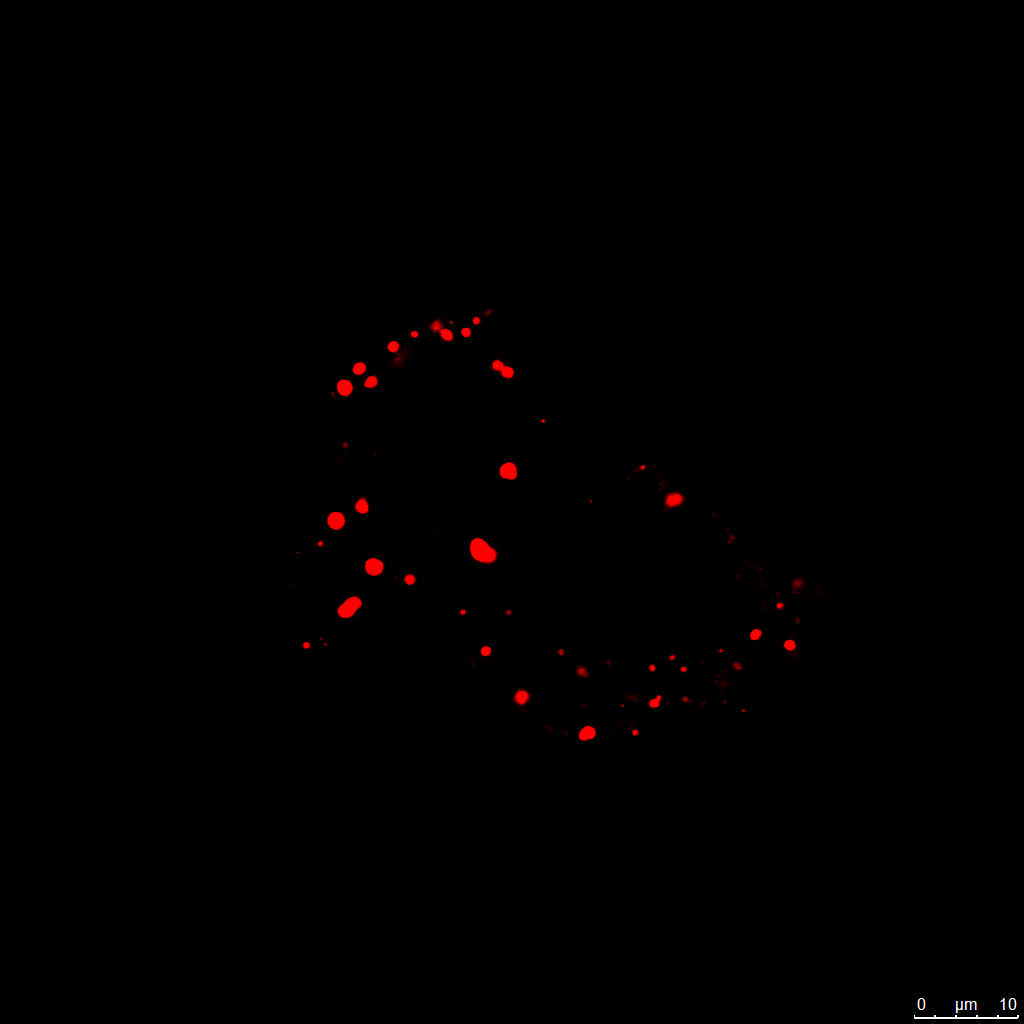

Supplement: Supplementary file 8 — Source data Fig. 5 [file 44319_2024_256_MOESM8_ESM.zip › SourceDateForFigure 5/5K/Experiment001_33O/Experiment001_lgx2-9_z0_ch02.tif]

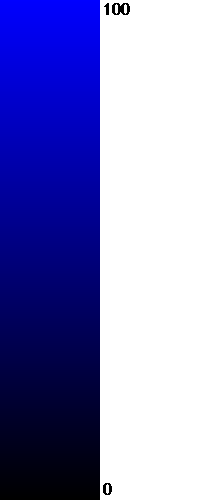

Supplement: Supplementary file 8 — Source data Fig. 5 [file 44319_2024_256_MOESM8_ESM.zip › SourceDateForFigure 5/5K/Experiment001_33O/MetaData/Experiment001_lgx2-9ch0LUT.png]

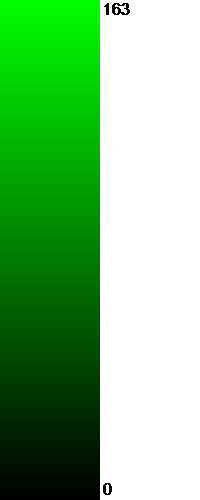

Supplement: Supplementary file 8 — Source data Fig. 5 [file 44319_2024_256_MOESM8_ESM.zip › SourceDateForFigure 5/5K/Experiment001_33O/MetaData/Experiment001_lgx2-9ch1LUT.png]

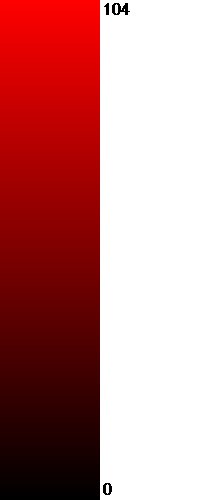

Supplement: Supplementary file 8 — Source data Fig. 5 [file 44319_2024_256_MOESM8_ESM.zip › SourceDateForFigure 5/5K/Experiment001_33O/MetaData/Experiment001_lgx2-9ch2LUT.png]

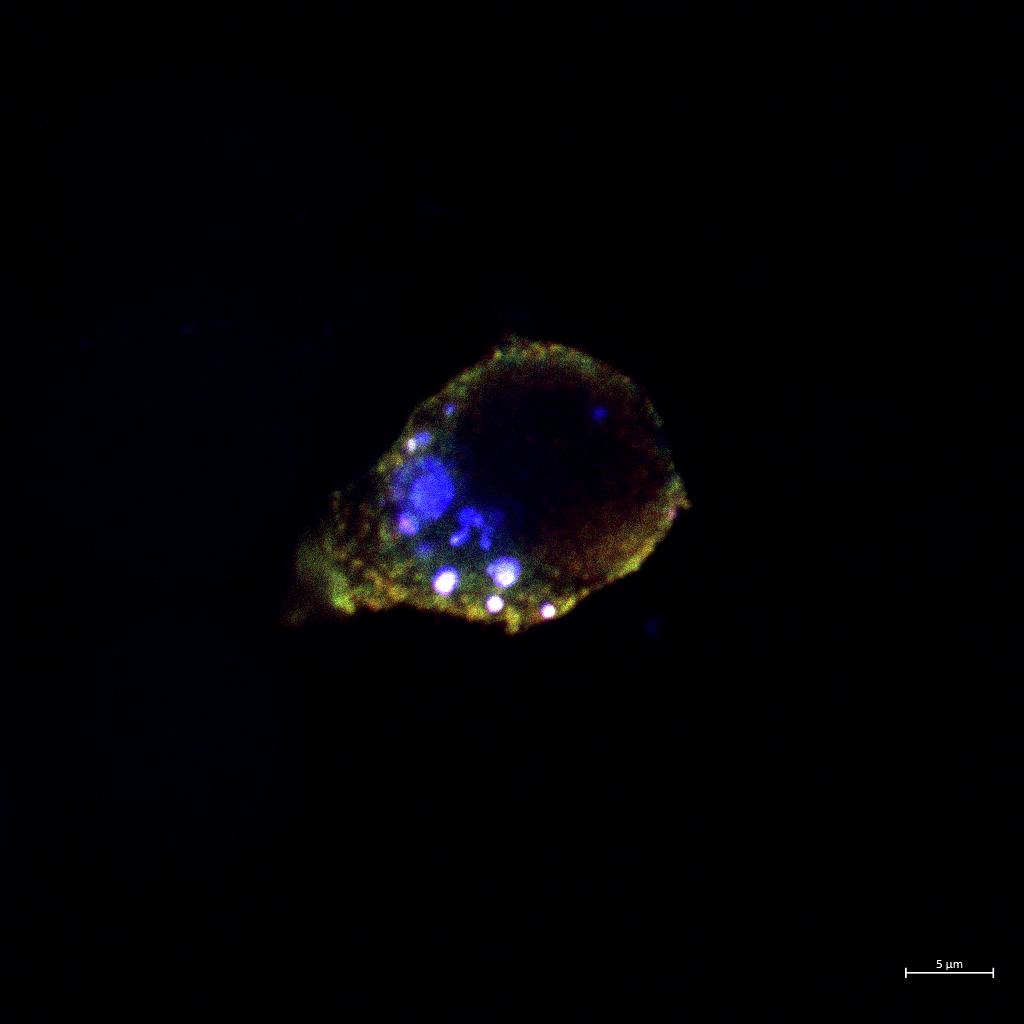

Supplement: Supplementary file 8 — Source data Fig. 5 [file 44319_2024_256_MOESM8_ESM.zip › SourceDateForFigure 5/5K/Snap-27R/Snap-1883-3-3_c1-3.tif]

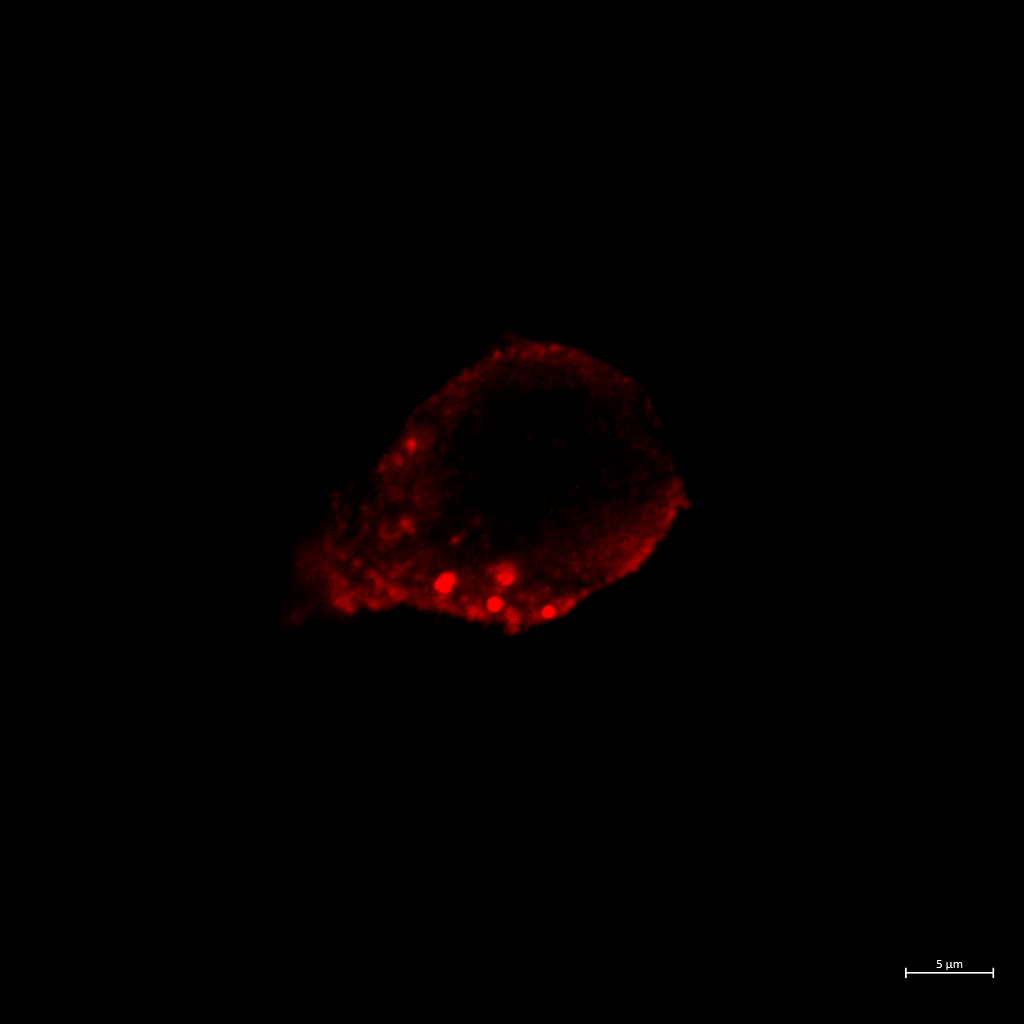

Supplement: Supplementary file 8 — Source data Fig. 5 [file 44319_2024_256_MOESM8_ESM.zip › SourceDateForFigure 5/5K/Snap-27R/Snap-1883-3-3_c1.tif]

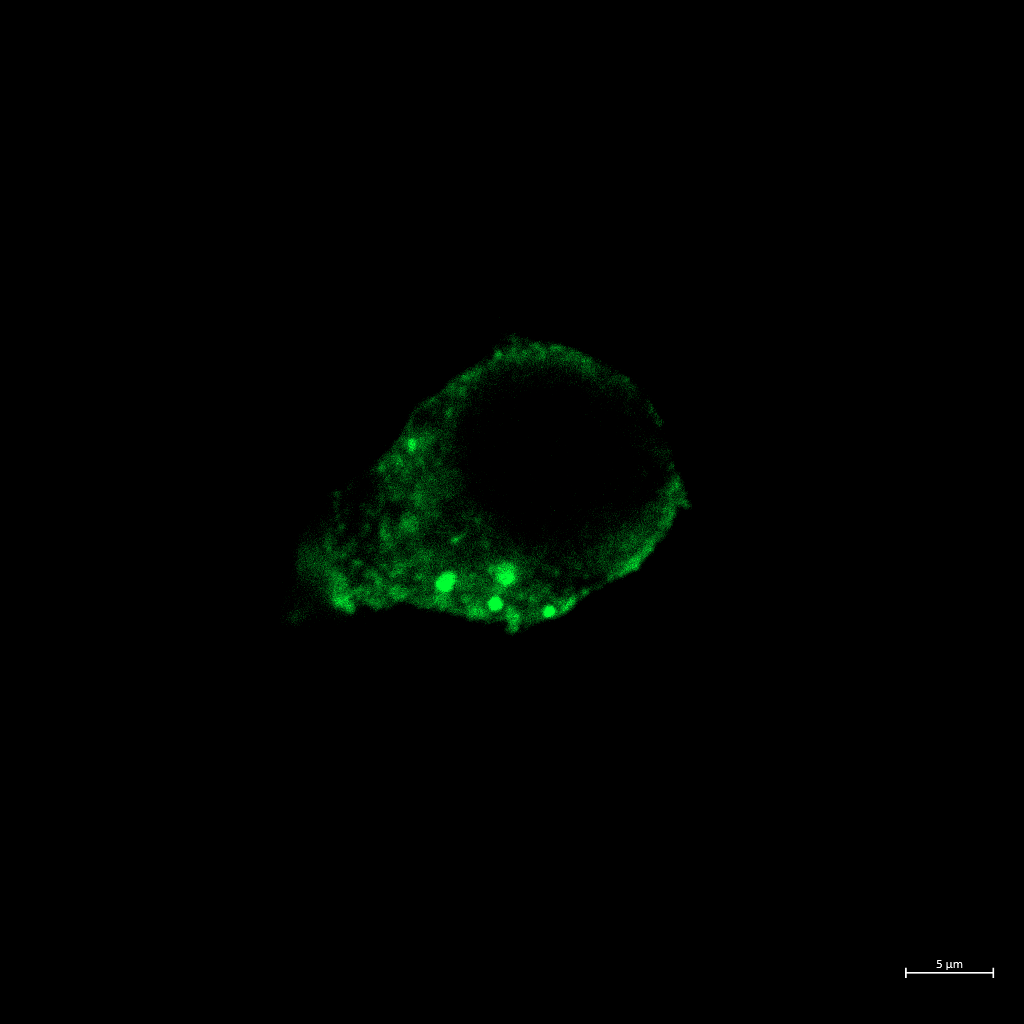

Supplement: Supplementary file 8 — Source data Fig. 5 [file 44319_2024_256_MOESM8_ESM.zip › SourceDateForFigure 5/5K/Snap-27R/Snap-1883-3-3_c2.tif]

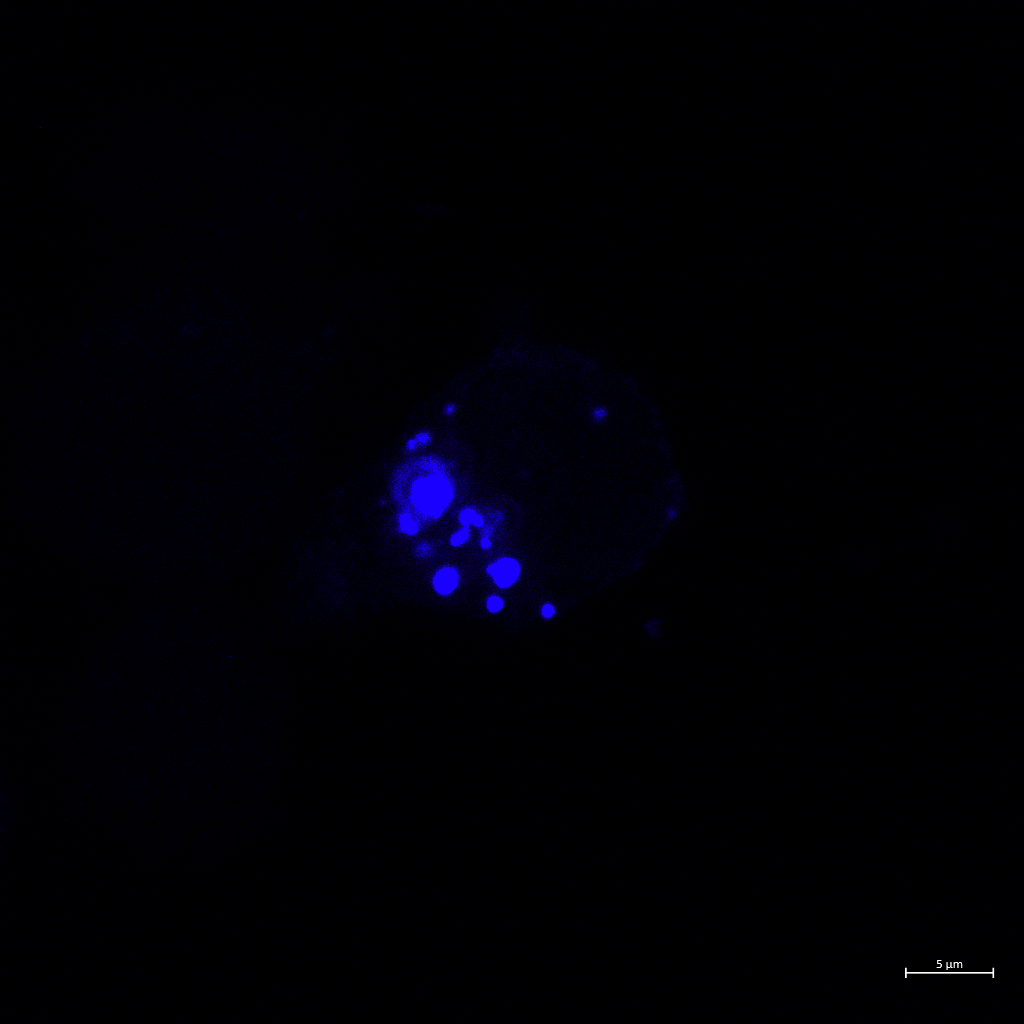

Supplement: Supplementary file 8 — Source data Fig. 5 [file 44319_2024_256_MOESM8_ESM.zip › SourceDateForFigure 5/5K/Snap-27R/Snap-1883-3-3_c3.tif]

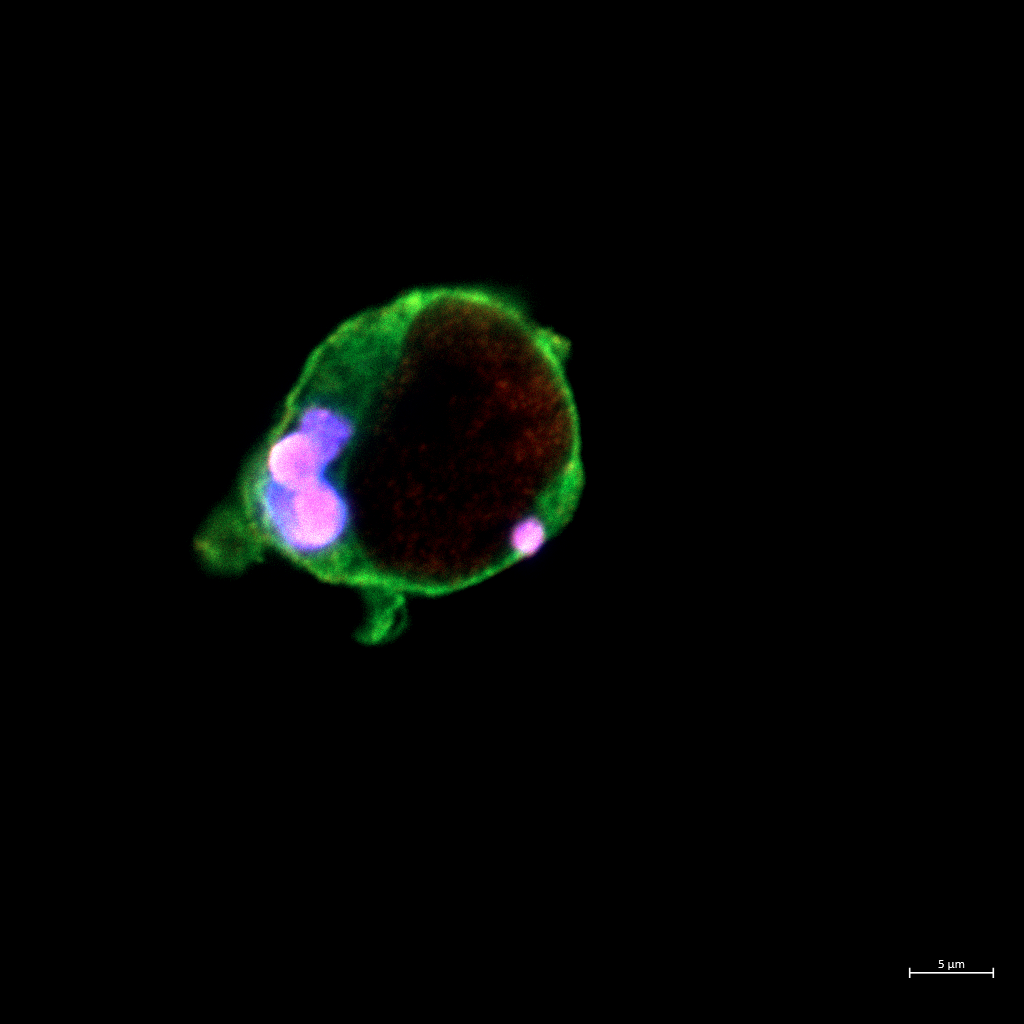

Supplement: Supplementary file 8 — Source data Fig. 5 [file 44319_2024_256_MOESM8_ESM.zip › SourceDateForFigure 5/5K/Snap-33R/Snap-1877-1-7_c1-3.tif]

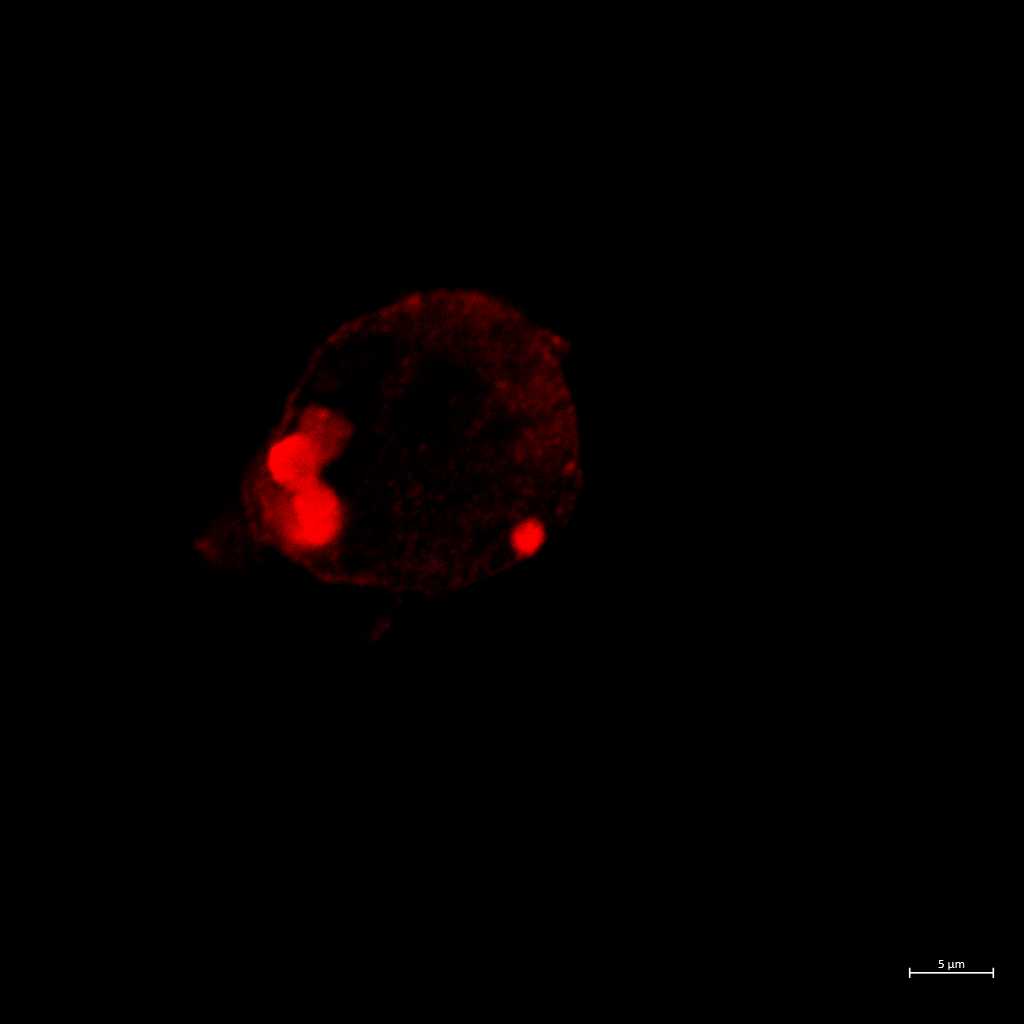

Supplement: Supplementary file 8 — Source data Fig. 5 [file 44319_2024_256_MOESM8_ESM.zip › SourceDateForFigure 5/5K/Snap-33R/Snap-1877-1-7_c1.tif]

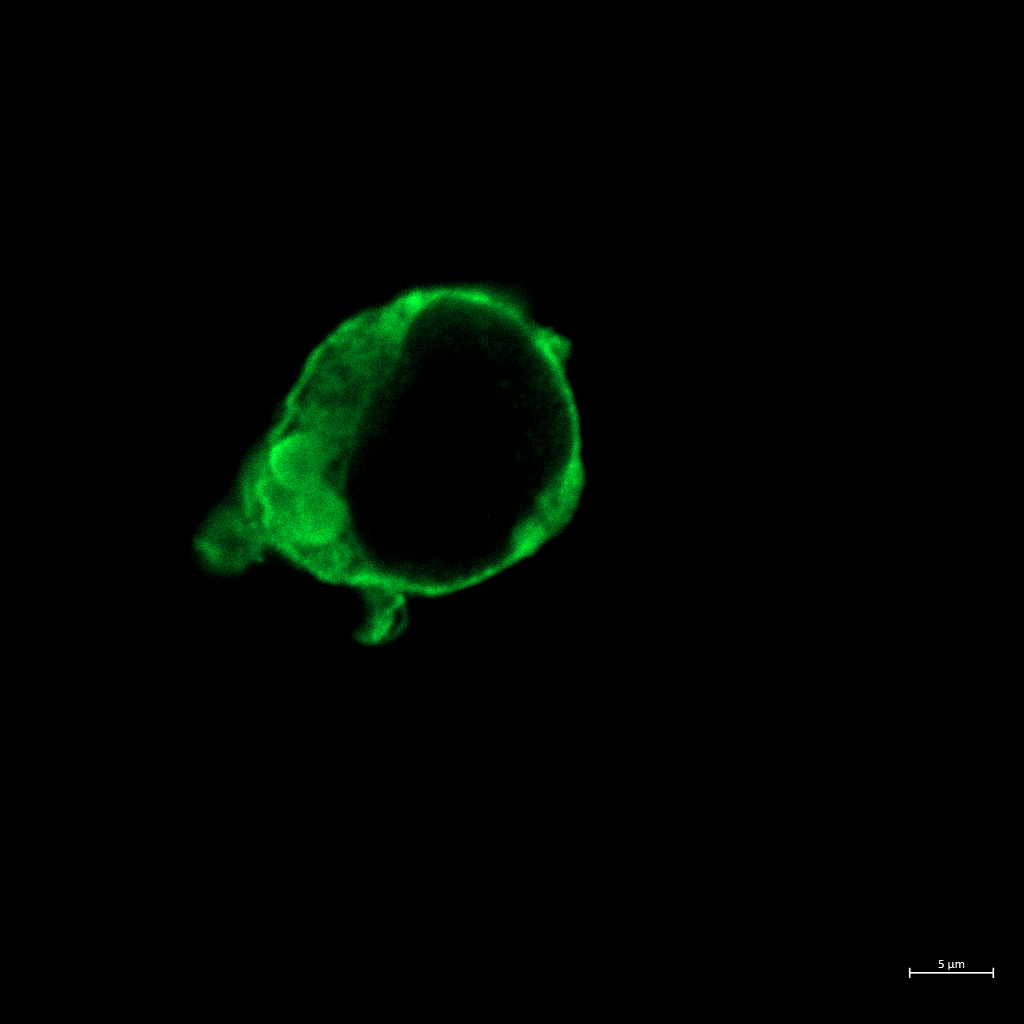

Supplement: Supplementary file 8 — Source data Fig. 5 [file 44319_2024_256_MOESM8_ESM.zip › SourceDateForFigure 5/5K/Snap-33R/Snap-1877-1-7_c2.tif]

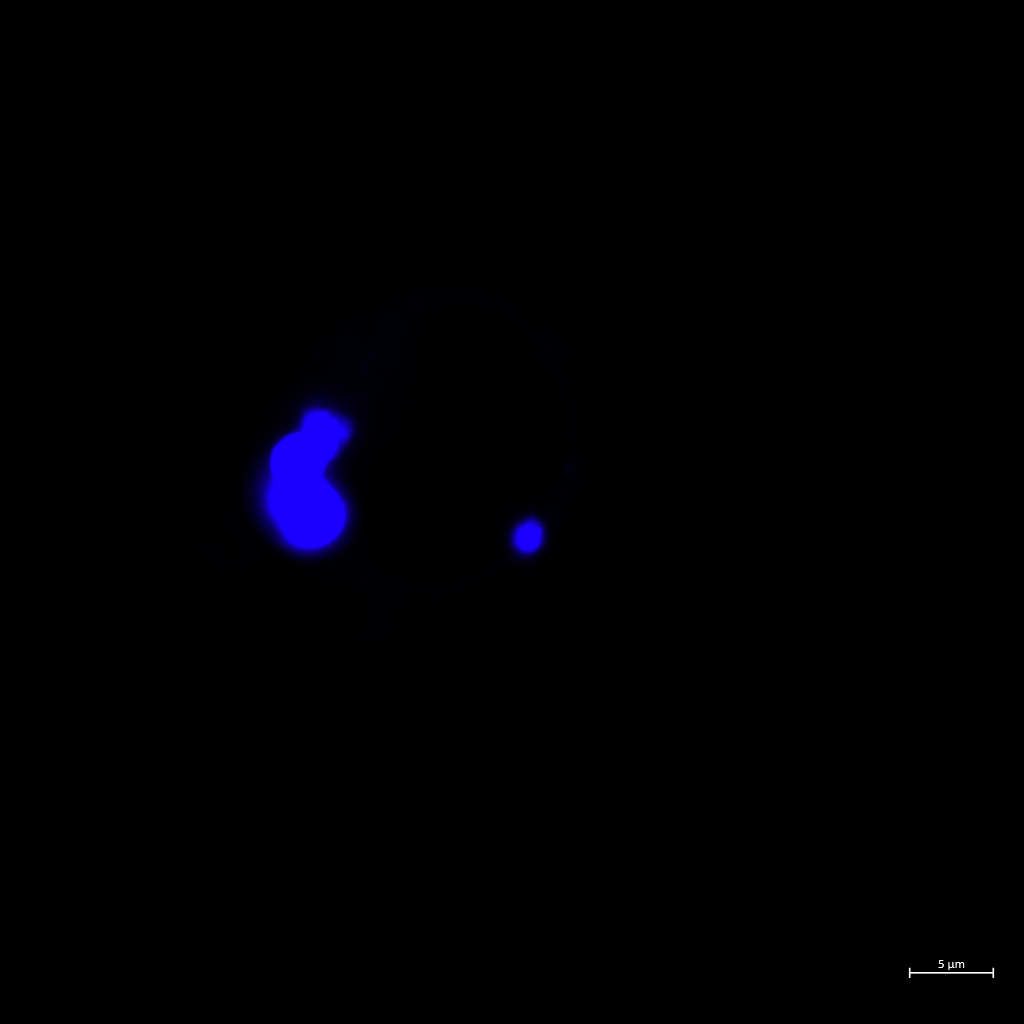

Supplement: Supplementary file 8 — Source data Fig. 5 [file 44319_2024_256_MOESM8_ESM.zip › SourceDateForFigure 5/5K/Snap-33R/Snap-1877-1-7_c3.tif]

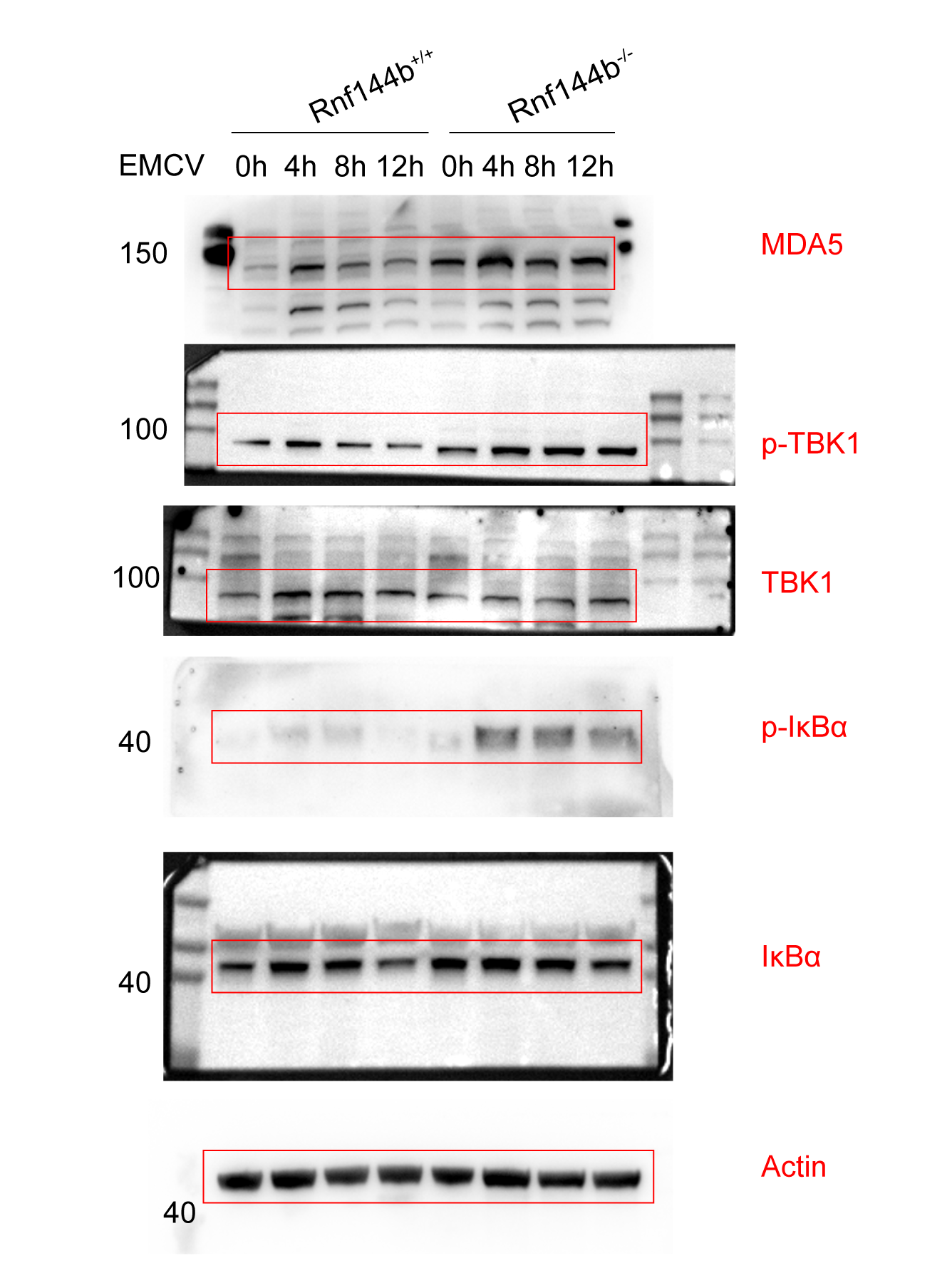

Supplement: Supplementary file 9 — Source data Fig. 6 [file 44319_2024_256_MOESM9_ESM.zip › SourceDateForFigure 6/6C.tif]

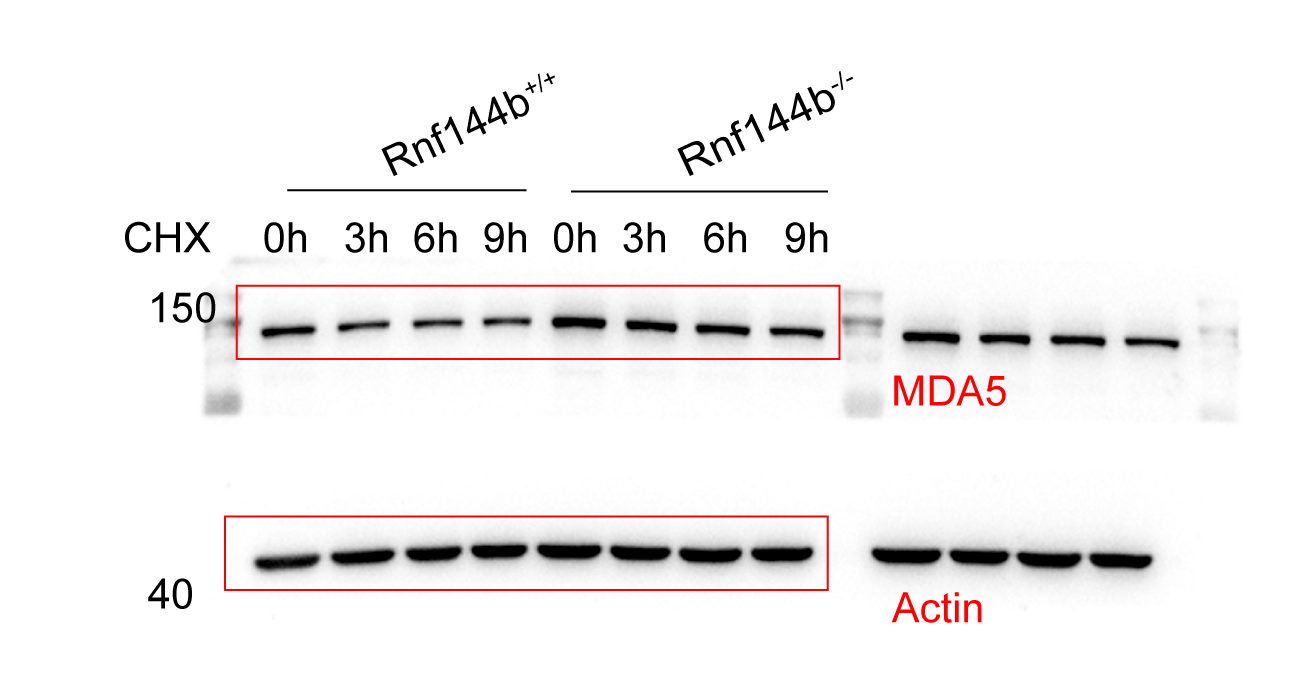

Supplement: Supplementary file 9 — Source data Fig. 6 [file 44319_2024_256_MOESM9_ESM.zip › SourceDateForFigure 6/6E.tif]

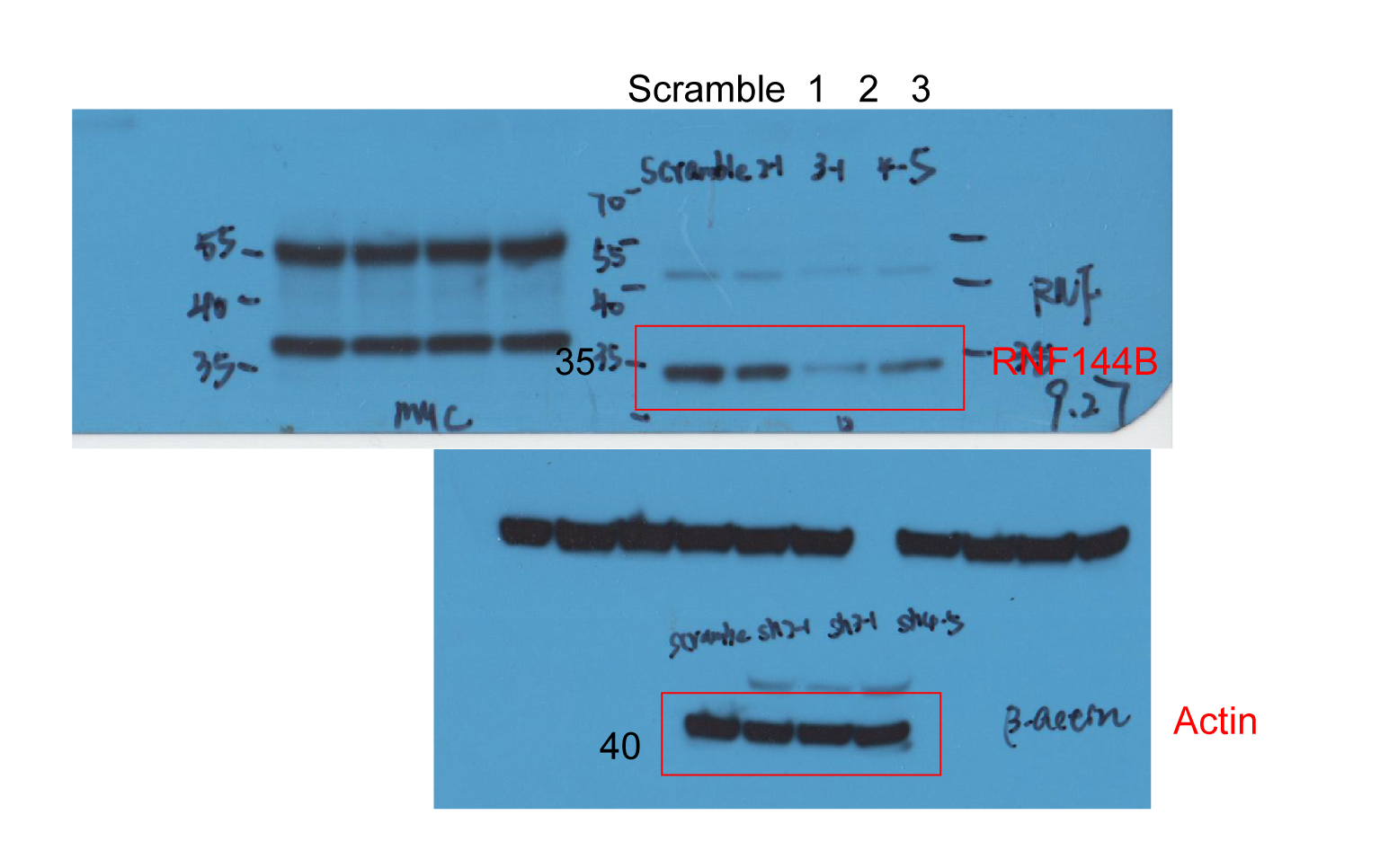

Supplement: Supplementary file 10 — Figure EV Source Data [file 44319_2024_256_MOESM10_ESM.zip › SourceDateForExpanded View/Figure EV1/1I.tif]

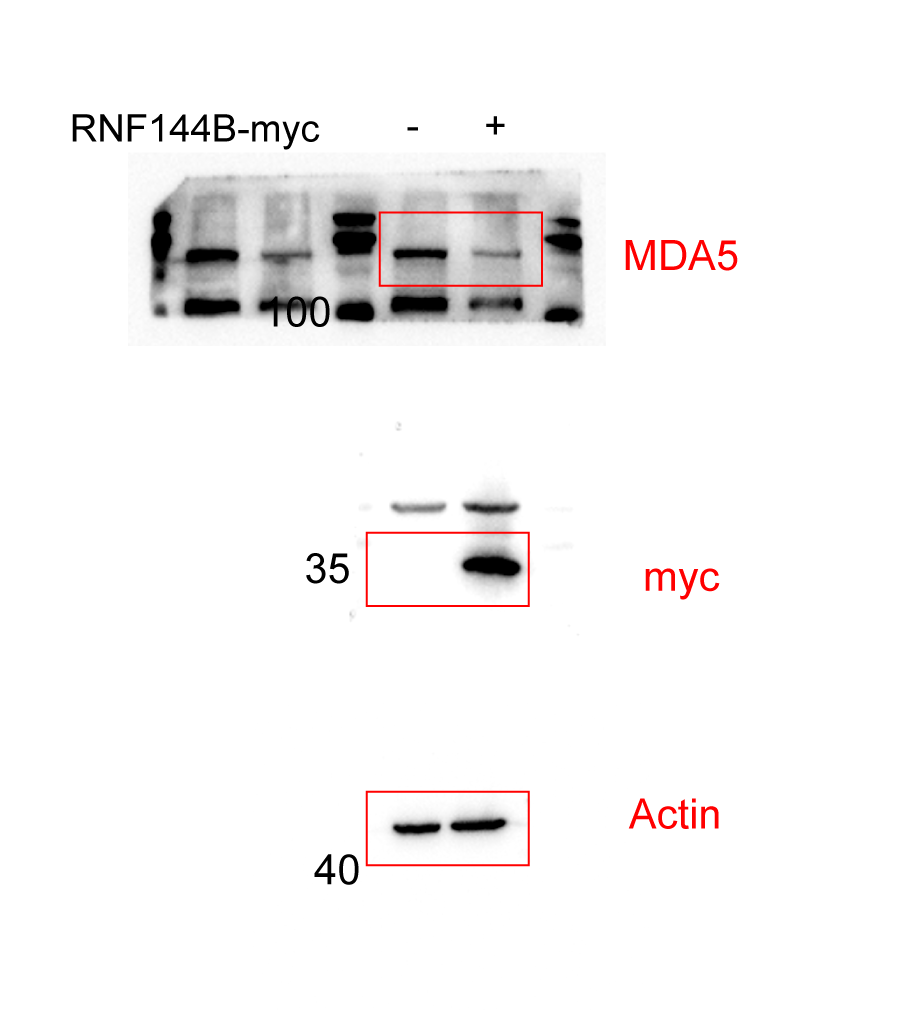

Supplement: Supplementary file 10 — Figure EV Source Data [file 44319_2024_256_MOESM10_ESM.zip › SourceDateForExpanded View/Figure EV2/2A.tif]

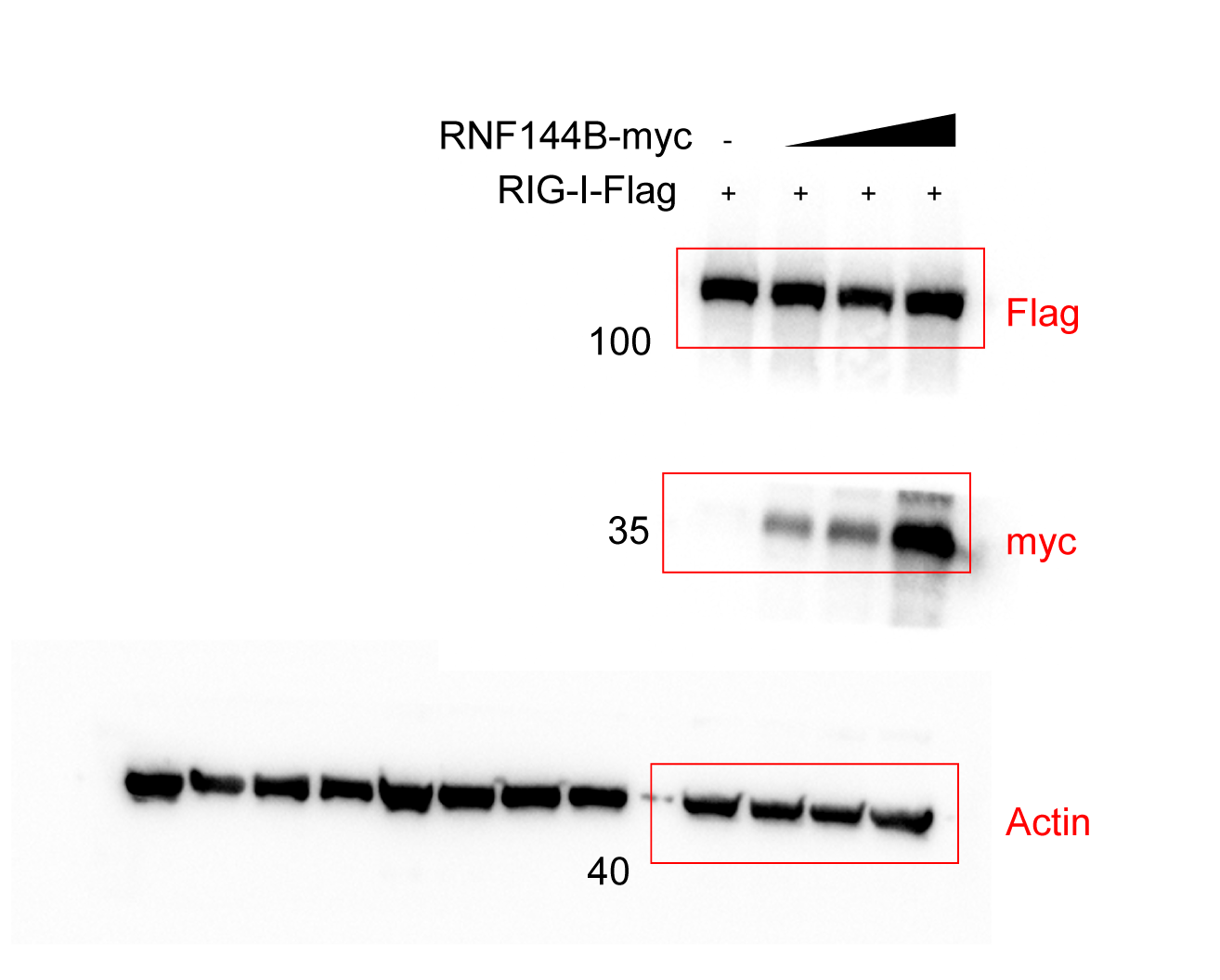

Supplement: Supplementary file 10 — Figure EV Source Data [file 44319_2024_256_MOESM10_ESM.zip › SourceDateForExpanded View/Figure EV2/2B.tif]

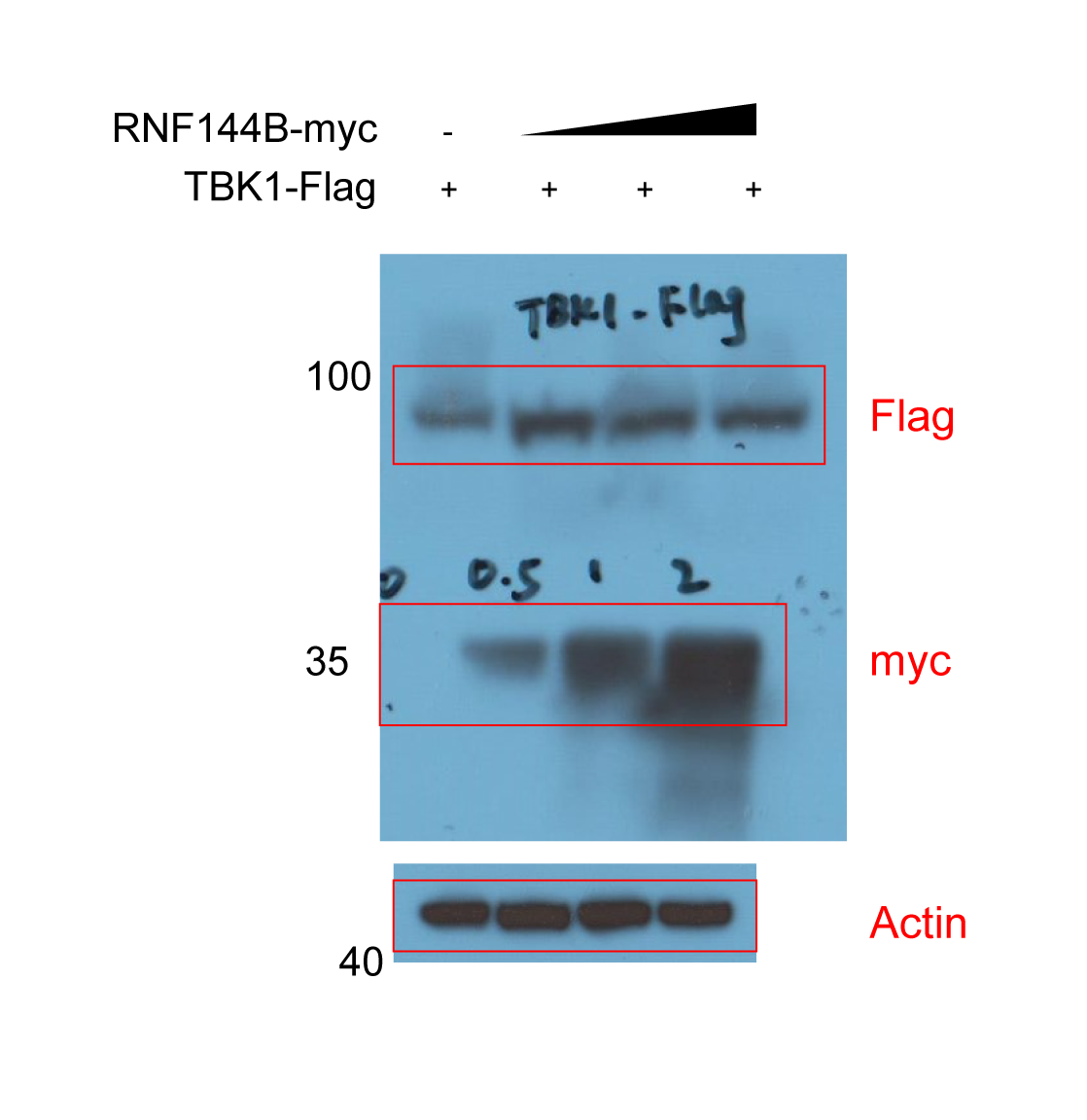

Supplement: Supplementary file 10 — Figure EV Source Data [file 44319_2024_256_MOESM10_ESM.zip › SourceDateForExpanded View/Figure EV2/2C.tif]

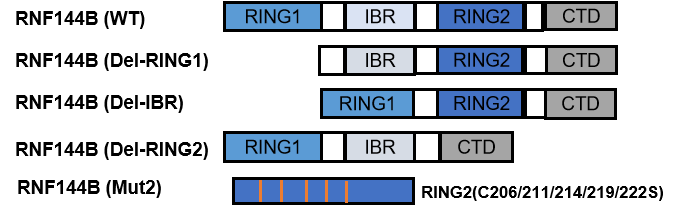

Supplement: Supplementary file 10 — Figure EV Source Data [file 44319_2024_256_MOESM10_ESM.zip › SourceDateForExpanded View/Figure EV2/2E.png]

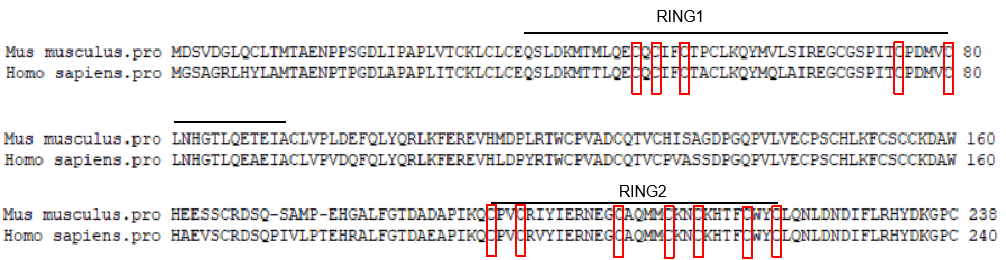

Supplement: Supplementary file 10 — Figure EV Source Data [file 44319_2024_256_MOESM10_ESM.zip › SourceDateForExpanded View/Figure EV2/2F.png]

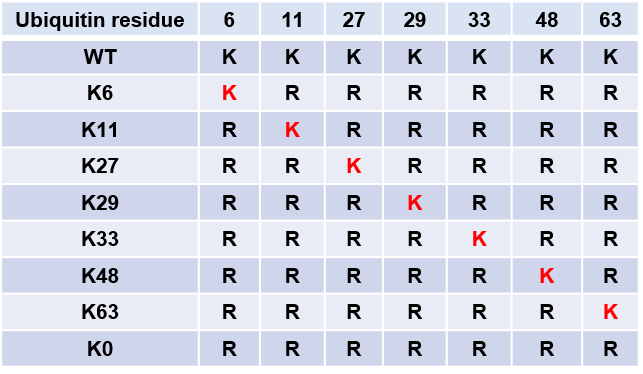

Supplement: Supplementary file 10 — Figure EV Source Data [file 44319_2024_256_MOESM10_ESM.zip › SourceDateForExpanded View/Figure EV2/2H(K-O).png]

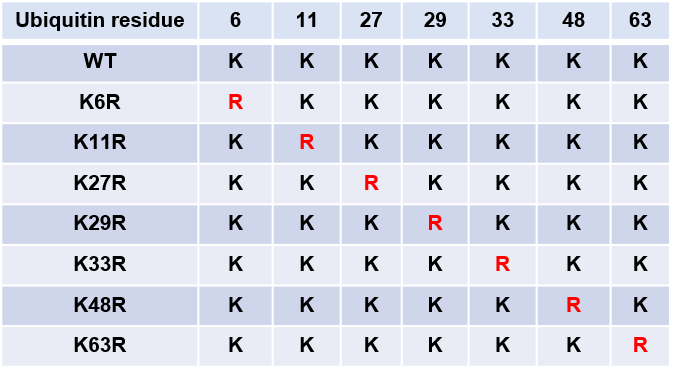

Supplement: Supplementary file 10 — Figure EV Source Data [file 44319_2024_256_MOESM10_ESM.zip › SourceDateForExpanded View/Figure EV2/2H(K-R).png]

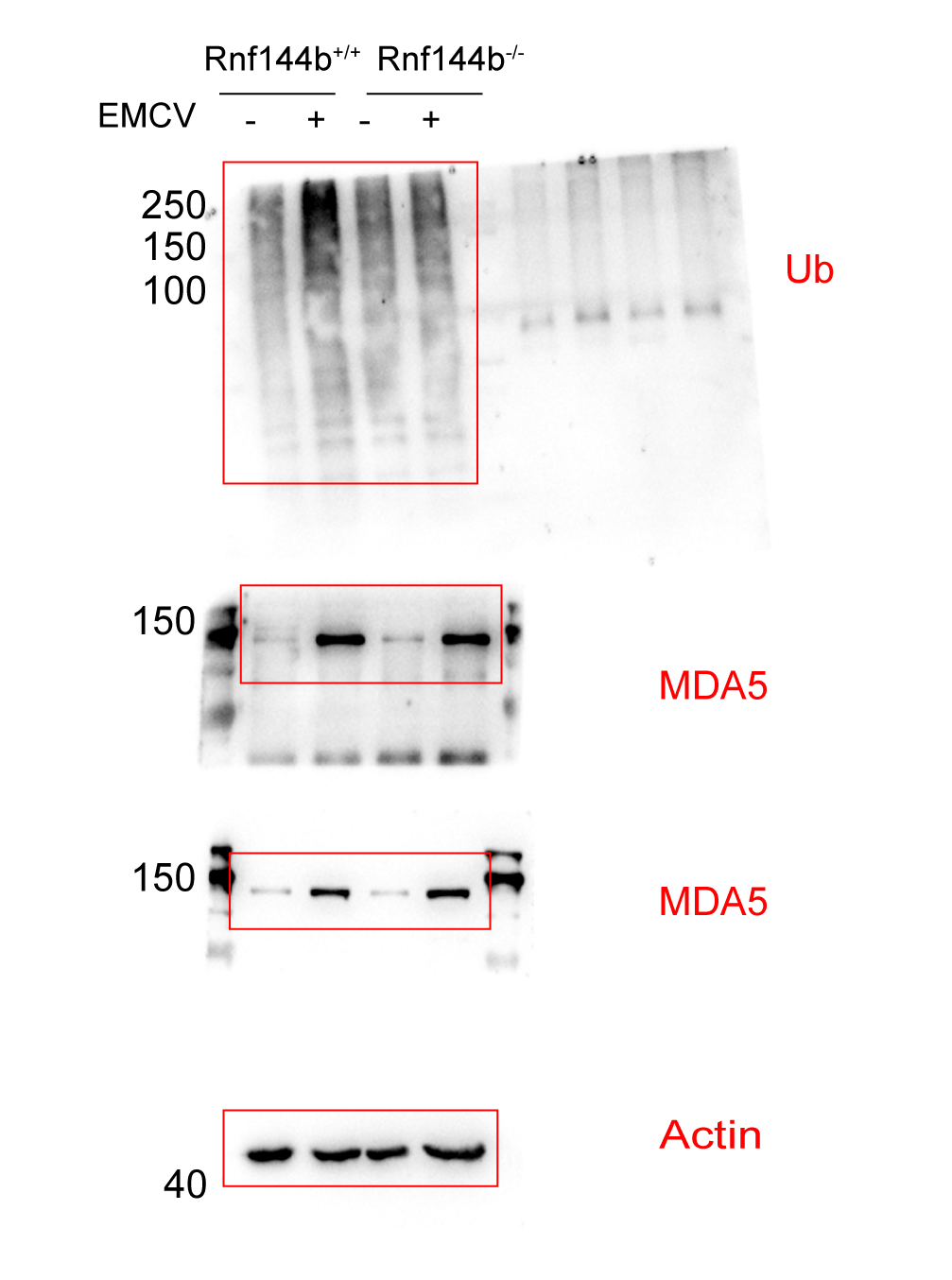

Supplement: Supplementary file 10 — Figure EV Source Data [file 44319_2024_256_MOESM10_ESM.zip › SourceDateForExpanded View/Figure EV2/2I.tif]

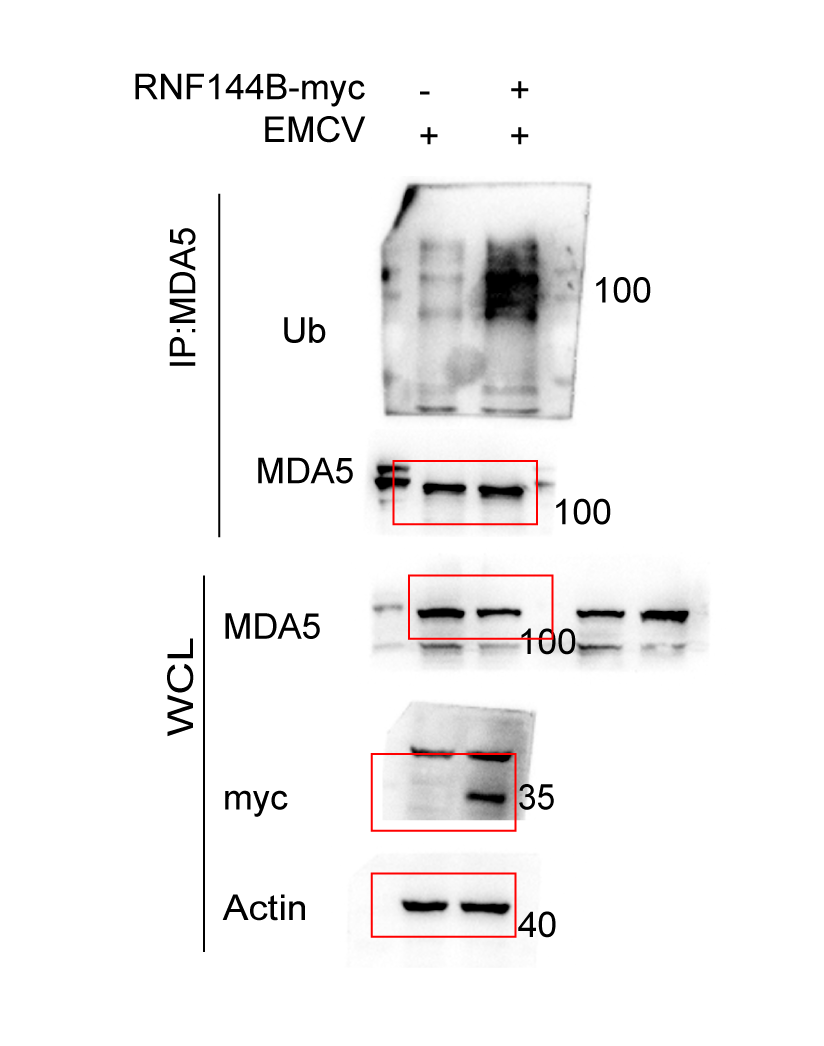

Supplement: Supplementary file 10 — Figure EV Source Data [file 44319_2024_256_MOESM10_ESM.zip › SourceDateForExpanded View/Figure EV2/2J.tif]
