# Supplementary figures and images for: RNF144B negatively regulates antiviral immunity by targeting MDA5 for autophagic degradation (part 2 of 2)
Source: EMBO Rep. 2024 Sep 16;25(10):4594–624. doi: 10.1038/s44319-024-00256-w (PMC11467429; doi:10.1038/s44319-024-00256-w)

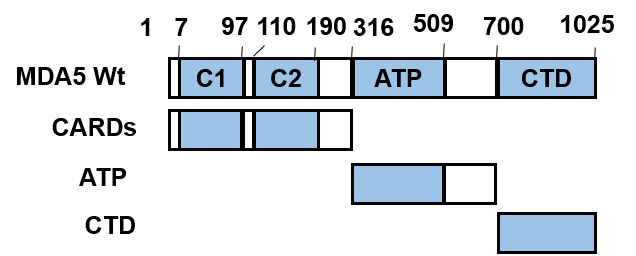

Supplement: Supplementary file 10 — Figure EV Source Data [file 44319_2024_256_MOESM10_ESM.zip › SourceDateForExpanded View/Figure EV2/2K.png]

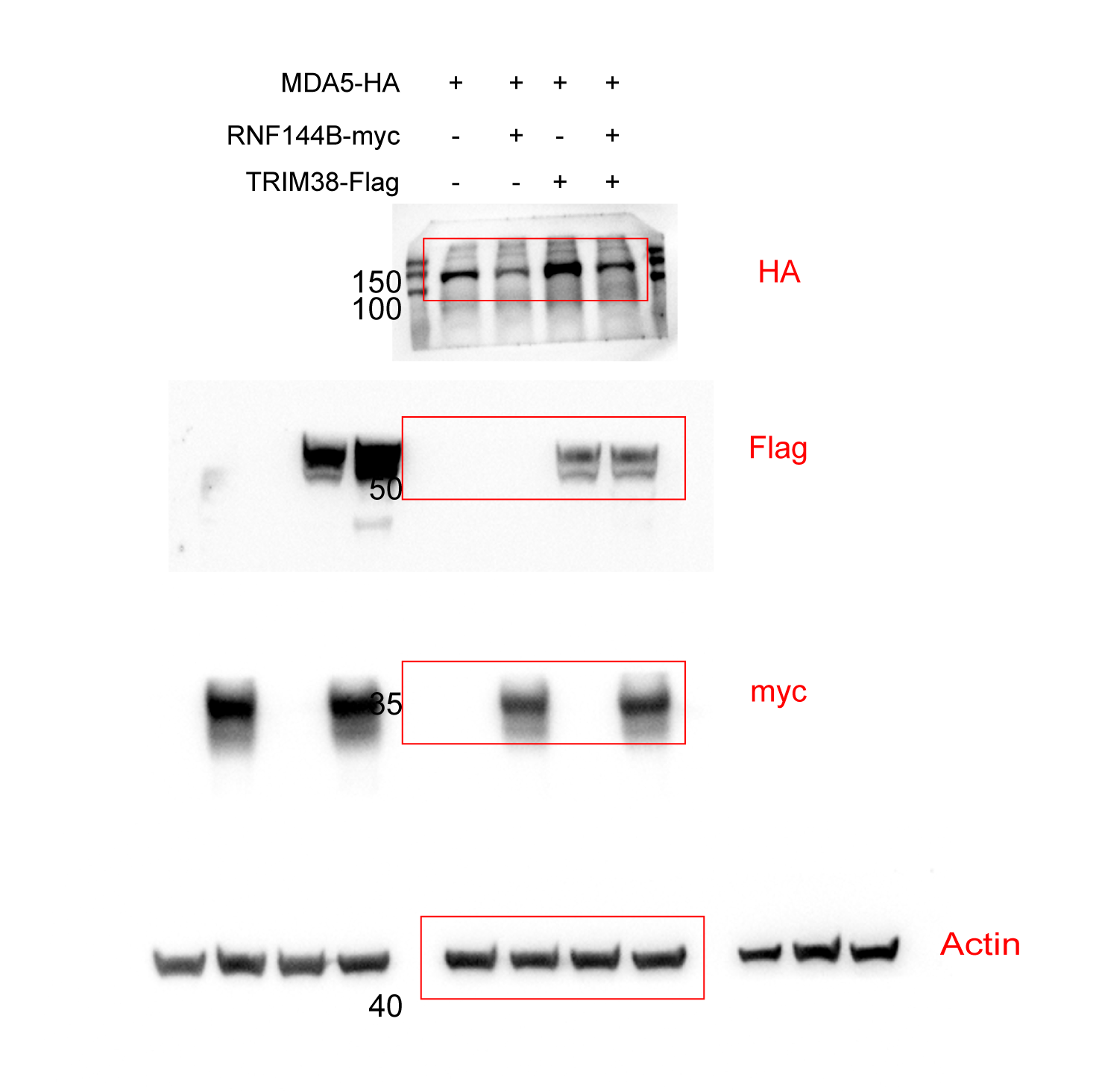

Supplement: Supplementary file 10 — Figure EV Source Data [file 44319_2024_256_MOESM10_ESM.zip › SourceDateForExpanded View/Figure EV2/2L.tif]

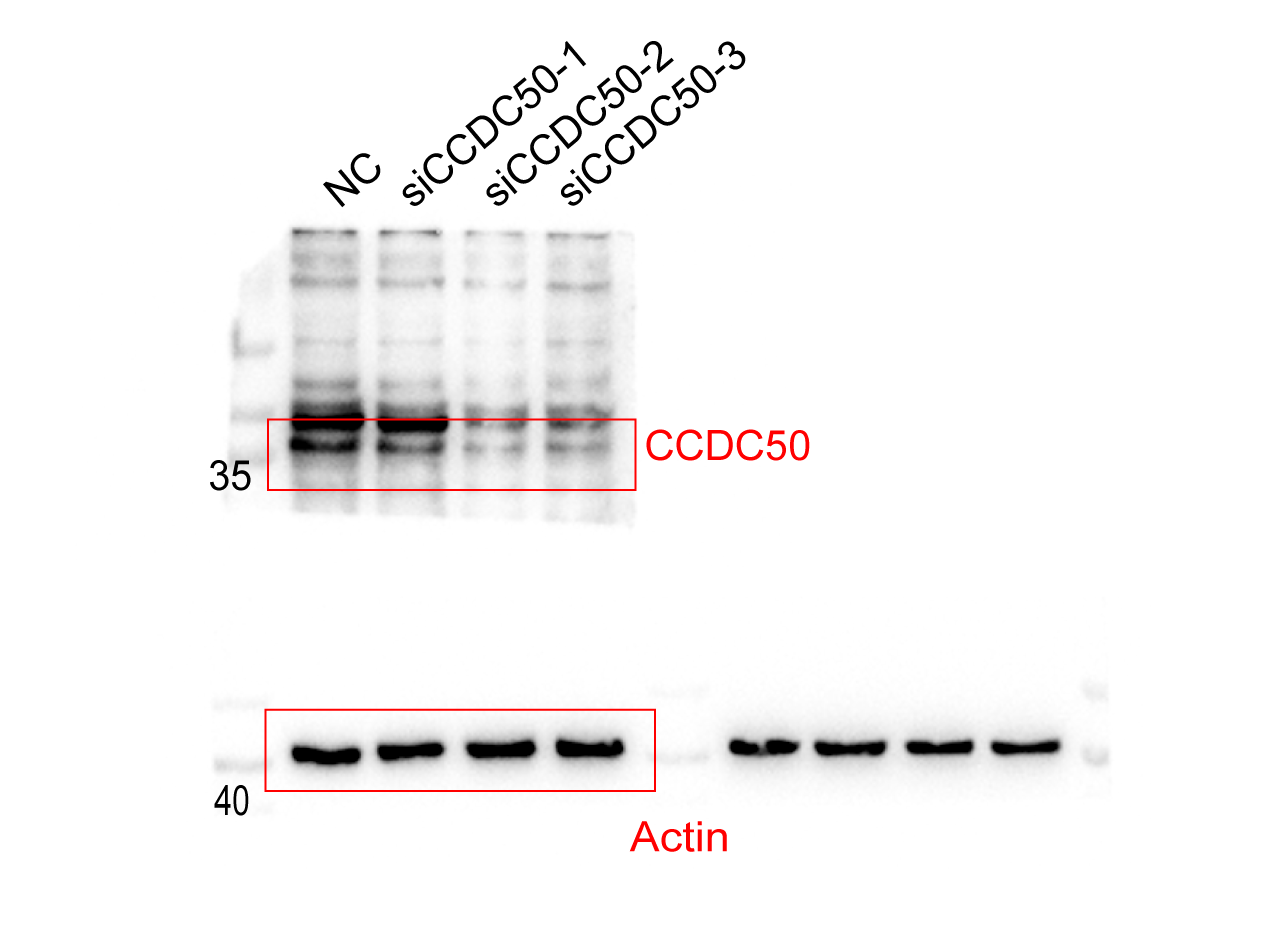

Supplement: Supplementary file 10 — Figure EV Source Data [file 44319_2024_256_MOESM10_ESM.zip › SourceDateForExpanded View/Figure EV3/3A.tif]

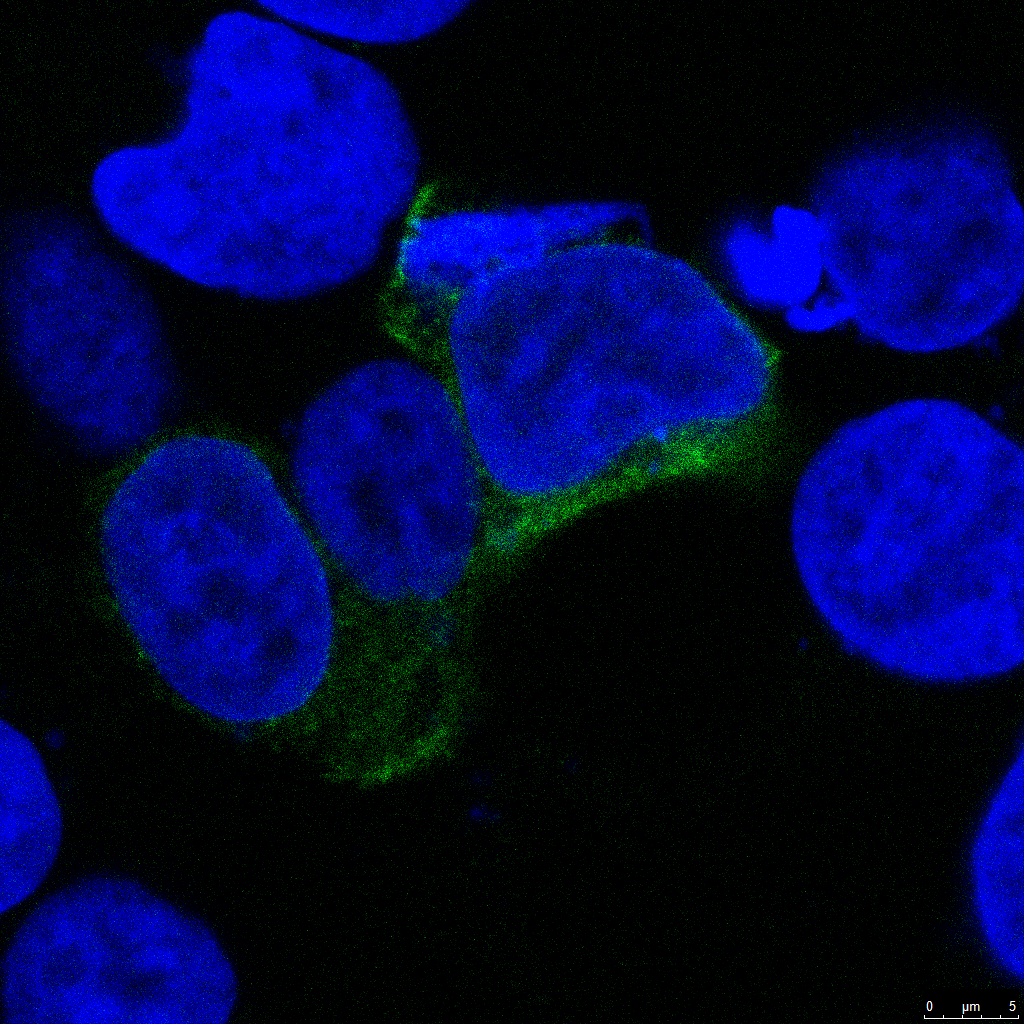

Supplement: Supplementary file 10 — Figure EV Source Data [file 44319_2024_256_MOESM10_ESM.zip › SourceDateForExpanded View/Figure EV3/3B/√-Experiment_HA-vector-MDA5-3-supplementFig3/Experiment_HA-MDA5-3_z0.tif]

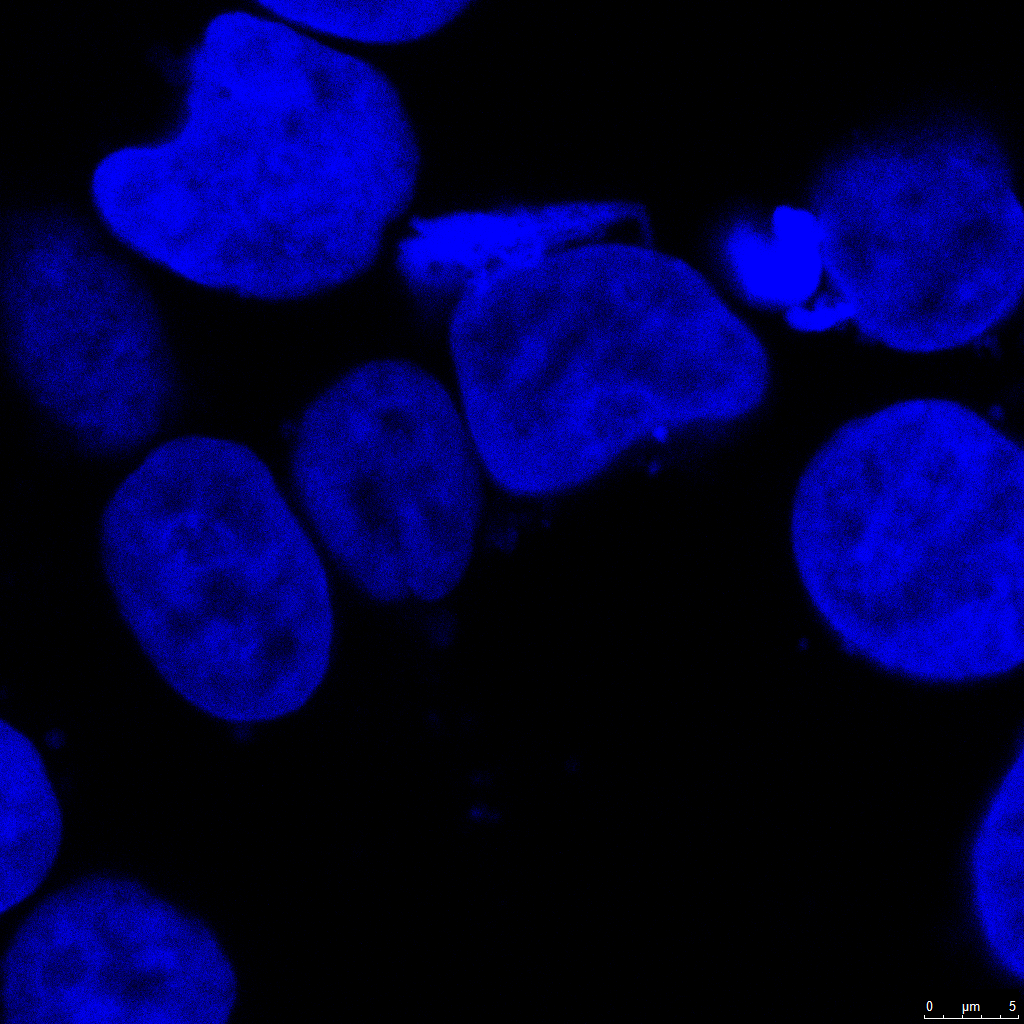

Supplement: Supplementary file 10 — Figure EV Source Data [file 44319_2024_256_MOESM10_ESM.zip › SourceDateForExpanded View/Figure EV3/3B/√-Experiment_HA-vector-MDA5-3-supplementFig3/Experiment_HA-MDA5-3_z0_ch00.tif]

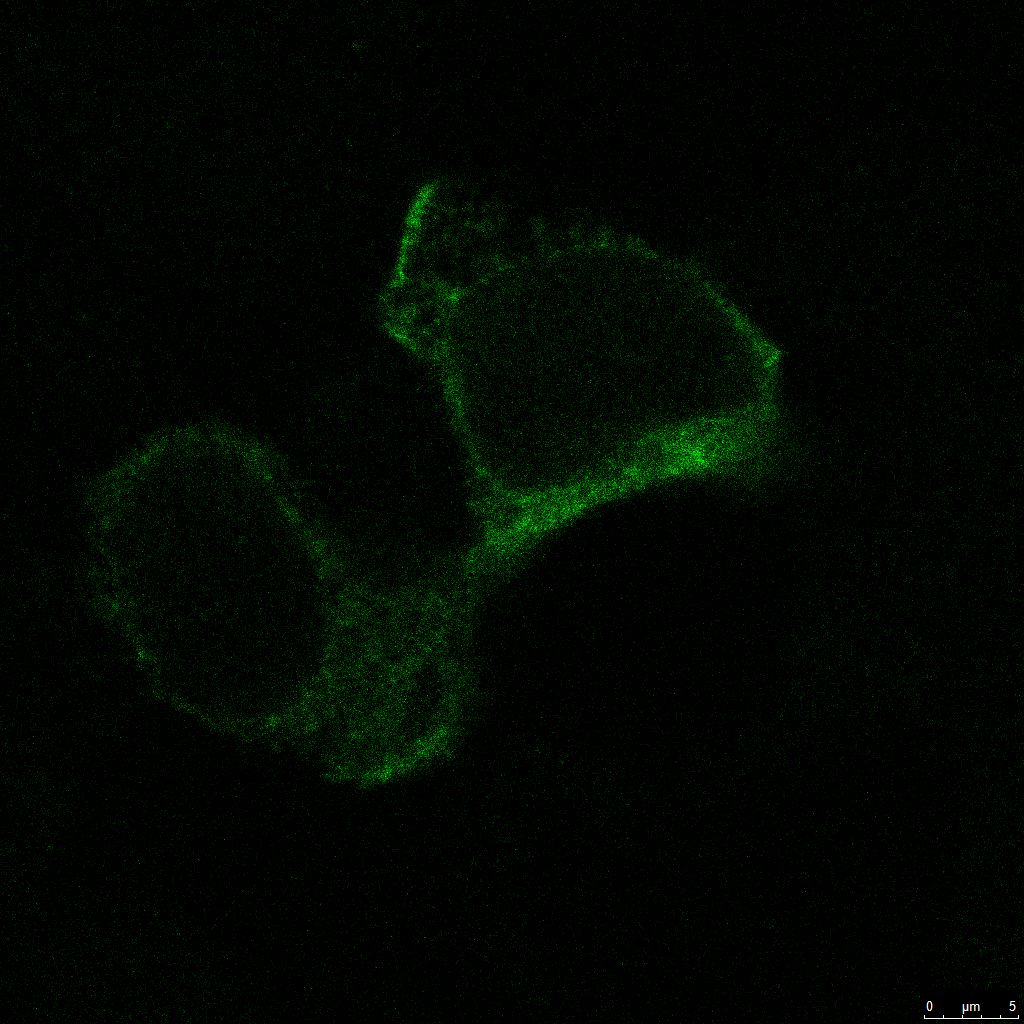

Supplement: Supplementary file 10 — Figure EV Source Data [file 44319_2024_256_MOESM10_ESM.zip › SourceDateForExpanded View/Figure EV3/3B/√-Experiment_HA-vector-MDA5-3-supplementFig3/Experiment_HA-MDA5-3_z0_ch01.tif]

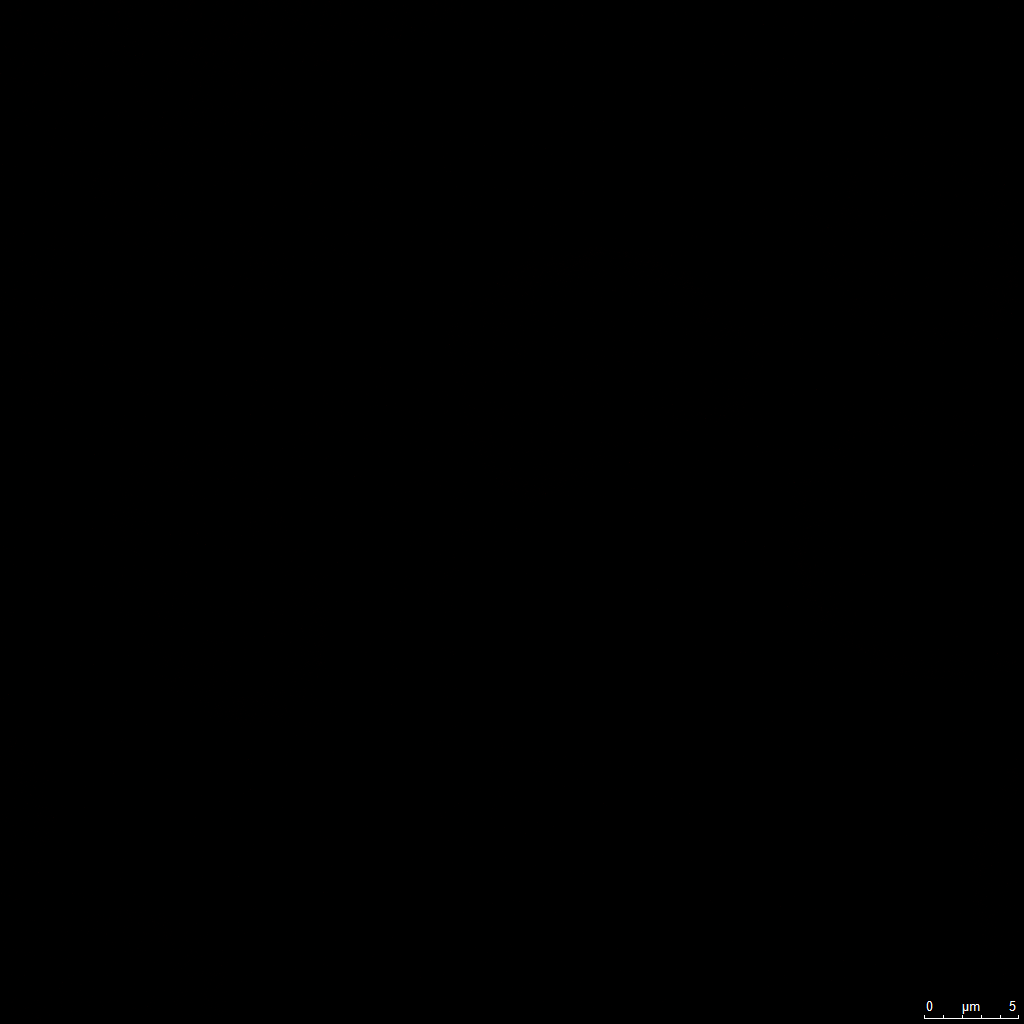

Supplement: Supplementary file 10 — Figure EV Source Data [file 44319_2024_256_MOESM10_ESM.zip › SourceDateForExpanded View/Figure EV3/3B/√-Experiment_HA-vector-MDA5-3-supplementFig3/Experiment_HA-MDA5-3_z0_ch02.tif]

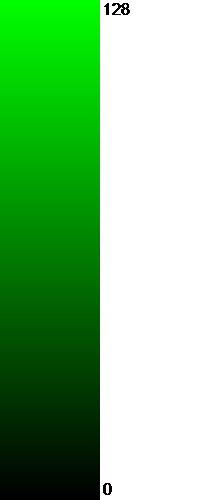

Supplement: Supplementary file 10 — Figure EV Source Data [file 44319_2024_256_MOESM10_ESM.zip › SourceDateForExpanded View/Figure EV3/3B/√-Experiment_HA-vector-MDA5-3-supplementFig3/MetaData/Experiment_HA-MDA5-3ch1LUT.png]

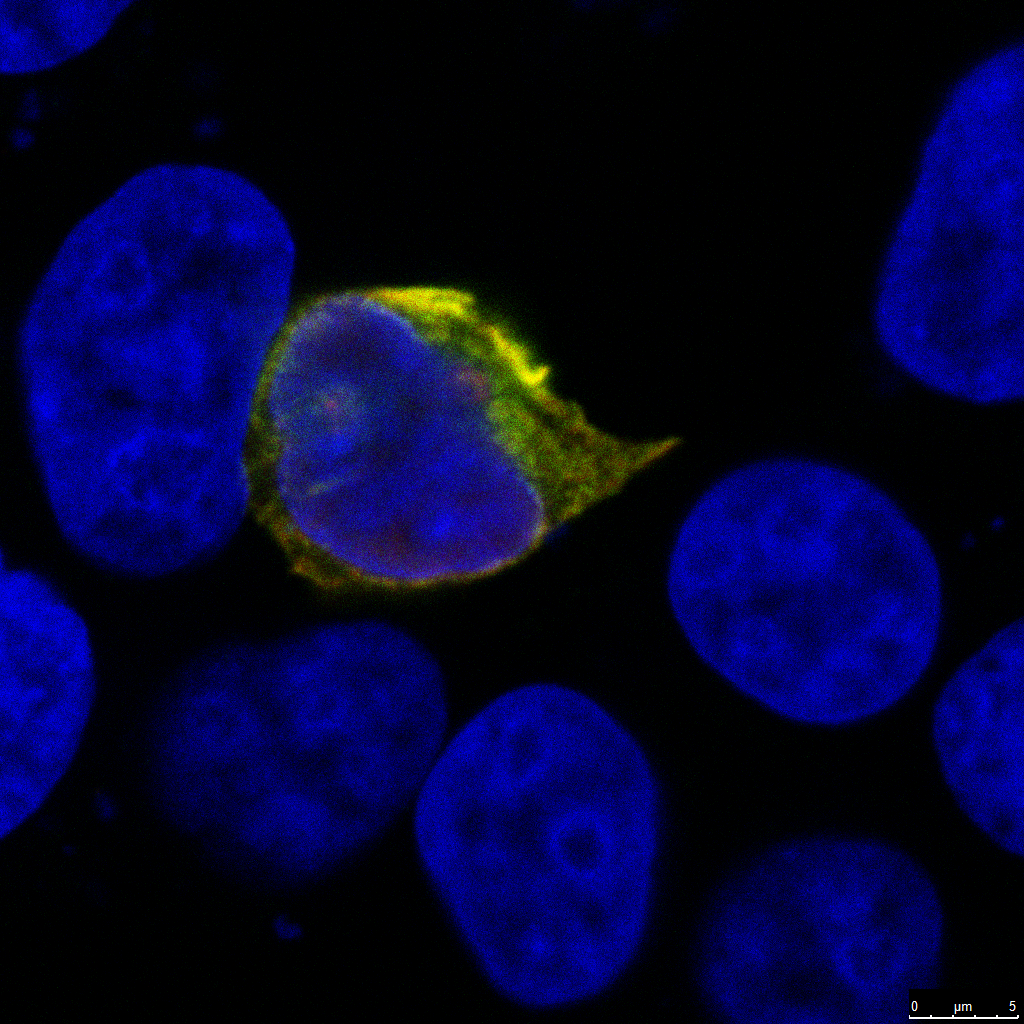

Supplement: Supplementary file 10 — Figure EV Source Data [file 44319_2024_256_MOESM10_ESM.zip › SourceDateForExpanded View/Figure EV3/3B/√Experiment_CCDC50-MDA5-4-supplementFig3/Experiment_CC-MDA5-4_z0.tif]

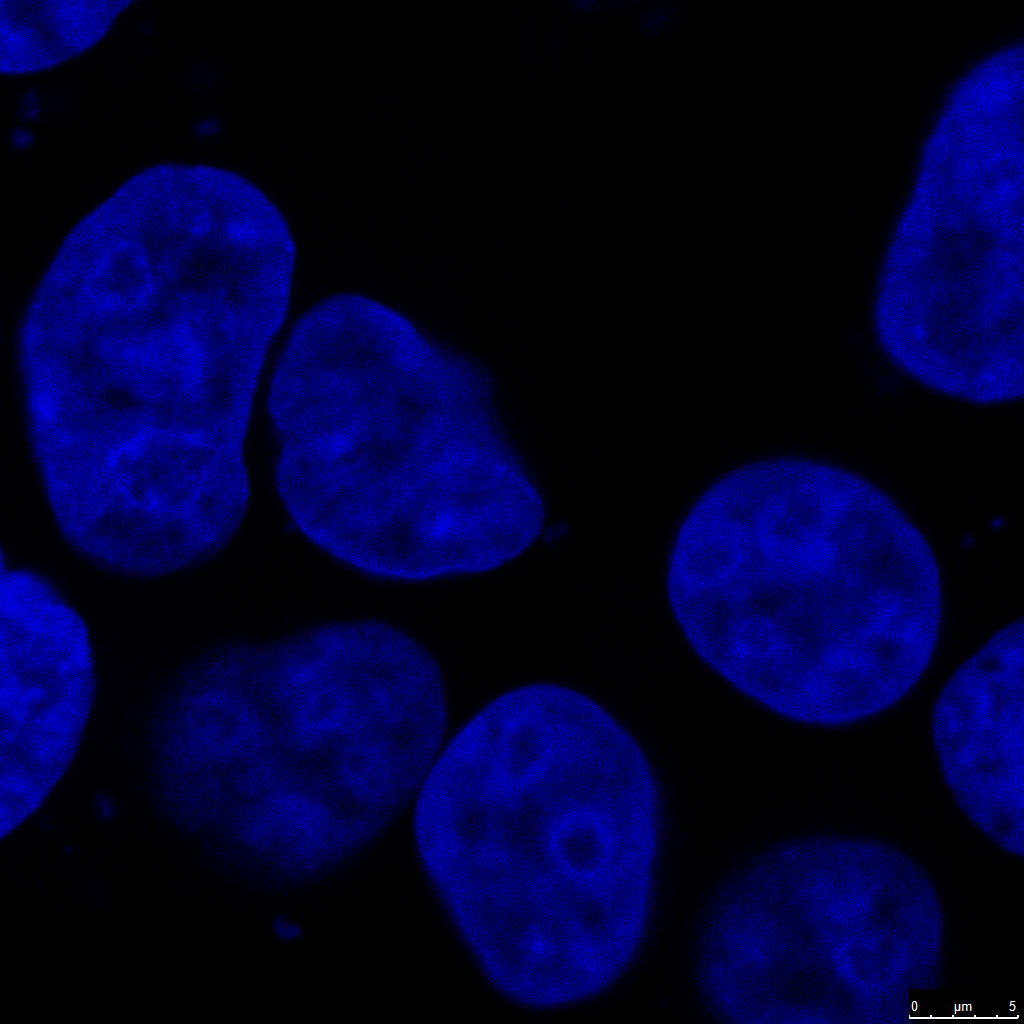

Supplement: Supplementary file 10 — Figure EV Source Data [file 44319_2024_256_MOESM10_ESM.zip › SourceDateForExpanded View/Figure EV3/3B/√Experiment_CCDC50-MDA5-4-supplementFig3/Experiment_CC-MDA5-4_z0_ch00.tif]

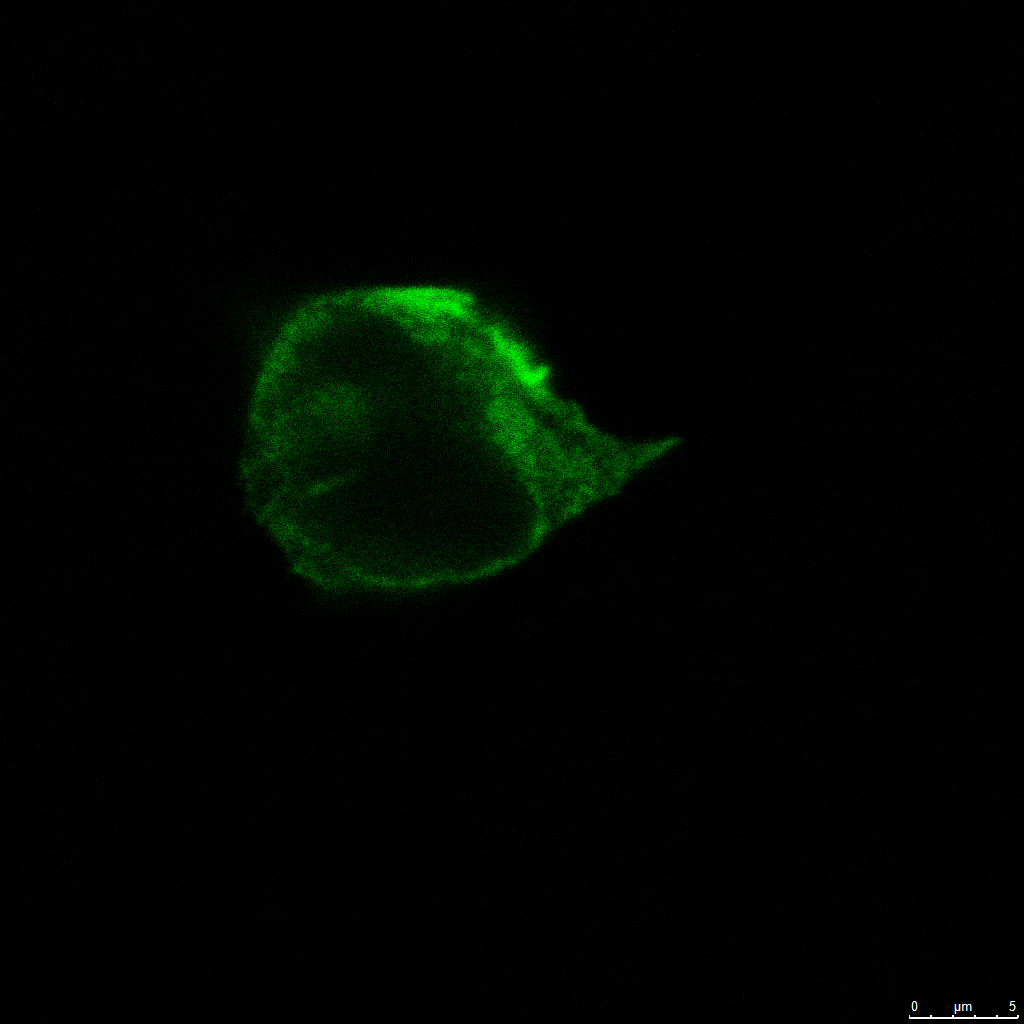

Supplement: Supplementary file 10 — Figure EV Source Data [file 44319_2024_256_MOESM10_ESM.zip › SourceDateForExpanded View/Figure EV3/3B/√Experiment_CCDC50-MDA5-4-supplementFig3/Experiment_CC-MDA5-4_z0_ch01.tif]

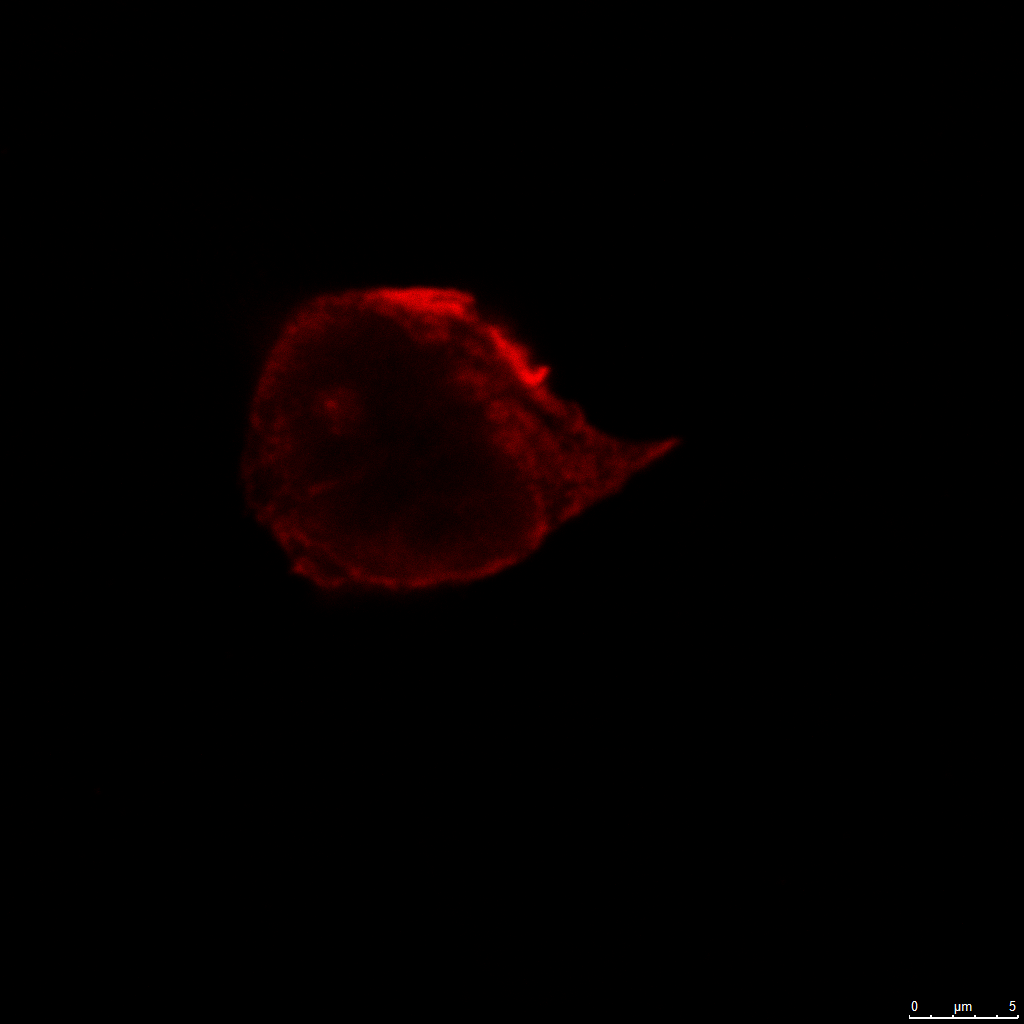

Supplement: Supplementary file 10 — Figure EV Source Data [file 44319_2024_256_MOESM10_ESM.zip › SourceDateForExpanded View/Figure EV3/3B/√Experiment_CCDC50-MDA5-4-supplementFig3/Experiment_CC-MDA5-4_z0_ch02.tif]

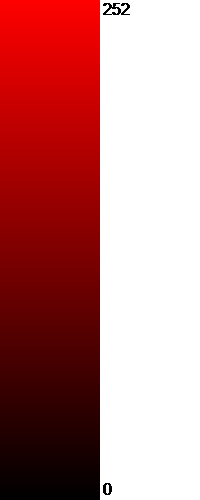

Supplement: Supplementary file 10 — Figure EV Source Data [file 44319_2024_256_MOESM10_ESM.zip › SourceDateForExpanded View/Figure EV3/3B/√Experiment_CCDC50-MDA5-4-supplementFig3/MetaData/Experiment_CC-MDA5-4ch2LUT.png]

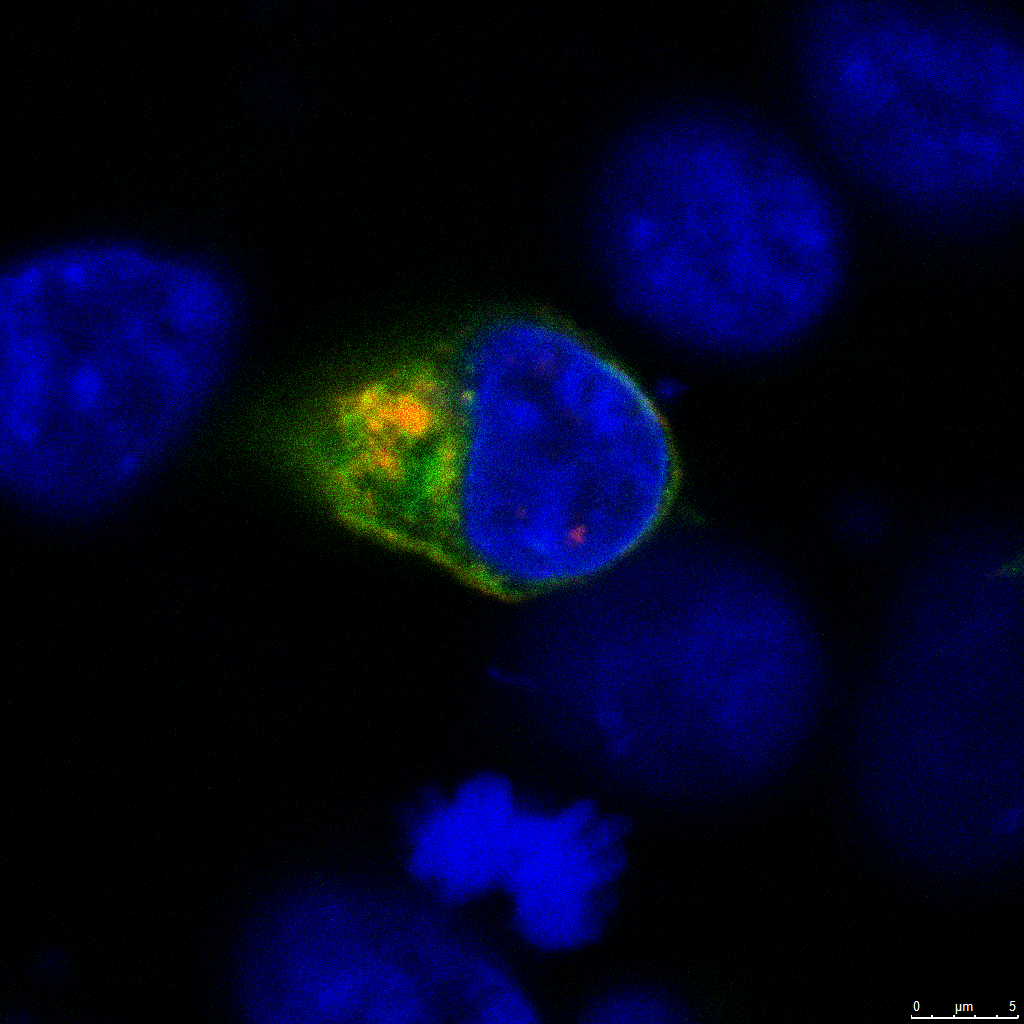

Supplement: Supplementary file 10 — Figure EV Source Data [file 44319_2024_256_MOESM10_ESM.zip › SourceDateForExpanded View/Figure EV3/3B/√Experiment_tollip-MDA5-9-supplementFig3/Experiment_TO-MDA5-9_z0.tif]

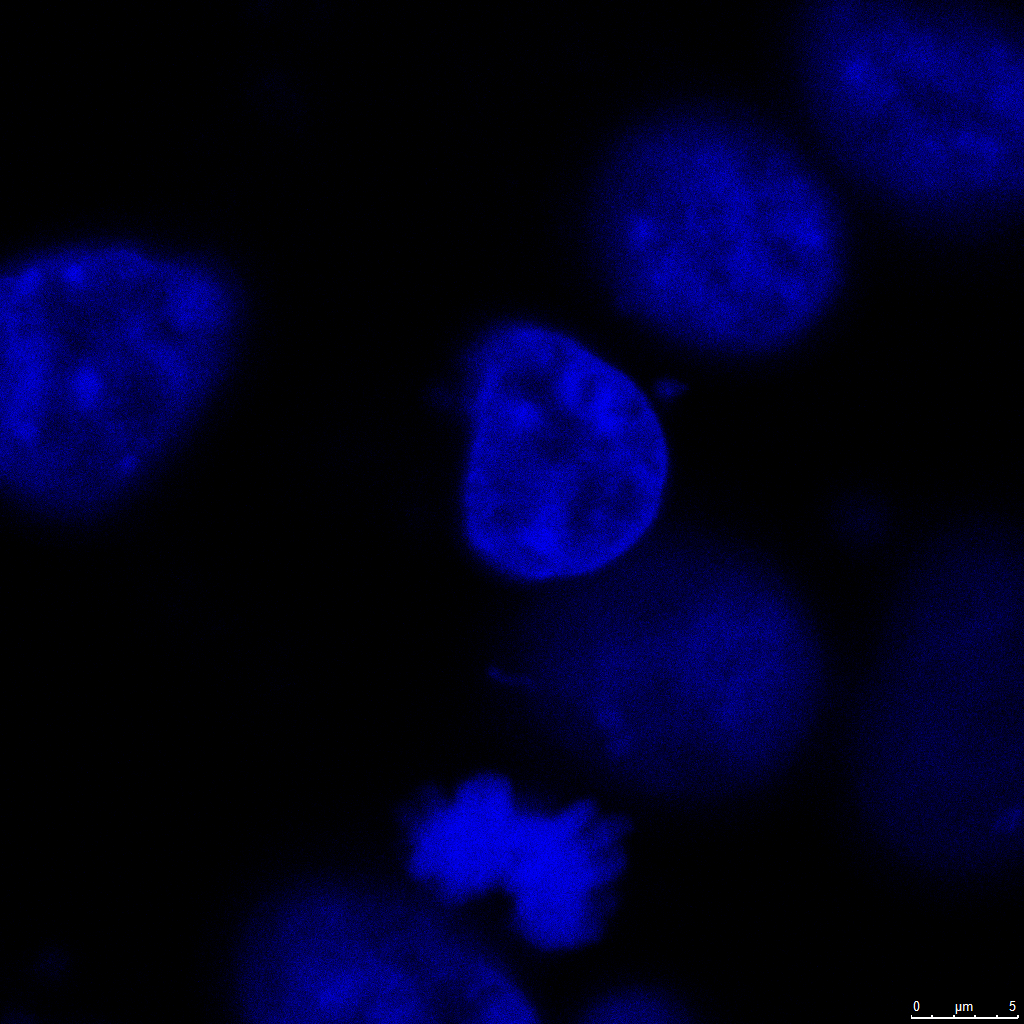

Supplement: Supplementary file 10 — Figure EV Source Data [file 44319_2024_256_MOESM10_ESM.zip › SourceDateForExpanded View/Figure EV3/3B/√Experiment_tollip-MDA5-9-supplementFig3/Experiment_TO-MDA5-9_z0_ch00.tif]

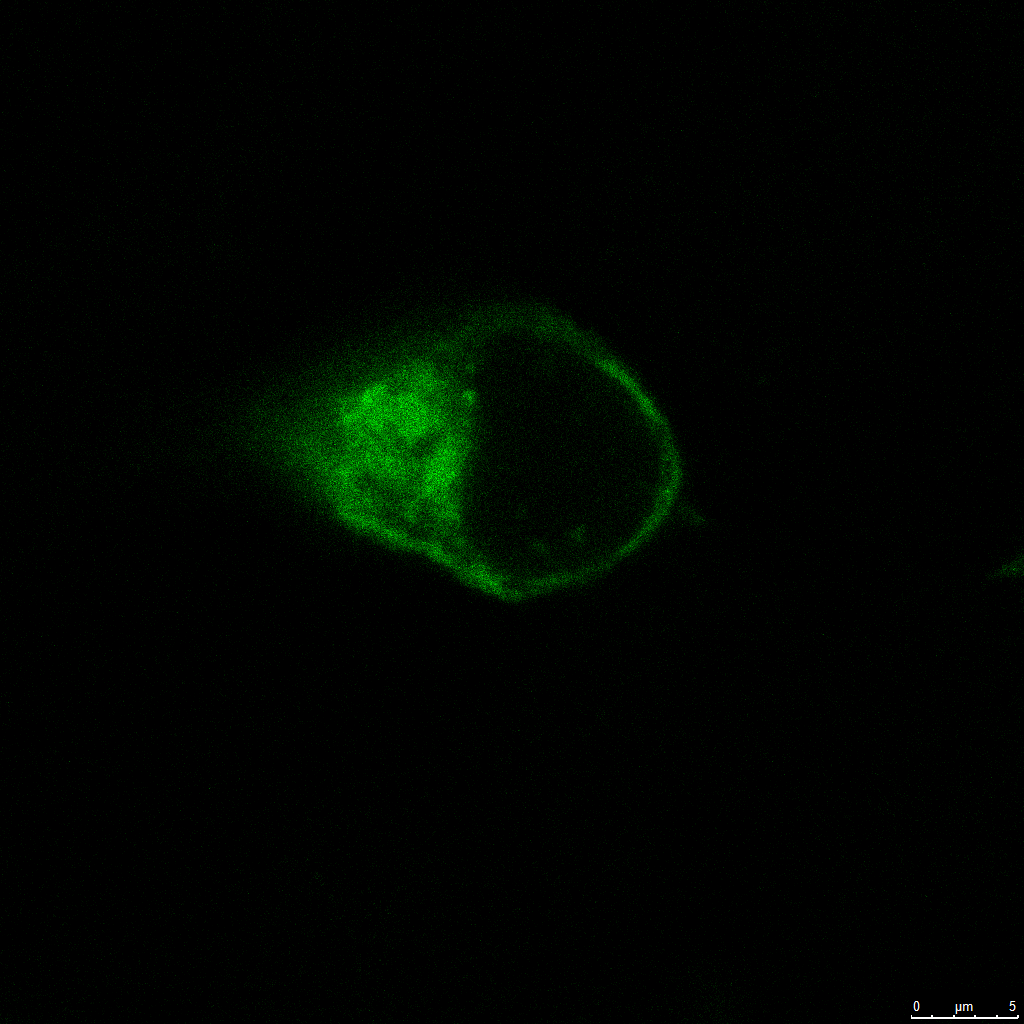

Supplement: Supplementary file 10 — Figure EV Source Data [file 44319_2024_256_MOESM10_ESM.zip › SourceDateForExpanded View/Figure EV3/3B/√Experiment_tollip-MDA5-9-supplementFig3/Experiment_TO-MDA5-9_z0_ch01.tif]

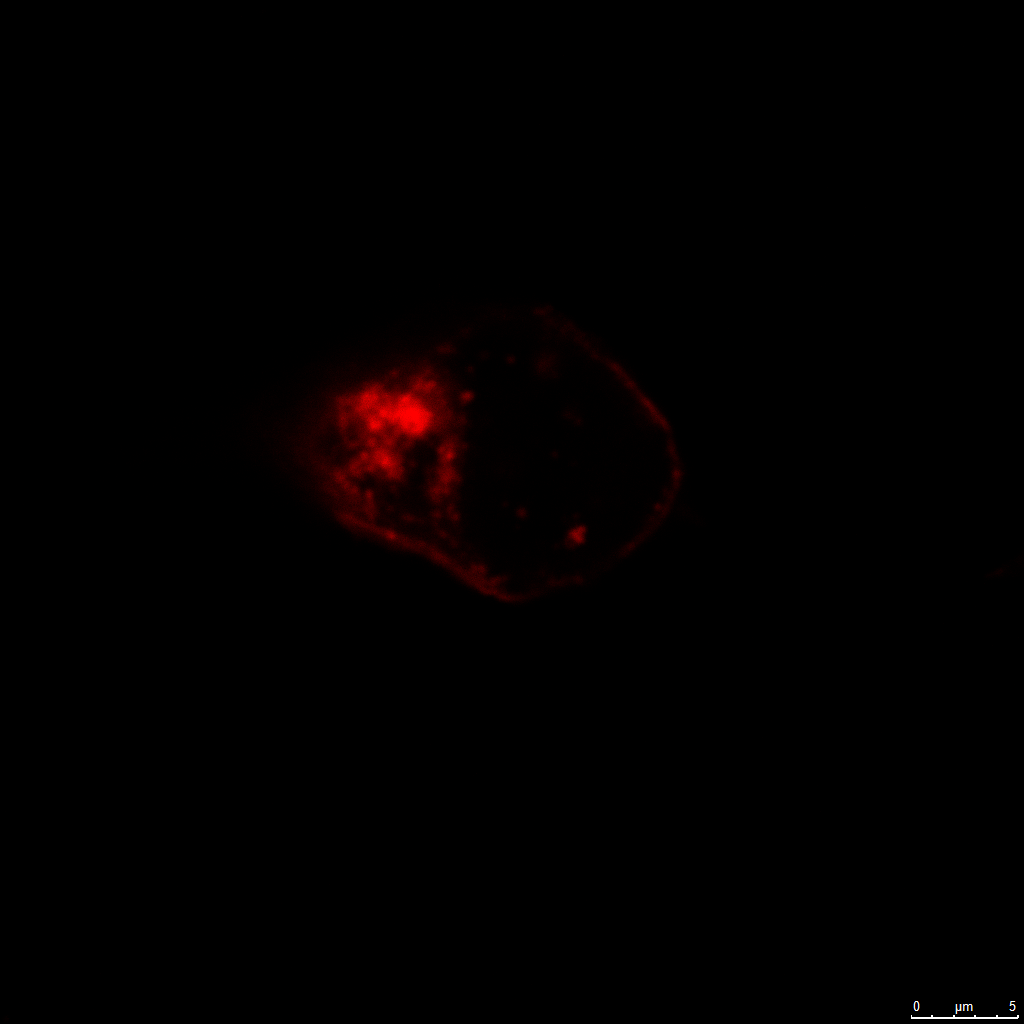

Supplement: Supplementary file 10 — Figure EV Source Data [file 44319_2024_256_MOESM10_ESM.zip › SourceDateForExpanded View/Figure EV3/3B/√Experiment_tollip-MDA5-9-supplementFig3/Experiment_TO-MDA5-9_z0_ch02.tif]

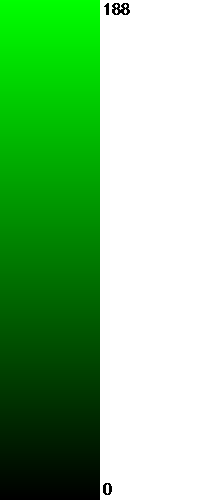

Supplement: Supplementary file 10 — Figure EV Source Data [file 44319_2024_256_MOESM10_ESM.zip › SourceDateForExpanded View/Figure EV3/3B/√Experiment_tollip-MDA5-9-supplementFig3/MetaData/Experiment_TO-MDA5-9ch1LUT.png]

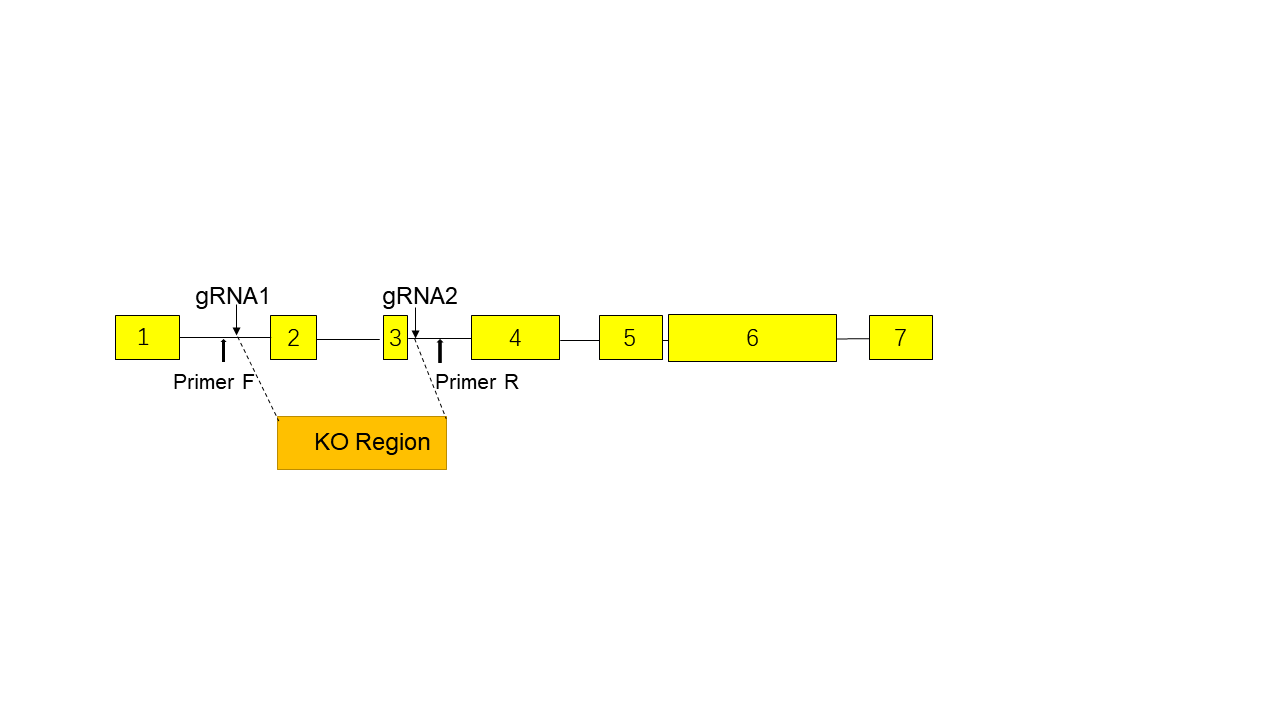

Supplement: Supplementary file 10 — Figure EV Source Data [file 44319_2024_256_MOESM10_ESM.zip › SourceDateForExpanded View/Figure EV3/3C.tif]

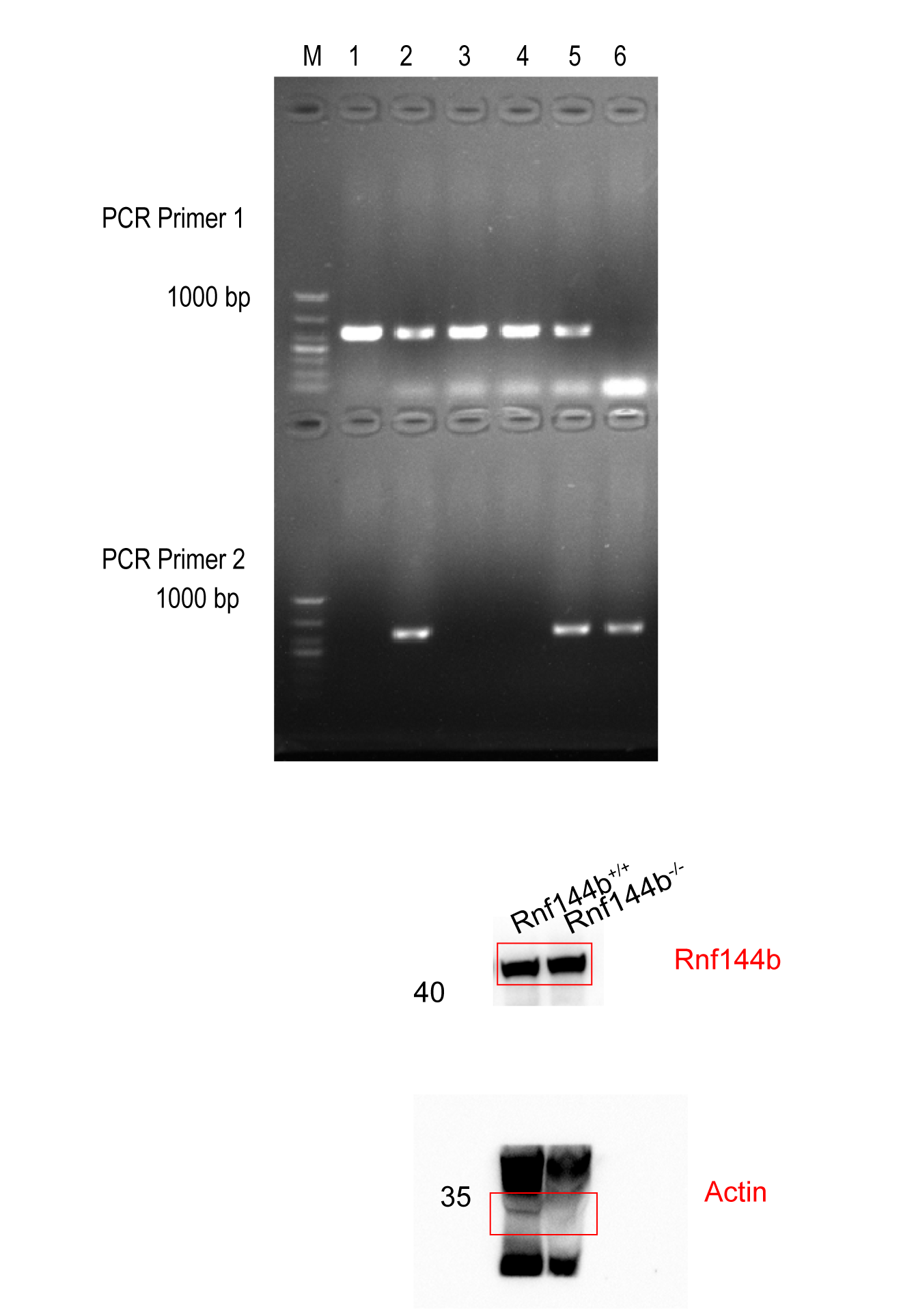

Supplement: Supplementary file 10 — Figure EV Source Data [file 44319_2024_256_MOESM10_ESM.zip › SourceDateForExpanded View/Figure EV3/3D.tif]

# BD FACSDiva 8.0.2

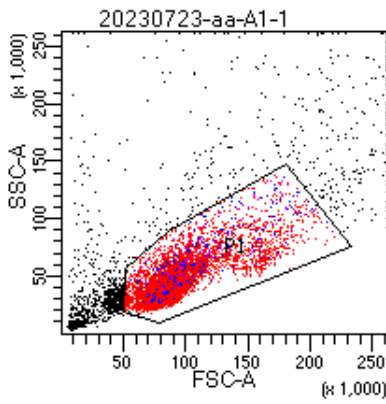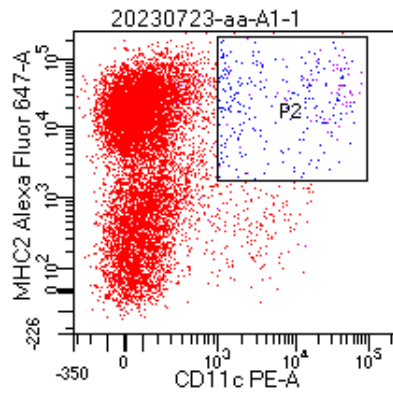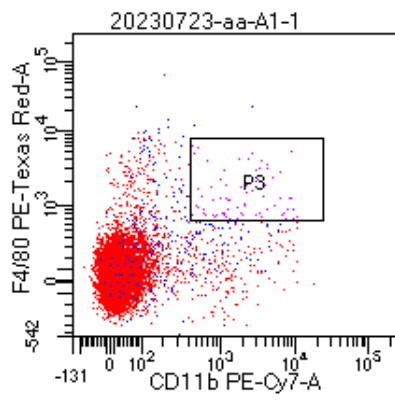

| Tube: aa-A1-1 |         |         |        |  |
|---------------|---------|---------|--------|--|
| Population    | #Events | %Parent | %Total |  |
| All Events    | 12,130  | ####    | 100.0  |  |
| P1            | 10,757  | 88.7    | 88.7   |  |
| P2            | 285     | 2.6     | 2.3    |  |
| P3            | 73      | 0.7     | 0.6    |  |

BD FACSDiva 8.0.2

BD FACSDiva 8.0.2

Supplement: Supplementary file 10 — Figure EV Source Data [file 44319_2024_256_MOESM10_ESM.zip › SourceDateForExpanded View/Figure EV3/3E/DC,2f,Mo_20230723_aa-A1-1_23072023204853.pdf]

# BD FACSDiva 8.0.2

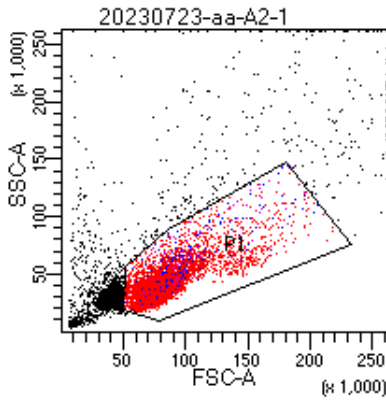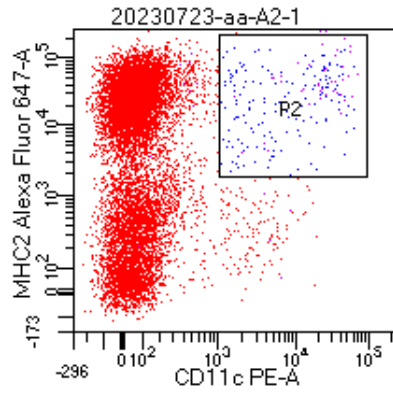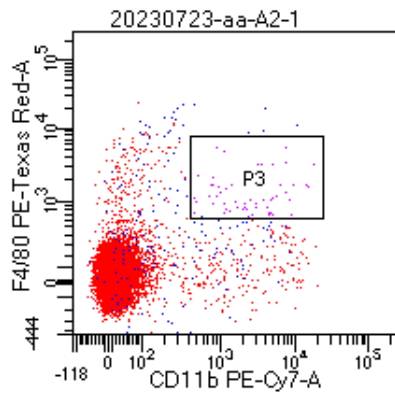

| Tube: aa-A2-1 |         |         |        |
|---------------|---------|---------|--------|
| Population    | #Events | %Parent | %Total |
| All Events    | 12,222  | ####    | 100.0  |
| P1            | 10,412  | 85.2    | 85.2   |
| P2            | 203     | 1.9     | 1.7    |
| P3            | 56      | 0.5     | 0.5    |

BD FACSDiva 8.0.2

BD FACSDiva 8.0.2

Supplement: Supplementary file 10 — Figure EV Source Data [file 44319_2024_256_MOESM10_ESM.zip › SourceDateForExpanded View/Figure EV3/3E/DC,2f,Mo_20230723_aa-A2-1_23072023204859.pdf]

# BD FACSDiva 8.0.2

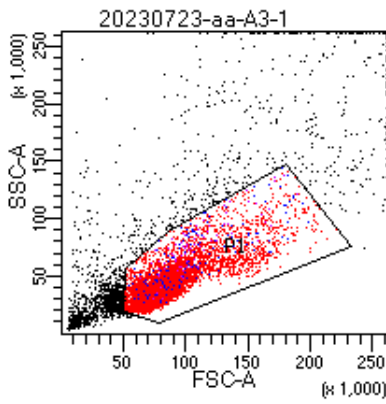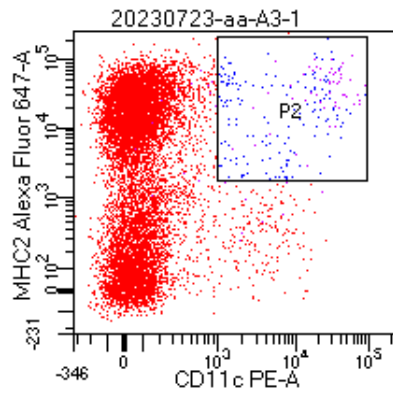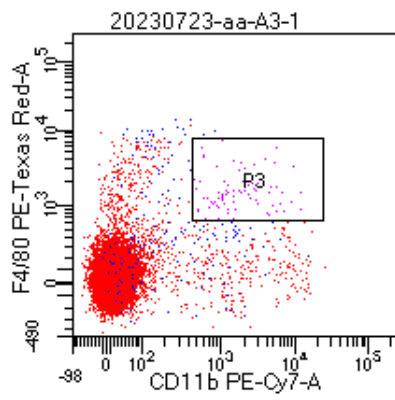

| Tube: aa-A3-1 |         |         |        |
|---------------|---------|---------|--------|
| Population    | #Events | %Parent | %Total |
| All Events    | 12,553  | ####    | 100.0  |
| P1            | 10,692  | 85.2    | 85.2   |
| P2            | 211     | 2.0     | 1.7    |
| P3            | 77      | 0.7     | 0.6    |

BD FACSDiva 8.0.2

BD FACSDiva 8.0.2

Supplement: Supplementary file 10 — Figure EV Source Data [file 44319_2024_256_MOESM10_ESM.zip › SourceDateForExpanded View/Figure EV3/3E/DC,2f,Mo_20230723_aa-A3-1_23072023204903.pdf]

# BD FACSDiva 8.0.2

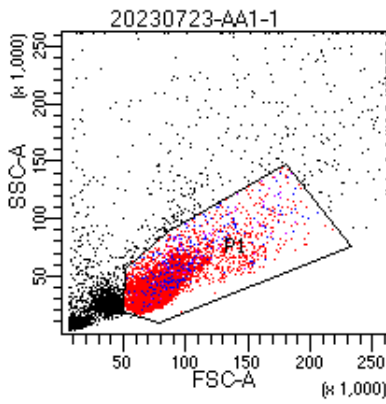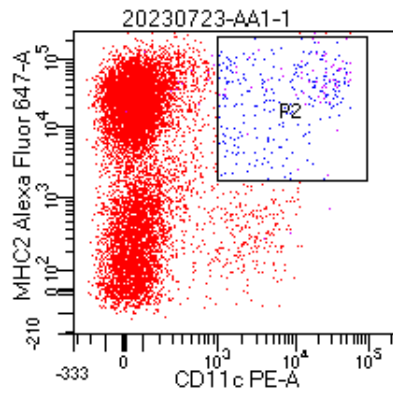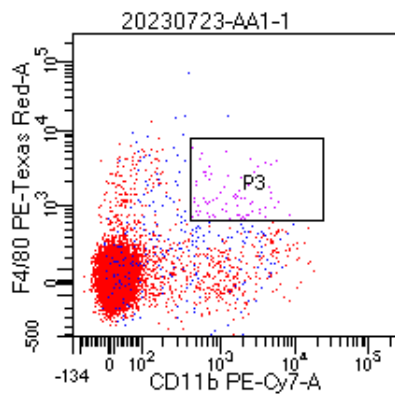

| Tube: AA1-1 |         |         |        |
|-------------|---------|---------|--------|
| Population  | #Events | %Parent | %Total |
| All Events  | 12,793  | ####    | 100.0  |
| P1          | 10,432  | 81.5    | 81.5   |
| P2          | 298     | 2.9     | 2.3    |
| P3          | 74      | 0.7     | 0.6    |

BD FACSDiva 8.0.2

BD FACSDiva 8.0.2

Supplement: Supplementary file 10 — Figure EV Source Data [file 44319_2024_256_MOESM10_ESM.zip › SourceDateForExpanded View/Figure EV3/3E/DC,2f,Mo_20230723_AA1-1_23072023204835.pdf]

# BD FACSDiva 8.0.2

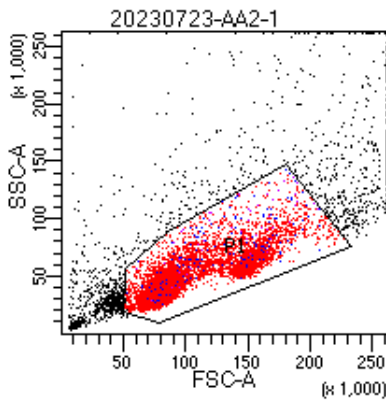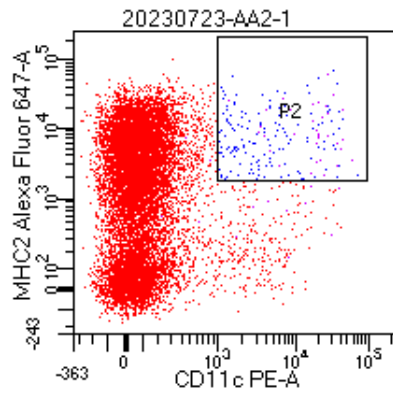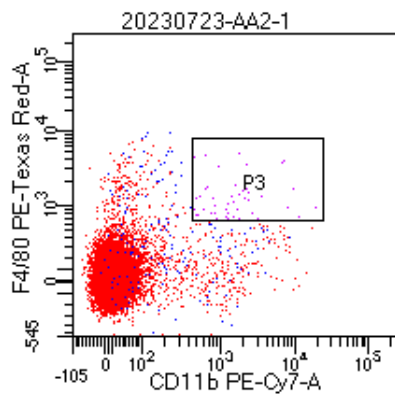

| Tube: AA2-1 |         |         |        |
|-------------|---------|---------|--------|
| Population  | #Events | %Parent | %Total |
| All Events  | 13,319  | ####    | 100.0  |
| P1          | 11,828  | 88.8    | 88.8   |
| P2          | 193     | 1.6     | 1.4    |
| P3          | 49      | 0.4     | 0.4    |

BD FACSDiva 8.0.2

BD FACSDiva 8.0.2

Supplement: Supplementary file 10 — Figure EV Source Data [file 44319_2024_256_MOESM10_ESM.zip › SourceDateForExpanded View/Figure EV3/3E/DC,2f,Mo_20230723_AA2-1_23072023204840.pdf]

# BD FACSDiva 8.0.2

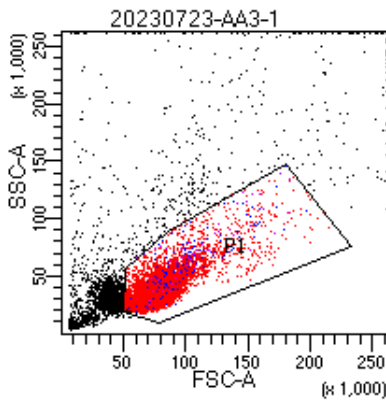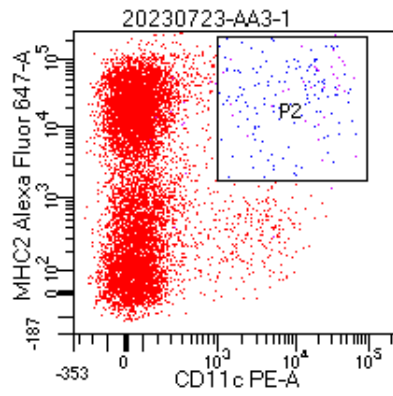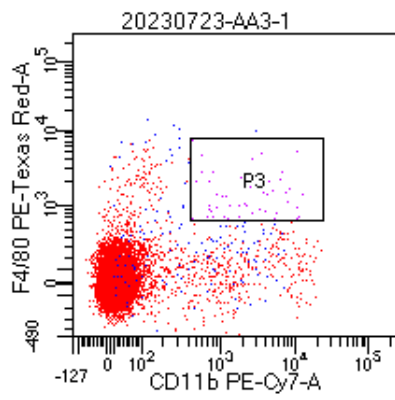

| Tube: AA3-1 |         |         |        |
|-------------|---------|---------|--------|
| Population  | #Events | %Parent | %Total |
| All Events  | 12,619  | ####    | 100.0  |
| P1          | 10,244  | 81.2    | 81.2   |
| P2          | 169     | 1.6     | 1.3    |
| P3          | 49      | 0.5     | 0.4    |

BD FACSDiva 8.0.2

BD FACSDiva 8.0.2

Supplement: Supplementary file 10 — Figure EV Source Data [file 44319_2024_256_MOESM10_ESM.zip › SourceDateForExpanded View/Figure EV3/3E/DC,2f,Mo_20230723_AA3-1_23072023204846.pdf]

# BD FACSDiva 8.0.2

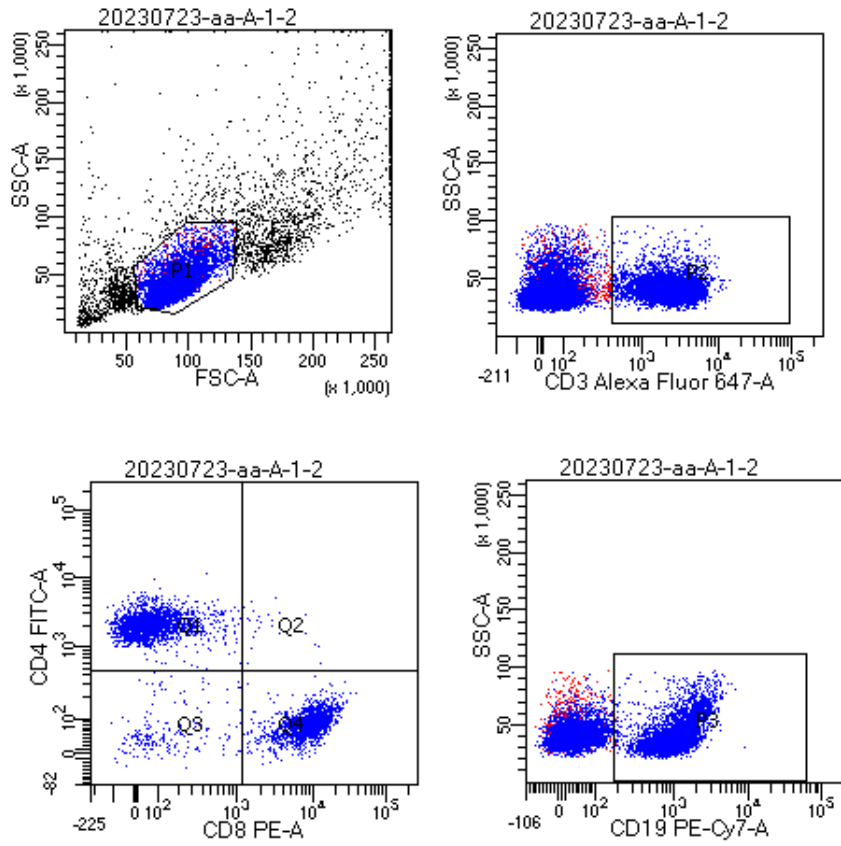

| Tube: aa-A-1-2 |         |         |        |
|----------------|---------|---------|--------|
| Population     | #Events | %Parent | %Total |
| ■ All Events   | 12,630  | ####    | 100.0  |
| ■ P1           | 10,006  | 79.2    | 79.2   |
| ■ P2           | 4,125   | 41.2    | 32.7   |
| ☒ Q1           | 2,309   | 56.0    | 18.3   |
| ☒ Q2           | 24      | 0.6     | 0.2    |
| ☒ Q3           | 194     | 4.7     | 1.5    |
| ☒ Q4           | 1,598   | 38.7    | 12.7   |
| ■ P3           | 5,466   | 54.6    | 43.3   |

Supplement: Supplementary file 10 — Figure EV Source Data [file 44319_2024_256_MOESM10_ESM.zip › SourceDateForExpanded View/Figure EV3/3E/T,2f,B cell_20230723_aa-A-1-2_23072023204121.pdf]

# BD FACSDiva 8.0.2

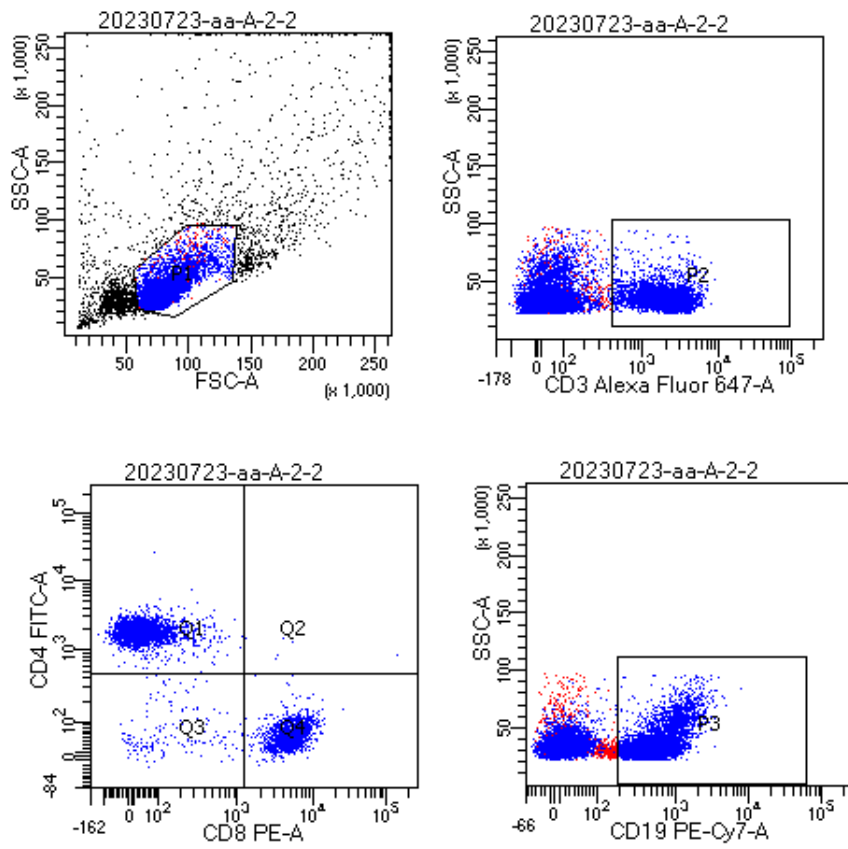

| Tube: aa-A-2-2 |         |         |        |  |
|----------------|---------|---------|--------|--|
| Population     | #Events | %Parent | %Total |  |
| ■ All Events   | 12,226  | ####    | 100.0  |  |
| ■ P1           | 10,054  | 82.2    | 82.2   |  |
| ■ P2           | 3,925   | 39.0    | 32.1   |  |
| ▣ Q1           | 2,516   | 64.1    | 20.6   |  |
| ▣ Q2           | 7       | 0.2     | 0.1    |  |
| ▣ Q3           | 157     | 4.0     | 1.3    |  |
| ▣ Q4           | 1,245   | 31.7    | 10.2   |  |
| ■ P3           | 5,496   | 54.7    | 45.0   |  |

Supplement: Supplementary file 10 — Figure EV Source Data [file 44319_2024_256_MOESM10_ESM.zip › SourceDateForExpanded View/Figure EV3/3E/T,2f,B cell_20230723_aa-A-2-2_23072023204129.pdf]

# BD FACSDiva 8.0.2

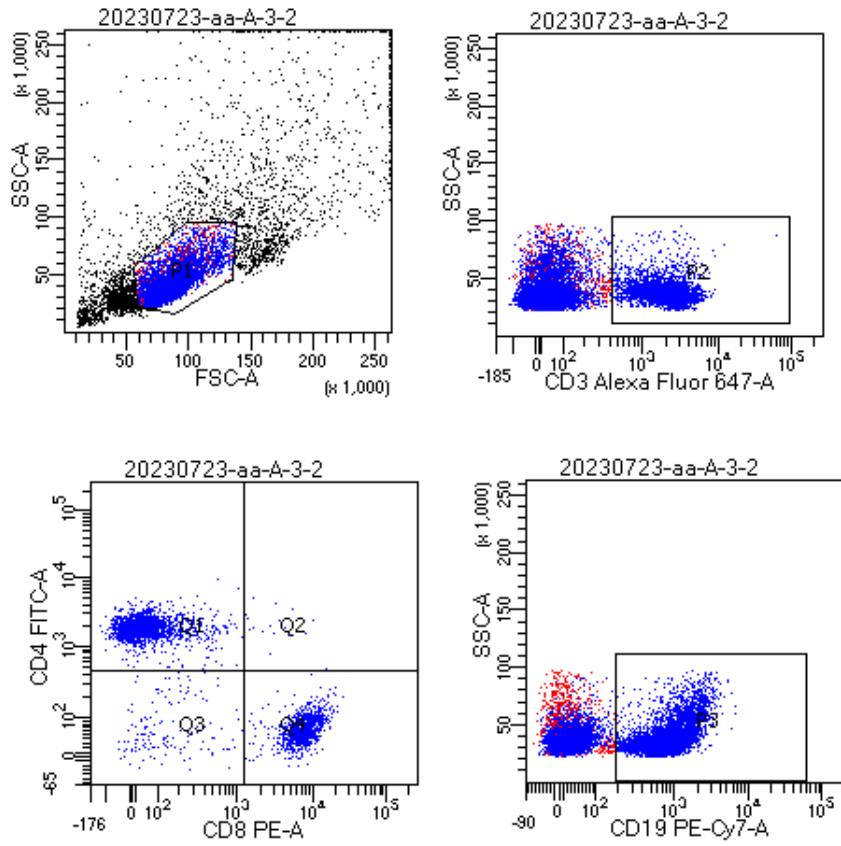

| Tube: aa-A-3-2 |         |         |        |
|----------------|---------|---------|--------|
| Population     | #Events | %Parent | %Total |
| All Events     | 13,002  | ####    | 100.0  |
| P1             | 10,074  | 77.5    | 77.5   |
| P2             | 3,184   | 31.6    | 24.5   |
| Q1             | 1,986   | 62.4    | 15.3   |
| Q2             | 17      | 0.5     | 0.1    |
| Q3             | 140     | 4.4     | 1.1    |
| Q4             | 1,041   | 32.7    | 8.0    |
| P3             | 5,928   | 58.8    | 45.6   |

Supplement: Supplementary file 10 — Figure EV Source Data [file 44319_2024_256_MOESM10_ESM.zip › SourceDateForExpanded View/Figure EV3/3E/T,2f,B cell_20230723_aa-A-3-2_23072023204135.pdf]

# BD FACSDiva 8.0.2

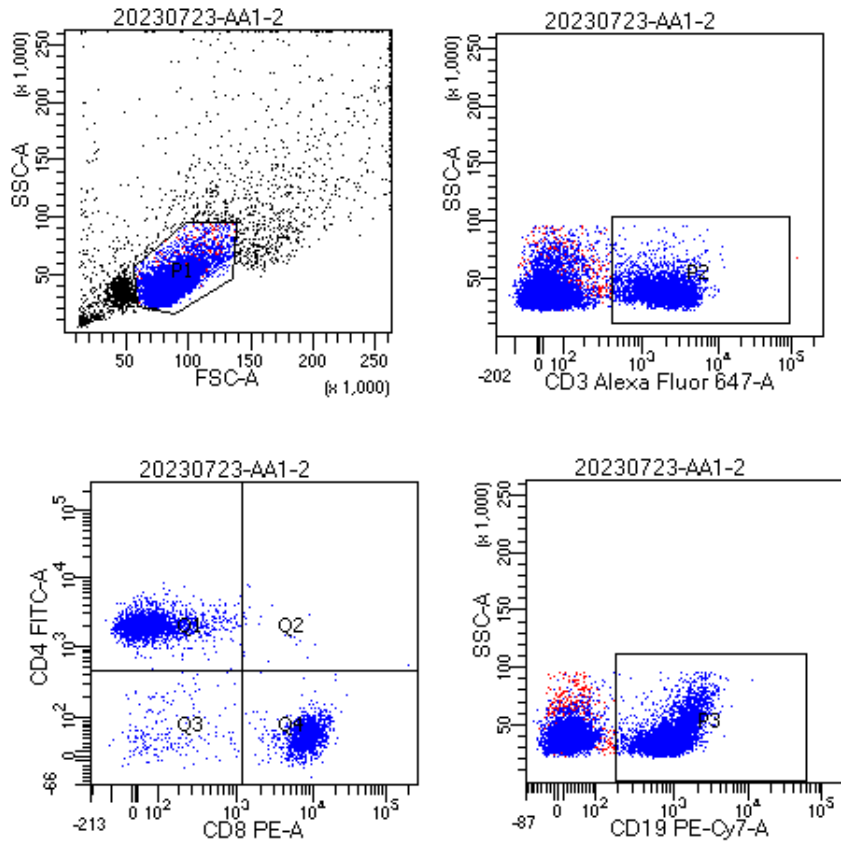

| Tube: AA1-2  |         |         |        |  |
|--------------|---------|---------|--------|--|
| Population   | #Events | %Parent | %Total |  |
| ■ All Events | 12,357  | ####    | 100.0  |  |
| ■ P1         | 10,044  | 81.3    | 81.3   |  |
| ■ P2         | 3,774   | 37.6    | 30.5   |  |
| ▣ Q1         | 2,265   | 60.0    | 18.3   |  |
| ▣ Q2         | 22      | 0.6     | 0.2    |  |
| ▣ Q3         | 191     | 5.1     | 1.5    |  |
| ▣ Q4         | 1,296   | 34.3    | 10.5   |  |
| ■ P3         | 5,785   | 57.6    | 46.8   |  |

Supplement: Supplementary file 10 — Figure EV Source Data [file 44319_2024_256_MOESM10_ESM.zip › SourceDateForExpanded View/Figure EV3/3E/T,2f,B cell_20230723_AA1-2_23072023204058.pdf]

# BD FACSDiva 8.0.2

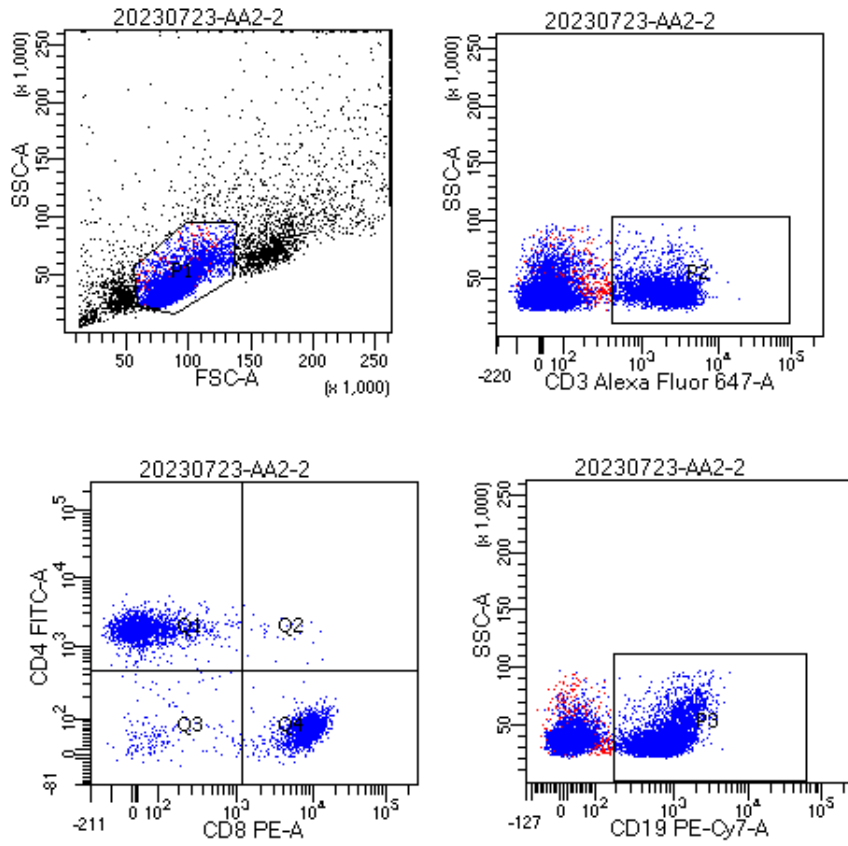

| Tube: AA2-2  |         |         |        |  |
|--------------|---------|---------|--------|--|
| Population   | #Events | %Parent | %Total |  |
| ■ All Events | 13,202  | ####    | 100.0  |  |
| ■ P1         | 10,034  | 76.0    | 76.0   |  |
| ■ P2         | 3,460   | 34.5    | 26.2   |  |
| ▣ Q1         | 2,094   | 60.5    | 15.9   |  |
| ▣ Q2         | 26      | 0.8     | 0.2    |  |
| ▣ Q3         | 171     | 4.9     | 1.3    |  |
| ▣ Q4         | 1,169   | 33.8    | 8.9    |  |
| ■ P3         | 6,012   | 59.9    | 45.5   |  |

Supplement: Supplementary file 10 — Figure EV Source Data [file 44319_2024_256_MOESM10_ESM.zip › SourceDateForExpanded View/Figure EV3/3E/T,2f,B cell_20230723_AA2-2_23072023204110.pdf]

# BD FACSDiva 8.0.2

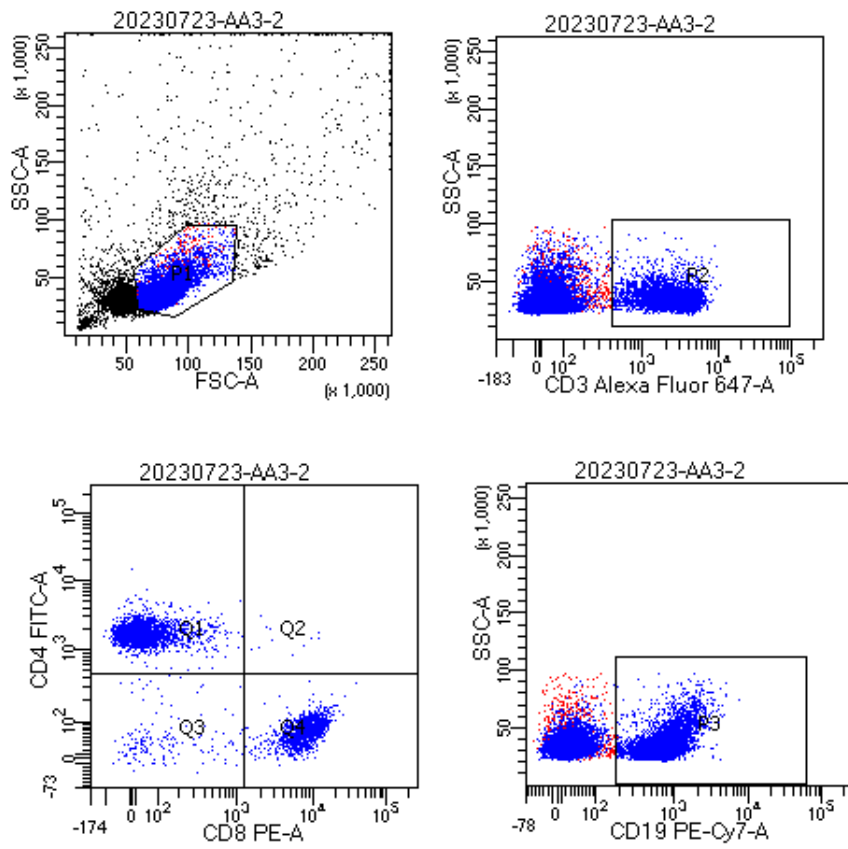

| Tube: AA3-2  |         |         |        |
|--------------|---------|---------|--------|
| Population   | #Events | %Parent | %Total |
| ■ All Events | 12,813  | ####    | 100.0  |
| ■ P1         | 10,043  | 78.4    | 78.4   |
| ■ P2         | 4,129   | 41.1    | 32.2   |
| ▣ Q1         | 2,558   | 62.0    | 20.0   |
| ▣ Q2         | 12      | 0.3     | 0.1    |
| ▣ Q3         | 157     | 3.8     | 1.2    |
| ▣ Q4         | 1,402   | 34.0    | 10.9   |
| ■ P3         | 5,166   | 51.4    | 40.3   |

Supplement: Supplementary file 10 — Figure EV Source Data [file 44319_2024_256_MOESM10_ESM.zip › SourceDateForExpanded View/Figure EV3/3E/T,2f,B cell_20230723_AA3-2_23072023204116.pdf]

# BD FACSDiva 8.0.2

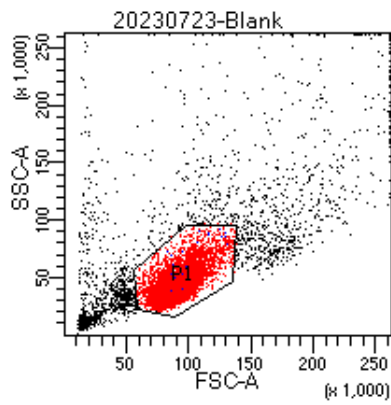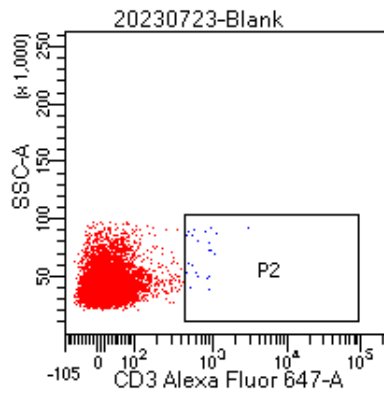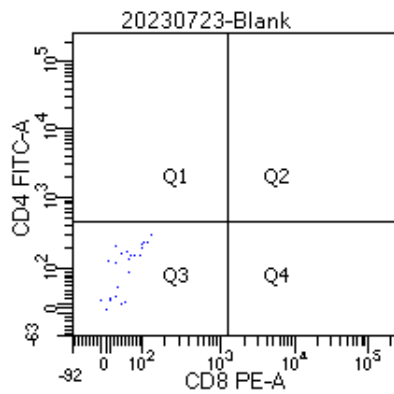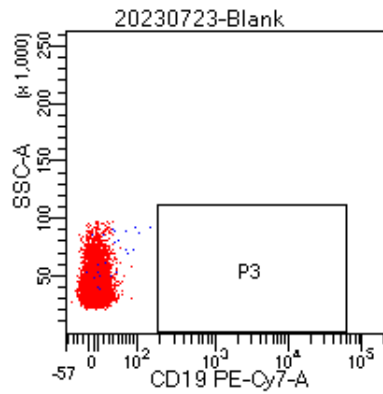

| Tube: Blank  |         |         |        |
|--------------|---------|---------|--------|
| Population   | #Events | %Parent | %Total |
| ■ All Events | 11,794  | ####    | 100.0  |
| ■ P1         | 10,013  | 84.9    | 84.9   |
| ■ P2         | 24      | 0.2     | 0.2    |
| ▣ Q1         | 0       | 0.0     | 0.0    |
| ▣ Q2         | 0       | 0.0     | 0.0    |
| ▣ Q3         | 24      | 100.0   | 0.2    |
| ▣ Q4         | 0       | 0.0     | 0.0    |
| ■ P3         | 0       | 0.0     | 0.0    |

Supplement: Supplementary file 10 — Figure EV Source Data [file 44319_2024_256_MOESM10_ESM.zip › SourceDateForExpanded View/Figure EV3/3E/T,2f,B cell_20230723_Blank_23072023204031.pdf]

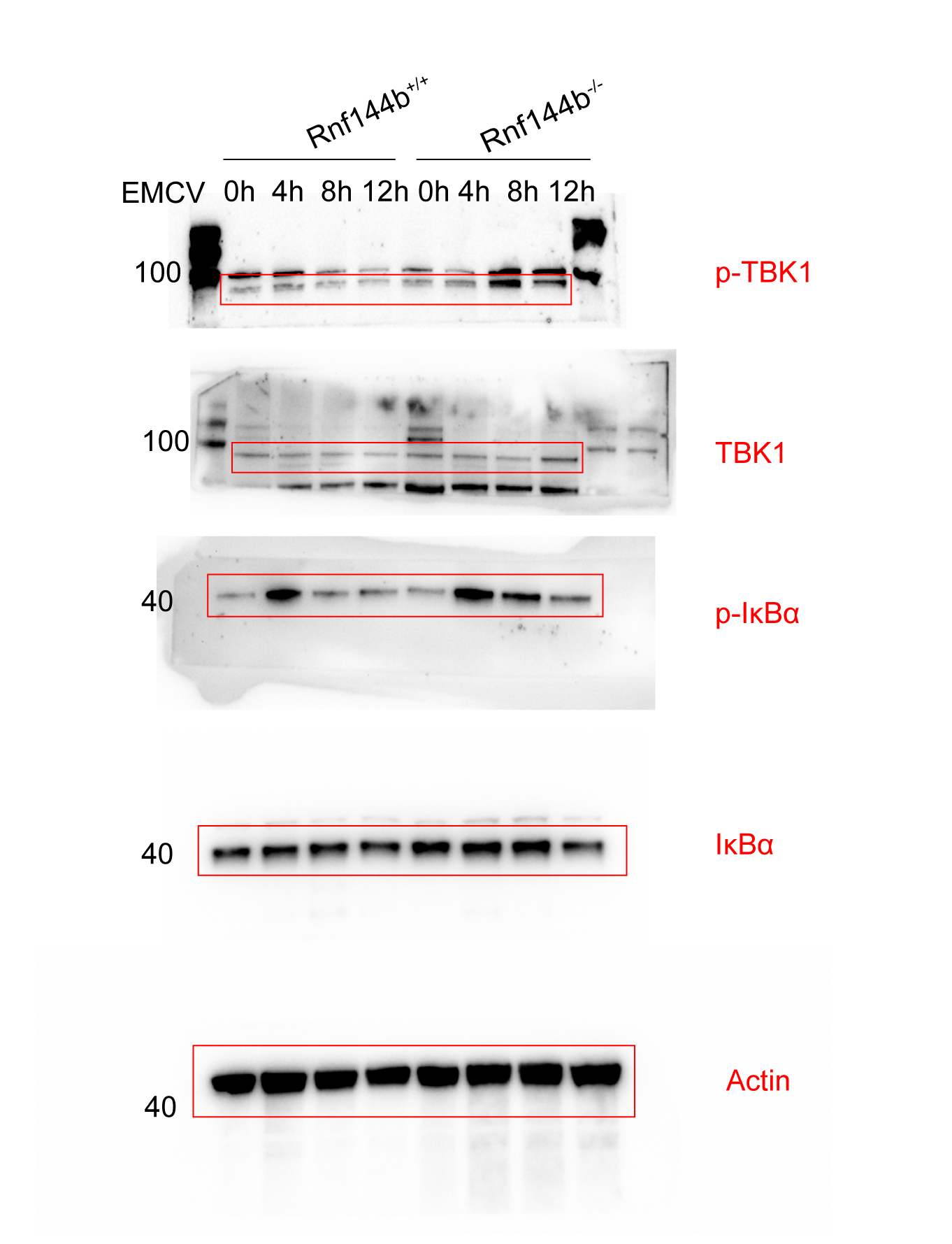

Supplement: Supplementary file 10 — Figure EV Source Data [file 44319_2024_256_MOESM10_ESM.zip › SourceDateForExpanded View/Figure EV4/4D.tif]

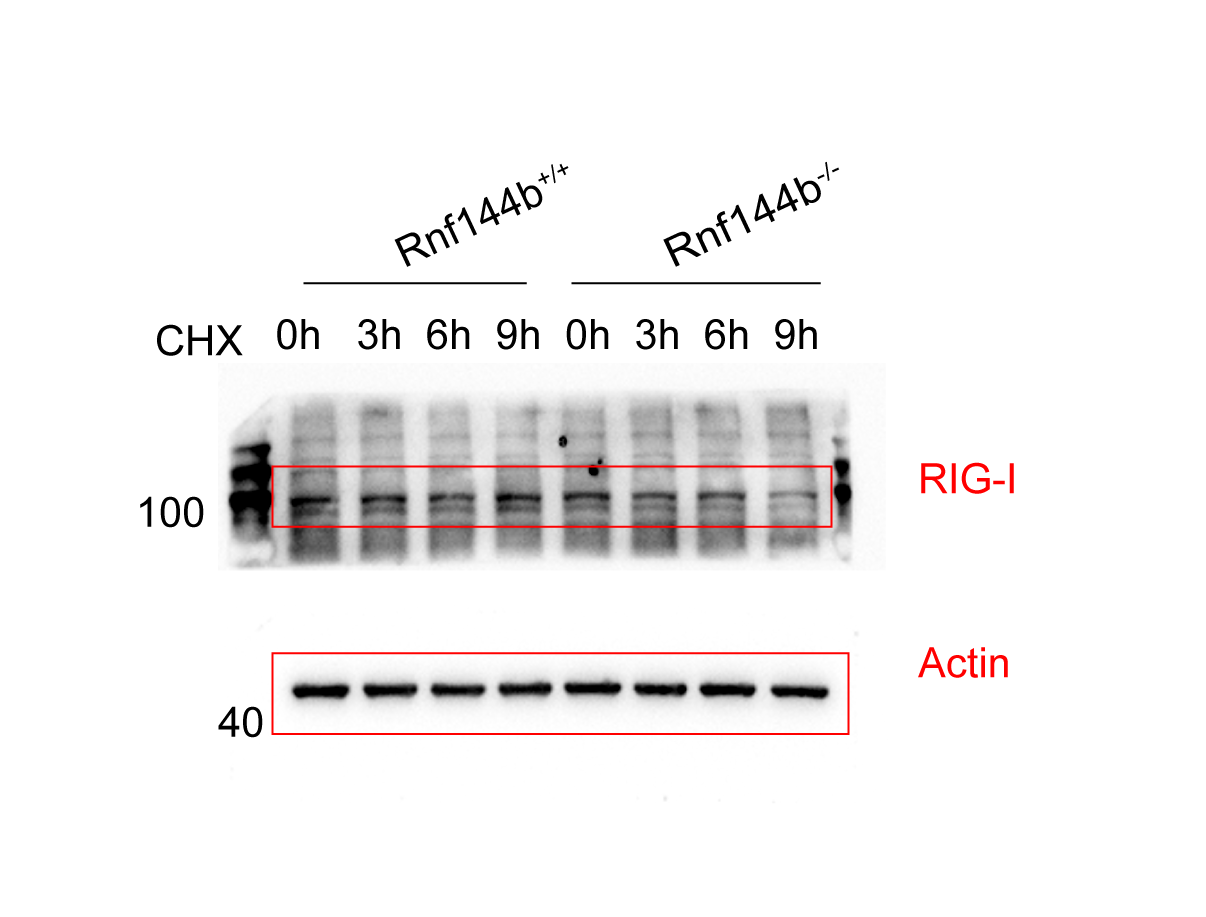

Supplement: Supplementary file 10 — Figure EV Source Data [file 44319_2024_256_MOESM10_ESM.zip › SourceDateForExpanded View/Figure EV4/4G.tif]

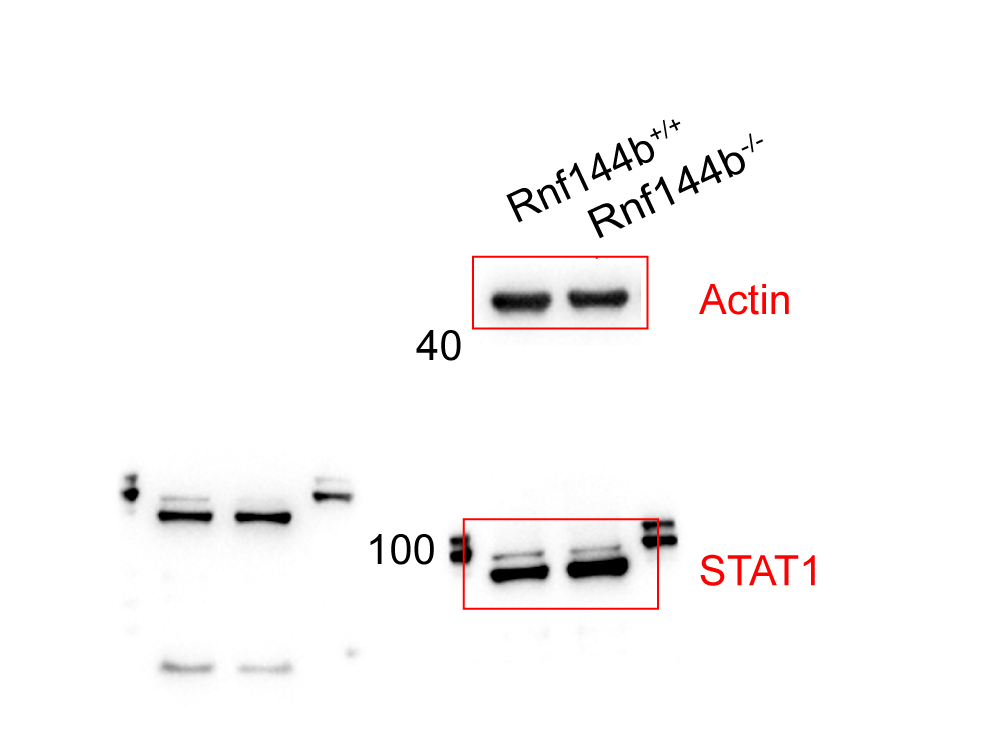

Supplement: Supplementary file 10 — Figure EV Source Data [file 44319_2024_256_MOESM10_ESM.zip › SourceDateForExpanded View/Figure EV5/5B.tif]

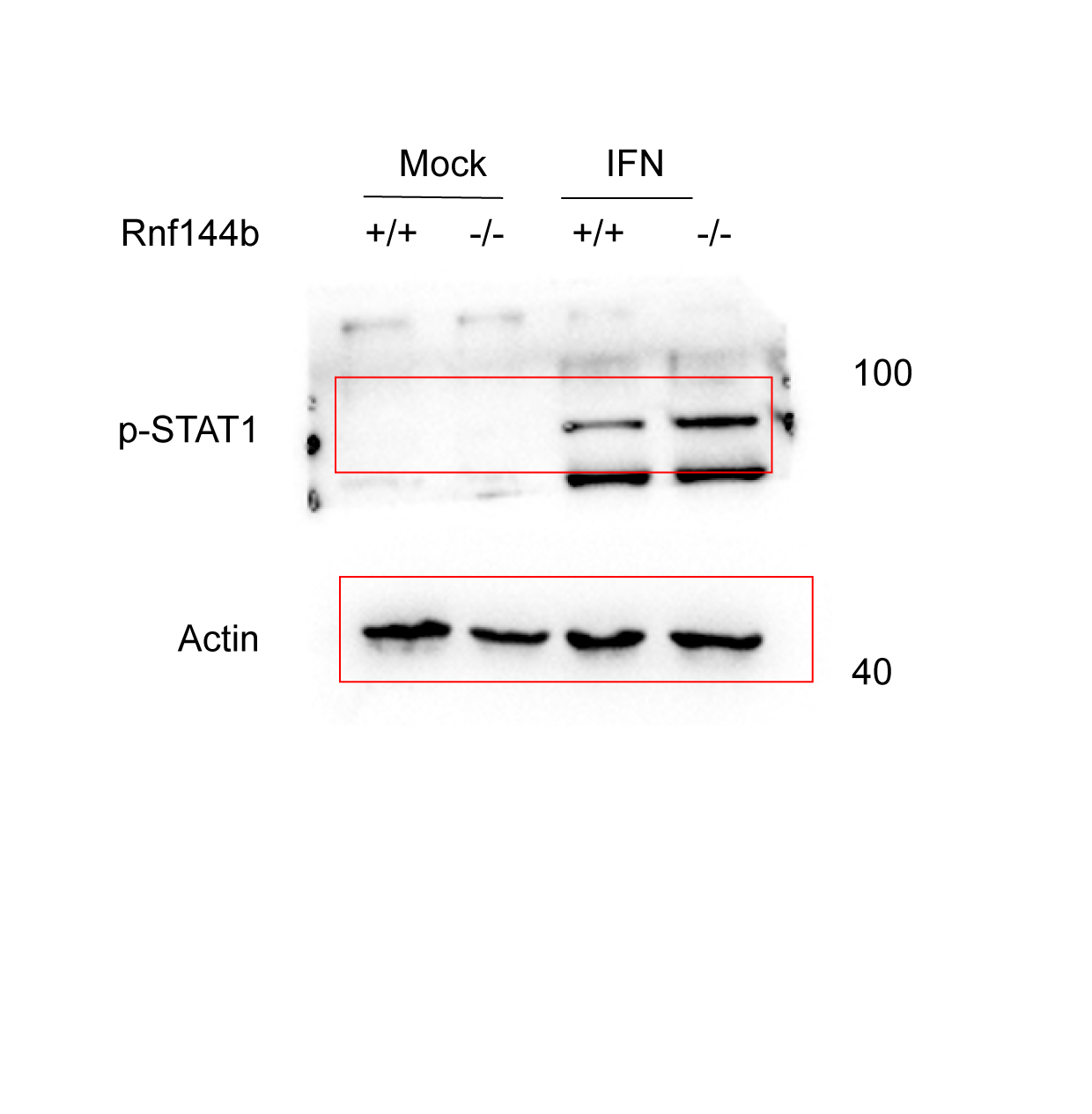

Supplement: Supplementary file 10 — Figure EV Source Data [file 44319_2024_256_MOESM10_ESM.zip › SourceDateForExpanded View/Figure EV5/5C.tif]

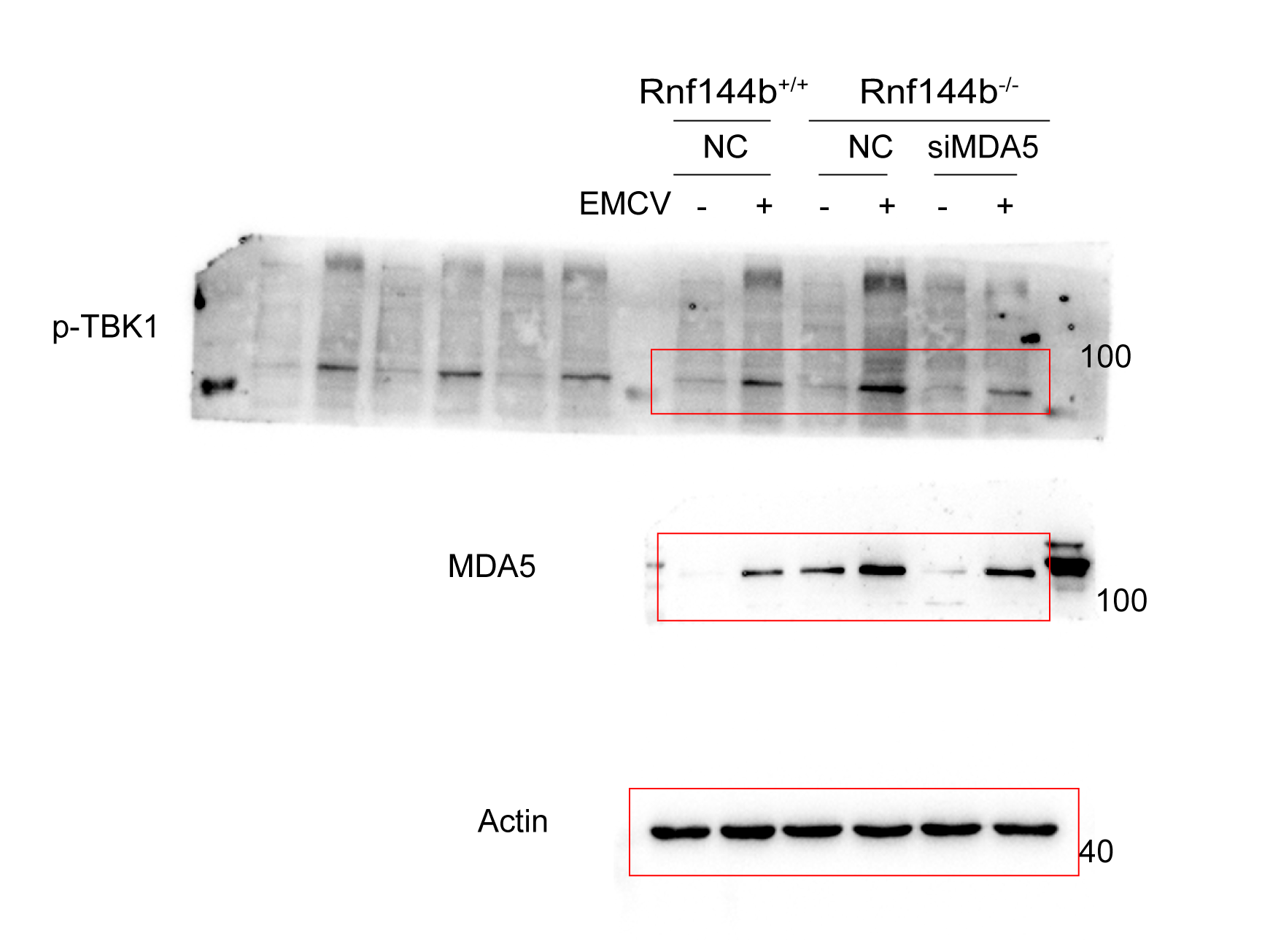

Supplement: Supplementary file 10 — Figure EV Source Data [file 44319_2024_256_MOESM10_ESM.zip › SourceDateForExpanded View/Figure EV5/5E.tif]

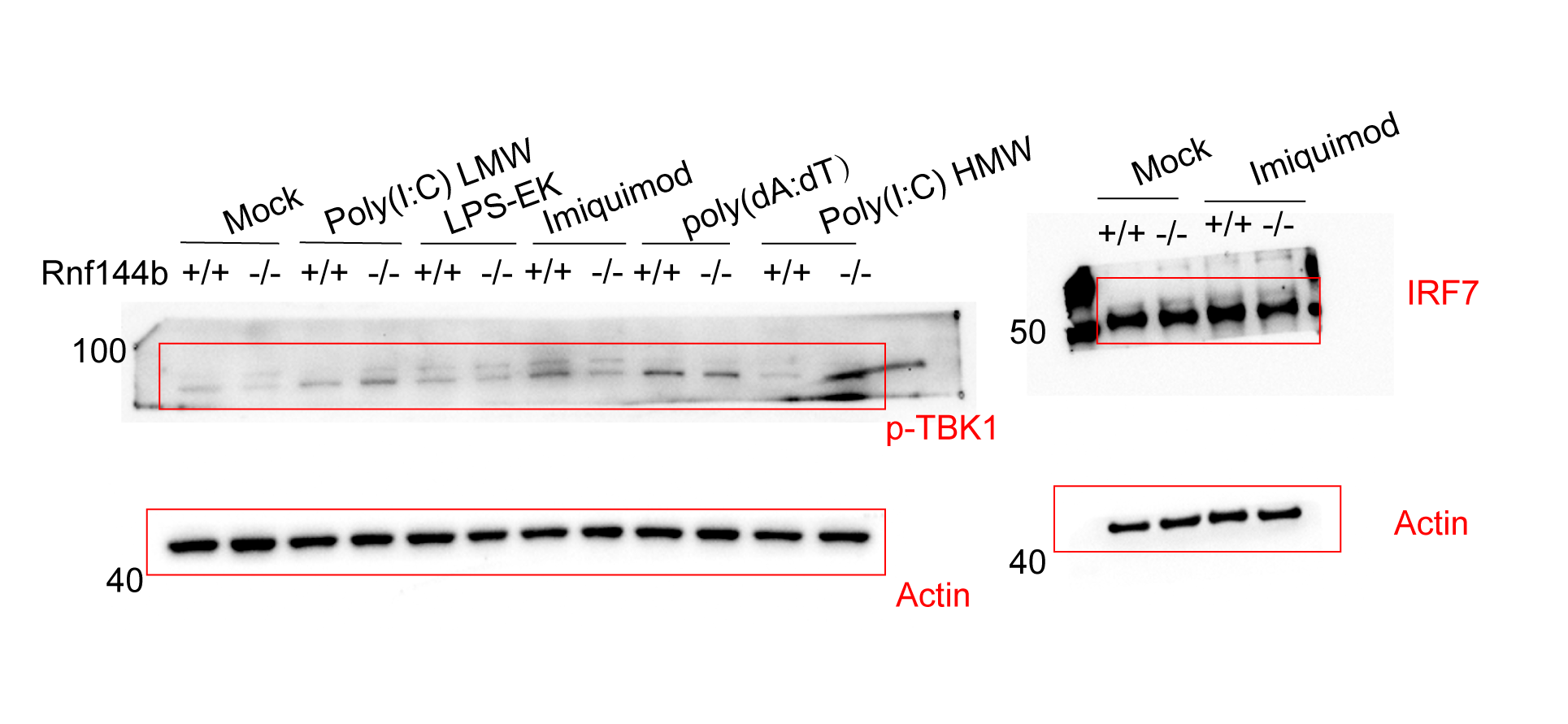

Supplement: Supplementary file 10 — Figure EV Source Data [file 44319_2024_256_MOESM10_ESM.zip › SourceDateForExpanded View/Figure EV6/6A.tif]

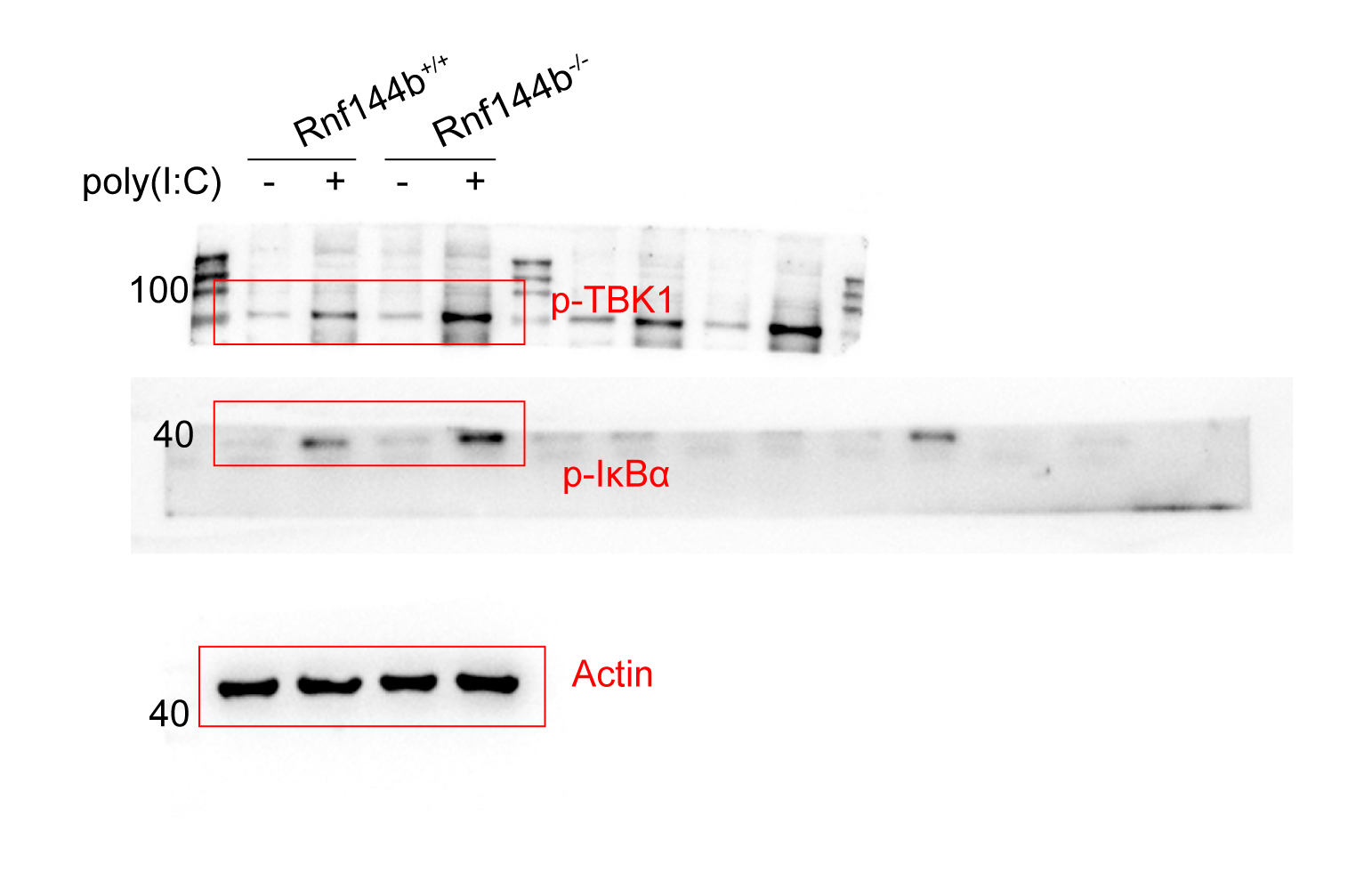

Supplement: Supplementary file 10 — Figure EV Source Data [file 44319_2024_256_MOESM10_ESM.zip › SourceDateForExpanded View/Figure EV6/6B.tif]
