# Supplementary material for: RNF144B negatively regulates antiviral immunity by targeting MDA5 for autophagic degradation
Source: EMBO Rep. 2024 Sep 16;25(10):4594–624. doi: 10.1038/s44319-024-00256-w (PMC11467429; doi:10.1038/s44319-024-00256-w)
Supplement: Supplementary file 11 — Expanded View Figures [file 44319_2024_256_MOESM11_ESM.pdf]

Expanded View Figures

Figure EV1. RNF144B suppresses RNA-induced innate immune responses.

(**A**) Venn diagram of common DEGs from RNA-Seq data of host cells infected with porcine reproductive and respiratory syndrome virus, foot-and-mouth disease virus, and Seneca virus A. (**B–D**) Validation of knockdown efficiency of siRNAs targeting FJX1, ZC3H12C, and RNF144B in HEK293T cells. $n = 3$ biological replicates. Statistical significance was determined by two-tailed unpaired Student's t-test. "ns" indicates no significant difference. $^{**}P = 0.0017$ (siFJX1-1088), $^{***}P = 0.0002$ (siFJX1-1229), $^{****}P = 0.00001$ (siZC3H12C-186), $^{***}P = 0.0004$ (siZC3H12C-1564), $^{**}P = 0.009$ (siZC3H12C-2254), $^{***}P = 0.0002$ (siRNF144B-532), $^{***}P = 0.0005$ (siRNF144B-976), $^{***}P = 0.0008$ (siRNF144B-1112). (**E**, **F**) HEK293T cells were transfected with *NC* or *siRNA* targeting FJX1, ZC3H12C, and RNF144B for 36 h. Cells were infected with VSV (MOI = 0.1) for indicated time points and subjected to qPCR analysis for VSV mRNA level. $n = 3$ biological replicates. Statistical significance was determined by two-tailed unpaired Student's t-test. "ns" indicates no significant difference. $^{***}P = 0.00010$ (**F**). (**G**) HEK293T cells were transfected with NC or *siRNA* targeting RNF144B for 36 h. Cells were infected with EMCV (MOI = 1) for 12 h and subjected to qPCR analysis for EMCV mRNA level. $n = 3$ biological replicates. Statistical significance was determined by two-tailed unpaired Student's t-test. $^{*}P = 0.02080$. (**H**) HEK293T cells were transfected with empty vector or RNF144B-Myc for 18 h. Cells were infected with EMCV (MOI = 1) for 12 h and subjected to qPCR analysis for EMCV mRNA level. $n = 3$ biological replicates. Statistical significance was determined by two-tailed unpaired Student's t-test. $^{**}P = 0.00110$. (**I**) Immunoblot analysis of RNF144B expression in HEK293T cells stably expressing *shRNA* against RNF144B. (**J–M**) Control or RNF144B KD HEK293T cells were transfected with poly (I:C) for 12 h and subjected to qPCR analysis for IFNB1 and ISG15 mRNA level. $n = 3$ biological replicates. Statistical significance was determined by two-tailed unpaired Student's t-test. $^{***}P = 0.0005$ (**J**), $^{**}P = 0.0065$ (**K**), $^{**}P = 0.0057$ (**L**), $^{**}P = 0.0075$ (**M**). (**N**) HEK293T cells were transfected with poly(I:C) for 12 h and subjected to qPCR analysis of RNF144B mRNA level. $n = 3$ biological replicates. Statistical significance was determined by two-tailed unpaired Student's t-test. $^{**}P = 0.0049$. Data information: Data shown are representative of at least three biological replicates, with each data point representing a biological experiment. Error bars are presented as mean ± SD. Statistical significance was determined by Student's t-test. Source data are available online for this figure.

 © The Author(s)

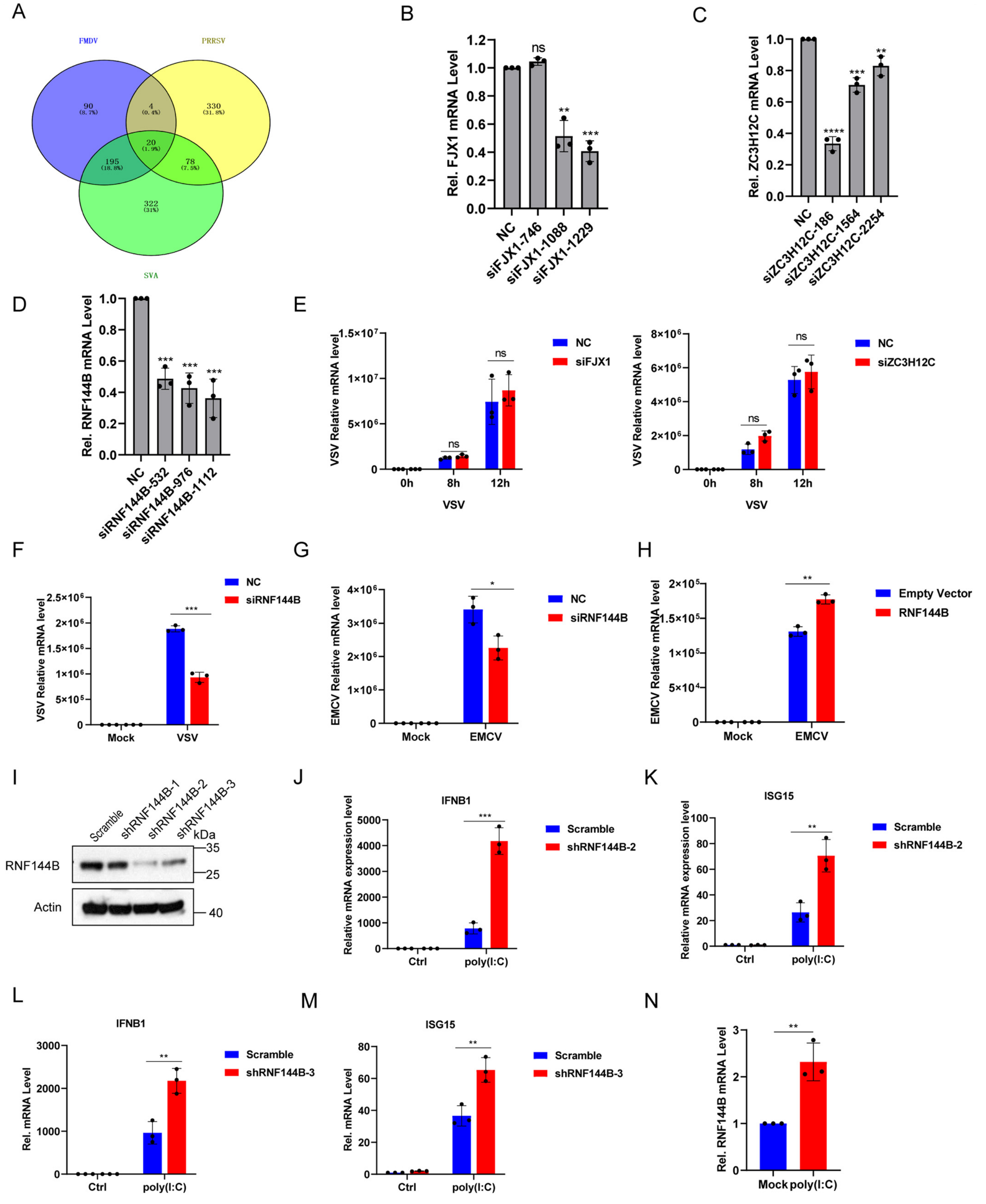
A
FMDV
PRRSV
SVA
90 (8.7%)
4 (0.4%)
330 (31.8%)
20 (1.9%)
195 (18.8%)
78 (7.5%)
322 (31%)
B
Rel. FJX1 mRNA Level
NC
siFJX1-746
siFJX1-1088
siFJX1-1229
ns
**
***
C
Rel. ZC3H12C mRNA Level
NC
siZC3H12C-186
siZC3H12C-1564
siZC3H12C-2254
****
***
**
D
Rel. RNF144B mRNA Level
NC
siRNF144B-532
siRNF144B-976
siRNF144B-1112
E
VSV Relative mRNA level
NC
siFJX1
siZC3H12C
0h
8h
12h
VSV
F
VSV Relative mRNA level
NC
siRNF144B
Mock
VSV
G
EMCV Relative mRNA level
Mock
EMCV
H
Empty Vector
RNF144B
I
Scramble
shRNF144B-1
shRNF144B-2
shRNF144B-3
kDa
RNF144B
Actin
35
25
40
J
IFNB1
Relative mRNA expression level
Scramble
shRNF144B-2
Ctrl
poly(I:C)
K
ISG15
L
IFNB1
Rel. mRNA Level
shRNF144B-3
M
ISG15
N
Rel. RNF144B mRNA Level
Mock
poly(I:C)

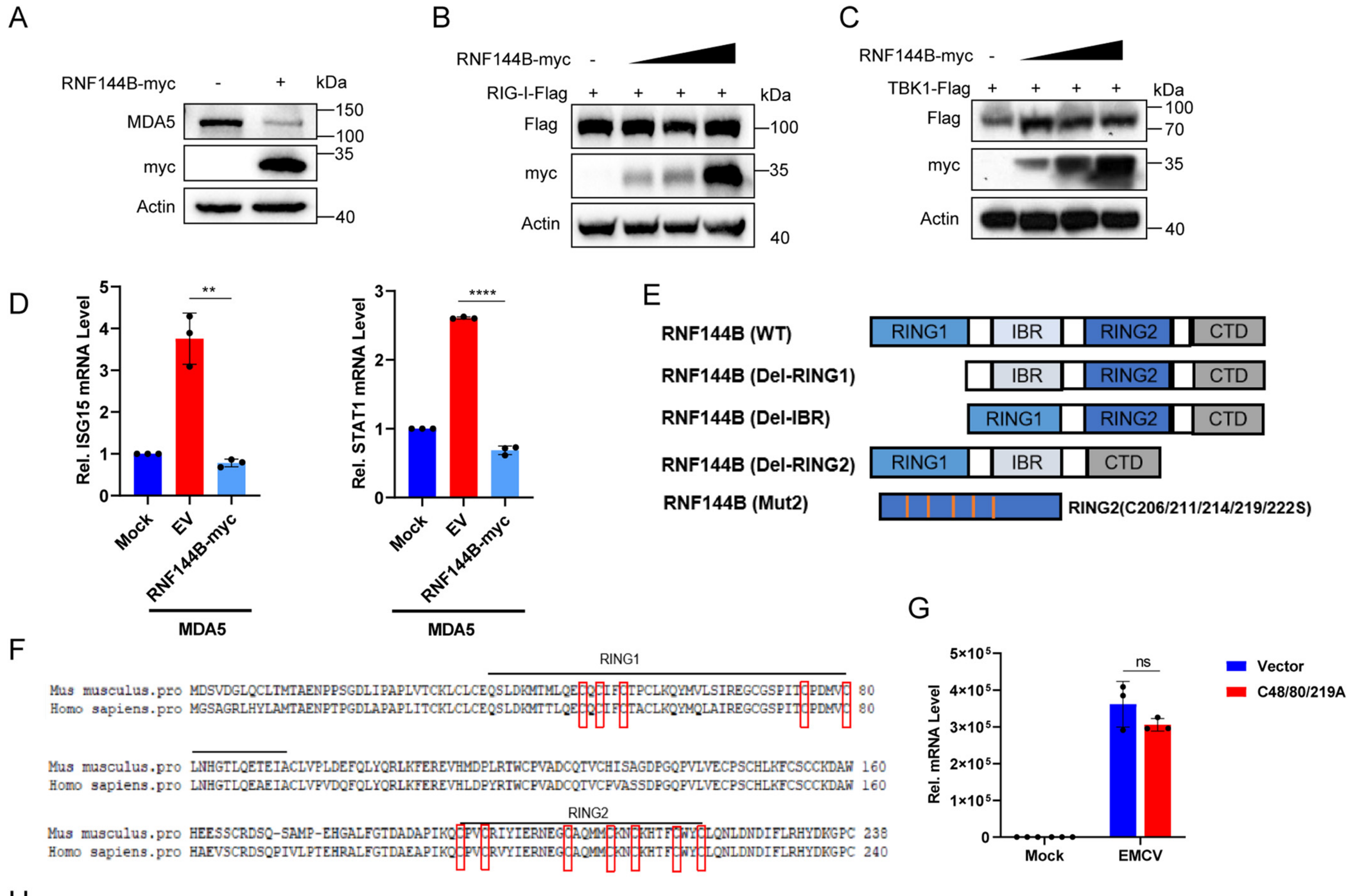

H

Ubiquitin residue	6	11	27	29	33	48	63
WT	K	K	K	K	K	K	K
K6	K	R	R	R	R	R	R
K11	R	K	R	R	R	R	R
K27	R	R	K	R	R	R	R
K29	R	R	R	K	R	R	R
K33	R	R	R	R	K	R	R
K48	R	R	R	R	R	K	R
K63	R	R	R	R	R	R	K
K0	R	R	R	R	R	R	R

Ubiquitin residue	6	11	27	29	33	48	63
WT	K	K	K	K	K	K	K
K6R	R	K	K	K	K	K	K
K11R	K	R	K	K	K	K	K
K27R	K	K	R	K	K	K	K
K29R	K	K	K	R	K	K	K
K33R	K	K	K	K	R	K	K
K48R	K	K	K	K	K	R	K
K63R	K	K	K	K	K	K	R

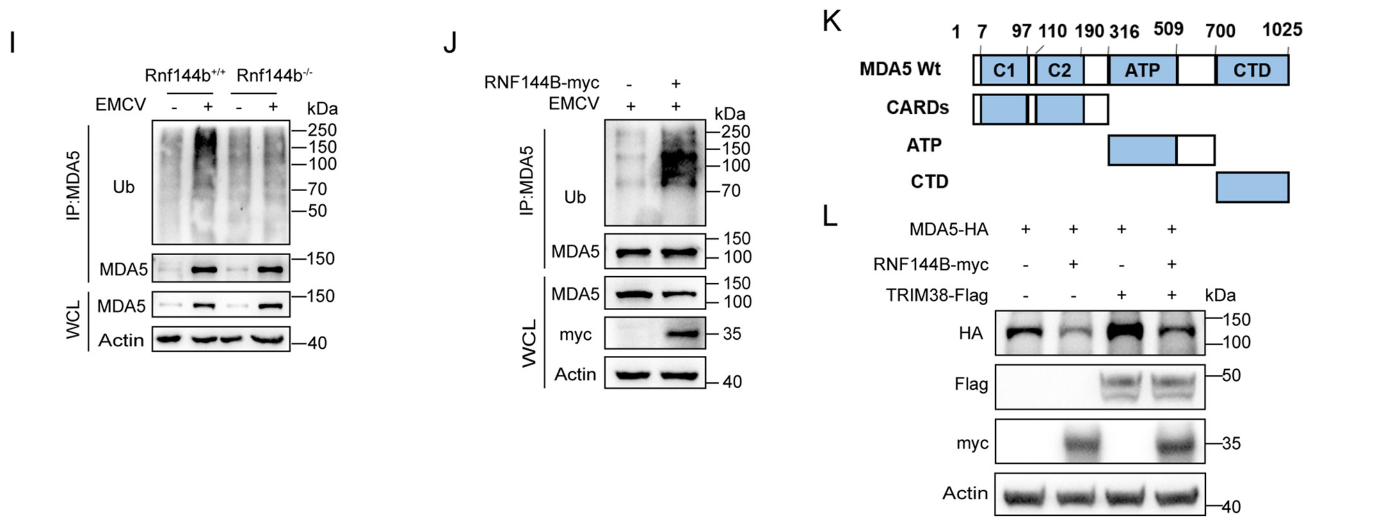

 © The Author(s)

Figure EV2. RNF144B targets MDA5.

(**A**) A549 cells were transfected with plasmid encoding RNF144B. 18 h post-transfection, protein extracts were used for immunoblot analysis of the endogenous MDA5 protein level. (**B**) HEK293T cells were co-transfected with empty vector or RNF144B-Myc (2 μg) and RIG-I-Flag. 18 h post-transfection, cell lysates were analyzed by immunoblot using the indicated antibodies. (**C**) HEK293T cells were co-transfected with empty vector or RNF144B-Myc (2 μg) and TBK1-Flag. 18 h post-transfection, cell lysates were analyzed by immunoblot using the indicated antibodies. (**D**) HEK293T cells were transfected with plasmids encoding RNF144B-myc and MDA5-Flag. 18 h post-transfection, cells were subjected to qPCR analysis for ISG15 and STAT1 mRNA level. $n = 3$ biological replicates. Statistical significance was determined by two-tailed unpaired Student's t-test. $^{**}P = 0.0011$, $^{****}P = 0.000001$. (**E**) The structure of RNF144B and its mutants. (**F**) The amino acid sequence of RING1 and RING2 domains of RNF144B. (**G**) HEK293T cells were transfected with empty vector or C48/80/219A-Myc plasmid. After 18 h, cells were infected with EMCV (MOI = 1) for 12 h. and subjected to qPCR analysis for EMCV mRNA level. $n = 3$ biological replicates. Statistical significance was determined by two-tailed unpaired Student's t-test. "ns" indicates no significant difference. (**H**) The model of mutant plasmids of Ubiquitin. (**I**) *Rnf144b*$^{+/+}$ and *Rnf144b*$^{-/-}$ MEFs infected with EMCV (MOI = 1) for 12 h. Cell lysates were harvested and immunoprecipitated using anti-MDA5 antibody, followed by immunoblots using the indicated antibodies. (**J**) A549 cells were transfected with RNF144B-myc. After 18 h, cells were infected with EMCV (MOI = 1) for indicated time points. Cell lysates were subjected to immunoprecipitation with anti-MDA5 antibody followed by immunoblot analysis with indicated antibodies. (**K**) The structure of MDA5 and its mutants. (**L**) HEK293T cells were co-transfected with empty vector or RNF144B-Myc, TRIM38-Flag, and MDA5-HA. 18 h post-transfection, cell lysates were analyzed by immunoblot using the indicated antibodies. Data information: Data shown are representative of at least three biological replicates, with each data point representing a biological experiment. Error bars are presented as mean ± SD. Statistical significance was determined by Student's t-test. Source data are available online for this figure.

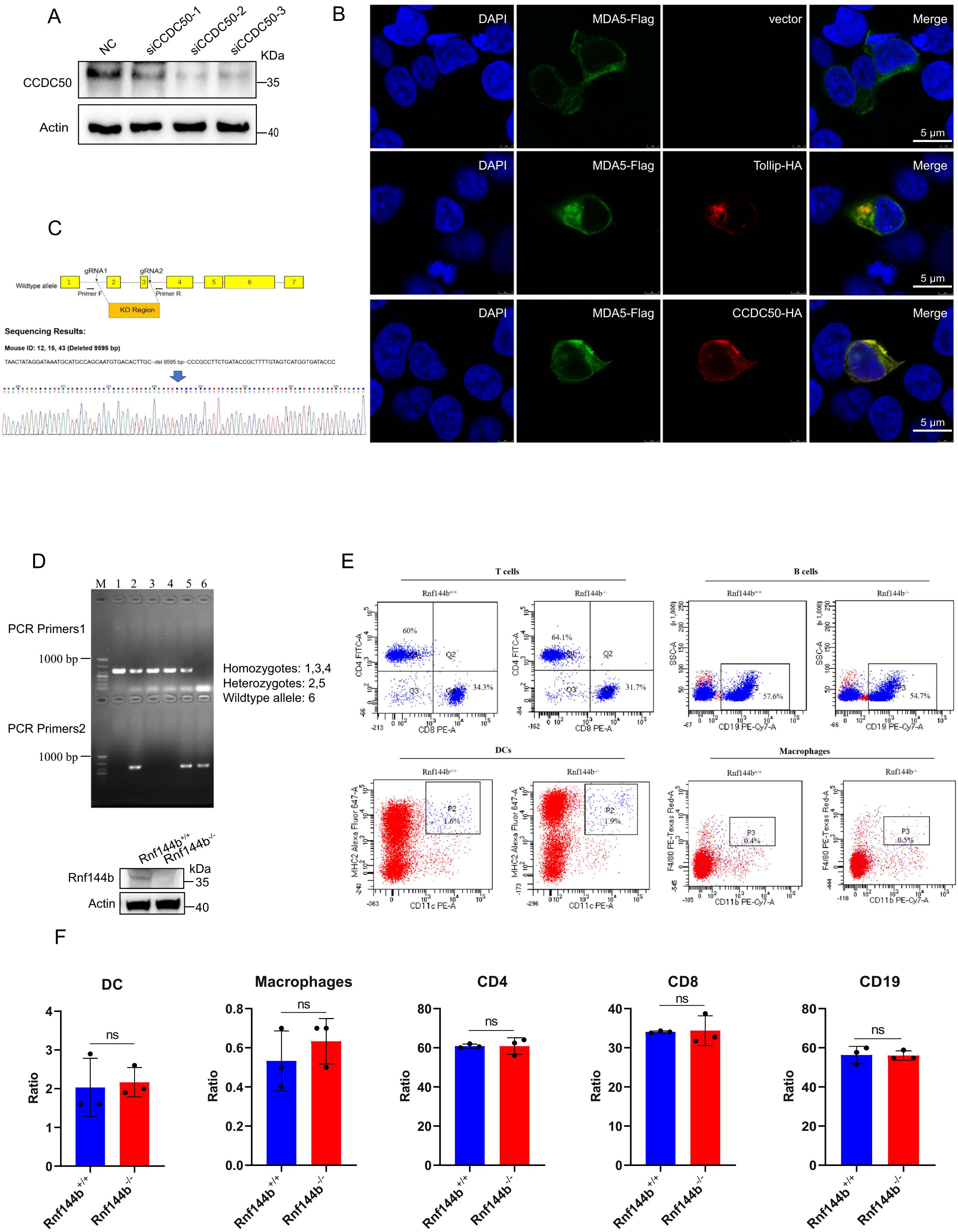
A
NC
siCCDC50-1
siCCDC50-2
siCCDC50-3
KDa
CCDC50
35
Actin
40
B
DAPI
MDA5-Flag
vector
Merge
5 μm
Tollip-HA
CCDC50-HA
C
gRNA1
gRNA2
Wildtype allele
Primer F
Primer R
KO Region
Sequencing Results:
Mouse ID: 12, 15, 43 (Deleted 9595 bp)
D
M 1 2 3 4 5 6
PCR Primers1
1000 bp
PCR Primers2
Homozygotes: 1,3,4
Heterozygotes: 2,5
Wildtype allele: 6
Rnf144b+/+
Rnf144b-/-
Rnf144b
kDa
35
Actin
40
E
T cells
B cells
DCs
Macrophages
CD4 FITC-A
CD8 PE-A
SSC-A
CD19 PE-Cy7-A
MHC2 Alexa Fluor 647-A
CD11c PE-A
F4/80 PE-Texas Red-A
CD11b PE-Cy7-A
60%
34.3%
64.1%
31.7%
57.6%
54.7%
1.6%
1.9%
0.4%
0.5%
F
DC
Macrophages
CD4
CD8
CD19
Ratio
ns
Rnf144b+/+
Rnf144b-/-

 © The Author(s)

Figure EV3. Rnf144b knockout does not affect immune cell development.

(**A**) Immunoblot analysis of CCDC50 expression in HEK293T cells expressing *siRNA* against CCDC50. (**B**) Confocal microscopy analysis of A549 cells co-transfected with MDA5-Flag (green) and Tollip-HA or CCDC50-HA (red). Scale bar, 5 μm. (**C**) Construct strategy diagram of *Rnf144b* gene knockout mouse. (**D**) Verification of knockout efficiency of *Rnf144b* through PCR analysis of tail genome DNA and by immunoblot analysis conducted on heart of WT and KO mice. (**E**) Flow cytometry analysis of percentage of the T cells, B cells, DCs and macrophages in splenocytes of WT and KO mice (6–8 weeks, female). (**F**) The percentage of dendritic cells (DCs), macrophages, CD4, CD8, CD19 positive cells in splenocytes from *Rnf144b*$^{+/+}$ and *Rnf144b*$^{-/-}$ mice were analyzed by flow cytometry. $n = 3$ biological replicates. Statistical significance was determined by two-tailed unpaired Student's t-test. "ns" indicates no significant difference. Data information: Data shown are representative of at least three biological replicates, with each data point representing a biological experiment. Error bars are presented as mean ± SD. Statistical significance was determined by Student's t-test. Source data are available online for this figure.

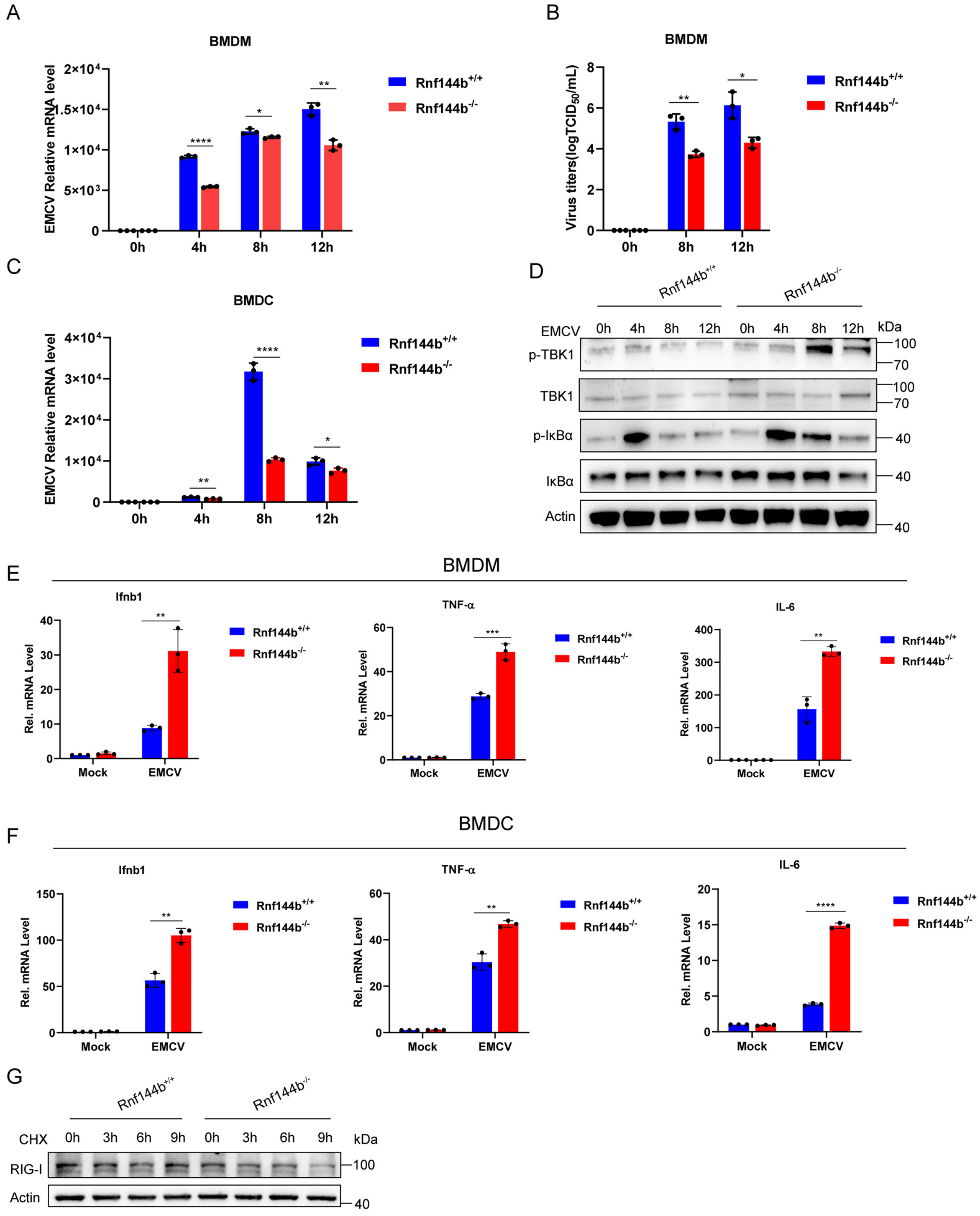
A
BMDM
EMCV Relative mRNA level
2×10⁴
1.5×10⁴
1×10⁴
5×10³
0
0h
4h
8h
12h
****
*
**
Rnf144b+/+
Rnf144b-/-
B
BMDM
Virus titers(logTCID50/mL)
8
6
4
2
0
0h
8h
12h
**
*
Rnf144b+/+
Rnf144b-/-
C
BMDC
EMCV Relative mRNA level
4×10⁴
3×10⁴
2×10⁴
1×10⁴
0
0h
4h
8h
12h
**
****
*
Rnf144b+/+
Rnf144b-/-
D
Rnf144b+/+
Rnf144b-/-
EMCV
0h 4h 8h 12h 0h 4h 8h 12h
kDa
p-TBK1
TBK1
p-IκBα
IκBα
Actin
100
70
100
70
40
40
40
E
BMDM
Ifnb1
TNF-α
IL-6
Rel. mRNA Level
Mock
EMCV
Rnf144b+/+
Rnf144b-/-
**
***
**
F
BMDC
Ifnb1
TNF-α
IL-6
Rel. mRNA Level
Mock
EMCV
Rnf144b+/+
Rnf144b-/-
**
**
****
G
Rnf144b+/+
Rnf144b-/-
CHX
0h 3h 6h 9h 0h 3h 6h 9h
kDa
RIG-I
Actin
100
40

 © The Author(s)

Figure EV4. Rnf144b deficiency enhances anti-RNA viral responses.

(**A**) *Rnf144b*$^{+/+}$ and *Rnf144b*$^{-/-}$ BMDMs were infected with EMCV (MOI = 1), and then cell lysates were analyzed for EMCV replication level by qPCR. *n* = 3 biological replicates. Statistical significance was determined by two-tailed unpaired Student's t-test. ****P = 0.000004, *P = 0.02040, **P = 0.00150. (**B**) *Rnf144b*$^{+/+}$ and *Rnf144b*$^{-/-}$ BMDMs were infected with EMCV (MOI = 1) for indicated time points. The cell supernatant was harvested and analyzed by $TCID_{50}$ assay. n = 3 biological replicates. Statistical significance was determined by two-tailed unpaired Student's t-test. **P = 0.00250, *P = 0.01070. (**C**) *Rnf144b*$^{+/+}$ and *Rnf144b*$^{-/-}$ BMDCs were infected with EMCV (MOI = 1), and then cell lysates were analyzed for EMCV replication level by qPCR. *n* = 3 biological replicates. Statistical significance was determined by two-tailed unpaired Student's t-test. **P = 0.00270, ****P = 0.000064, *P = 0.02330. (**D**) Western blot analysis of the indicated signaling proteins in BMDMs from *Rnf144b*$^{+/+}$ or *Rnf144b*$^{-/-}$ mice infected with EMCV (MOI = 1) for the indicated time periods. (**E**) *Rnf144b*$^{+/+}$ and *Rnf144b*$^{-/-}$ BMDMs were infected with EMCV (MOI = 1). After 12 h, cell lysates were analyzed for Ifnb1, TNF-α, IL-6 mRNA level by qPCR. *n* = 3 biological replicates. Statistical significance was determined by two-tailed unpaired Student's t-test. **P = 0.00340 (Ifnb1), ***P = 0.00080 (TNF-α), **P = 0.00160 (IL-6). (**F**) *Rnf144b*$^{+/+}$ and *Rnf144b*$^{-/-}$ BMDCs were infected with EMCV (MOI = 1). After 12 h, cell lysates were analyzed for Ifnb1, TNF-α, IL-6 mRNA level by qPCR. *n* = 3 biological replicates. Statistical significance was determined by two-tailed unpaired Student's t-test. **P = 0.00140 (Ifnb1), **P = 0.00170 (TNF-α), ****P = 0.000001 (IL-6). (**G**) *Rnf144b*$^{+/+}$ MEFs and *Rnf144b*$^{-/-}$ MEFs were infected with EMCV (MOI = 1). 12 h post-infection, cells were treated by CHX (30 μM) for indicated time points. Protein extracts were used for immunoblot analysis of the endogenous RIG-I protein level. Data information: Data shown are representative of at least three biological replicates, with each data point representing a biological experiment. Error bars are presented as mean ± SD. Statistical significance was determined by Student's t-test. Source data are available online for this figure.

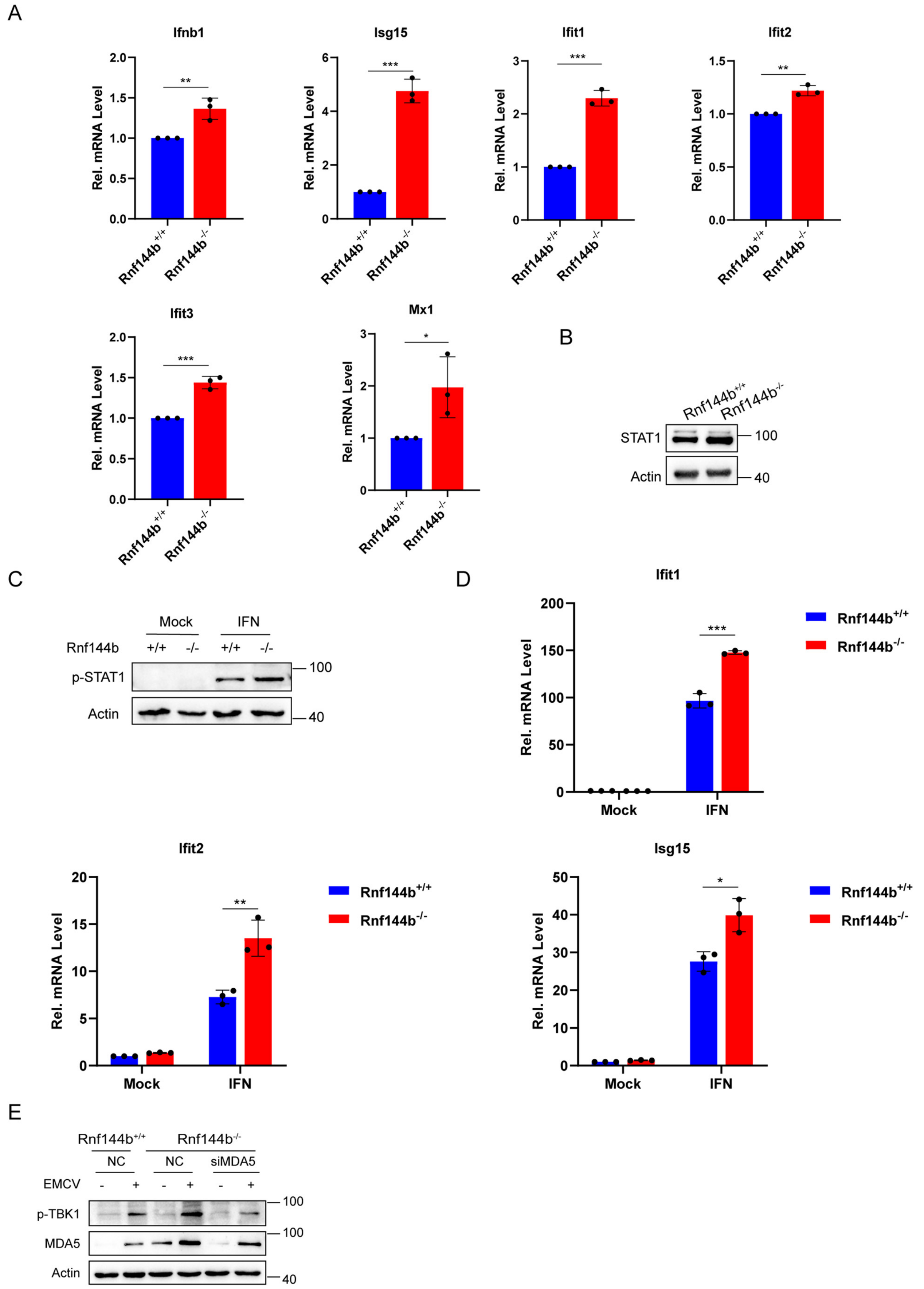
A
Ifnb1
Isg15
Ifit1
Ifit2
Ifit3
Mx1
Rel. mRNA Level
Rnf144b+/+
Rnf144b-/-
B
STAT1
Actin
100
40
C
Mock
IFN
Rnf144b
+/+ -/- +/+ -/-
p-STAT1
Actin
100
40
D
Ifit1
Ifit2
Isg15
Mock
IFN
E
NC
siMDA5
EMCV
p-TBK1
MDA5
Actin
100
40

 © The Author(s)

Figure EV5. RNF144B moderately regulates tonic IFN signaling.

(**A**) *Rnf144b*$^{+/+}$ and *Rnf144b*$^{-/-}$ MEFs cell lysates were analyzed for ISGs mRNA level by qPCR. $n = 3$ biological replicates. Statistical significance was determined by two-tailed unpaired Student's t-test. $^{**}P = 0.0088$ (Ifnb1), $^{***}P = 0.0001$ (Isg15), $^{***}P = 0.00010$ (Ifit1), $^{**}P = 0.0015$ (Ifit2), $^{***}P = 0.0006$ (Ifit3), $^{*}P = 0.0445$ (Mx1). (**B**) *Rnf144b*$^{+/+}$ and *Rnf144b*$^{-/-}$ MEFs cell lysates were analyzed for STAT1 protein level by Western blot. (**C**) Western blot analysis of the indicated signaling proteins in MEFs from *Rnf144b*$^{+/+}$ or *Rnf144b*$^{-/-}$ mice were treated with IFN for 2 h. (**D**) qPCR analysis of ISGs mRNA level in MEFs from *Rnf144b*$^{+/+}$ or *Rnf144b*$^{-/-}$ mice were treated with IFN for the indicated time periods. $n = 3$ biological replicates. Statistical significance was determined by two-tailed unpaired Student's t-test. $^{***}P = 0.00040$ (Ifit1), $^{**}P = 0.00610$ (Ifit2), $^{*}P = 0.01410$ (Isg15). (**E**) *siRNAs* targeting MDA5 or NC were transfected into *Rnf144b*$^{+/+}$ or *Rnf144b*$^{-/-}$ MEFs cells and infected with EMCV (MOI = 1) for 12 h. Cell lysates were used for immunoblot analysis with the indicated antibodies. Data information: Data shown are representative of at least three biological replicates, with each data point representing a biological experiment. Error bars are presented as mean ± SD. Statistical significance was determined by Student's t-test. Source data are available online for this figure.

© The Author(s)

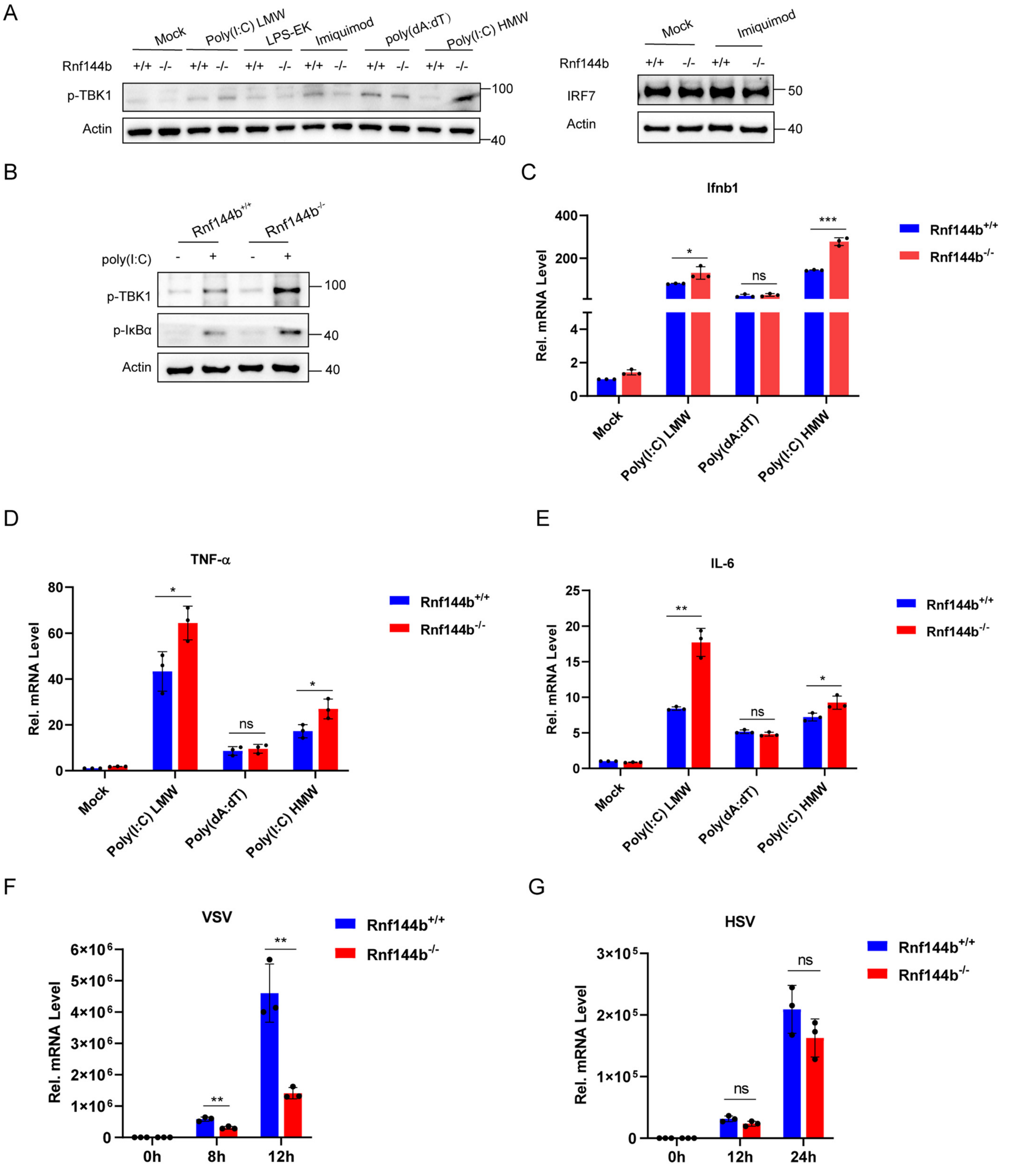
A
Mock
Poly(I:C) LMW
LPS-EK
Imiquimod
poly(dA:dT)
Poly(I:C) HMW
Rnf144b +/+ -/- +/+ -/- +/+ -/- +/+ -/- +/+ -/- +/+ -/-
p-TBK1
Actin
100
40
Mock
Imiquimod
Rnf144b +/+ -/- +/+ -/-
IRF7
Actin
50
40
B
Rnf144b+/+
Rnf144b-/-
poly(I:C) - + - +
p-TBK1
p-IκBα
Actin
100
40
40
C
Ifnb1
Rel. mRNA Level
Rnf144b+/+
Rnf144b-/-
Mock
Poly(I:C) LMW
Poly(dA:dT)
Poly(I:C) HMW
ns
D
TNF-α
Rel. mRNA Level
Rnf144b+/+
Rnf144b-/-
Mock
Poly(I:C) LMW
Poly(dA:dT)
Poly(I:C) HMW
ns
E
IL-6
Rel. mRNA Level
Rnf144b+/+
Rnf144b-/-
Mock
Poly(I:C) LMW
Poly(dA:dT)
Poly(I:C) HMW
ns
F
VSV
Rel. mRNA Level
Rnf144b+/+
Rnf144b-/-
0h
8h
12h
G
HSV
Rel. mRNA Level
Rnf144b+/+
Rnf144b-/-
0h
12h
24h
ns
ns

 © The Author(s)

Figure EV6. RNF144B negatively regulates EMCV induced IFN production and antiviral innate immunity.

(**A**) Western blot analysis of the indicated signaling proteins in MEFs from *Rnf144b*$^{+/+}$ or *Rnf144b*$^{-/-}$ mice were transfected with different ligands for 12 h. (**B**) Western blot analysis of the indicated signaling proteins in MEFs from *Rnf144b*$^{+/+}$ or *Rnf144b*$^{-/-}$ mice were transfected with poly (I:C) for 12 h. (**C**) qPCR analysis of Ifnb1 mRNA level in MEFs from *Rnf144b*$^{+/+}$ or *Rnf144b*$^{-/-}$ mice were transfected with different ligands for 12 h. $n = 3$ biological replicates. Statistical significance was determined by two-tailed unpaired Student's t-test. *$P = 0.04020$, ***$P = 0.00020$. (**D**) qPCR analysis of TNF-α mRNA level in MEFs from *Rnf144b*$^{+/+}$ or *Rnf144b*$^{-/-}$ mice were transfected with different ligands for 12 h. $n = 3$ biological replicates. Statistical significance was determined by two-tailed unpaired Student's t-test. *$P = 0.03210$, *$P = 0.03140$. (**E**) qPCR analysis of IL-6 mRNA level in MEFs from *Rnf144b*$^{+/+}$ or *Rnf144b*$^{-/-}$ mice were transfected with different ligands for 12 h. $n = 3$ biological replicates. Statistical significance was determined by two-tailed unpaired Student's t-test. **$P = 0.0013$, *$P = 0.0311$. (**F**) *Rnf144b*$^{+/+}$ and *Rnf144b*$^{-/-}$ MEFs were infected with VSV, and then cell lysates were analyzed for VSV replication level by qPCR. $n = 3$ biological replicates. Statistical significance was determined by two-tailed unpaired Student's t-test. **$P = 0.00390$ (8 h), **$P = 0.00430$ (12 h). (**G**) *Rnf144b*$^{+/+}$ and *Rnf144b*$^{-/-}$ MEFs were infected with HSV, and then cell lysates were analyzed for HSV replication level by qPCR. $n = 3$ biological replicates. Statistical significance was determined by two-tailed unpaired Student's t-test. "ns" indicates no significant difference. Data information: Data shown are representative of at least three biological replicates, with each data point representing a biological experiment. Error bars are presented as mean ± SD. Statistical significance was determined by Student's t-test. Source data are available online for this figure.

© The Author(s)
